# Genome-wide association and high-resolution phenotyping link *Oryza sativa* panicle traits to numerous trait-specific QTL clusters

**DOI:** 10.1038/ncomms10527

**Published:** 2016-02-04

**Authors:** Samuel Crowell, Pavel Korniliev, Alexandre Falcão, Abdelbagi Ismail, Glenn Gregorio, Jason Mezey, Susan McCouch

**Affiliations:** 1 Plant Biology Section, School of Integrative Plant Science, Cornell University, Ithaca, New York 14853, USA; 2 Department of Biological Statistics and Computational Biology, Cornell University, Ithaca, New York 14853, USA; 3 Department of Information Systems, Institute of Computing, University of Campinas, São Paulo CEP 13083-852, Brazil; 4 Crop and Environmental Sciences Division, International Rice Research Institute, DAPO Box 7777, Metro Manila 1301, Philippines; 5 Plant Breeding, Genetics and Biotechnology Division, International Rice Research Insititute, Los Baños, Laguna 4031, Philippines; 6 Plant Breeding and Genetics Section, School of Integrative Plant Science, Cornell University, 162 Emerson Hall, Ithaca, New York 14853, USA

## Abstract

Rice panicle architecture is a key target of selection when breeding for yield and grain quality. However, panicle phenotypes are difficult to measure and susceptible to confounding during genetic mapping due to correlation with flowering and subpopulation structure. Here we quantify 49 panicle phenotypes in 242 tropical rice accessions with the imaging platform PANorama. Using flowering as a covariate, we conduct a genome-wide association study (GWAS), detect numerous subpopulation-specific associations, and dissect multi-trait peaks using panicle phenotype covariates. Ten candidate genes in pathways known to regulate plant architecture fall under GWAS peaks, half of which overlap with quantitative trait loci identified in an experimental population. This is the first study to assess inflorescence phenotypes of field-grown material using a high-resolution phenotyping platform. Herein, we establish a panicle morphocline for domesticated rice, propose a genetic model underlying complex panicle traits, and demonstrate subtle links between panicle size and yield performance.

## Abstract (web summary)

Panicle architecture is an important determinant of crop yield and a target of selection by plant breeders. Here, Crowell *et al.* combine image-based phenotyping with high-density array-based genotyping to perform a genome-wide association study revealing a number of candidate genes linked to panicle variation in rice.

## Graphical abstract

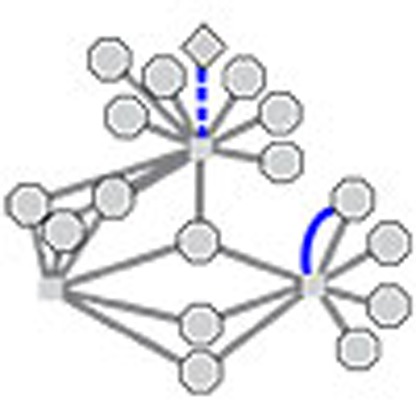

As the bearers of grain, grass inflorescences have been the target of selection for thousands of years^1^. In Asian rice (*Oryza sativa*), a staple crop for billions of people, optimizing rice panicle size and structure represents a challenge for breeders attempting to improve yield potential and maximize grain quality^1^,^2^. Panicle size and branching patterns in rice have increased in complexity throughout domestication and modern breeding; however, when compared to its wild ancestors, it is clear that changes in *O. sativa* panicle architecture have been relatively subtle. Seeds are born on long primary branches that sometimes iterate into secondary and tertiary branches^2^,^3^, and although phenotypes are often variety specific, they are also variable under different environmental conditions^3^,^4^,^5^. Meristematic transitions during panicle development are spatiotemporally regulated, affecting the number and position of rice grains, as well as grain filling rate and seed quality^6^,^7^. Thus, unlike in maize (*Zea mays*), where inflorescences have been selected for extreme divergence into a branchless female cob and a highly branched male tassel^8^, panicles from many modern rice varieties still resemble those from their closest wild relatives, *Oryza rufipogon* and *Oryza nivara*^9^.

Many genes have been cloned relating to rice inflorescence development^6^,^10^, several of which are agronomically important. The *OsLIGULESS1* (*OsLG1*) locus was recently identified as a domestication gene and controls the shift from open to closed panicles^11^. A natural allele of *DENSE AND ERECT PANICLE 1* (*DEP1*) within high-yielding Chinese rice varieties boosts yield potential by pleiotropically reducing panicle internode length (NL), while increasing both primary and secondary branch number^12^. In addition, a major effect allele for *Grain Number 1a* (*GN1a*) significantly increases secondary panicle branching, grain count and yield^13^, and is already being incorporated into breeding pipelines. However, although many studies have examined the role of candidate genes in the reference sequenced variety (Nipponbare) or a few close relatives, panicle architecture has not been characterized in detail across diverse varieties grown in field conditions.

The inbreeding nature of rice and multiple origins of domestication have led to the formation of deep subpopulation structure, which has partitioned genetic and phenotypic variation in the species. *O. sativa* comprises two major varietal groups (sometimes referred to as subspecies), *Indica* and *Japonica*, which can be further divided into five subpopulations (*indica*, *aus*, *tropical japonica*, *temperate japonica* and *aromatic/Group V*)^14^,^15^,^16^. Several genome-wide association studies (GWASs) have confirmed that variation exists both within and between rice subpopulations for important agronomic traits^16^,^17^,^18^,^19^,^20^, including panicle count and panicle length (PL)^16^,^19^. However, low-resolution panicle phenotyping has probably limited the ability to accurately assess genetic architecture of panicle traits^21^.

In this study, we performed GWAS using phenotypes collected with a high-resolution panicle phenotyping platform, PANorama^21^, and a genotypic data set of 700,000 single-nucleotide polymorphisms (SNPs) assayed using a high-density rice array (HDRA)^20^. Unlike previous studies, which focused on collecting a few trait measurements in a large population of accessions^16^,^17^, we collected a large number of panicle and agronomic phenotypes on a targeted population of 242 diverse rice accessions grown under field conditions in the Philippines. Using phenotypic covariates within the GWAS model to examine relationships among traits, we identify a large number of GWAS peaks associated with panicle size, suggest pleiotropic relationships between panicle traits and link several candidate genes to rice panicle development. We validate these associations using quantitative trait loci (QTL) mapping in a recombinant inbred line (RIL) population and demonstrate that panicle traits share subtle relationships with other important agronomic traits, phenotypically and genotypically.

## Results

### Diversity panel selection and population structure

The phenotyping panel in this study contained 242 inbred rice varieties, most of which are tropically or subtropically adapted accessions, and represented germplasm from 60 countries (Supplementary Table 1). Using the Bayesian clustering software *fastStructure*^22^, we calculated varying levels of *K* means (Supplementary Fig. 1a). The *Indica* and *Japonica* varietal groups appear clearly at *K*=2, and at *K*=3 *Indica* further divides into the *indica* and *aus* subpopulations. Using principle component (PC) analysis, we confirmed that the top three PCs account for the *aus*, *indica* and *tropical japonica* subpopulations and explain ∼30% of the genetic variation within our panel (Supplementary Fig. 1b). The optimal number of subpopulations was predicted to be *K*=8, based on model complexity and model component analysis as computed by *fastStructure*^22^. Although *K*=7 or *K*=8 clearly defined variation within and between *indica*, *aus*, *tropical japonica*, *temperate japonica* and admixed accessions, we used the first three PCs as covariates within the GWAS model to control for subpopulation structure (see Methods and Supplementary Fig. 1). These results are consistent with previous studies quantifying the population structure of *O. sativa* and confirm that our panel captures abundant genetic variation in tropical rice germplasm^15^,^17^,^19^,^23^.

### Novel phenotyping reveals rice panicle trait relationships

**Figure 1:**
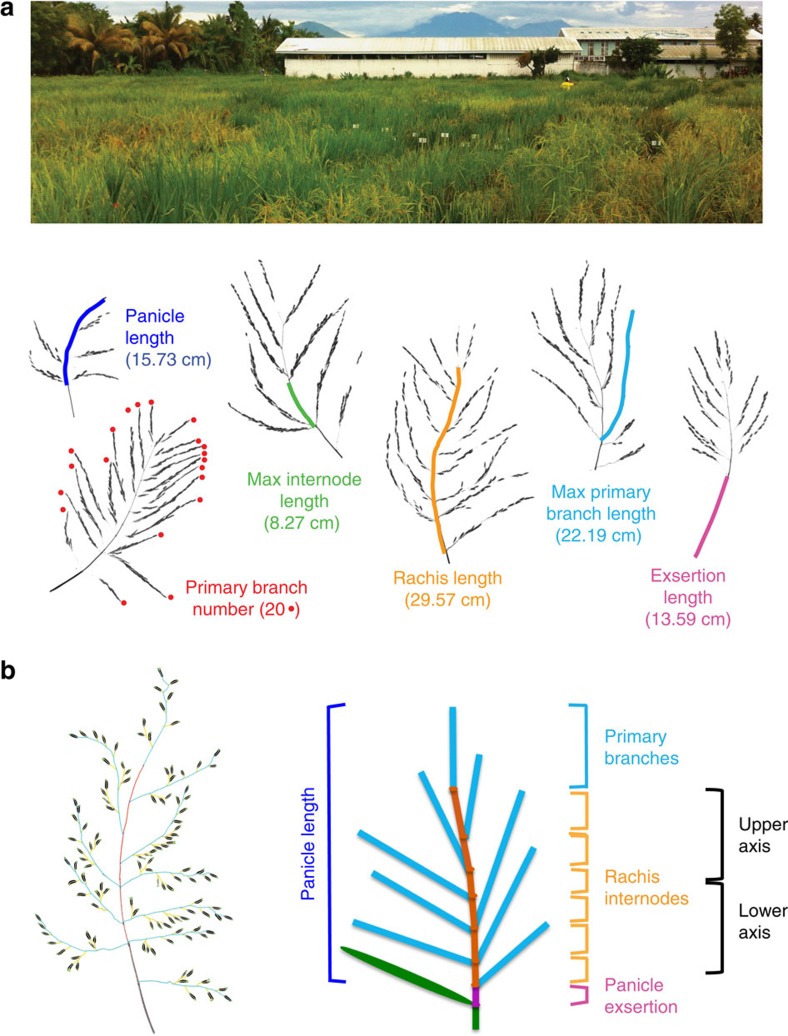
Panicle phenotyping in *O. sativa*. (**a**) A diverse collection of landraces were assessed for panicle and agronomic traits under field conditions. Photographs from different accessions with extreme panicle phenotypes, depicted in relative scale, highlight the range of phenotypic diversity within the panel. (**b**) The PANorama phenotyping platform generates skeletons (left) from panicle images using exact morphological erosions of shapes. The schematic depicts the major classes of phenotypes extracted from panicle skeletons (right).

Using the image skeletonization phenotyping platform PANorama^21^, we measured 49 phenotypes from over 3,400 images of rice panicles collected in the field (Fig. 1a). Width, length and count phenotypes were extracted from images by subdividing panicles into nested measurements of three major panicle traits: primary branches, rachis internodes and the peduncle above the flag leaf ligule, also referred to in rice as panicle exsertion^24^, which is a measurement of the uppermost internode of the panicle-bearing culm (Fig. 1b). Several novel, nested measurements were incorporated into PANorama and are available in an updated version of the open-source software (Methods). We also collected 11 vegetative and reproductive stage phenotypes, including a measurement of flowering time (heading date (HD)). Detailed descriptions of each phenotype are presented in Supplementary Table 2.

As the diversity panel comprises inbred accessions and does not contain heterozygous alleles, it was not possible to calculate true heritabilities for each phenotype; instead, we estimated narrow sense (*h*^2^) heritability by calculating additive+dominance (AD) heritability^25^ and broad sense (H) heritability by calculating repeatability between raw phenotypes (Methods). For some traits, AD and H heritabilities were nearly equivalent, demonstrating the power of image analysis in reducing measurement error (Supplementary Fig. 2). We also calculated genetic correlation among phenotypes and compared it with phenotype × phenotype correlations (see Methods and Supplementary Figs 3 and 4).

**Figure 2:**
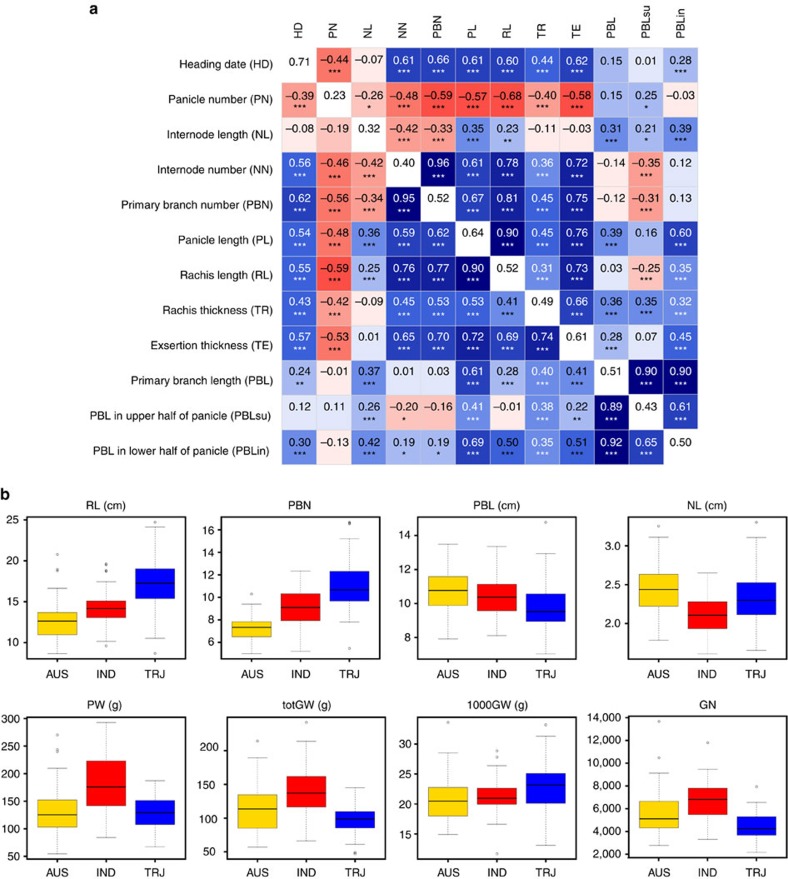
Phenotypic analysis reveals trait relationships and subpopulation characteristics. (**a**) A heatmap depicting Pearson's correlation coefficients between phenotype means (lower triangle) and genetic best linear unbiased predictors (GBLUPs) (upper triangle) for a subset of traits across all varieties within the study. AD heritabilities are located within the diagonal. Trait acronyms are in parentheses. Asterisks indicate significant correlations using a two-tailed *t*-test (**P<0.001 and ***P<0.0001) and *n*=242 varieties. All pairwise comparisons between traits are in Supplementary Figs 3 and 4. (**b**) Phenotypic distributions of panicle and agronomic traits, divided by the *aus* (AUS), *indica* (IND) and *tropical japonica* (TRJ) subpopulations: RL, PBN, PBL, NL, panicle weight (PW), total grain weight (totGW), 1,000GW and grain number (GN). Within boxplots, the bold line represents the median, box edges represent upper and lower quantiles, and whiskers are 1.5 times the quantile of the data. Outliers are shown as open dots. The number of varieties within each subpopulation was as follows: panicle traits (63 *aus*, 84 *indica*, 79 *tropical japonica*) and yield traits (34 *aus*, 51 *indica* and 45 *tropical japonica*).

In general, phenotypic and genotypic correlations among panicle traits mirrored one another and were highly significant (Fig. 2); the median Pearson's correlation coefficient between pairwise phenotypes was *r=*0.4. Increases in width traits such as rachis thickness and exsertion thickness were positively correlated with increased primary branch length (PBL) and primary branch number (PBN). Internode number (NN) and PBN, which estimate meristematic divisions, were positively correlated (Fig. 2a). Groups of sub-traits were tightly correlated, such as the nested phenotypes PBL in the lower and upper halves of the panicle (PBLin versus PBLsu) (Figs 1b and 2 and Supplementary Figs 3 and 4). In short, larger panicles always showed thicker axes, longer branches and higher counts of branches and internodes.

High-resolution phenotyping captured several novel relationships among traits. Inverse correlations between length and count traits have been well documented in rice, especially between panicle number and panicle size (Fig. 2 and Supplementary Figs 3 and 4), highlighting physiological and physical tradeoffs during development^7^,^26^. Although NL had a strong negative correlation with NN (*r*=−0.42), NL was weakly correlated with rachis length (RL) (Fig. 2 and Supplementary Figs 3 and 4), suggesting that increased NN is more important than NL in driving increases in overall panicle size. Surprisingly, PBL and PBN phenotypes were not significantly correlated or showed minimal positive correlation. PBL in the upper (PBLsu) and lower (PBLin) halves of the panicle (Fig. 1b) also had different phenotypic and genetic relationships with PBN and NN, which is consistent with previous evidence for differential protein expression in spikelets on the upper and lower halves of the panicle^27^ (Fig. 2 and Supplementary Figs 3 and 4).

Panicle phenotypes also showed distinct distributions within subpopulations (Fig. 2b). The *tropical japonica* subpopulation had the highest average RL (17 cm) and PBN (11), whereas the *aus* subpopulation had the largest average PBL (11 cm). Historically, many of the highest-yielding varieties have been bred within the *indica* subpopulation^28^,^29^; accordingly, *indica* outperformed both *aus* and *tropical japonica* in several components of yield as follows: panicle weight, total grain weight and grain number. Interestingly, *indica* accessions generally had intermediate-sized panicles, but distinctly had the smallest average NL. Despite varying distributions among phenotypes within the subpopulations, all phenotypic and genetic relationships between panicle traits and yield components were largely the same in the *Indica* and *Japonica* varietal groups (Supplementary Figs 3 and 4). The highest yielding accessions in our panel never had extreme panicle phenotypes.

### Subpopulation structure and flowering effects in GWAS

The inbreeding nature of rice has led to deep subpopulation structure and considerable linkage disequilibrium (LD), which confounds association studies by reducing mapping resolution and increasing type I error^15^,^17^,^30^. Within our panel, average LD does not decay below an *r*^*2*^=0.2 until ∼100 kb in *indica*, 150 kb in *aus* and 400 kb in *tropical japonica* (Supplementary Fig. 5). Further, as noted in previous GWAS in rice and *Arabidopsis*, reproductive phenotypes are particularly susceptible to confounding due to correlations with flowering time and ecological adaptation^16^,^19^,^30^. To address these issues, we used a mixed model to correct for subpopulation structure^31^, integrating the first three PCs as covariates within the model, and performed GWAS across all accessions and within individual subpopulations. In addition, we repeated all analyses with and without use of HD as a phenotypic covariate within the mixed model (see Methods and equation (2)). Detailed association results for every trait, subpopulation and covariate combination are located within the Supplementary Materials (Supplementary Figs 6–65 and Supplementary Data 1 and 2), as well as at www.ricediversity.org.

**Figure 3:**
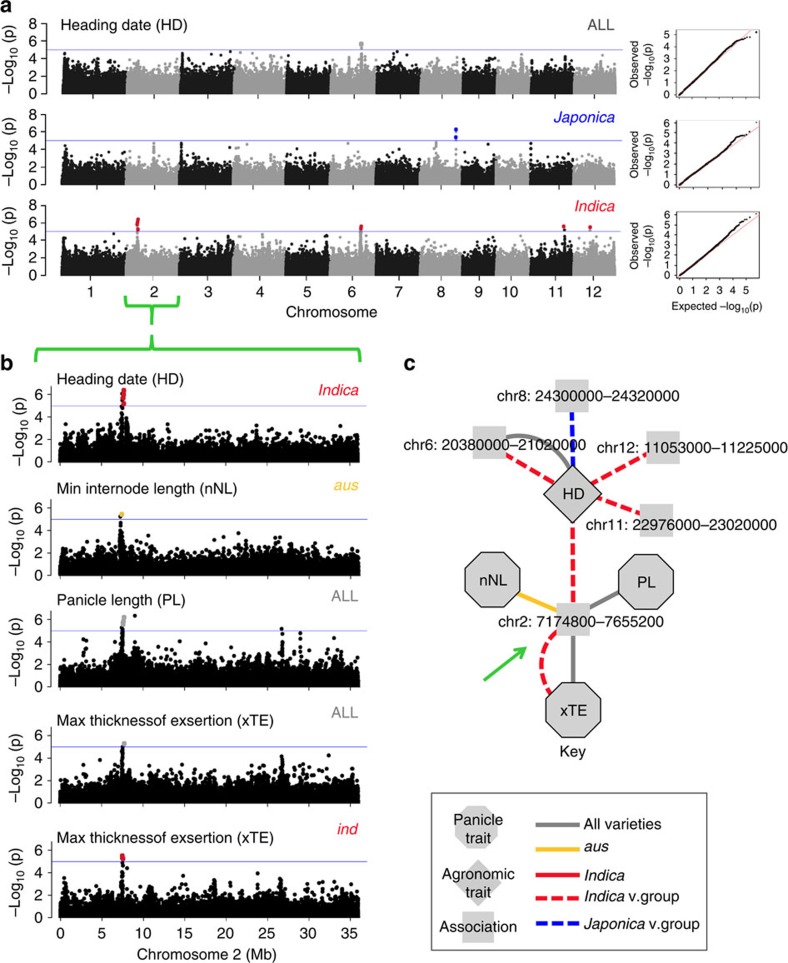
Genome-wide association results for HD. (**a**) Manhattan plots and quantile–quantile plots depicting GWAS results using a mixed model. Associations identified in all accessions, the *Indica* subspecies and the *Japonica* subspecies are depicted in separate panels. The *x* axis depicts the physical location of SNPs across the 12 chromosomes of rice and the *y* axis depicts the −log_10_(*P* value). Significant SNPs with *P*<1 × 10^−5^ are depicted as coloured dots, labelled to match the group in which they were identified (red for *Indica* and blue for *Japonica*). (**b**) Manhattan plots for chromosome 2. The significant SNP associations for HD in the *Indica* subspecies are in LD with significant associations for several panicle traits, depicted in separate panels: minimum NL (nNL) in the *aus* subpopulation (yellow SNPs); PL and maximum exsertion thickness (TE) across all accessions (grey SNPs); and maximum TE in the *indica* subpopulation (*ind*, red SNPs). (**c**) An association network summarizing all Manhattan plots in **a** and **b**. Traits are labelled with acronyms corresponding to **b**. LD blocks are labelled with chromosome number and coordinates. Traits and LD blocks containing significant SNPs are treated as nodes and are connected if an LD block contains a significant association for the trait of interest. The colour and style of the edges connecting the trait and associations indicate, which subpopulation or varietal group in which the association was detected. When multiple edges are present between a trait and LD block, a significant association was detected in more than one GWAS. The green arrow indicates the significant peak on chromosome 2 (**b**), which contains overlapping associations for different types of traits.

GWAS identified five loci associated with the HD phenotype across the panel, all of which overlap with previously identified HD QTL^32^,^33^,^34^,^35^,^36^,^37^ and were detected at low significance (*P*<1 × 10^−6^ or larger) (Fig. 3a). Only one of the peaks, a region on chromosome 2 in the *Indica* varietal group, overlapped with associations for the panicle traits minimum NL, PL and maximum exsertion thickness (Fig. 3b). When HD was used as a phenotypic covariate, GWAS peaks for panicle traits on chromosome 2 were attenuated or eliminated (Supplementary Figs 9, 12 and 53), suggesting that panicle phenology associated with this locus is largely explained by variation in flowering time. In addition, use of the HD covariate reduced the number of significant SNPs associated with panicle traits throughout the genome (Table 1). The effect was most striking within the *tropical japonica* subpopulation, although a few *tropical japonica* accessions within the panel are from subtropical regions and may be less adapted for growth in the irrigated tropics (Supplementary Table 1). Many significant SNPs were eliminated from two peaks within the pericentromeric region of chromosome 8 (∼45 SNPs in *tropical japonica* and 75 when mapping with all accessions). In addition, many were from PBN traits (∼130 SNPs), which generally showed improved quantile–quantile plots with the use of the HD covariate (Supplementary Figs 34–48).

These results confirm established pleiotropic relationships between flowering time and inflorescence architecture in rice^38^,^39^,^40^. However, this is not the whole story; including the HD covariate in the mixed model eliminated SNPs associated with several phenotypes, but many SNPs associated with length and width traits were not eliminated and occasionally showed increases in significance (Table 1 and Supplementary Figs 10d, 30d, 50d, and 51d). Having properly controlled for the effects of flowering time, we investigated the remaining significant loci associated with panicle phenotypes, which could represent candidates for breeders hoping to tweak panicle architecture to optimize yield performance in the tropics. Thus, unless otherwise noted, all results discussed within the following sections were generated using the HD covariate in the GWAS model.

### Visualization of complex trait relationships using networks

To compare association results across many traits, we constructed ‘association networks' using the programme Cytoscape^41^. Briefly, significant SNPs were binned into peaks based on physical map position, using a sliding window defined by association significance level and local LD (see Methods and Supplementary Data 3). We constructed networks in which traits and peaks were treated as nodes, connected by an edge only when the trait showed significant associations within a given region of the genome. Of the five significant peaks for HD detected in the genome (Fig. 3a), only the peak on chromosome 2 overlapped with panicle traits (Fig. 3b,c). Association networks provided a visual summary of how peaks were distributed across different traits and allowed us to quantitatively identify regions of the genome associated with multiple phenotypes (Supplementary Data 4).

### GWAS links panicle trait variation to numerous loci

When mapping across all accessions within the panel using the HD covariate, we detected 496 significant SNP associations clustered under 256 peaks located on all 12 chromosomes (Table 1). Many SNPs had small-to-intermediate significance levels (*P*<1 × 10^−6^ or larger); only 18 SNPs showed a *P*<1 × 10^−7^ and the most significant panicle trait association was for PBL s.d. (*P*=8.2 × 10^−9^; Supplementary Fig. 27). These results suggest that panicle morphology is determined by many genes, each with small effect.

**Figure 4:**
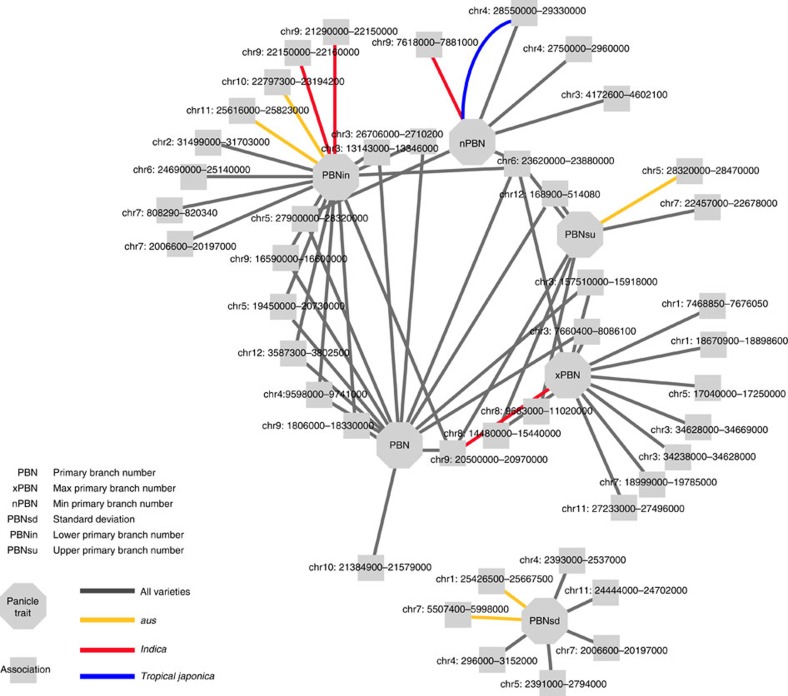
Genome-wide association links numerous loci to variation in panicle traits. An association network constructed using PBN traits. Traits and LD blocks containing significant SNPs (*P*<1 × 10^−5^) are treated as nodes and are connected if an LD block contains a significant association for the trait of interest. LD blocks are labelled with chromosome number and coordinates. The colour and style of the edges connecting the trait and associations indicate which subpopulation or subspecies in which the association was detected. When multiple edges are present between a trait and LD block, significant associations were detected in multiple populations. Trait abbreviations are as follows: PBN, primary branch number; PBNin, (PBN in lower half of panicle; PBNsu, PBN in upper half of panicle; nPBN, minimum PBN; PBNsd, PBN s.d.; xPBN, maximum PBN.

Although nested phenotypes often shared the same peaks, we detected an increased number of peaks by dividing panicles into sub-traits. For example, we identified 14 significant peaks when mapping for average PBN across all accessions (Fig. 4). Mapping with maximum PBN, minimum PBN and s.d. of PBN (PBNsd) identified an additional 15 peaks on 7 chromosomes that were not detected when mapping with (PBN) (Fig. 4 and Supplementary Figs 34–39). As described above, previous research demonstrated that spikelets located on lower versus upper panicle branches had differential regulation and expression of proteins^27^. Mapping for PBN in the lower and upper halves of the panicle separately (Fig. 1b) identified additional five peaks not observed among other primary branch traits (Fig. 4). We also detected an increased number of associations when other traits were subdivided into multiple phenotypes (Supplementary Figs 17–29, 40–48 and 66, and Supplementary Data 4). These results suggest that partitioning a trait into multiple sub-traits minimizes the variance among raw values, which in turn maximizes the ability to detect differences for that sub-trait. This increases the power of GWAS to detect significant associations. Thus, although clusters of related measurements are highly correlated with one another morphologically and genetically (Figs 2 and 4, and Supplementary Figs 3 and 4), separating a trait into nested phenotypes appears to resolve the location of small-effect QTL in unique regions of the genome^21^.

### Subpopulation-specific panicle trait associations

Performing GWAS within individual subpopulations identified an additional 107 significant peaks (Table 1). When comparing significant peaks using association networks, we noted that certain types of traits showed enrichment for subpopulation-specific SNPs. For example, although we only identified 10 subpopulation-specific peaks for PBN traits (Fig. 4), we identified 23 peaks for PBL traits (Supplementary Fig. 66). Strikingly, no two subpopulations had a significant peak for the same trait within the same region of the genome (Supplementary Data 4). Only one region of the genome contained peaks for panicle traits identified in two separate subpopulations (Supplementary Fig. 67). Both these observations have been made in rice for other phenotypes, including the traditional breeding phenotype PL (Fig. 1b)^15^,^16^,^17^,^19^. The genetic heterogeneity within *O. sativa* drives trait variation at the subpopulation and subspecies level, and probably explains the phenotypic differences we observe for each subpopulation (Fig. 2). These results also suggest that a sizable portion of the genetic variation responsible for panicle morphology remains isolated within individual subpopulations.

### Assessing pleiotropy between panicle traits

**Figure 5:**
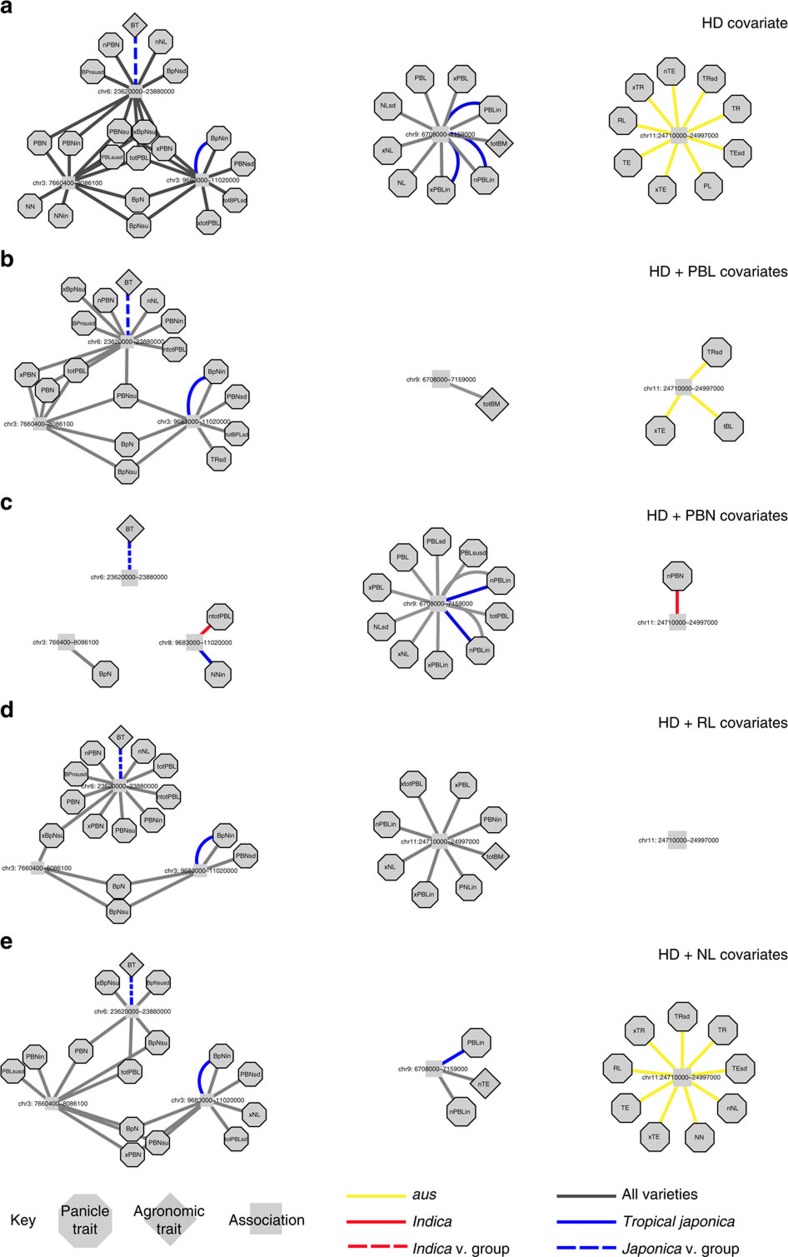
Panicle covariates dissect genomic regions containing many associations for different types of traits. Peaks detected using (**a**) the HD covariate within the GWAS mixed model; (**b**) HD and PBL covariate; (**c**) HD and PBN covariate; (**d**) HD and RL covariate; or (**e**) HD and NL covariate. Association networks demonstrate that clusters of related traits frequently have overlapping associations within LD blocks. Traits and LD blocks containing significant SNPs (*P*<1 × 10^−5^) are treated as nodes and connected if an LD block contains a significant association for the trait of interest. LD blocks are labelled with chromosome number and coordinates. The colour and style of the edges connecting the trait and associations indicate the subpopulation or subspecies in which the association was detected. When multiple edges are present between a trait and LD block, a significant association was detected in more than one GWAS. To simplify networks, acronyms are used. Agronomic traits: booting (BT) and total shoot biomass (totBM). Count traits: PBN, NN and number of branches per internode (BpN). Length traits: PL, RL, PBL and NL. Width traits: thickness of rachis (TR) and thickness of exsertion (TE). In addition to overall trait averages, sub-traits are depicted using acronyms with prefixes and/or suffixes: maximum (x−), minimum (n−), s.d. (−sd), lower (−in) and upper (−su). For example, maximum PBL in the lower half of the panicle (xPBLin).

To determine whether relationships between different types of traits (length, width and count) were the result of linkage or pleiotropy, we constructed association networks using every phenotype in the panel. We observed 92 regions of the genome with significant SNPs for more than 1 trait; 10 regions had associations for 8 or more panicle traits (Supplementary Data 4). In most cases, the regions associated with more than one trait were identified when mapping across all accessions in the panel; when mapping within a single subpopulation, the same region was associated with just one or a few traits (Fig. 5a). A careful examination of allele frequencies demonstrated that in most cases, the reason for this distribution was the presence of subpopulation-specific alleles that remained significant when all subpopulations were considered together (Supplementary Data 1 and 2). However, we occasionally detected subpopulation-specific associations for multiple traits at one genomic address; an unusual region on chromosome 11 within the *aus* subpopulation contained associations for nine length and width traits (Fig. 5a).

In general, the same types of traits had overlapping peaks within a given region of the genome. For example, peaks on chromosomes 3 and 8 were associated with PBN and NN traits across all subpopulations, a peak on chromosome 9 was associated with NL and PBL traits, and the peak on chromosomes 11 identified in *aus* (mentioned above) was associated with overall size traits such as RL, PL and width traits (Fig. 5a). This suggested that panicle traits with a shared morphological origin, expansion of tissue versus division of meristems, are more likely to be co-inherited.

To test for pleiotropy among panicle traits, we repeated GWAS and sequentially incorporated different panicle traits as a second phenotypic covariate (alongside HD) within the mixed model: PBL, PBN, RL or NL (see Methods, equation (3) and Supplementary Data File 5–10). We noted several patterns common to all panicle covariate runs. Although the total number of significant peaks did not drastically change (Supplementary Table 3), peaks identified when mapping across all accessions tended to lose associations with some phenotypes or disappear entirely (Fig. 5 and Supplementary Figs 68–71) and the number of subpopulation-specific peaks increased (Supplementary Table 3 and Supplementary Figs 68–71). In addition, the majority of peaks overlapped with peaks from the HD covariate run (Supplementary Table 4).

Interestingly, covariates had different impacts on associations at individual peaks, which mimicked the genetic and phenotypic relationships quantified above (Fig. 2 and Supplementary Figs 3 and 4). The PBL and PBN covariates affected peaks in opposite ways. The PBL covariate eliminated the peak for branch length and NL traits on chromosome 9, yet did not eliminate associations for PBN traits on chromosomes 3, 6 or 8 (Fig. 5b). In contrast, the PBN covariate had little effect on the chromosome 9 peak, yet largely eliminated peaks on chromosome 3, 6, 8 and 11 for branch number traits, NN traits and composite traits such as PL or total PBL (Fig. 5c). Although the RL covariate eliminated the significant peak in *aus* on chromosome 11, it did not eliminate the peaks on chromosomes 3, 6, 8 or 9 (Fig. 5d); this indicates that genetic variation at certain loci may have an impact on specific stages of panicle development spatiotemporally. Finally, the NL covariate had an impact on peaks similar to the PBL covariate, rather than the RL covariate, indicating that certain peaks may affect NL without affecting RL or overall size of the panicle (Fig. 5d). Taken together, these results suggest that specific panicle traits are highly correlated and/or pleiotropic, at least in the environment tested in this study. The way in which panicle covariates ubiquitously had an impact on associations for phenotypes at other loci is also indicative (Supplementary Figs 68–71); small differences in individual traits are likely to be the result of genetic variation that has an impact on a single compensatory network of developmental genes that drives inflorescence morphology as a whole.

### Relationships among panicle and agronomic trait associations

**Figure 6:**
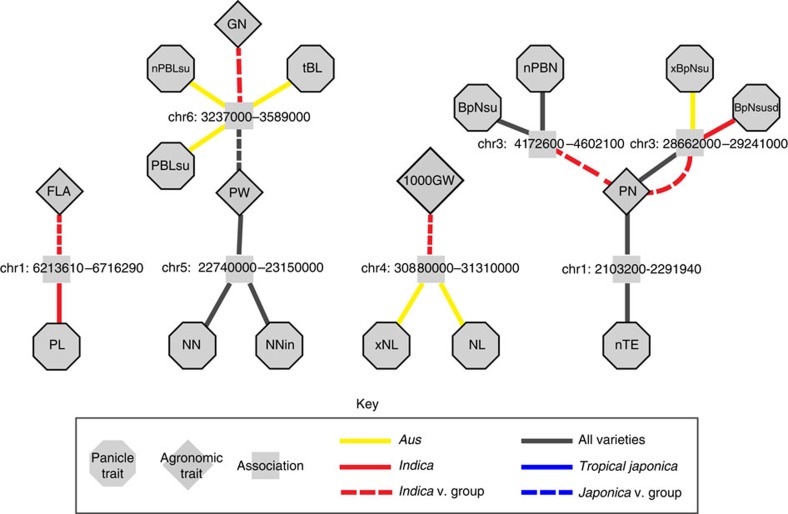
Genomic regions containing associations for panicle and agronomic traits when using the HD covariate. Association networks where traits and LD blocks containing significant SNPs (*P*<1 × 10^−5^) are treated as nodes and are connected if an LD block contains a significant association for the trait of interest. LD blocks are labelled with chromosome number and coordinates. The colour and style of the edges connecting the trait and associations indicate the subpopulation or varietal group in which the association was detected. When multiple edges are present between a trait and LD block, a significant association was detected in more than one GWAS. To simplify networks, acronyms are used. Agronomic traits: booting (BT) and total shoot biomass (totBM). Count traits: PBN, NN and number of branches per internode (BpN). Length traits: PL, RL, PBL, NL and tip branch length (tBL). Width traits: thickness of rachis (TR) and thickness of exsertion (TE). In addition to overall trait averages, sub-traits are depicted using acronyms with prefixes and/or suffixes: maximum (x−), minimum (n−), s.d. (−sd), lower (−in) and upper (−su). For example, maximum PBL in the lower half of the panicle (xPBLin). Agronomic traits: flag leaf area (FLA), panicle weight (PW), grain number (GN), 1,000GW and panicle number (PN).

As observed in previous studies, we detected several regions of the genome that contained significant associations for both panicle and agronomic traits. In some cases, agronomic traits were vegetative; for example, a peak on chromosome 1 was associated with PL and flag leaf area, and a peak on chromosome 9 for total shoot biomass overlapped with peaks for many length phenotypes (Fig. 6). Several yield performance traits, such as panicle weight, grain number and 1,000-grain weight (1,000GW) had overlapping peaks with different types of panicle traits as follows: NN, branch length and NL, respectively. Unlike the associations we observed when comparing panicle phenotypes, panicle and agronomic traits never shared exactly the same significant SNPs^19^ (Supplementary Data 1,2 and 5–8); rather, significant SNPs were often closely linked within the same LD block (<100 kb). In addition, the use of panicle trait covariates within the mixed model did not eliminate the most significant yield associations (Supplementary Fig. 72). Rather, panicle covariates altered which panicle traits overlapped with agronomic traits. This could suggest that the genetic networks governing agronomic traits operate independently, at least in part, of those responsible for variation in panicle traits detected in the field.

### Biparental mapping and candidate gene analysis

**Figure 7:**
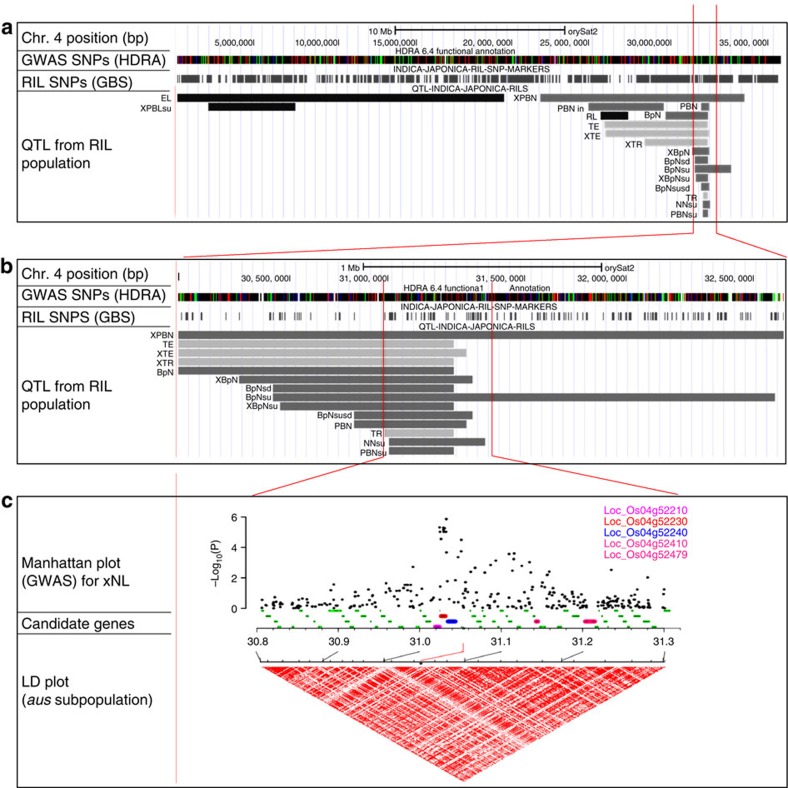
Mega-locus on chromosome 4 associated with panicle and yield traits. (**a**) Physical map of rice chromosome 4 showing the position of SNPs segregating in the GWAS panel (HDRA SNPs) and in the RIL population (genotyping by sequencing (GBS) SNPs). Grey bars indicate the position of QTL identified in the RIL population. Trait acronyms are as follows: 1000GW, PBN, NN, RL, PBL, NL, rachis thickness (TR), exsertion thickness (TE), exsertion length (EL), and number of branches per internode (BpN); sub-traits are depicted using acronyms with prefixes and/or suffixes: maximum (x−), minimum (n−), s.d. (−sd), lower (−in) and upper (−su). For example, PBN in the upper half of the panicle: PBNsu. (**b**) Zoom-in of 2.5 Mb region of chromosome 4 showing QTLs for panicle traits. (**c**) Zoom-in of 0.5 Mb region of chromosome 4 showing Manhattan plot, indicating position of GWAS-QTL associated with maximum NL (xNL) and 1000GW. An LD heatmap (D') is depicted in red. Five candidate genes are highlighted as coloured bars below the Manhattan plot: *OsKS3=*LOC_Os04g52210, *OsKS1=*LOC_Os04g52230, *OsKS2=*LOC_Os04g52240, *OsMADS31=*LOC_Os04g52410 and *NAL1=*LOC_Os04g52479.

To further assess the genetic architecture of panicle traits, QTL mapping was performed using 168 recombinant inbred lines (RILs) grown in a second environment (see Methods section). By subdividing traits and mapping with nested panicle phenotypes^21^, we were able to dissect several large QTL into overlapping small-effect QTL with varying sizes, significance levels and peak positions; these results mirrored the increased number of GWAS associations detected when mapping with sub-traits (Fig. 4). In total, we identified 129 QTL for panicle phenotypes, 7 for HD and 2 for panicle number (Supplementary Table 5). Strikingly, we observed QTL that overlapped with significant GWAS peaks on 11 out of 12 chromosomes (Supplementary Figs 83–93). Although biparental QTL generally encompassed more than one significant GWAS peak (due to lower resolution of QTL mapping versus GWAS), in several cases the QTL mapping resolution gained by using sub-traits in the RIL population allowed us to narrow in on a single GWAS peak (Fig. 7 and Supplementary Figs 83, 85–87,90 and 91).

Many genes involved in rice development have been cloned and characterized^6^,^10^. To leverage this resource, we assembled a list of 319 *a priori* candidate genes (Supplementary Table 6), roughly half of which have been described molecularly, to determine whether any known genes mapped within the expected LD surrounding our GWAS peaks. Based on the most stringent co-localization criteria, we identified ten candidate genes located within 30 kb or less of significant GWAS SNPs (interval of 1–3 genes) (Table 2). Seven of the candidate genes were associated with hormone signalling cascades^42^,^43^,^44^,^45^,^46^,^47^,^48^,^49^,^50^. Using the database RiceFREND^26^, we confirmed that eight of the ten candidates shared a gene co-expression network with at least one other candidate from our *a priori* gene list (Supplementary Note, Supplementary Figs 73–82 and Supplementary Data 11). In addition, five of the ten *a priori* candidate genes identified by GWAS were located within QTL identified by the biparental RIL population (Table 2 and Supplementary Figs 83, 84, 86, 87 and 92).

Interestingly, the traits associated with RIL–QTL were sometimes different than the traits associated with GWAS–QTL in the same region. For example, we identified several RIL–QTL for an agronomic trait that overlapped with a GWAS–QTL for a panicle trait, or vice versa (Supplementary Figs 83–85,87 and 91–93). To characterize the relationship between panicle traits and yield components, we examined a region on chromosome 4 that was simultaneously associated with a suite of biparental QTLs for nested panicle phenotypes, a GWAS peak for maximum NL detected in the *aus* subpopulation and a GWAS peak for 1,000GW detected in the *Indica* varietal group (*indica*+*aus* subpopulations) (Fig. 7). Within a 300-kb region containing overlap between 14 biparental QTLs and two GWAS peaks, we observed five *a priori* candidate genes: a cluster of three tandemly linked rice ent-kaurene synthase genes (*OsKS1*, *OsKS3* and *OsKS3*)^45^, a rice *MADS Box* gene (*OsMADS31*)^44^ and *NARROW LEAF1* (*NAL1*)^28^,^51^,^52^,^53^,^54^ (Fig. 7c and Supplementary Data 2). Zooming into the region, it became clear that the *OsKS* gene family fell directly underneath the most significant GWAS SNPs for both maximum NL and 1,000GW (Supplementary Fig. 94), well within the region of intersection between the RIL-QTLs and the GWAS-QTLs. The RiceFREND database^26^ also demonstrated that *OsKS1* is co-expressed with sucrose synthase (Supplementary Fig. 76). However, although *OsMADS31* and *NAL1* were further removed from the most significant GWAS–SNPs, neither GWAS nor biparental QTL mapping provided enough resolution to clearly identify which gene(s) are responsible for the phenotypes we observe, nor to distinguish whether multiple genes were acting combinatorially to generate a ‘synthetic' QTL of larger effect^55^. Linkage between multiple candidate genes within a 300-kb region associated with diverse panicle traits and the yield component 1,000GW warrants further investigation to unravel the potential breeding significance of this region of the rice genome.

## Discussion

Although panicles are the grain-bearing organs in rice, breeders have an incomplete picture of the genetic architecture underlying panicle development in different subpopulations and of the relationships between panicle traits and yield performance. Previous GWAS and QTL studies have collected only a limited number of panicle phenotypes and/or assessed plants grown in controlled environments^18^,^19^,^21^. In addition, owing to subpopulation structure and extensive LD in *O. sativa*, many studies have evaluated large panels of diverse germplasm to increase GWAS mapping resolution^16^,^17^,^19^,^23^. Although these approaches improve resolution, they considerably confound panicle trait associations with loci responsible for flowering and ecological adaptation^30^,^56^,^57^.

We demonstrate that with proper controls for subpopulation structure and flowering, it is possible to detect GWAS peaks associated with reproductive phenotypes and identify SNPs closely associated with *a priori* candidate genes from pathways known to regulate rice plant architecture (Table 2). The success of our GWAS was undoubtedly due to a combination of phenotypic resolution and use of a high-quality SNP data set from the HDRA^20^. Thus, for breeders and biologists interested in quantifying the genetic architecture of traits, medium-size populations can be used to detect both large- and small-effect loci in the field, provided dense marker data are complemented with precise phenotyping methodologies.

We document quantitative variation among panicle traits within and across rice subpopulations and suggest that, in contrast to maize, no one aspect of panicle size or morphology has been severely aggrandized or optimized during domestication. Instead, panicle architecture appears to comprise multiple correlated components of relatively small effect that interact and compensate for one another during development. The number of panicle associations we detect is consistent with the number of genes reported to be expressed during rice^58^,^59^ and maize inflorescence development^60^, and we hypothesize that combinations of alleles not detected by GWAS in this study may further enhance subpopulation-specific morphology. Thus, although the underlying genetics governing rice panicle traits are highly subpopulation-specific, overall phenotypic outcomes appear surprisingly similar—even to wild relatives^9^.

Gene expression levels have been shown to directly affect flowering time in rice^61^. Our ability to detect distinct associations when mapping for nested traits such as those from lower versus upper panicle traits suggests that we may be capturing genes with spatiotemporal expression differences. Peaks independently associated with length or count traits, or that were preferentially eliminated by certain panicle covariates, are particularly promising for breeders; they may tag genetic variation that can be targeted for selection to tweak individual traits without affecting other aspects of panicle architecture. In keeping with this perspective, we note that the highest yielding *indica* accessions in our panel have characteristically intermediate panicle phenotypes and the smallest NL (Fig. 2), a trait linked to rice yield performance^12^. However, deep genome-wide differentiation between subpopulations means that a gene can have different phenotypic consequences in different genetic backgrounds^18^,^19^. Thus, it may only be possible to predict the impact of genetic variation associated with panicle traits when operating within a subpopulation, although recombination across subpopulations provides opportunity to drive transgressive phenotypes with extraordinary outcomes.

Within the public breeding community, there have been two major initiatives over the past 70 years to boost yield by optimizing independent phases of rice development. The first occurred during the Green Revolution, when breeders successfully leveraged a large-effect allele of *SEMIDWARF1* (*SD1*) and optimized vegetative architecture without drastically changing panicle phenotypes^42^; our detection of discrete associations for agronomic traits unaffected by panicle trait covariates reconfirm that critical rice genes may operate only during specific stages of development^6^. The second breeding initiative, development of the ‘New Plant Type' ideotype, attempted to boost yield by simultaneously selecting for increased panicle size (sink) and photosynthetic capacity (source)^62^. This was done using introgression of genomic regions associated with large panicles and low-tillering from *tropical japonica* into *indica* varieties^63^,^64^.

Given the quantitative nature of panicle development that we detected within and between *indica* and *tropical japonica*, it is not surprising that the New Plant Type initiative successfully generated large panicle phenotypes but failed to achieve desired combinations of sink and source traits that generate high-yielding varieties^63^,^64^. The sheer number and non-additive behaviour of loci contributing to panicle morphology suggest that physiologically optimizing panicle architecture for grain filling and yield *per se* will probably involve managing a highly interactive network or trait complex. This will require integrating quantitative tools and strategies, such as those used in this study, into model-based crop improvement pipelines incorporating genomic selection. That being said, targeted introgression of key genomic regions encompassing specific combinations of beneficial alleles in the form of a complex or ‘synthetic' QTL holds great promise as a strategy for coordinately improving the suites of traits that are essential for resilience and yield improvement^55^.

The *NAL1-OsKS1* megalocus on chromosome 4 is of particular interest for rice breeding because it is rich in allelic variation and multiple studies have demonstrated yield improvement using introgression of Japonica alleles into Indica varieties^28^,^51^,^52^,^54^. These findings raise interesting questions about the value of subpopulation-specific allele introgression, genotype by genotype interaction and the role of linked genes that hitchhike along with a target introgression^55^. *NAL1* is hypothesized to have been a target of selection during rice domestication^54^ and is known to encode a plant-specific protein involved in control of the cell cycle, cell division and polar auxin transport, with pleiotropic effects on vascular patterning, flag leaf area, leaf chlorophyll content, photosynthetic efficiency, panicle size, panicle branching, spikelet number and overall plant architecture^28^,^51^,^52^,^53^,^54^. Less is known about the potential contribution of *OsKS1*, which is tandemly linked with two of its homologues (*OsKS2* and *OsKS3*) in the region and catalyses an early step in gibberellin biosynthesis, with mutant alleles leading to dwarfing phenotypes in both vegetative and reproductive tissue^45^, or *OsMADS31*, which is ubiquitously expressed throughout panicle development and the first stages of seed formation^44^. The opportunity to optimize linked arrays of alleles that are co-inherited in applied rice breeding represents an exciting new research horizon.

The phenotyping methods and mapping resolution presented in this study provide us with the ability to hypothesize the existence of numerous complex QTL that merit further dissection using expression QTL mapping^65^ or molecular studies using targeted genome editing. By identifying, understanding and integrating subpopulation-specific variation using a combination of approaches, breeders may one day close the gap between panicle development and yield optimization in rice.

## Methods

### GWAS germplasm selection

A collection of 1,568 accessions representing the five major subpopulations in *O. sativa* was recently genotyped for 700,000 SNPs using an HDRA^20^. We wished to maximize the diversity among rice accessions with HDRA genotypes and minimize confounding effects relating to poor adaptation for growth in the tropics. Most accessions were selected from three rice subpopulations (63 *aus*, 84 *indica*, 79 *tropical japonica*, 11 admixed *Japonica*, 3 *temperate japonica* and 2 admixed accessions). Detailed information regarding accessions is located within Supplementary Table 1.

### RIL population

The RIL population used in this study was originally developed from a wide cross between IR64 (*Indica*) and Azucena (*Japonica*), followed by single-seed descent in the greenhouse at Institut de Recherche pour le Développement in Montpellier, France. Both IR64 and Azucena were included the diversity panel used for GWAS (Supplementary Table 1). As described previously, the RILs were genotyped using genotyping by sequencing for 30,984 SNPs at Cornell University^66^.

### Population structure

The PC analysis was conducted using the svd() function in R^67^ (version 3.1.0), calculated using SNPs present in all accessions. The Bayesian clustering programme *fastStructure* was used to calculate varying levels of *K* (*K*=1–10) and the command chooseK.py was used to identify the model complexity that maximized the marginal likelihood (*K*=8). Supplementary Fig. 1a was generated using the programme distruct^68^. Genome-wide LD was estimated using pairwise *r*^2^ between SNPs, which was calculated using the --r2 --ld-window 99999 --ld-window-r2 0 command in PLINK^69^ (version 1.07).

### Phenotyping details

For the GWAS diversity panel, three replications of each variety were evaluated during the 2013 dry season (January–May) at the International Rice Research Institute in Los Baños, Philippines in a randomized block design under flooded paddy conditions. Each replication consisted of a two-row plot 4.6 m in length, with 0.2 m between plants and 0.3 m between rows. Panicle traits, HD and booting date were collected on all 242 accessions within the panel. All other yield components were collected on 136 randomly sampled accessions from the *indica*, *aus* and *tropical japonica* subpopulations (Supplementary Table 1). Detailed descriptions of all phenotypes, acronyms and measurement methods are presented in Supplementary Table 2. Raw phenotypes and trait averages used for genetic mapping are stored in Supplementary Data 12 and 13.

Three plant replicates for each of the 168 RILs used for QTL mapping were grown in the Guterman greenhouse in Ithaca, New York, during summer 2012 using a pseudo-randomized block design that accounted for extreme plant height differences^21^; the population is an expansion in both the number of lines and number of phenotypes over that described in Crowell *et al.*^21^ HD was measured as the point at which the first panicle on a plant had emerged 50% from the flag leaf sheath. Panicle number was measured as the total number of panicles on a plant. When available, 5 panicles per plant were photographed (*n*=15 panicles per RIL) using the PANorama imaging protocol described below^21^. Raw phenotypes and trait averages used for genetic mapping are stored in Supplementary Data 14 and 15.

### Panicle imaging protocol

Following the PANorama imaging protocol^21^, 3,443 images were collected and analysed using a pixel to length conversion of 114.5 pixels per cm. PANorama1.0 contained phenotyping capabilities for 18 major traits, which were calculated via image segmentation and subdivision of panicle axes^18^. Additional, nested phenotypes used in this study (that is, subdivision of the panicle axes into upper and lower halves) were calculated from measurements extracted after the image segmentation and skeletonization process, and thus did not require alternation to the algorithms implemented in PANorama1.0. Detailed descriptions of these phenotypes are available in Supplementary Table 2. An updated version of PANorama containing all nested phenotypes used within this study, PANorama2.0, is available for download at sourceforge.net/panorama1.

### Phenotype statistical analyses

Histograms, boxplots, correlations and GWAS analyses were constructed using phenotypic grand means for each variety. *P-*values for Pearson's correlation coefficients were calculated with a two-sided *t*-test using the cor.test() function in R^67^. We provide pseudo-heritability of several phenotypes, described here as ‘AD heritability', using the Methods described in Spindel *et al.*^25^ The restricted maximum likelihood estimate of the genetic variance was calculated using the mixed.solve() function in the R package rrBLUP (version 4.3) and the value was divided by the total phenotypic variance. Broad sense heritability (H) for each phenotype was estimated using repeatability among phenotypic measurements, calculated as the variance among variety grand means divided by the total phenotypic variance of raw trait values. The best linear unbiased predictors (BLUPs) for genetic values were calculated using the mixed.solve() function in the R package rrBLUP (version 4.3). *P-*values for genetic correlation coefficients between BLUPs were calculated with a two-sided *t*-test using the cor.test() function in R^67^.

### GWAS mapping

EMMAX was used to calculate the linear mixed model and significance levels within the GWAS model^31^. For all GWAS runs, within subpopulations or across all accessions, we used the equation:


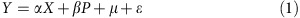


For GWAS runs incorporating HD as a covariate:


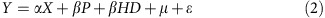


For GWAS runs incorporating a panicle trait as a covariate:


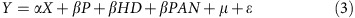


where *Y* and *X* represent the phenotype and SNP genotype vectors, respectively; *P* is a matrix containing the residuals of the first three PCs; *HD* represents a vector of the HD phenotype; and *PAN* represents a vector of the panicle phenotype used within the run (RL, PBN, PBL or NL depending on the run). For genotypic and environmental random effects, respectively, *μ*∼*N*(0, *σ*^2^_g_*K*) and *ɛ*∼*N*(0, *σ*^2^_e_*I*), where *K* is an identity by state kinship matrix accounting for pairwise relatedness between accessions. SNP marker filtering (minor allele frequency=0.1 and genetic missingness=0.3) and identity by state matrix calculations were performed using PLINK^69^. As yield components were collected on a subset of the accessions within our panel (Supplementary Table 1), we performed GWAS for these traits within the *Indica* and *Japonica* subspecies rather than in the individual subpopulations, to maximize our power to detect loci. We noted that certain traits were more susceptible to confounding than others, especially when performing GWAS across subpopulations using the entire panel of accessions. To correct for these issues, we systematically diagnosed the quantile–quantile plots for every trait–subpopulation–covariate combination and used logarithmic transformations on non-normal phenotypes (Supplementary Table 2). The significance threshold was set *at P*<1 × 10^−5^ for every trait and was similar to the false discovery rate^20^.

### QTL mapping

Using R/QTL (version 1.24.9), QTL mapping was performed as described in Crowell *et al.*^21^. Briefly, QTL were identified using Haley–Knott regression and the significance threshold was set using 1,000 permutations. We then scanned for QTL, condition on peaks that had already been detected. Finally, forward selection and backward elimination were used to refine QTL locations. All phenotypic distributions were systematically diagnosed for normality using a Shapiro–Wilkes test and non-normal phenotypes were transformed logarithmically before mapping. For highly non-normal phenotypes that could not be corrected using transformation, a non-parametric QTL model was used. Supplementary Table 5 contains a list of QTL results, including information regarding transformations and QTL model used on a per trait basis. We also provide visual summaries of significant QTL intervals using the track feature in the UCSC Genome Browser (Supplementary Figs 83–93) (www.genome.ucsc.edu).

### Association networks

Significant SNPs were binned together into peaks using a sliding window based on the decay of a LD using the PLINK^67^ command --clump-p1 0.00001 --clump-p2 0.0001 --clump-r2 0.3 --clump-kb 150 --clump-allow-overlap. Thus, for every SNP with *P*<1 × 10^−5^, pairwise *r*^2^-values were calculated between surrounding SNPs that (1) fell within 150 kb and (2) had a *P*<1 × 10^−3^; any two SNPs meeting this criteria that also shared an *r*^2^≥0.3 were clumped into bins. All significant SNPs within the study were used in the construction of bins, regardless of the traits with which they shared associations. In addition, any bins sharing overlapping borders after using the PLINK clump command were collapsed into a single bin. Singleton, significant SNPs (<1 × 10^−5^) were discarded if no other SNP within the LD window was<2.5 × 10^−4^. To construct association networks, traits and their corresponding bins were treated as nodes within the programme Cytoscape^41^ (version 3.1) and edges were labelled by the subpopulation in which the trait association was identified.

### Candidate gene analyses

A list of 319 candidate genes was assembled using a literature review and BLAST searches for candidate gene homologues (Supplementary Table 6). Single gene coexpression networks for the *a priori* candidate genes in Table 2 were constructed in RiceFREND^26^ (http://ricefrend.dna.affrc.go.jp/) using the settings displayed alongside the HyperTree in Supplementary Figs 73–82. Raw RiceFREND data are available in Supplementary Data 9. LD plots and *r*^2^-values for candidate gene zoom-ins were constructed using Haploview^70^ (version 4.2).

## Additional information

**How to cite this article:** Crowell, S. *et al.* Genome-wide association and high-resolution phenotyping link *Oryza sativa* panicle traits to numerous trait-specific QTL clusters. *Nat. Commun.* 7:10527 doi: 10.1038/ncomms10527 (2016).

## Supplementary Material

Supplementary Information Supplementary Figures 1-94, Supplementary Tables 1-6, Supplementary Note and Supplementary References

## Figures and Tables

**Table 1:** Genome-wide association results for panicle traits divided by subpopulation and covariate combinations.

**Subpopulation**	**Mixed model**	**Mixed model+HD Cov.**
All	709 (358)	496 (256)
*aus*	148 (52)	117 (44)
*indica*	48 (38)	54 (39)
*tropical japonica*	132 (41)	49 (24)
Total associations	1,037 (489)	716 (363)

HD, heading date; LD, linkage disequilbrium; SNP, single-nucleotide polymorphism.

The number of significant peaks is in parentheses and is defined by binning significant SNPs using a sliding window of LD.

**Table 2:** Candidate genes identified near significant GWAS peaks.

**Candidate gene**^(ref.)^	**Trait**	**Chr.**	**Gene**	**Biological pathway**	Position*
SD1^42^	Plant height	1	LOC_Os01g66100†	Gibberellin enzyme	38,418,739
EP3/LP^43^	PL	2	LOC_Os02g15950†	F-box transcription factor; cytokinin homeostasis	9,109,565
*OsMADS47*^44^	Panicle branch number	3	LOC_Os03g08754	MADS-Box transcription factor	4,468,547
OsKS1^45^	Panicle NL traits	4	LOC_Os04g52230†	Gibberellin enzyme	31,029,056
CYP90D3^46^	Panicle branch length, panicle NL traits	5	LOC_Os05g11130	Brassinosteroid enzyme	6,264,833
GID1^47^	Booting	5	LOC_Os05g33730†	Soluble gibberellin receptor	19,891,242
OsGA2 oxidase-5^44^	Shoot biomass	7	LOC_Os07g01340‡	Gibberellin enzyme	216,325
OsBZR1^48^	Panicle NL traits	7	LOC_Os07g39220	Transcription factor; brassinosteroid homeostasis	23,477,027
FZP^49^	Secondary panicle branching	7	LOC_Os07g47330	AP2 domain transcription factor	28,297,303
WRKY2^50^	Panicle branch length traits	10	LOC_Os10g42850†	WRKY transcription factor	23,095,323

Chr., chromosome; GWAS, genome-wide association study; NL, internode length; PL, panicle length; QTL, quantitative trait loci; RIL, recombinant inbred line; SNP, single-nucleotide polymorphism.

^*^Closest significant SNP. Position in bp based on MSUv7 assembly. Manhattan plots for trait associations are located within Supplementary Figs 6–64.

^†^Overlap with QTL identified using the IR64 × Azucena RIL population.

^‡^Identified without heading covariate.

## References

[b1] Doust A. Architectural evolution and its implications for domestication in grasses. Ann. Bot. 100, 941–950 (2007). 17478546 10.1093/aob/mcm040 PMC2759198

[b2] Wang Y. H. & Li J. Y. Branching in rice. Curr. Opin. Plant Biol. 14, 94–99 (2011). 21144796 10.1016/j.pbi.2010.11.002

[b3] Ikeda K., Sunohara H. & Nagato Y. Developmental course of inflorescence and spikelet in rice. Breeding Sci. 54, 147–156 (2004).

[b4] Kobayashi S., Fukuta Y., Sato T., Osaki M. & Khush G. S. Molecular marker dissection of rice (*Oryza sativa* L.) plant architecture under temperate and tropical climates. Theor. Appl. Genet. 107, 1350–1356 (2003). 12920520 10.1007/s00122-003-1388-8

[b5] Li Z. K. *et al.* QTL x environment interactions in rice. I. Heading date and plant height. Theor. Appl. Genet. 108, 141–153 (2003). 12961067 10.1007/s00122-003-1401-2

[b6] Yoshida H. & Nagato Y. Flower development in rice. J. Exp. Bot. 62, 4719–4730 (2011). 21914655 10.1093/jxb/err272

[b7] Ohsumi A. *et al.* Evaluation of yield performance in rice near-isogenic lines with increased spikelet number. Field Crop Res. 120, 68–75 (2011).

[b8] Brown P. J. *et al.* Distinct genetic architectures for male and female inflorescence traits of maize. PLoS Genet. 7, e1002383 (2011). 22125498 10.1371/journal.pgen.1002383 PMC3219606

[b9] Yamaki S. *et al.* Diversity of panicle branching patterns in wild relatives of rice. Breeding Sci. 60, 586–596 (2010).

[b10] Zhang D. B. & Yuan Z. Molecular control of grass inflorescence development. Annu. Rev. Plant Biol. 65, 553 (2014). 24471834 10.1146/annurev-arplant-050213-040104

[b11] Ishii T. *et al.* OsLG1 regulates a closed panicle trait in domesticated rice. Nat. Genet. 45, 462–465 (2013). 23435087 10.1038/ng.2567

[b12] Huang X. Z. *et al.* Natural variation at the DEP1 locus enhances grain yield in rice. Nat. Genet. 41, 494–497 (2009). 19305410 10.1038/ng.352

[b13] Ashikari M. *et al.* Cytokinin oxidase regulates rice grain production. Science 309, 741–745 (2005). 15976269 10.1126/science.1113373

[b14] Garris A. J., Tai T. H., Coburn J., Kresovich S. & McCouch S. Genetic structure and diversity in *Oryza sativa* L. Genetics 169, 1631–1638 (2005). 15654106 10.1534/genetics.104.035642 PMC1449546

[b15] Zhao K. Y. *et al.* Genomic diversity and introgression in *O. sativa* reveal the impact of domestication and breeding on the rice genome. PLoS ONE 5, e10780 (2010). 20520727 10.1371/journal.pone.0010780 PMC2875394

[b16] Huang X. H. *et al.* Genome-wide association study of flowering time and grain yield traits in a worldwide collection of rice germplasm. Nat. Genet. 44, 32–39 (2012). 22138690 10.1038/ng.1018

[b17] Huang X. H. *et al.* Genome-wide association studies of 14 agronomic traits in rice landraces. Nat. Genet, 42, 961–967 (2010). 20972439 10.1038/ng.695

[b18] Famoso A. N. *et al.* Genetic architecture of aluminum tolerance in rice (*Oryza sativa*) determined through genome-wide association analysis and QTL mapping. PLoS Genet. 7, e1002221 (2011). 21829395 10.1371/journal.pgen.1002221 PMC3150440

[b19] Zhao K. *et al.* Genome-wide association mapping reveals a rich genetic architecture of complex traits in *Oryza sativa*. Nat. Commun. 2, 467 (2011). 21915109 10.1038/ncomms1467 PMC3195253

[b20] McCouch S. *et al.* Open access resources for genome wide association mapping in rice. *Nat. Commun*. 7, 10532 (2016). 10.1038/ncomms10532 PMC4742900 26842267

[b21] Crowell S. *et al.* High-resolution inflorescence phenotyping using a novel image-analysis pipeline, PANorama. Plant Physiol. 165, 479–495 (2014). 24696519 10.1104/pp.114.238626 PMC4044845

[b22] Raj A., Stephens M. & Pritchard J. K. fastSTRUCTURE: variational inference of population structure in large SNP data sets. Genetics 197, 573–589 (2014). 24700103 10.1534/genetics.114.164350 PMC4063916

[b23] Huang X. H. *et al.* A map of rice genome variation reveals the origin of cultivated rice. Nature 490, 497 (2012). 23034647 10.1038/nature11532 PMC7518720

[b24] Luo A. D. *et al.* EUI1, encoding a putative cytochrome P450 monooxygenase, regulates internode elongation by modulating gibberellin responses in rice. Plant Cell Physiol. 47, 181–191 (2006). 16306061 10.1093/pcp/pci233

[b25] Spindel J. *et al.* Genomic selection and association mapping in rice (*Oryza sativa*): effect of trait genetic architecture, training population composition, marker number and statistical model on accuracy of rice genomic selection in elite, tropical rice breeding lines. PLoS Genet. 11, e1004982 (2015). 25689273 10.1371/journal.pgen.1004982 PMC4334555

[b26] Sato Y. *et al.* RiceFREND: a platform for retrieving coexpressed gene networks in rice. Nucleic Acids Res. 41, D1214–D1221 (2013). 23180784 10.1093/nar/gks1122 PMC3531108

[b27] Zhang Z. X. *et al.* A proteomic study on molecular mechanism of poor grain-filling of rice (*Oryza sativa* L.) inferior spikelets. PLoS ONE 9, e89140 (2014). 24586550 10.1371/journal.pone.0089140 PMC3931721

[b28] Fujita D. *et al.* NAL1 allele from a rice landrace greatly increases yield in modern indica cultivars. Proc. Natl Acad. Sci. USA 110, 20431–20436 (2013). 24297875 10.1073/pnas.1310790110 PMC3870739

[b29] Hedden P. The genes of the green revolution. Trends Genet. 19, 5–9 (2003). 12493241 10.1016/s0168-9525(02)00009-4

[b30] Atwell S. *et al.* Genome-wide association study of 107 phenotypes in *Arabidopsis thaliana* inbred lines. Nature 465, 627–631 (2010). 20336072 10.1038/nature08800 PMC3023908

[b31] Kang H. M. *et al.* Variance component model to account for sample structure in genome-wide association studies. Nat. Genet. 42, 348–354 (2010). 20208533 10.1038/ng.548 PMC3092069

[b32] Mei H. W. *et al.* Gene actions of QTLs affecting several agronomic traits resolved in a recombinant inbred rice population and two backcross populations. Theor. Appl. Genet. 110, 649–659 (2005). 15647921 10.1007/s00122-004-1890-7

[b33] Mei H. W. *et al.* Gene actions of QTLs affecting several agronomic traits resolved in a recombinant inbred rice population and two testcross populations. Theor. Appl. Genet. 107, 89–101 (2003). 12721635 10.1007/s00122-003-1192-5

[b34] Yamamoto T., Taguchi-Shiobara F., Ukai Y., Sasaki T. & Yano M. Mapping quantitative trait loci for days-to-heading, and culm, panicle and internode lengths in a BC1F3 population using an elite rice variety, Koshihikari, as the recurrent parent. Breeding Sci. 51, 63–71 (2001).

[b35] He P. *et al.* Comparison of molecular linkage maps and agronomic trait loci between DH and RIL populations derived from the same rice cross. Crop Sci. 41, 1240–1246 (2001).

[b36] Xiao J., Li J., Yuan L. & Tanksley S. D. Identification of QTLs affecting traits of agronomic importance in a recombinant inbred population derived from a subspecific rice cross. Theor. Appl. Genet. 92, 230–244 (1996). 24166172 10.1007/BF00223380

[b37] Xiao J. H. *et al.* Identification of trait-improving quantitative trait loci alleles from a wild rice relative, *Oryza rufipogon*. Genetics 150, 899–909 (1998). 9755218 10.1093/genetics/150.2.899 PMC1460369

[b38] Matsubara K. *et al.* Ehd2, a rice ortholog of the maize INDETERMINATE1 gene, promotes flowering by up-regulating Ehd1. Plant Physiol. 148, 1425–1435 (2008). 18790997 10.1104/pp.108.125542 PMC2577255

[b39] Xue W. Y. *et al.* Natural variation in Ghd7 is an important regulator of heading date and yield potential in rice. Nat. Genet. 40, 761–767 (2008). 18454147 10.1038/ng.143

[b40] Yan W. H. *et al.* A major QTL, Ghd8, plays pleiotropic roles in regulating grain productivity, plant height, and heading date in rice. Mol. Plant 4, 319–330 (2011). 21148627 10.1093/mp/ssq070

[b41] Shannon P. *et al.* Cytoscape: a software environment for integrated models of biomolecular interaction networks. Genome Res. 13, 2498–2504 (2003). 14597658 10.1101/gr.1239303 PMC403769

[b42] Ashikari M. *et al.* Loss-of-function of a rice gibberellin biosynthetic gene, GA20 oxidase (GA20ox-2), led to the rice ‘green revolution'. Breeding Sci. 52, 143–150 (2002).

[b43] Piao R. *et al.* Map-based cloning of the ERECT PANICLE 3 gene in rice. Theor. Appl. Genet. 119, 1497–1506 (2009). 19756471 10.1007/s00122-009-1151-x

[b44] Arora R. *et al.* MADS-box gene family in rice: genome-wide identification, organization and expression profiling during reproductive development and stress. BMC Genomics 8, 242 (2007). 17640358 10.1186/1471-2164-8-242 PMC1947970

[b45] Sakamoto T. *et al.* An overview of gibberellin metabolism enzyme genes and their related mutants in rice. Plant Physiol. 134, 1642–1653 (2004). 15075394 10.1104/pp.103.033696 PMC419838

[b46] Sakamoto T., Ohnishi T., Fujioka S., Watanabe B. & Mizutani M. Rice CYP90D2 and CYP90D3 catalyze C-23 hydroxylation of brassinosteroids *in vitro*. Plant Physiol. Biochem. 58, 220–226 (2012). 22846333 10.1016/j.plaphy.2012.07.011

[b47] Ueguchi-Tanaka M. *et al.* Molecular interactions of a soluble gibberellin receptor, GID1, with a rice DELLA protein, SLR1, and gibberellin. Plant Cell 19, 2140–2155 (2007). 17644730 10.1105/tpc.106.043729 PMC1955699

[b48] Bai M. Y. *et al.* Functions of OsBZR1 and 14-3-3 proteins in brassinosteroid signaling in rice. Proc. Natl Acad. Sci. USA 104, 13839–13844 (2007). 17699623 10.1073/pnas.0706386104 PMC1959469

[b49] Komatsu M., Chujo A., Nagato Y., Shimamoto K. & Kyozuka J. FRIZZY PANICLE is required to prevent the formation of axillary meristems and to establish floral meristem identity in rice spikelets. Development 130, 3841–3850 (2003). 12835399 10.1242/dev.00564

[b50] Ross C. A., Liu Y. & Shen Q. X. J. The WRKY gene family in rice (*Oryza sativa*). J. Integr. Plant Biol. 49, 827–842 (2007).

[b51] Takai T. *et al.* A natural variant of NAL1, selected in high-yield rice breeding programs, pleiotropically increases photosynthesis rate. Sci. Rep. 3, 2149 (2013). 23985993 10.1038/srep02149 PMC3756344

[b52] Zhang G. H. *et al.* LSCHL4 from *Japonica* cultivar, which is allelic to NAL1, increases yield of *Indica* super rice 93-11. Mol. Plant 7, 1350–1364 (2014). 24795339 10.1093/mp/ssu055 PMC4115278

[b53] Jiang D. *et al.* Characterization of a null allelic mutant of the rice NAL1 gene reveals its role in regulating cell division. PLoS ONE 10, e0118169 (2015). 25658704 10.1371/journal.pone.0118169 PMC4320051

[b54] Taguchi-Shiobara F. *et al.* Natural variation in the flag leaf morphology of rice due to a mutation of the NARROW LEAF 1 gene in *Oryza sativa* L. Genetics 201, 795–808 (2015). 26275424 10.1534/genetics.115.181040 PMC4596685

[b55] Dixit S. *et al.* Action of multiple intra-QTL genes concerted around a co-localized transcription factor underpins a large effect QTL. Sci. Rep. 5, 15183 (2015). 26507552 10.1038/srep15183 PMC4623671

[b56] Brachi B., Morris G. P. & Borevitz J. O. Genome-wide association studies in plants: the missing heritability is in the field. Genome Biol. 12, 232 (2011). 22035733 10.1186/gb-2011-12-10-232 PMC3333769

[b57] Horton M. W. *et al.* Genome-wide patterns of genetic variation in worldwide *Arabidopsis thaliana* accessions from the RegMap panel. Nat. Genet. 44, 212–216 (2012). 22231484 10.1038/ng.1042 PMC3267885

[b58] Furutani I., Sukegawa S. & Kyozuka J. Genome-wide analysis of spatial and temporal gene expression in rice panicle development. Plant J. 46, 503–511 (2006). 16623909 10.1111/j.1365-313X.2006.02703.x

[b59] Sato Y. *et al.* Field transcriptome revealed critical developmental and physiological transitions involved in the expression of growth potential in *japonica* rice. BMC Plant Biol. 11, 10 (2011). 21226959 10.1186/1471-2229-11-10 PMC3031230

[b60] Eveland A. L. *et al.* Regulatory modules controlling maize inflorescence architecture. Genome Res. 24, 431–443 (2014). 24307553 10.1101/gr.166397.113 PMC3941108

[b61] Takahashi Y., Teshima K. M., Yokoi S., Innan H. & Shimamoto K. Variations in Hd1 proteins, Hd3a promoters, and Ehd1 expression levels contribute to diversity of flowering time in cultivated rice. Proc. Natl Acad. Sci. USA 106, 4555–4560 (2009). 19246394 10.1073/pnas.0812092106 PMC2647979

[b62] Khush G. S. Breaking the yield frontier of rice. GeoJournal 35, 329–332 (1995).

[b63] Peng S., Cassman K. G., Virmani S. S., Sheehy J. & Khush G. S. Yield potential trends of tropical rice since the release of IR8 and the challenge of increasing rice yield potential. Crop Sci. 39, 1552–1559 (1999).

[b64] Peng S. B., Khush G. S., Virk P., Tang Q. Y. & Zou Y. B. Progress in ideotype breeding to increase rice yield potential. Field Crop Res. 108, 32–38 (2008).

[b65] Cookson W., Liang L., Abecasis G., Moffatt M. & Lathrop M. Mapping complex disease traits with global gene expression. Nat. Rev. Genet. 10, 184–194 (2009). 19223927 10.1038/nrg2537 PMC4550035

[b66] Spindel J. *et al.* Bridging the genotyping gap: using genotyping by sequencing (GBS) to add high-density SNP markers and new value to traditional bi-parental mapping and breeding populations. Theor. Appl. Genet. 126, 2699–2716 (2013). 23918062 10.1007/s00122-013-2166-x

[b67] R Development Core Team. R: A Language and Environment for Statistical Computing R Foundation for Statistical Computing (2012).

[b68] Rosenberg N. A. DISTRUCT: a program for the graphical display of population structure. Mol. Ecol. Notes 4, 137–138 (2004).

[b69] Purcell S. *et al.* PLINK: A tool set for whole-genome association and population-based linkage analyses. Am. J. Hum. Genet. 81, 559–575 (2007). 17701901 10.1086/519795 PMC1950838

[b70] Barrett J. C., Fry B., Maller J. & Daly M. J. Haploview: analysis and visualization of LD and haplotype maps. Bioinformatics 21, 263–265 (2005). 15297300 10.1093/bioinformatics/bth457

