# Supplementary material for: Genome-wide association and high-resolution phenotyping link Oryza sativa panicle traits to numerous trait-specific QTL clusters
Source: Nat Commun. 2016 Feb 4;7:10527. doi: 10.1038/ncomms10527 (PMC4742901; doi:10.1038/ncomms10527)
Supplement: Supplementary Information — Supplementary Figures 1-94, Supplementary Tables 1-6, Supplementary Note and Supplementary References [file ncomms10527-s1.pdf]

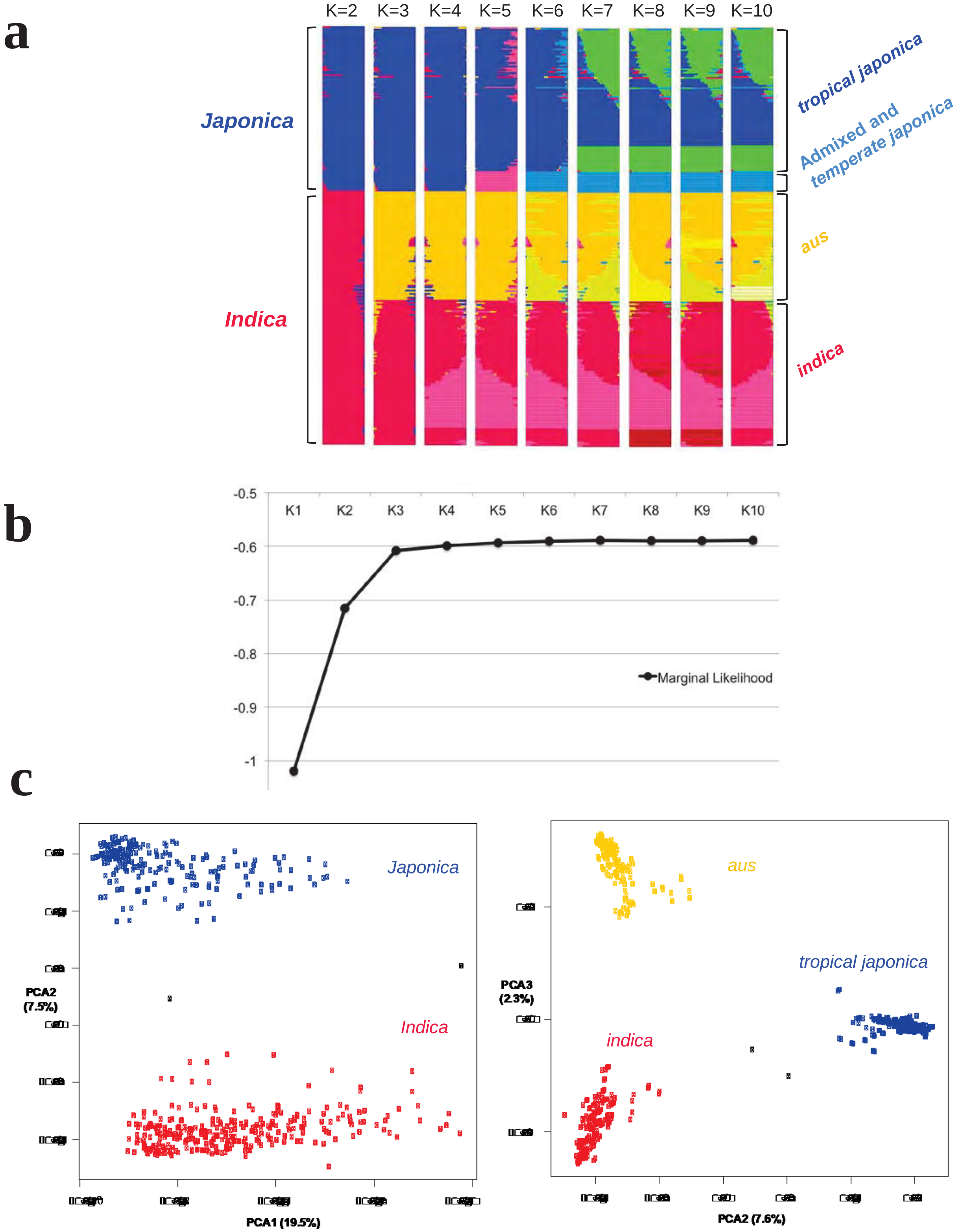

Supplementary Figure 1. Genetic architecture of the diversity panel. (a, b) Genetic structure of the panel analyzed using the program *fastStructure*. Although a K=8 maximizes the marginal likelihood within the panel (a), the marginal likelihood begins to plateau at a K=3 (c) Principle components analysis reveals that the first 3 principle components explain ~30% of the genetic variance within the panel.

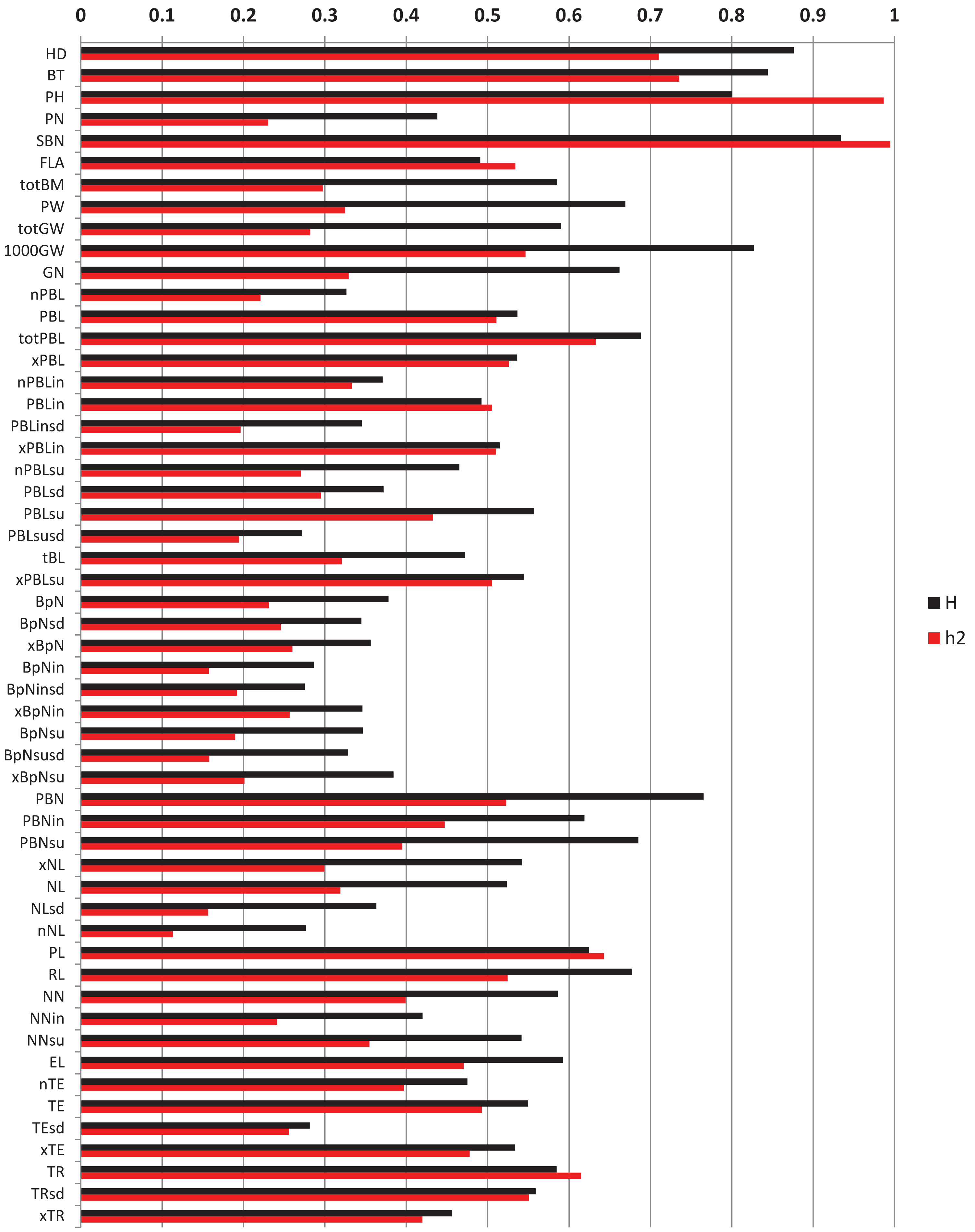

Supplementary Figure 2. Heritability of traits. Broad (H) and narrow sense (h^2) heritability of traits are estimated using repeatability among raw measures and additive+dominance (AD) heritability, respectively. Trait abbreviations correspond to Supplementary Table 2.

Supplementary Figure 3-4. Trait-by-trait relationships. The lower and upper triangles were calculated using varieties in the *Indica* and *Japonica* varietal groups, respectively. Trait abbreviations correspond to phenotypes listed in Supplementary Table 2. (** $P < 0.001$, *** $P < 0.0001$). Supplementary Figure 3 corresponds to phenotype-by-by correlations. Supplementary Figure 4 contains genetic correlations, calculated as correlations between genetic best linear unbiased predictors (GBLUPS).

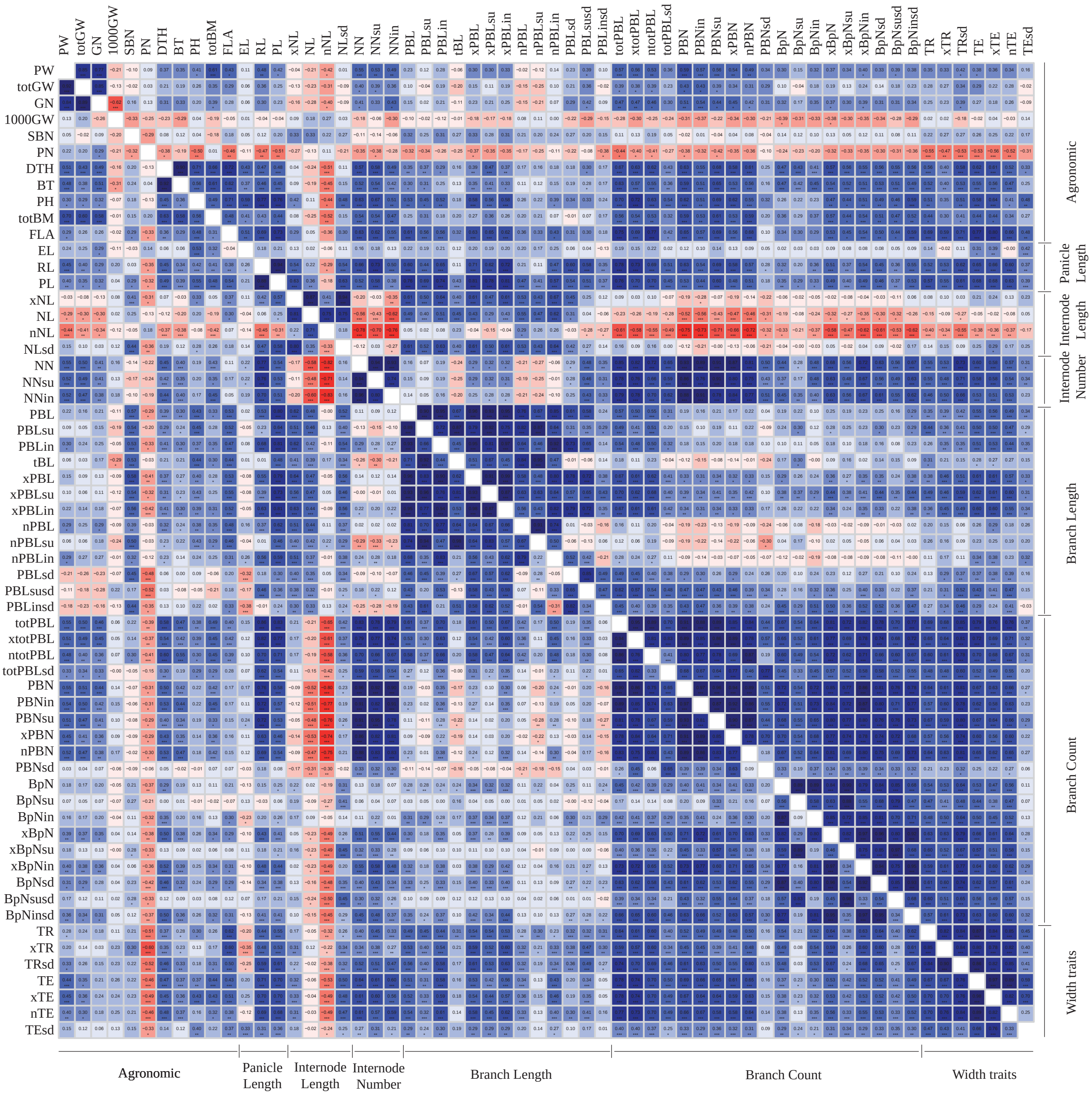

Supplementary Figure 3. Pairwise phenotype correlations.

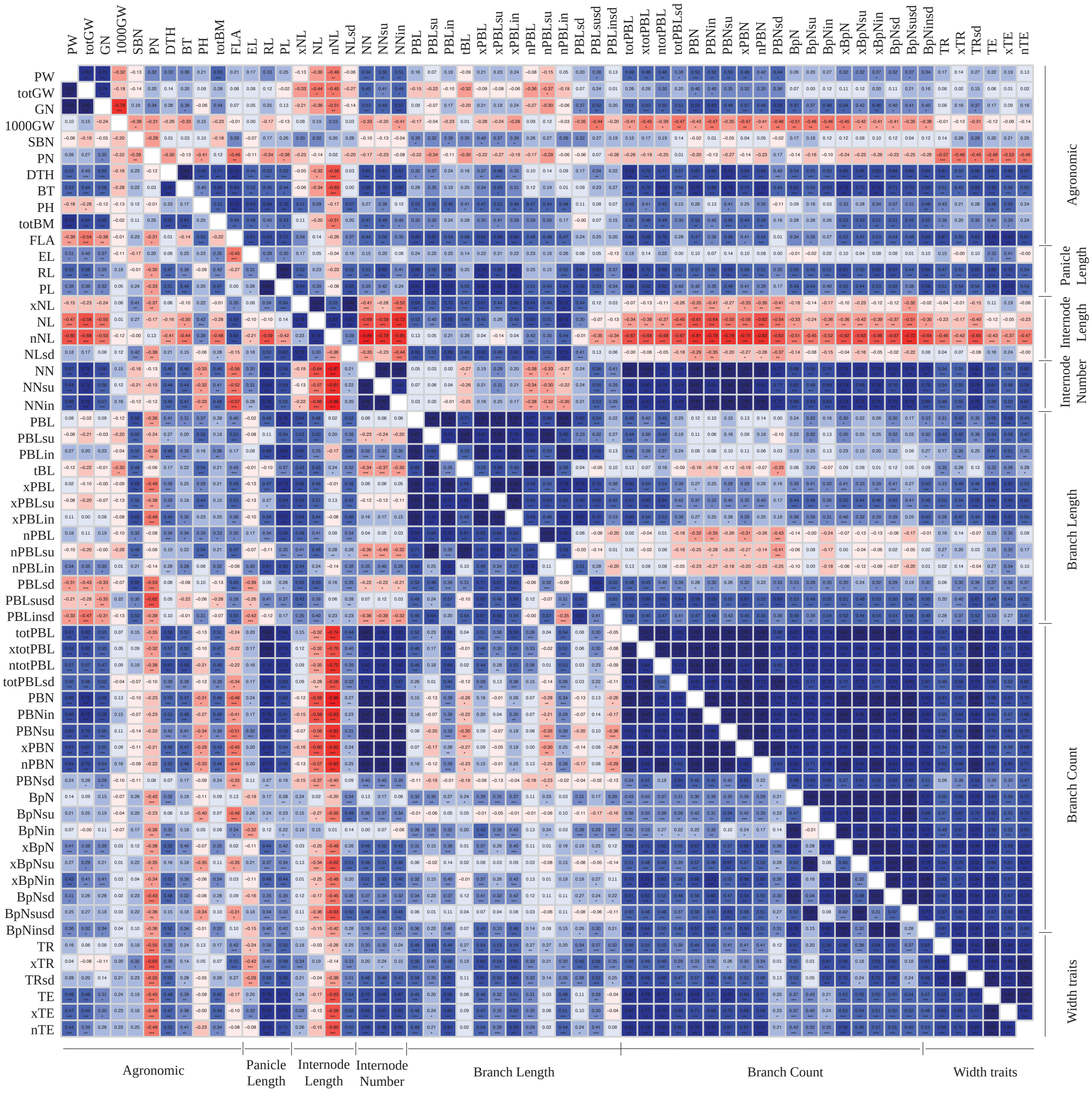

Supplementary Figure 4. Pairwise genetic correlations between traits.

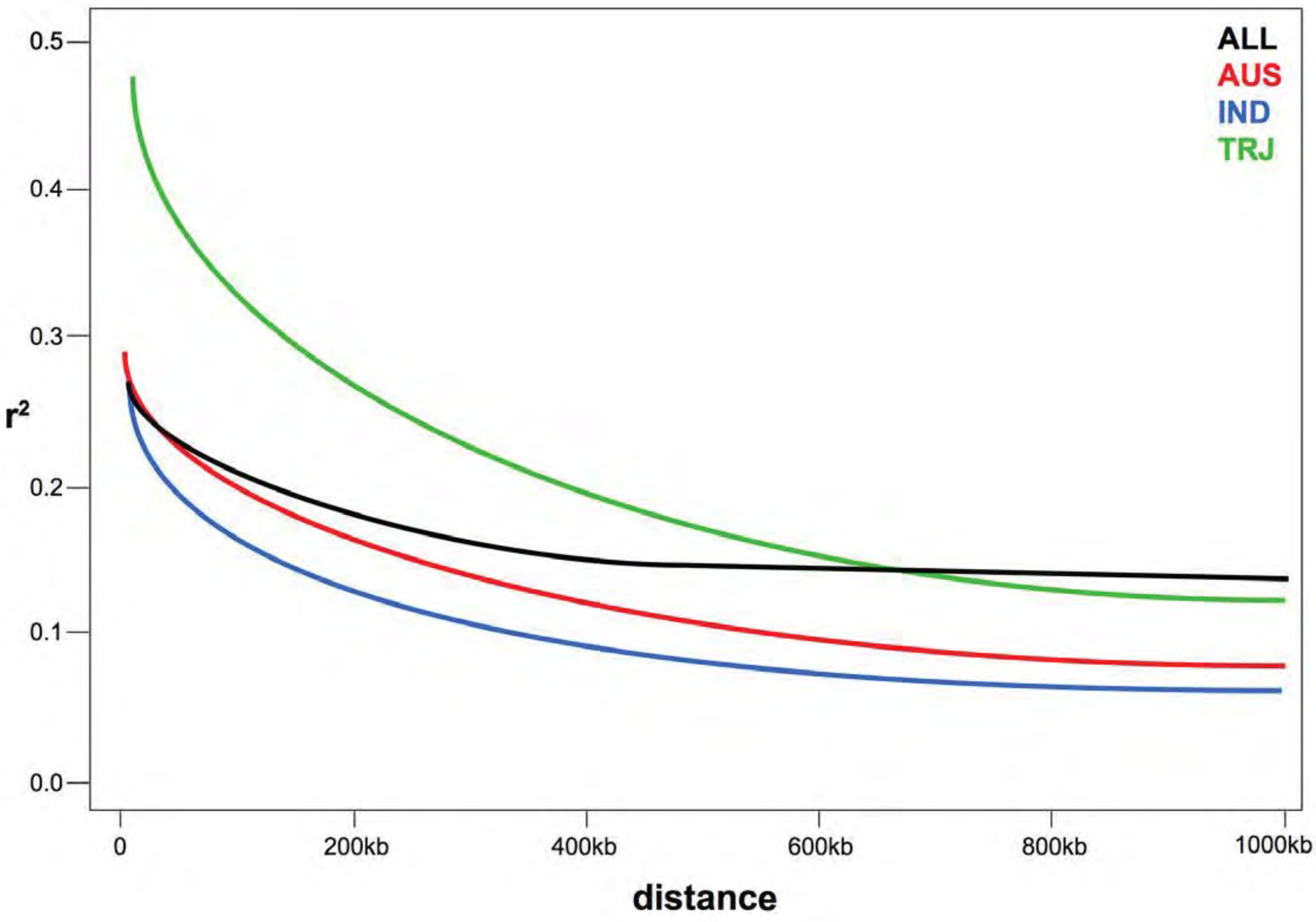

Supplementary Figure 5. LD decay in subpopulations. Average genome-wide linkage disequilibrium (LD) decay as a function of distance, measured as r^2, using pairwise comparisons of SNPs.

Supplementary Figures 6-65. Genome-wide association (GWAS) results separated by trait. (a) A histogram depicting the trait averages of every variety within the panel. (b) A boxplot depicting the mean and range of each phenotype, divided by subpopulation: *aus* (AUS), *indica* (IND), and *tropical japonica* (TRJ). The admixed and *temperate japonica* lines were grouped together in the same boxplot (Admix_or_TEJ). (c) Manhanntan plots and quantile-quantile (QQ) plots summarizing GWAS SNP association results for every trait and subpopulation combination. ALL indicates that every variety in the panel was used when performing associations. For agronomic phenotypes, varietal groups were used instead of subpopulations in order to increase statistical power (*Indica*: *aus* and *indica* lines; *Japonica*: *tropical japonica*, *temperate japonica*, and admixed *japonica* lines). SNPs in red indicate that an *a priori* candidate gene fell within ~30kb of the significant associations. (d) Manhattan plots and QQ plots summarizing GWAS SNP associations for every trait and subpopulation combination, using the heading date covariate within the mixed model.

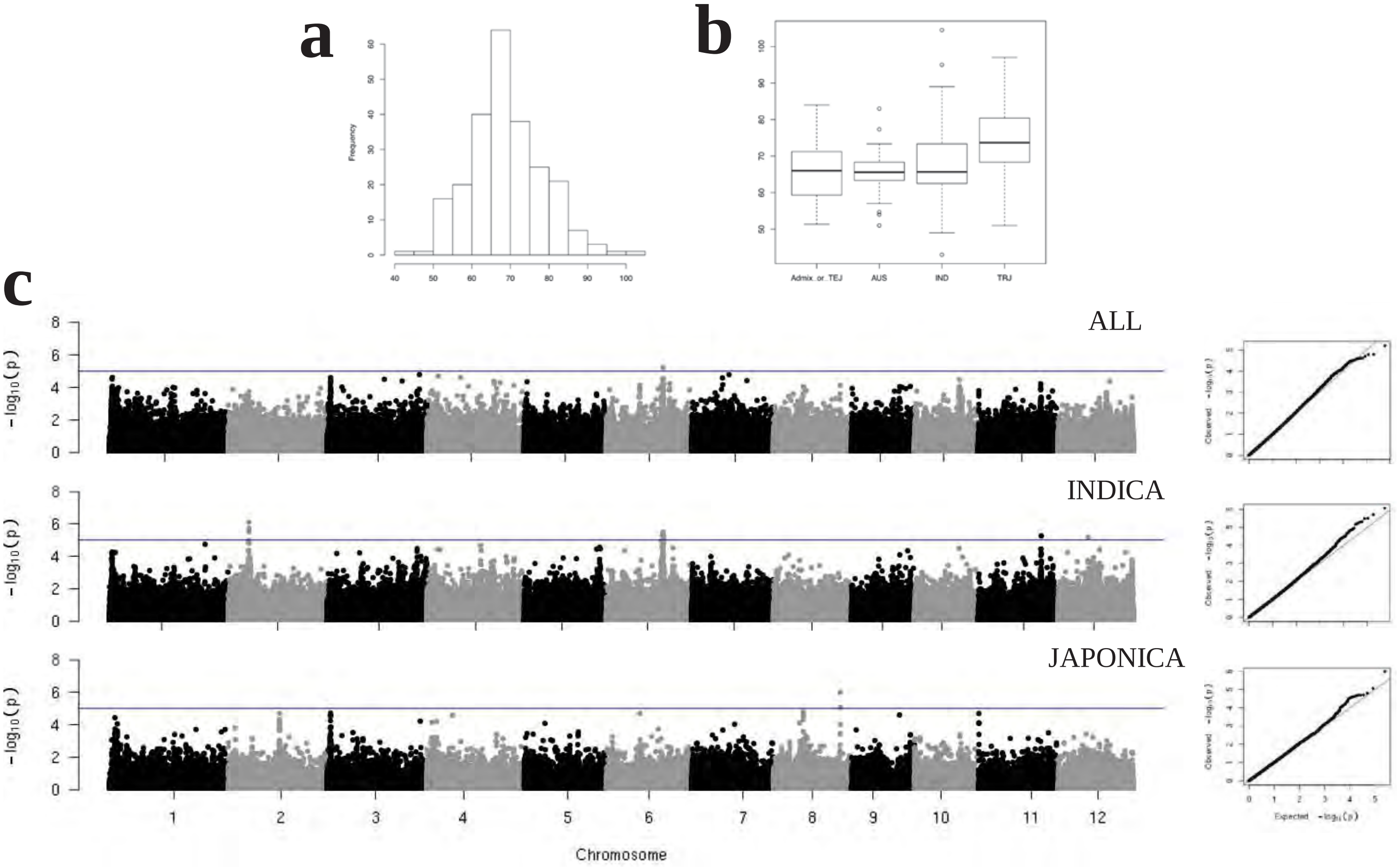

Supplementary Figure 6. Summary of GWAS results for Heading Date (days after transplanting). (**a, b**) Phenotypic distributions. (**c**) Mixed model controlling for subpopulation structure across all varieties (ALL) and within the two major varietal groups *Indica* (INDICA) and *Japonica* (JAPONICA).

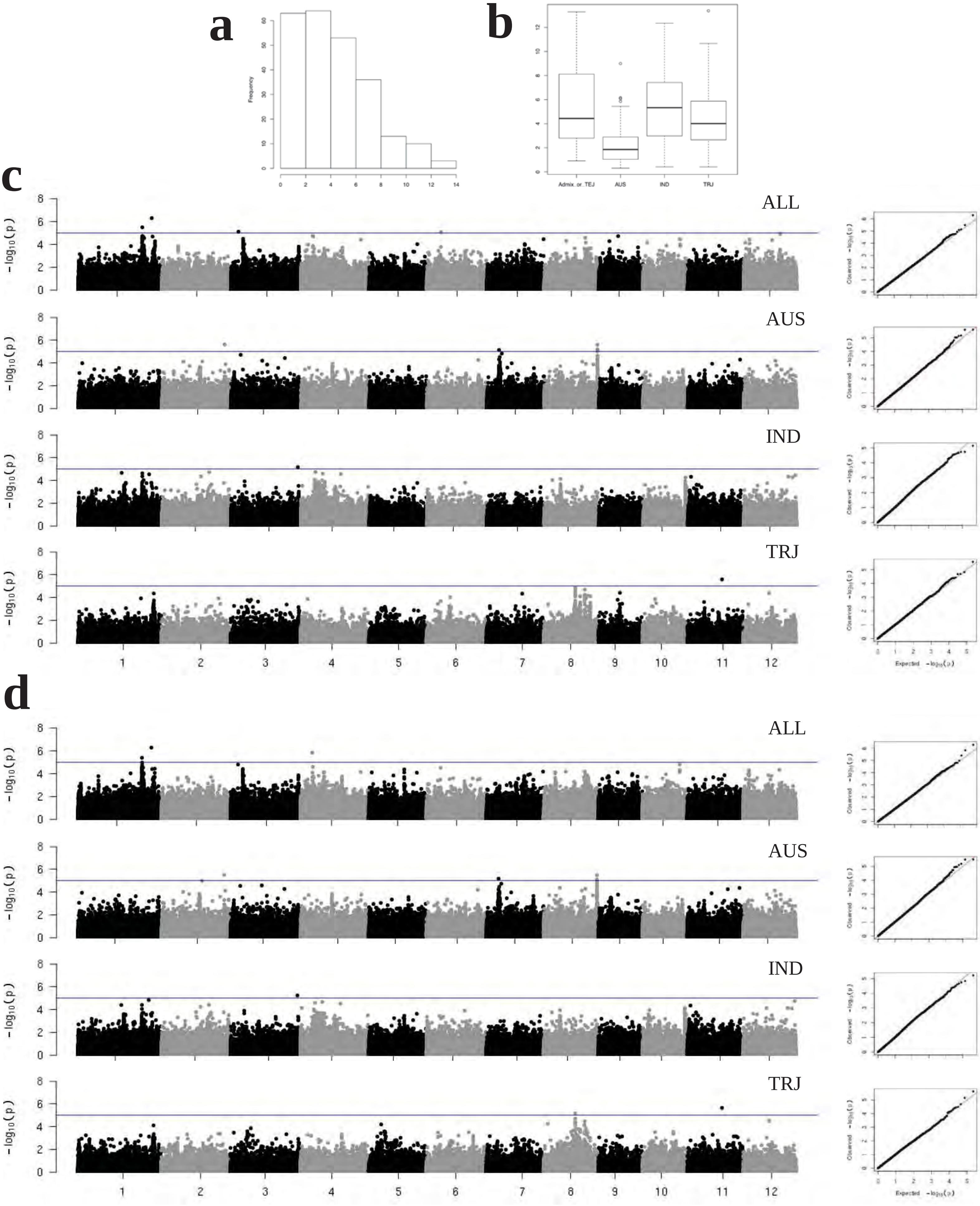

Supplementary Figure 7. Summary of GWAS results for Exsertion Length (cm). (a, b) Phenotypic distributions. (c) Mixed model controlling for subpopulation structure across all varieties (ALL) and within individual subpopulations (AUS = *aus*, IND = *indica*, TRJ = *tropical japonica*). (d) Mixed model with heading date as a covariate.

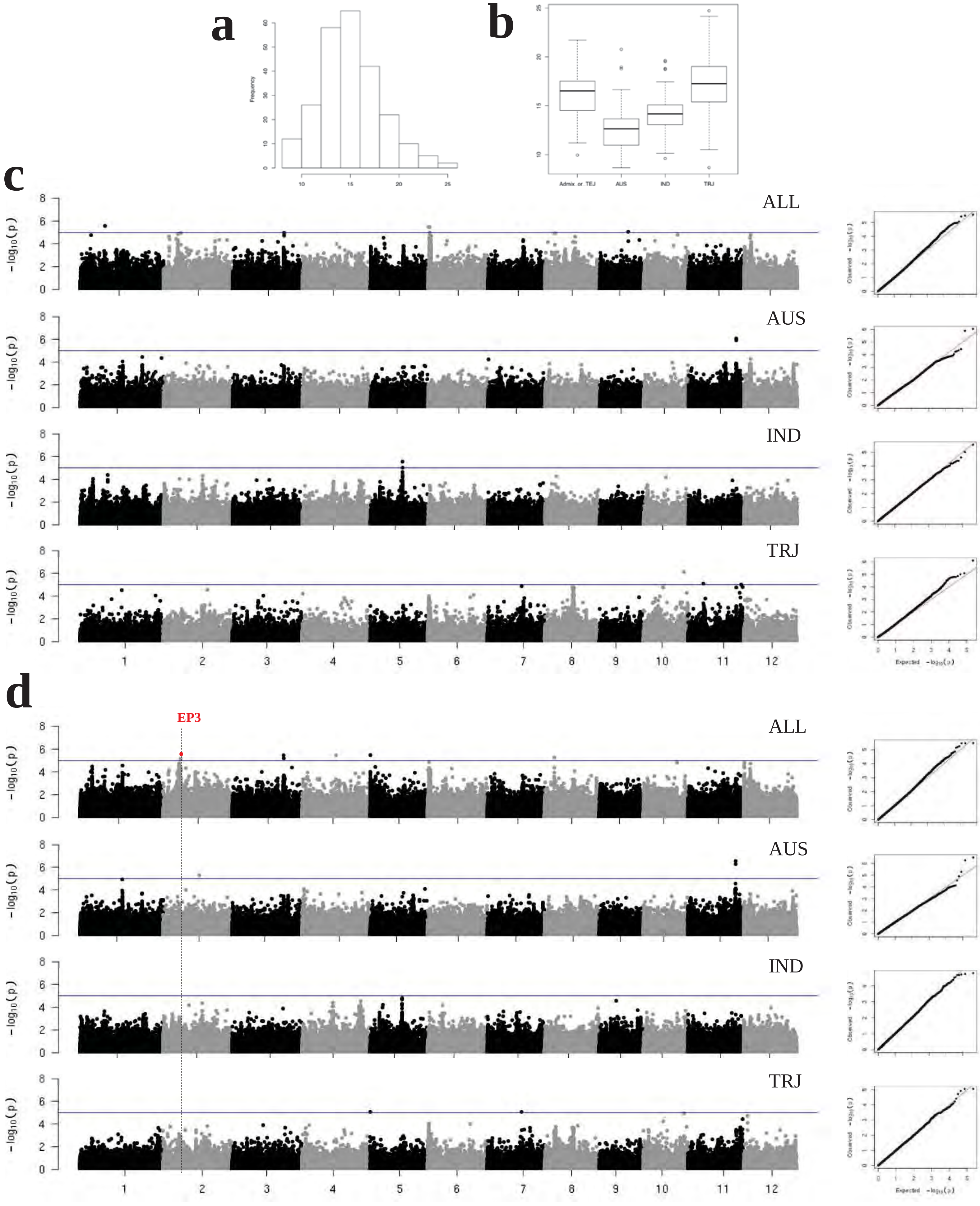

Supplementary Figure 8. Summary of GWAS results for Rachis Length (cm). (**a, b**) Phenotypic distributions. (**c**) Mixed model controlling for subpopulation structure across all varieties (ALL) and within individual subpopulations (AUS = *aus*, IND = *indica*, TRJ = *tropical japonica*). (**d**) Mixed model with heading date as a covariate.

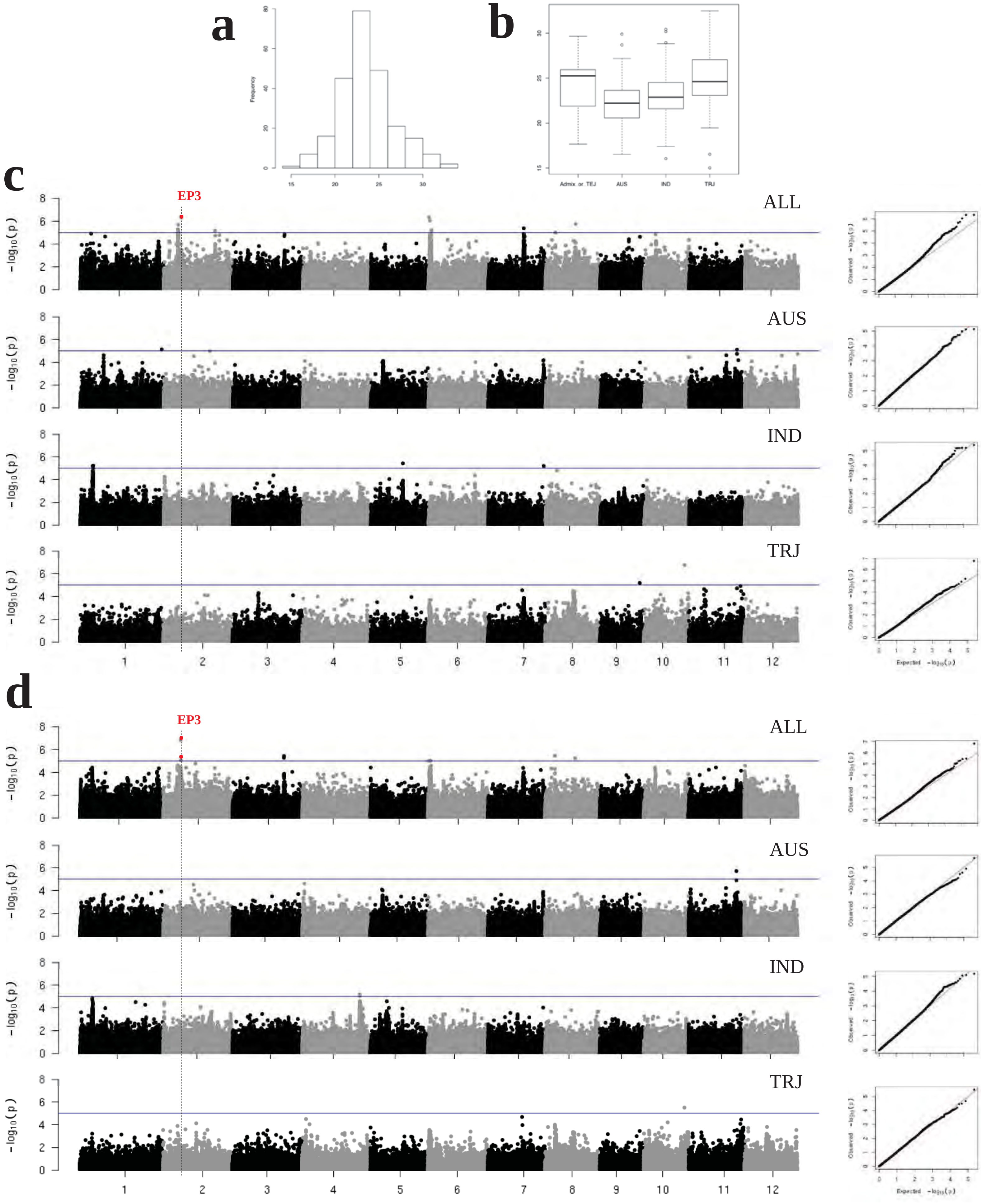

Supplementary Figure 9. Summary of GWAS results for Panicle Length (cm). (**a, b**) Phenotypic distributions. (**c**) Mixed model controlling for subpopulation structure across all varieties (ALL) and within individual subpopulations (AUS = *aus*, IND = *indica*, TRJ = *tropical japonica*). (**d**) Mixed model with heading date as a covariate.

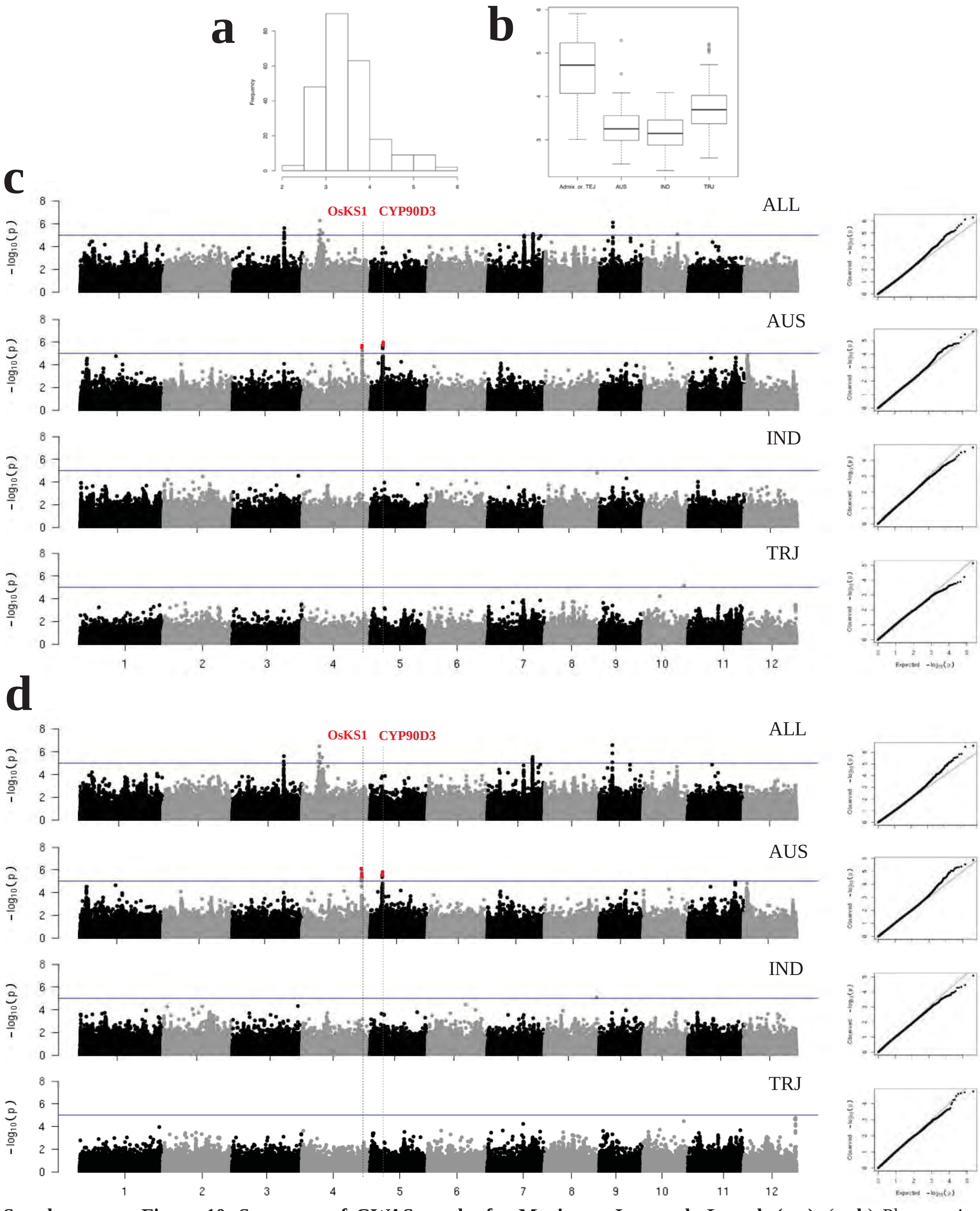

Supplementary Figure 10. Summary of GWAS results for Maximum Internode Length (cm). (**a, b**) Phenotypic distributions. (**c**) Mixed model controlling for subpopulation structure across all varieties (ALL) and within individual subpopulations (AUS = *aus*, IND = *indica*, TRJ = *tropical japonica*). (**d**) Mixed model with heading date as a covariate.

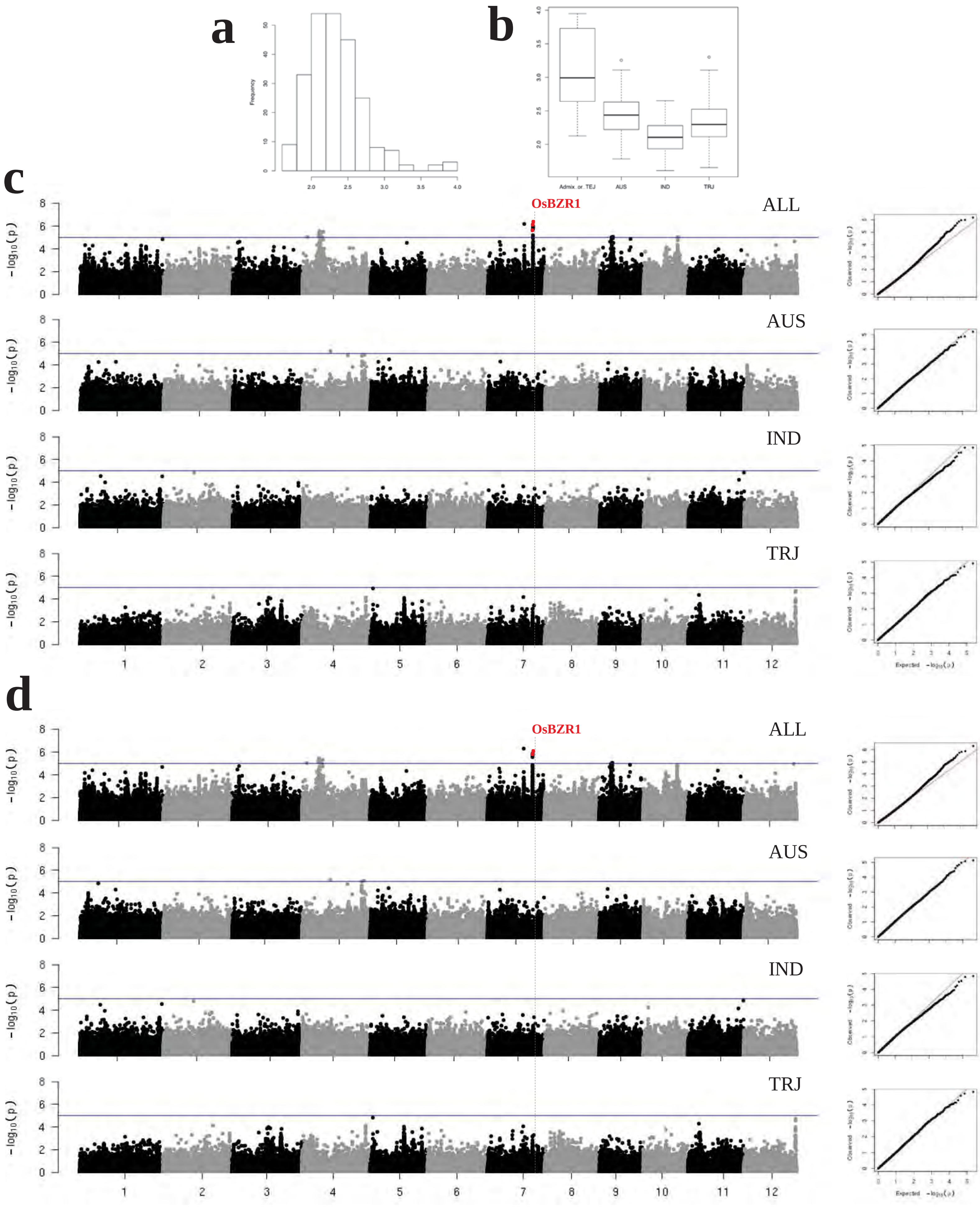

Supplementary Figure 11. Summary of GWAS results for Average Internode Length (cm). (**a, b**) Phenotypic distributions. (**c**) Mixed model controlling for subpopulation structure across all varieties (ALL) and within individual subpopulations (AUS = *aus*, IND = *indica*, TRJ = *tropical japonica*). (**d**) Mixed model with heading date as a covariate.

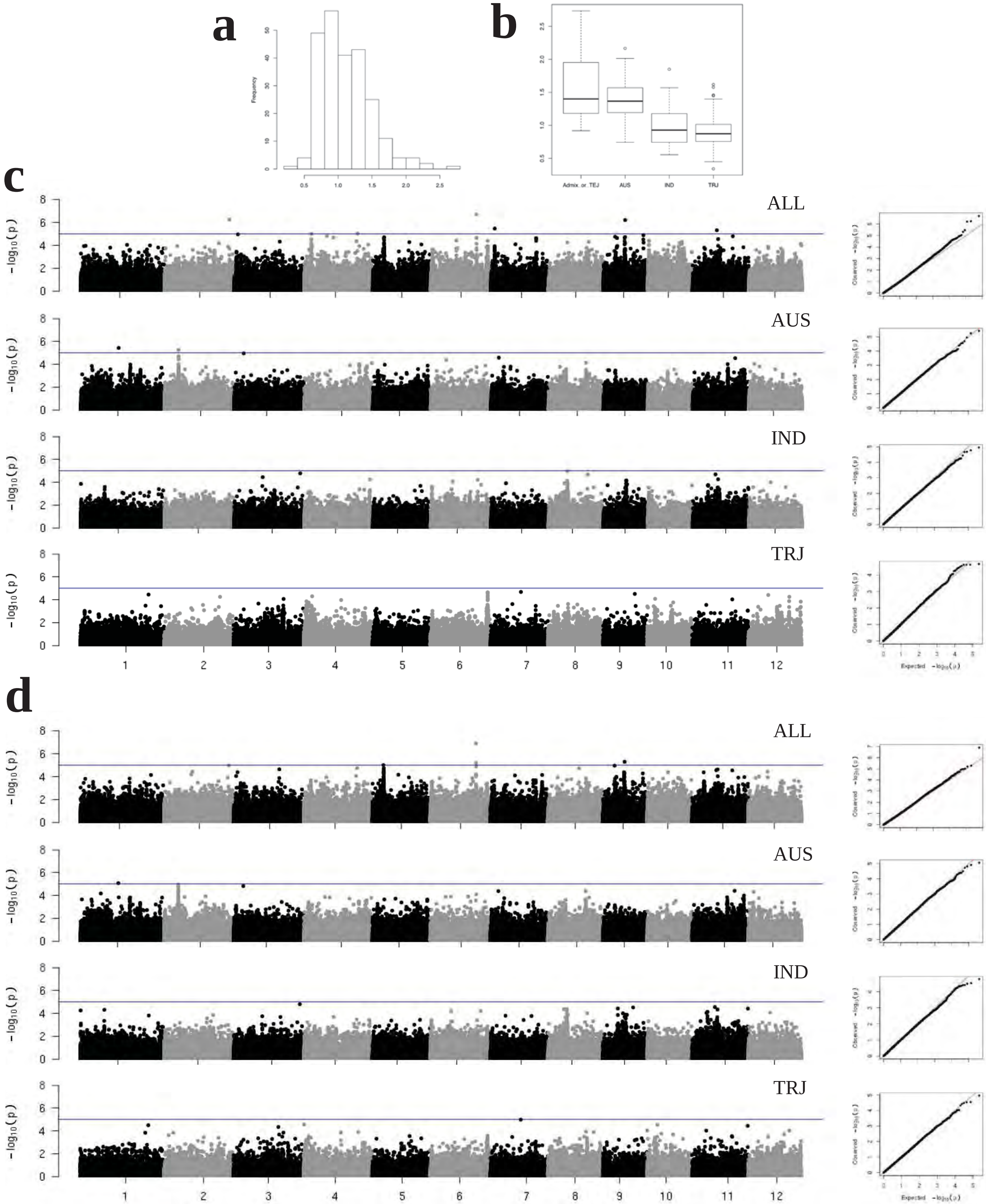

Supplementary Figure 12. Summary of GWAS results for Minimum Internode Length (cm). (**a, b**) Phenotypic distributions. (**c**) Mixed model controlling for subpopulation structure across all varieties (ALL) and within individual subpopulations (AUS = *aus*, IND = *indica*, TRJ = *tropical japonica*). (**d**) Mixed model with heading date as a covariate.

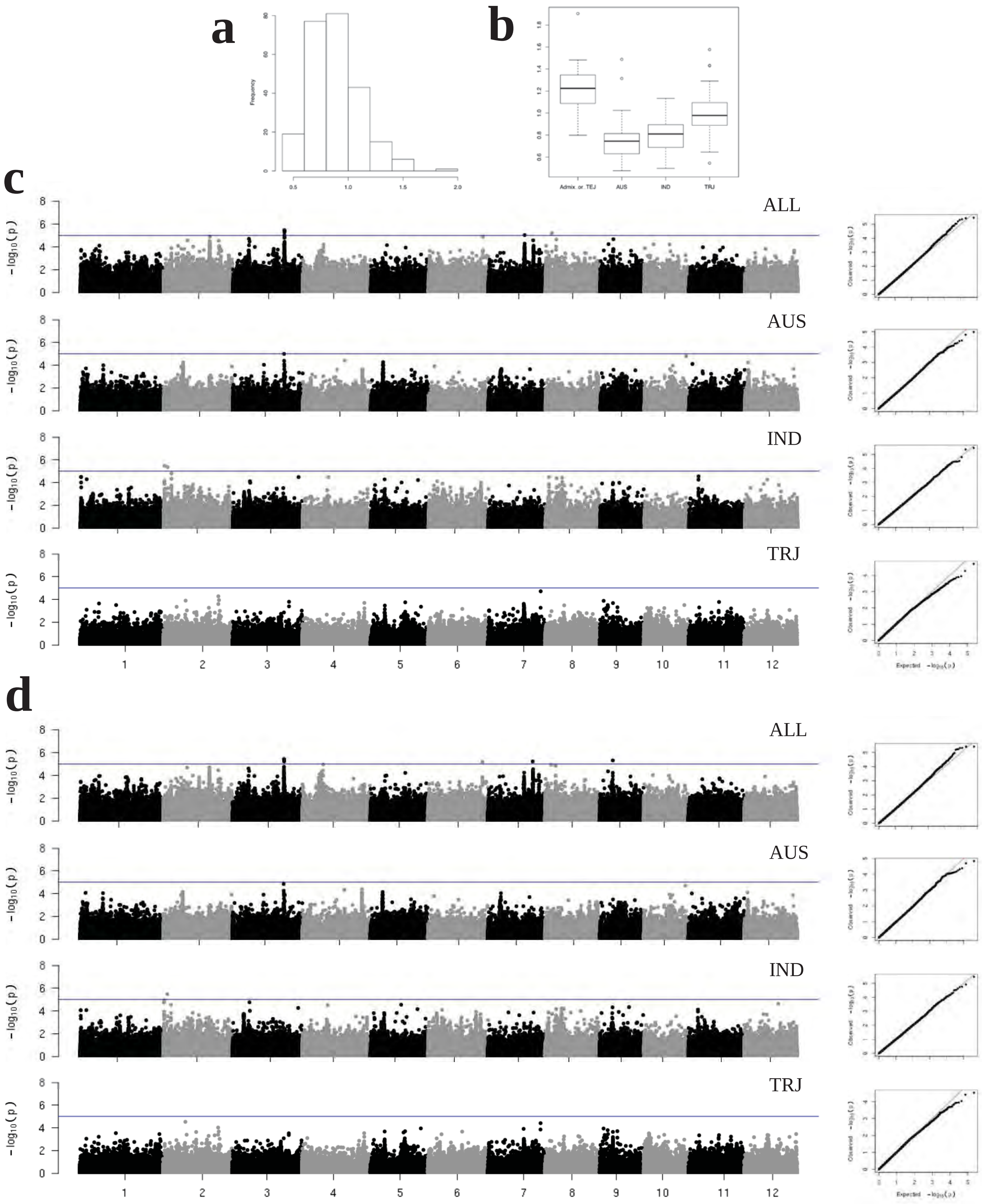

Supplementary Figure 13. Summary of GWAS results for Internode Length Standard Deviation. (**a, b**) Phenotypic distributions. (**c**) Mixed model controlling for subpopulation structure across all varieties (ALL) and within individual subpopulations (AUS = *aus*, IND = *indica*, TRJ = *tropical japonica*). (**d**) Mixed model with heading date as a covariate.

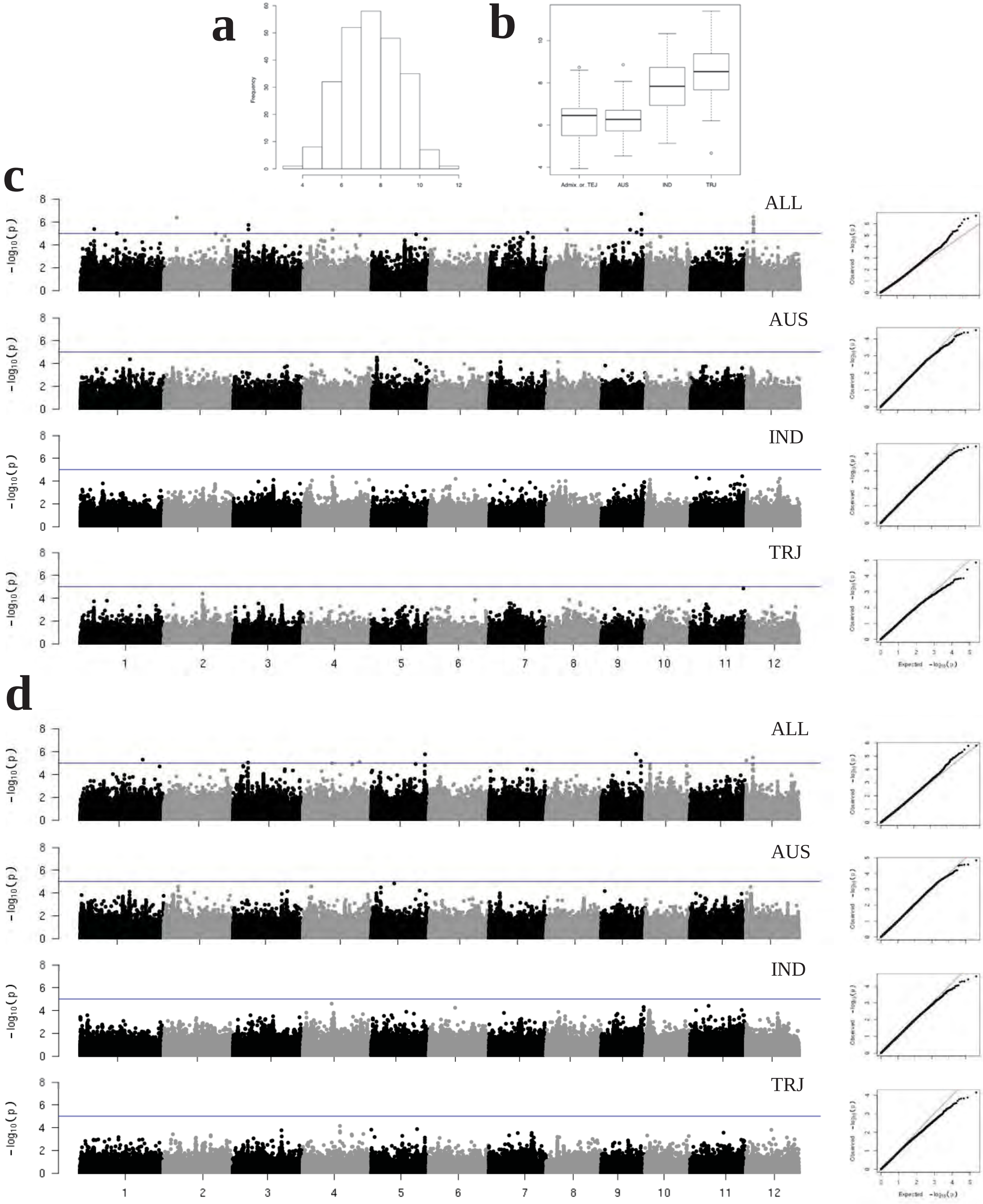

Supplementary Figure 14. Summary of GWAS results for Internode Number. (**a, b**) Phenotypic distributions. (**c**) Mixed model controlling for subpopulation structure across all varieties (ALL) and within individual subpopulations (AUS = *aus*, IND = *indica*, TRJ = *tropical japonica*). (**d**) Mixed model with heading date as a covariate.

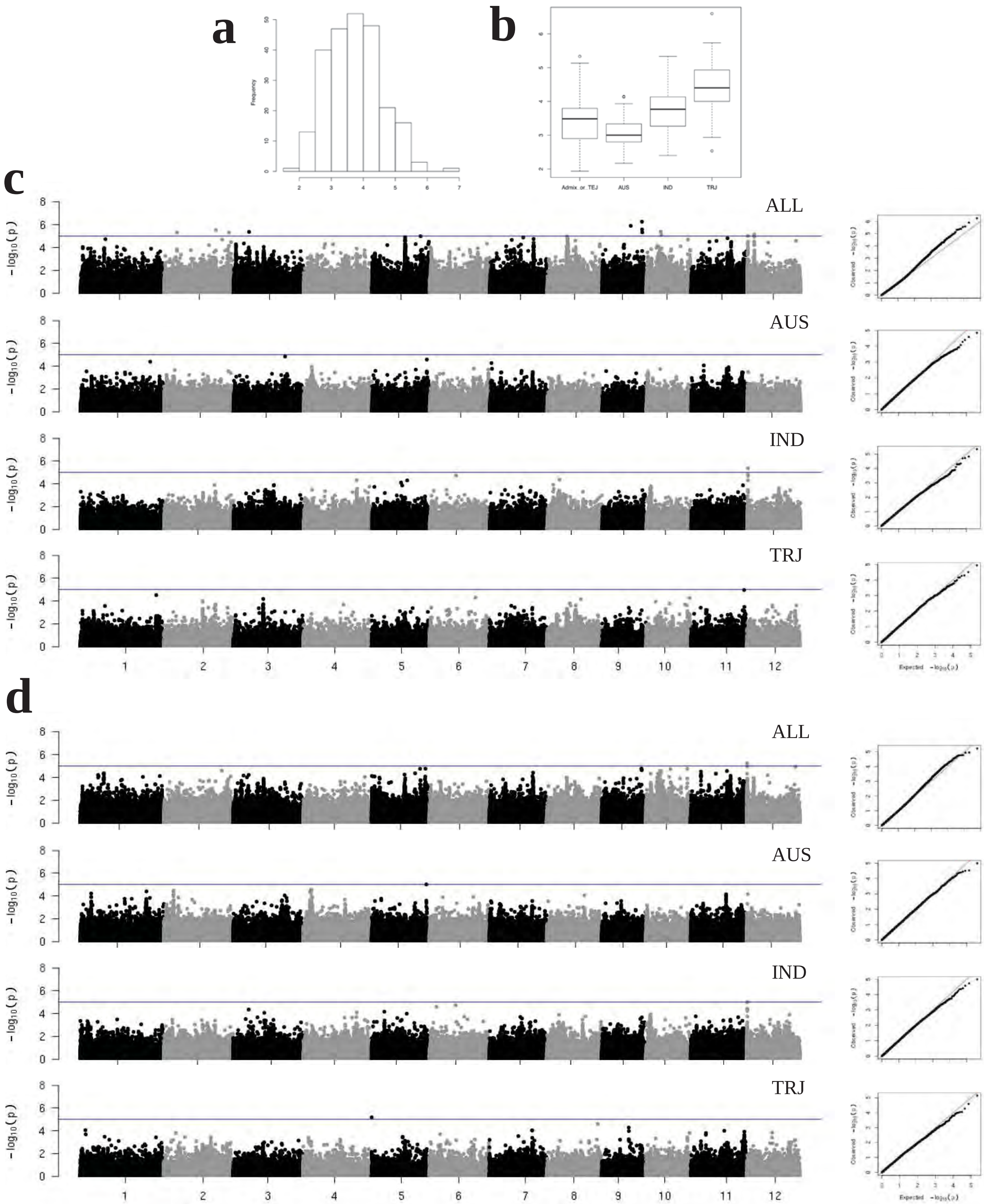

Supplementary Figure 15. Summary of GWAS results for Internode Number in the Upper Half of the Panicle. (**a, b**) Phenotypic distributions. (**c**) Mixed model controlling for subpopulation structure across all varieties (ALL) and within individual subpopulations (AUS = *aus*, IND = *indica*, TRJ = *tropical japonica*). (**d**) Mixed model with heading date as a covariate.

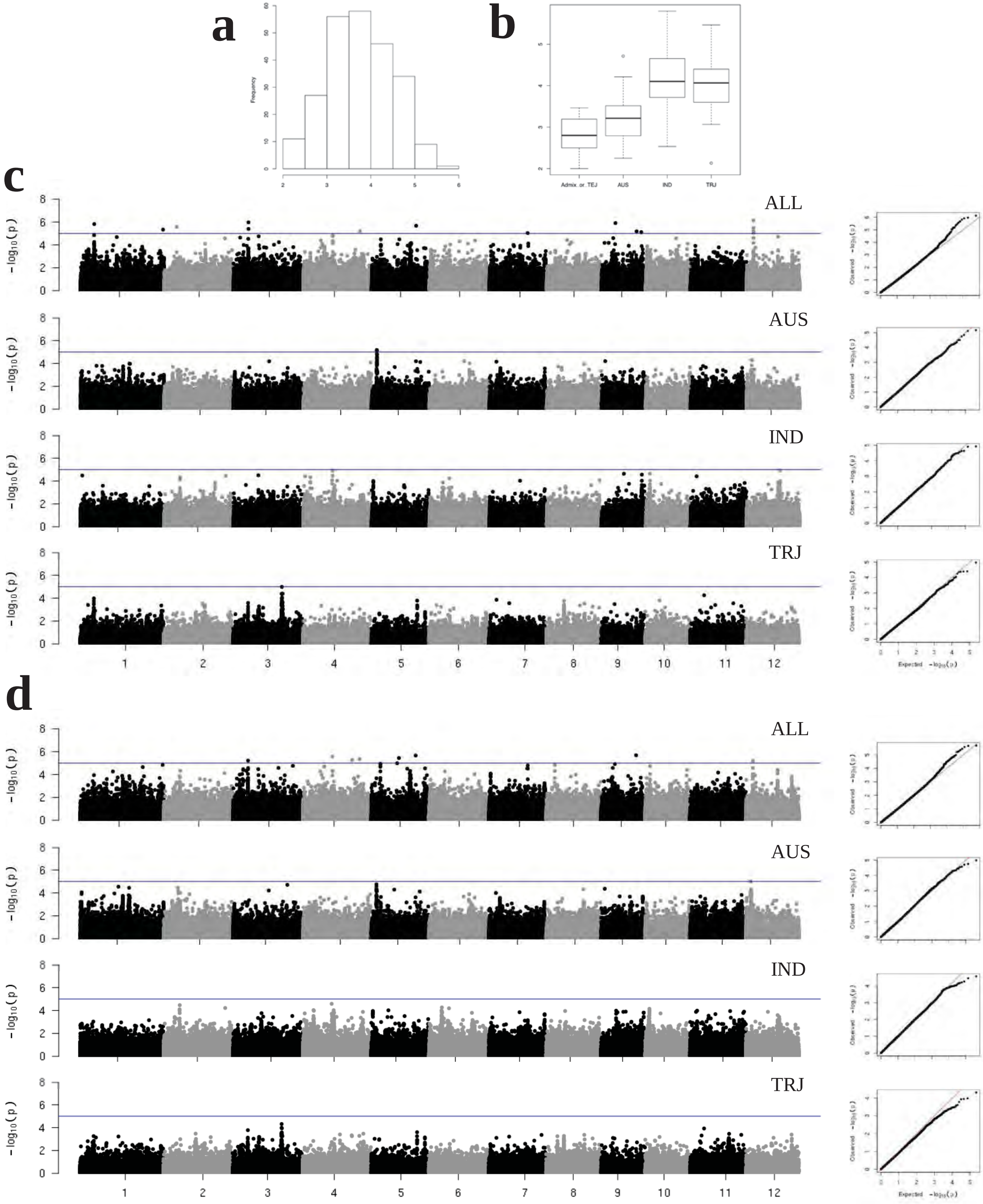

Supplementary Figure 16. Summary of GWAS results for Internode Number in the Lower Half of the Panicle. (**a, b**) Phenotypic distributions. (**c**) Mixed model controlling for subpopulation structure across all varieties (ALL) and within individual subpopulations (AUS = *aus*, IND = *indica*, TRJ = *tropical japonica*). (**d**) Mixed model with heading date as a covariate.

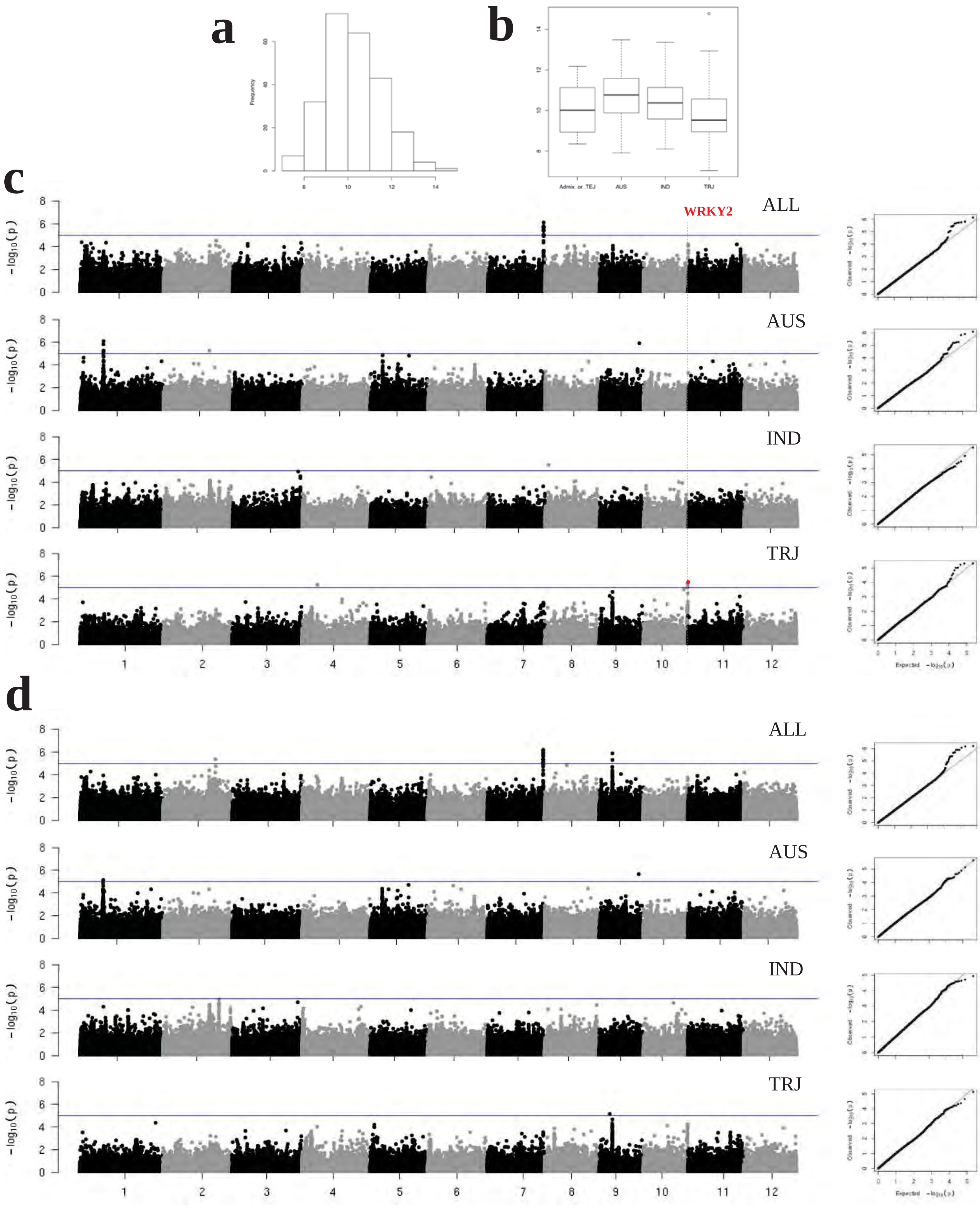

Supplementary Figure 17. Summary of GWAS results for Primary Branch Length (cm). (**a, b**) Phenotypic distributions. (**c**) Mixed model controlling for subpopulation structure across all varieties (ALL) and within individual subpopulations (AUS = *aus*, IND = *indica*, TRJ = *tropical japonica*). (**d**) Mixed model with heading date as a covariate.

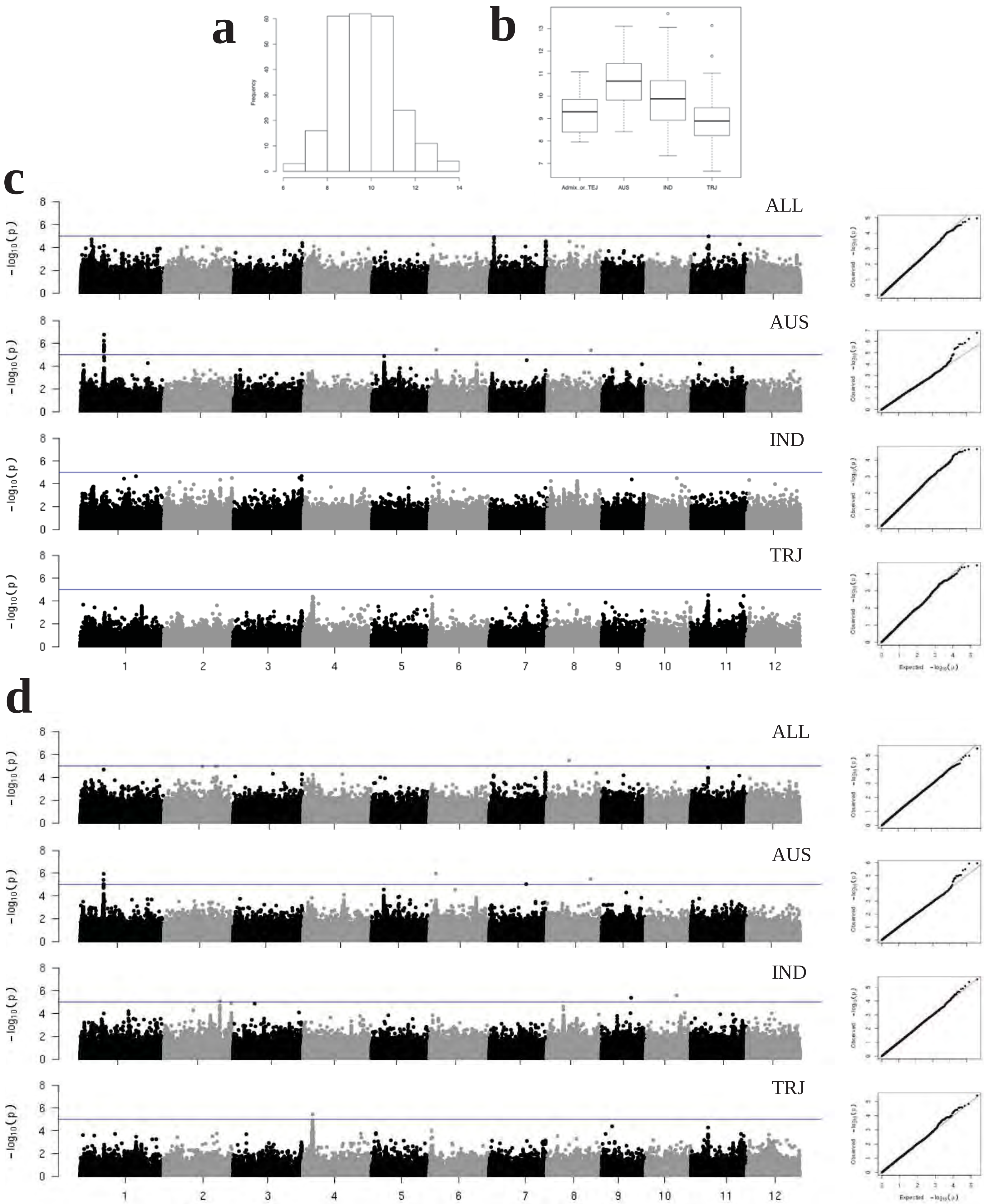

Supplementary Figure 18. Summary of GWAS results for Primary Branch Length in the Upper Half of the Panicle (cm). (**a, b**) Phenotypic distributions. (**c**) Mixed model controlling for subpopulation structure across all varieties (ALL) and within individual subpopulations (AUS = *aus*, IND = *indica*, TRJ = *tropical japonica*). (**d**) Mixed model with heading date as a covariate.

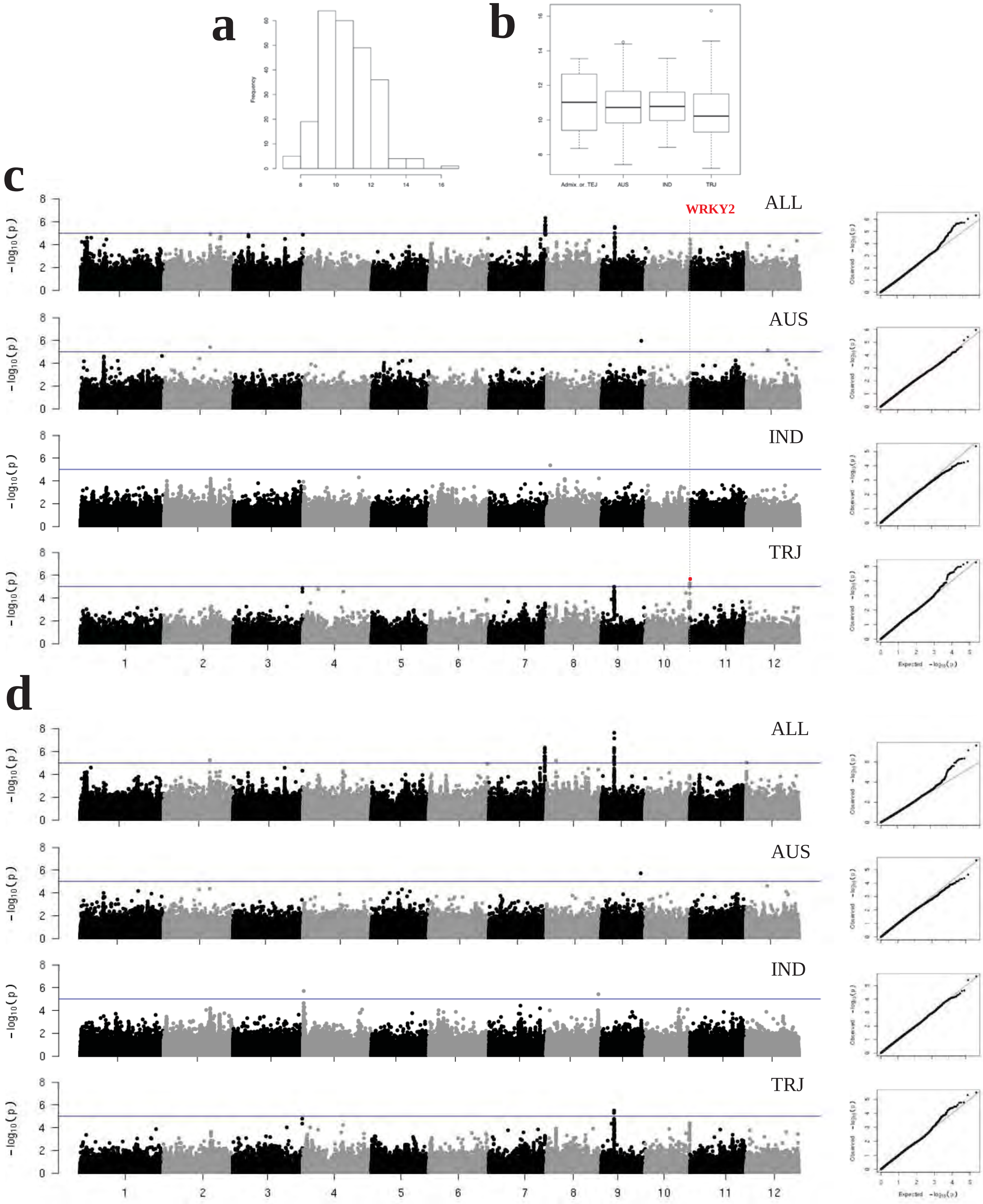

Supplementary Figure 19. Summary of GWAS results for Primary Branch Length in the Lower Half of the Panicle (cm). (**a, b**) Phenotypic distributions. (**c**) Mixed model controlling for subpopulation structure across all varieties (ALL) and within individual subpopulations (AUS = *aus*, IND = *indica*, TRJ = *tropical japonica*). (**d**) Mixed model with heading date as a covariate.

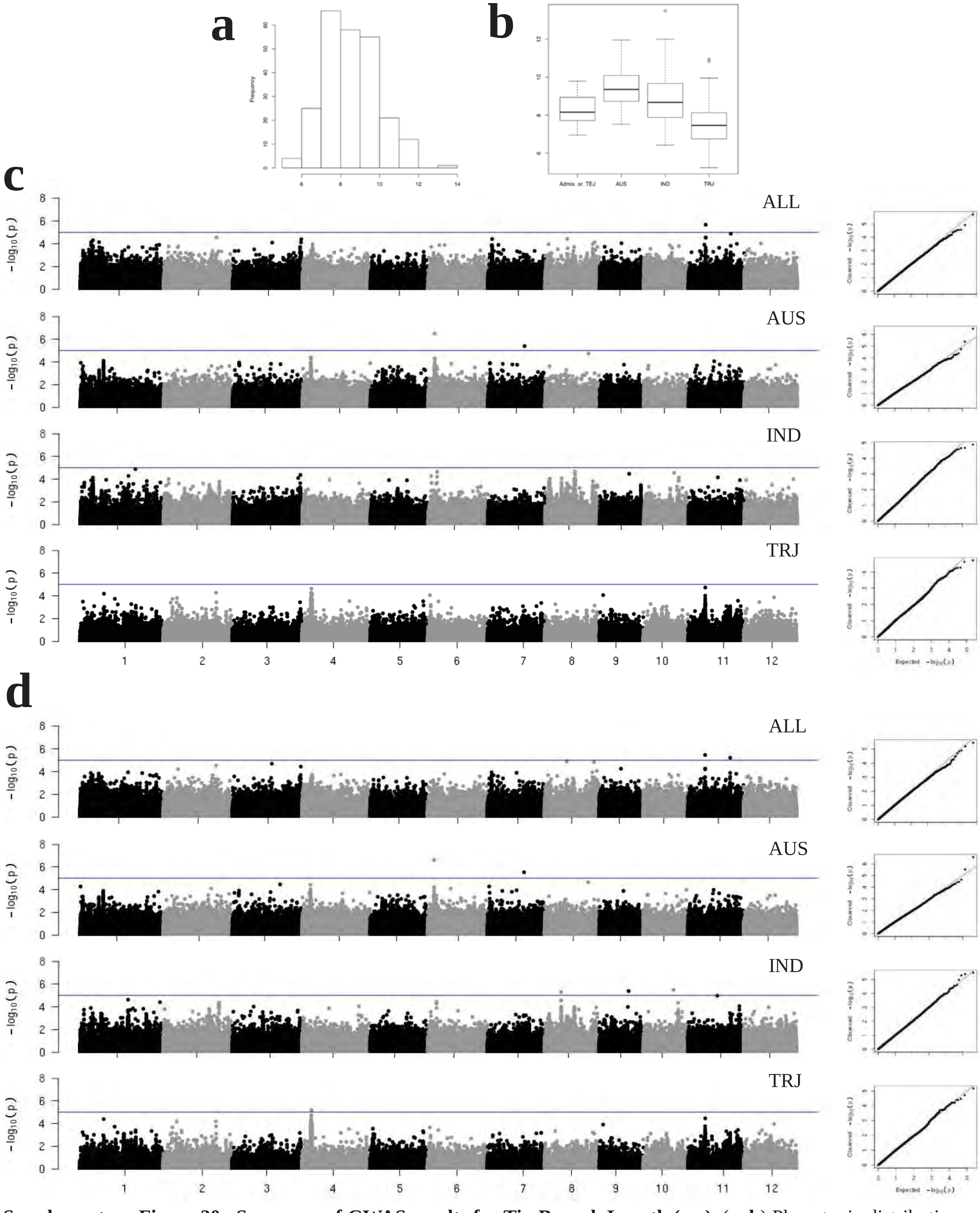

Supplementary Figure 20. Summary of GWAS results for Tip Branch Length (cm). (**a, b**) Phenotypic distributions. (**c**) Mixed model controlling for subpopulation structure across all varieties (ALL) and within individual subpopulations (AUS = *aus*, IND = *indica*, TRJ = *tropical japonica*). (**d**) Mixed model with heading date as a covariate.

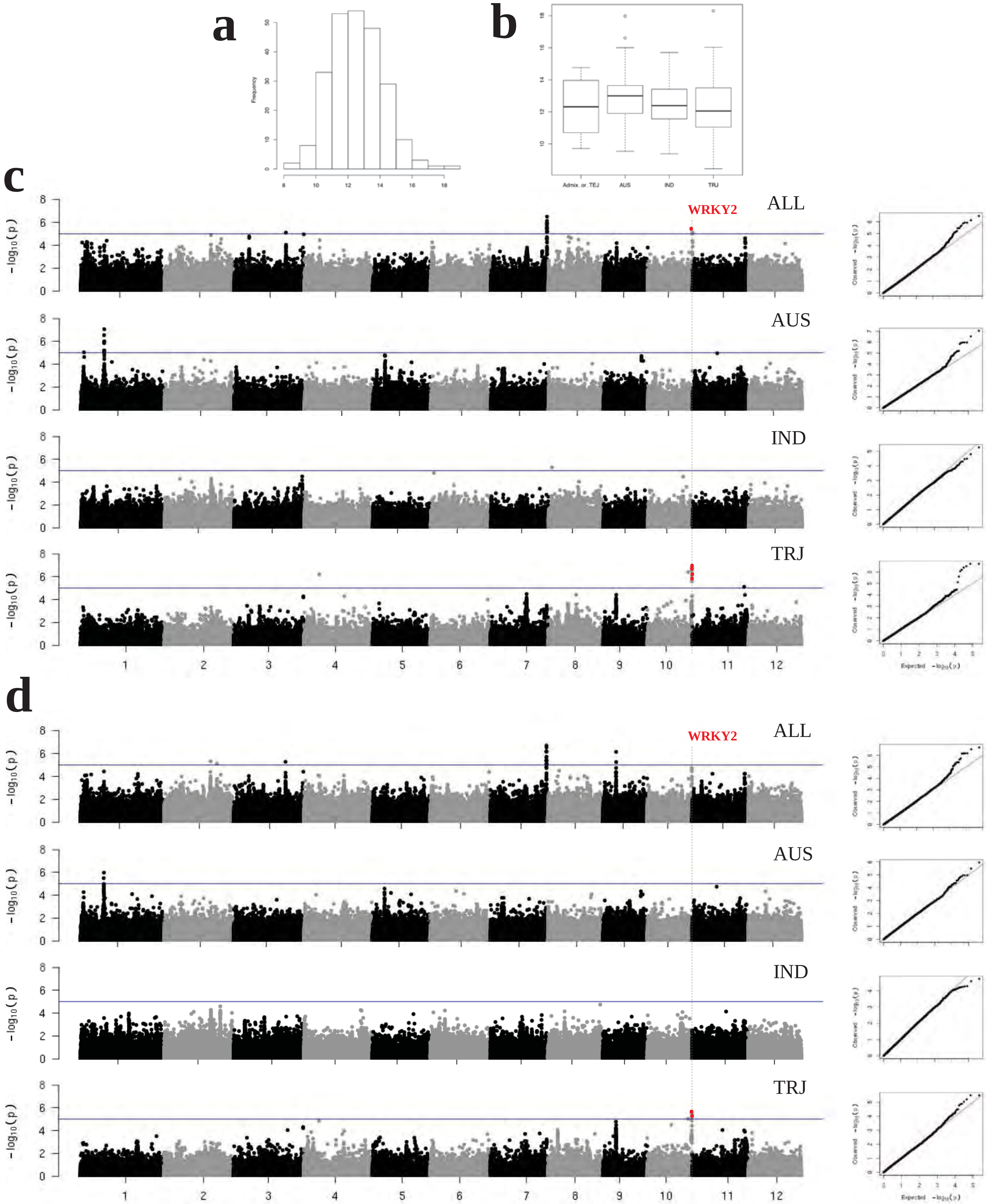

Supplementary Figure 21. Summary of GWAS results for Maximum Primary Branch Length (cm). (**a, b**) Phenotypic distributions. (**c**) Mixed model controlling for subpopulation structure across all varieties (ALL) and within individual subpopulations (AUS = *aus*, IND = *indica*, TRJ = *tropical japonica*). (**d**) Mixed model with heading date as a covariate.

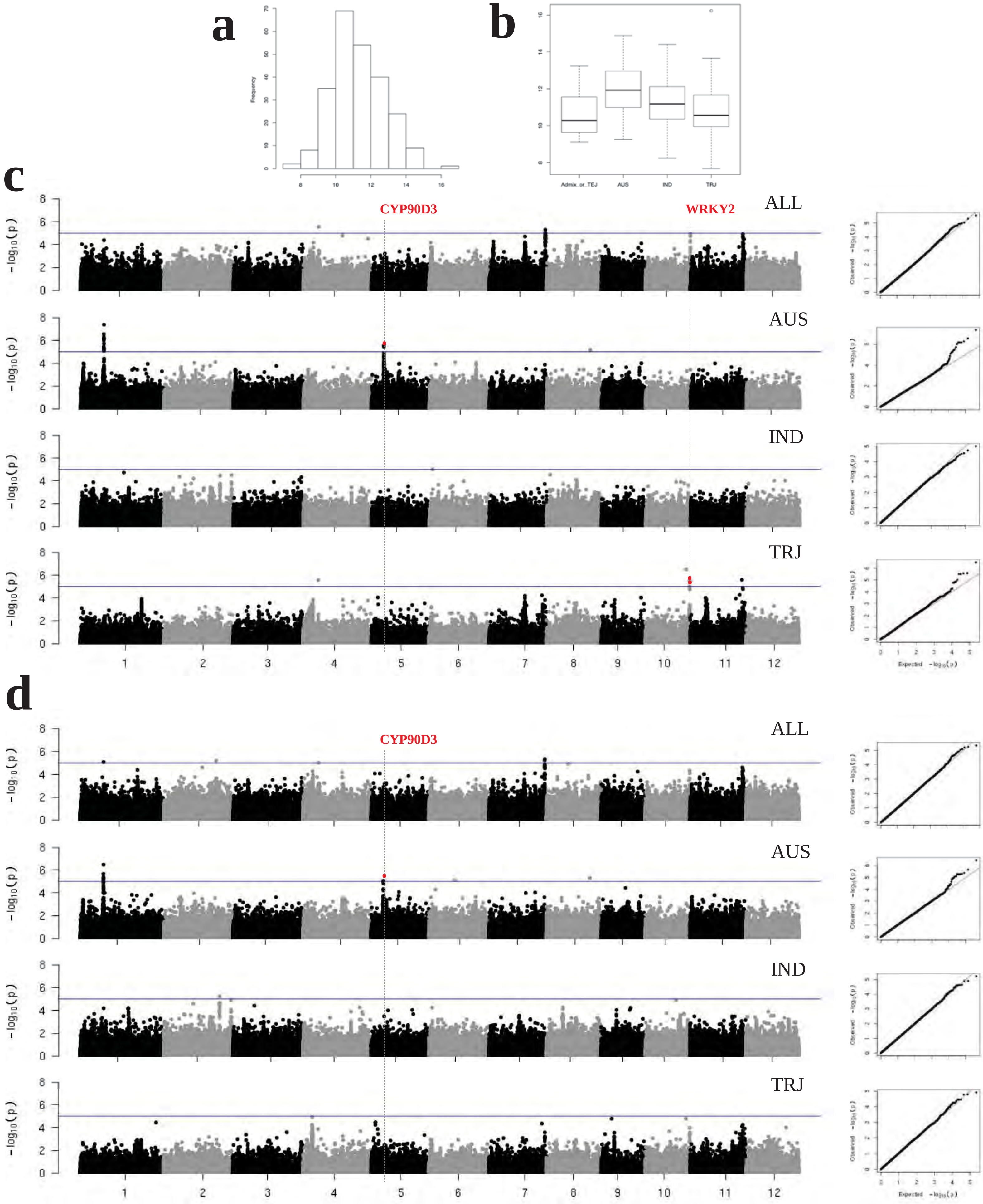

Supplementary Figure 22. Summary of GWAS results for Maximum Primary Branch Length in the Upper Half of the Panicle (cm). (**a, b**) Phenotypic distributions. (**c**) Mixed model controlling for subpopulation structure across all varieties (ALL) and within individual subpopulations (AUS = *aus*, IND = *indica*, TRJ = *tropical japonica*). (**d**) Mixed model with heading date as a covariate.

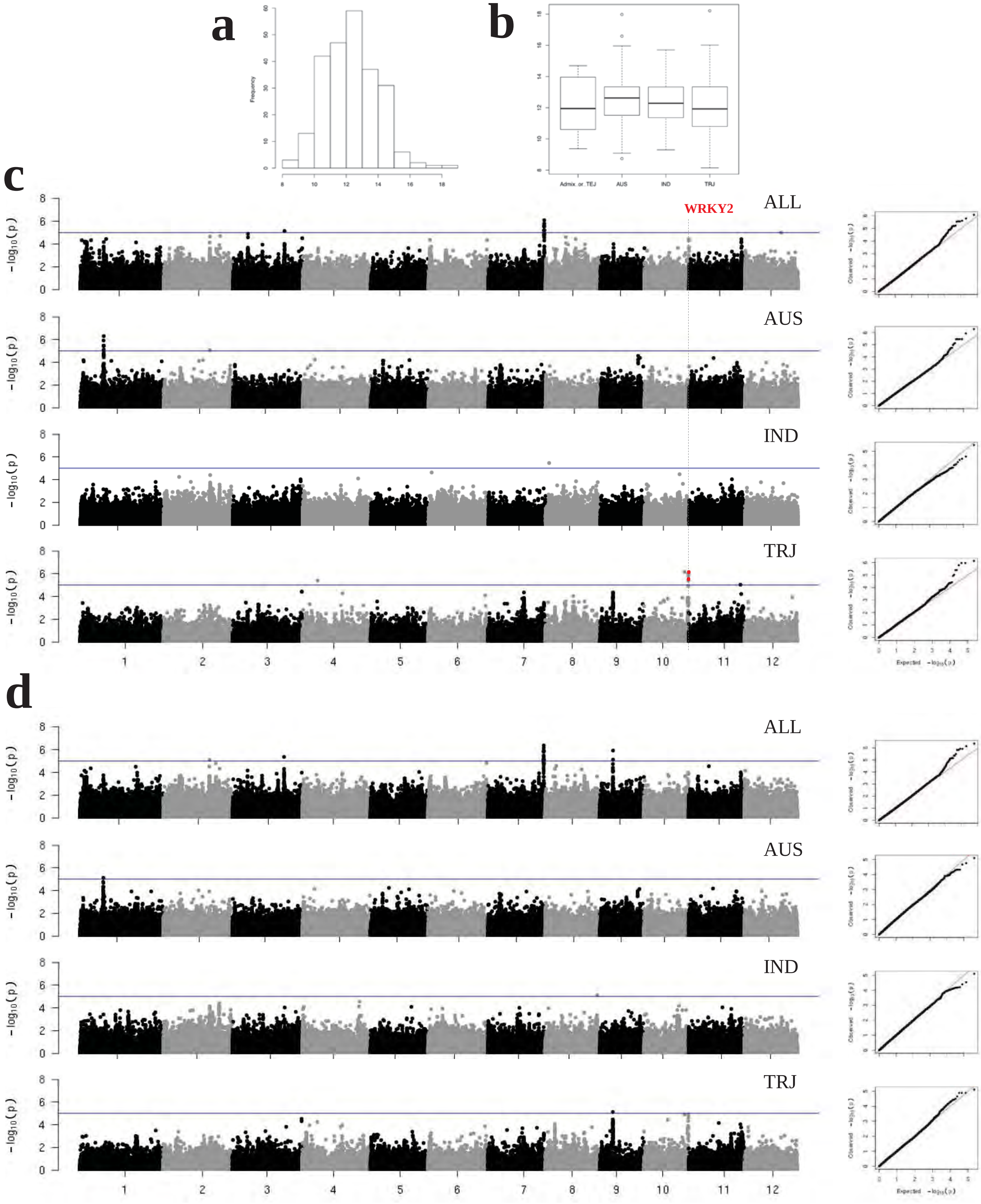

Supplementary Figure 23. Summary of GWAS results for Maximum Primary Branch Length in the Lower Half of the Panicle (cm). (**a, b**) Phenotypic distributions. (**c**) Mixed model controlling for subpopulation structure across all varieties (ALL) and within individual subpopulations (AUS = *aus*, IND = *indica*, TRJ = *tropical japonica*). (**d**) Mixed model with heading date as a covariate.

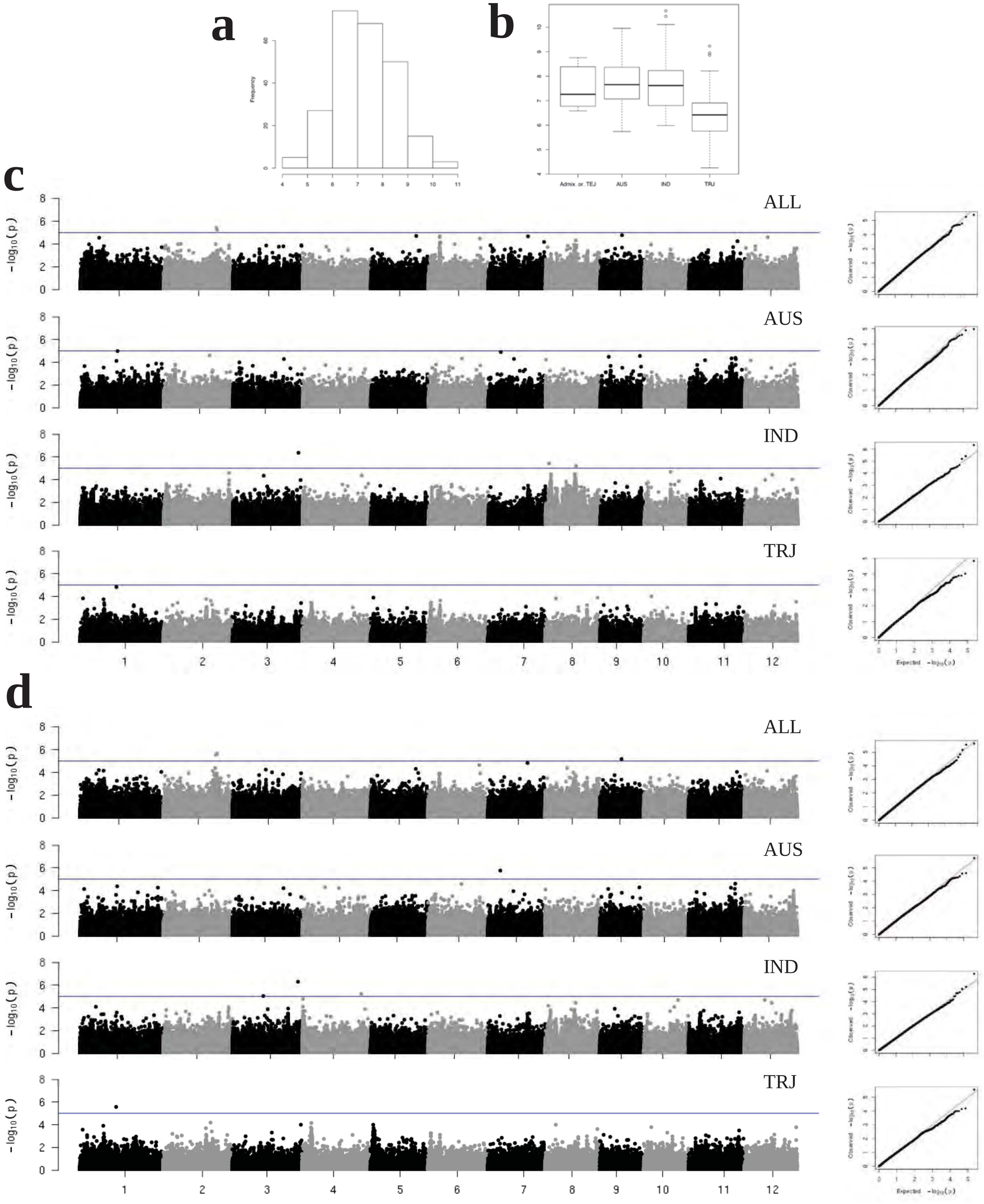

Supplementary Figure 24. Summary of GWAS results for Minimum Primary Branch Length (cm). (**a, b**) Phenotypic distributions. (**c**) Mixed model controlling for subpopulation structure across all varieties (ALL) and within individual subpopulations (AUS = *aus*, IND = *indica*, TRJ = *tropical japonica*). (**d**) Mixed model with heading date as a covariate.

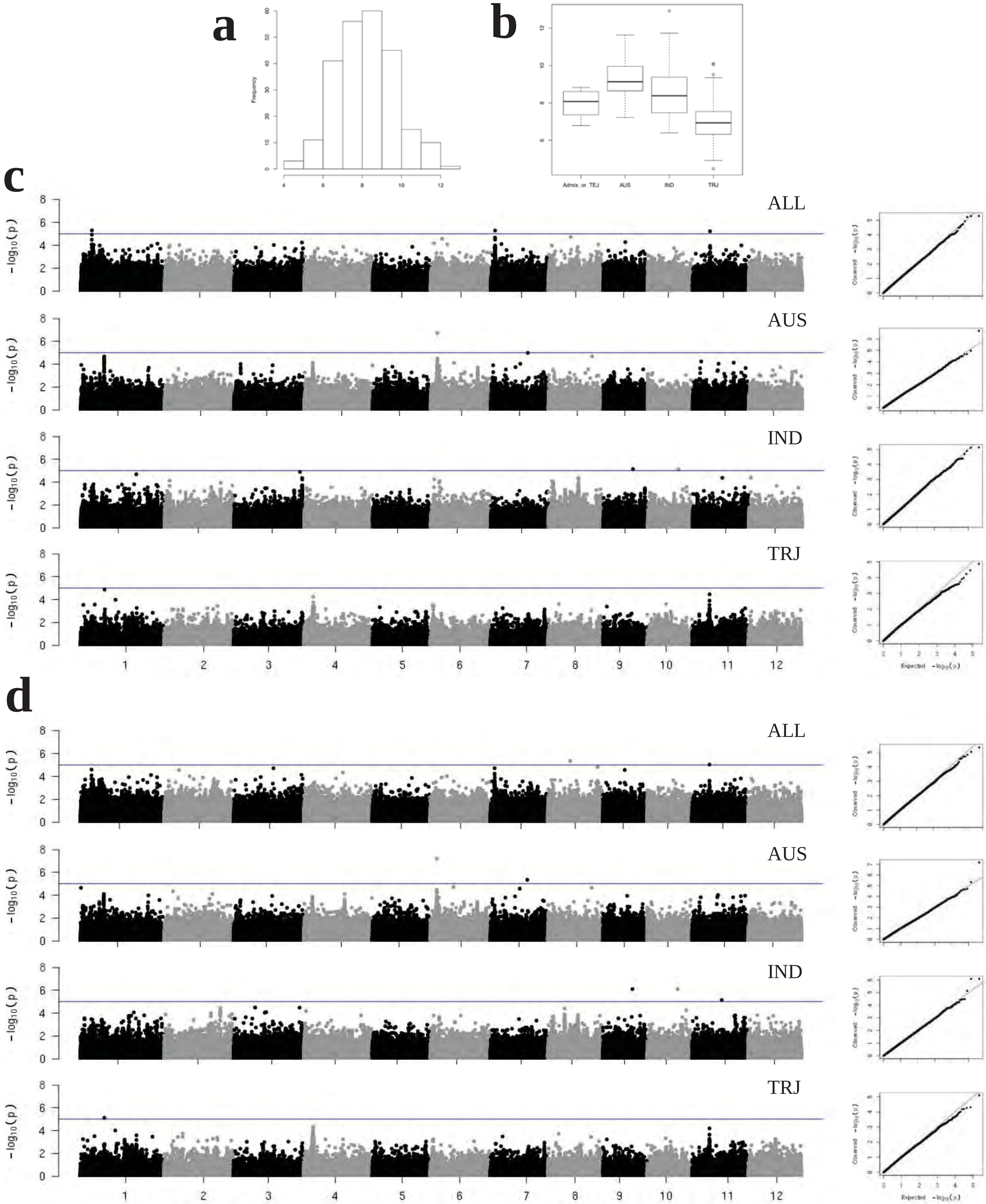

Supplementary Figure 25. Summary of GWAS results for Minimum Primary Branch Length in the Upper Half of the Panicle (cm). (**a, b**) Phenotypic distributions. (**c**) Mixed model controlling for subpopulation structure across all varieties (ALL) and within individual subpopulations (AUS = *aus*, IND = *indica*, TRJ = *tropical japonica*). (**d**) Mixed model with heading date as a covariate.

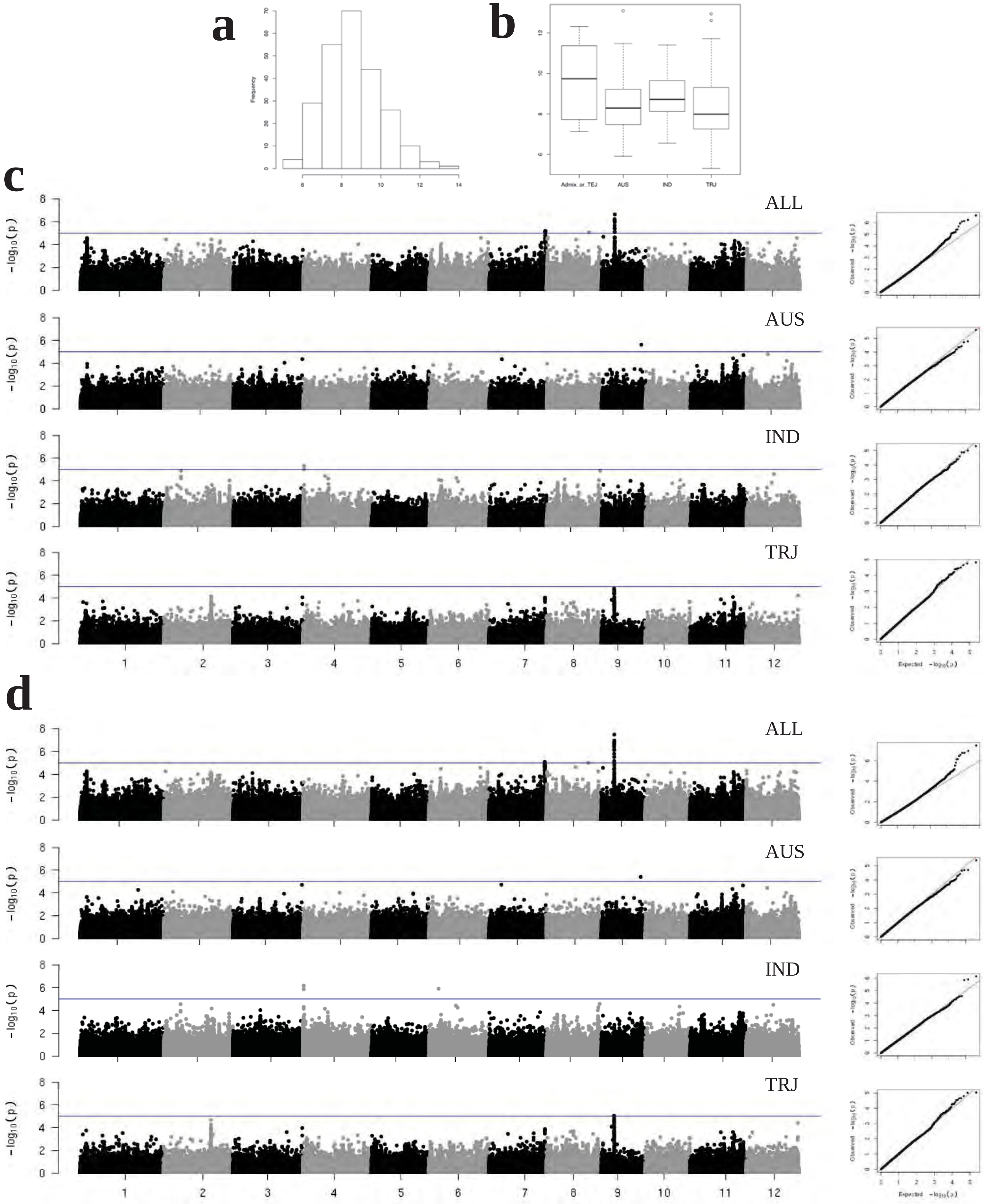

Supplementary Figure 26. Summary of GWAS results for Minimum Primary Branch Length in the Lower Half of the Panicle (cm). (**a, b**) Phenotypic distributions. (**c**) Mixed model controlling for subpopulation structure across all varieties (ALL) and within individual subpopulations (AUS = *aus*, IND = *indica*, TRJ = *tropical japonica*). (**d**) Mixed model with heading date as a covariate.

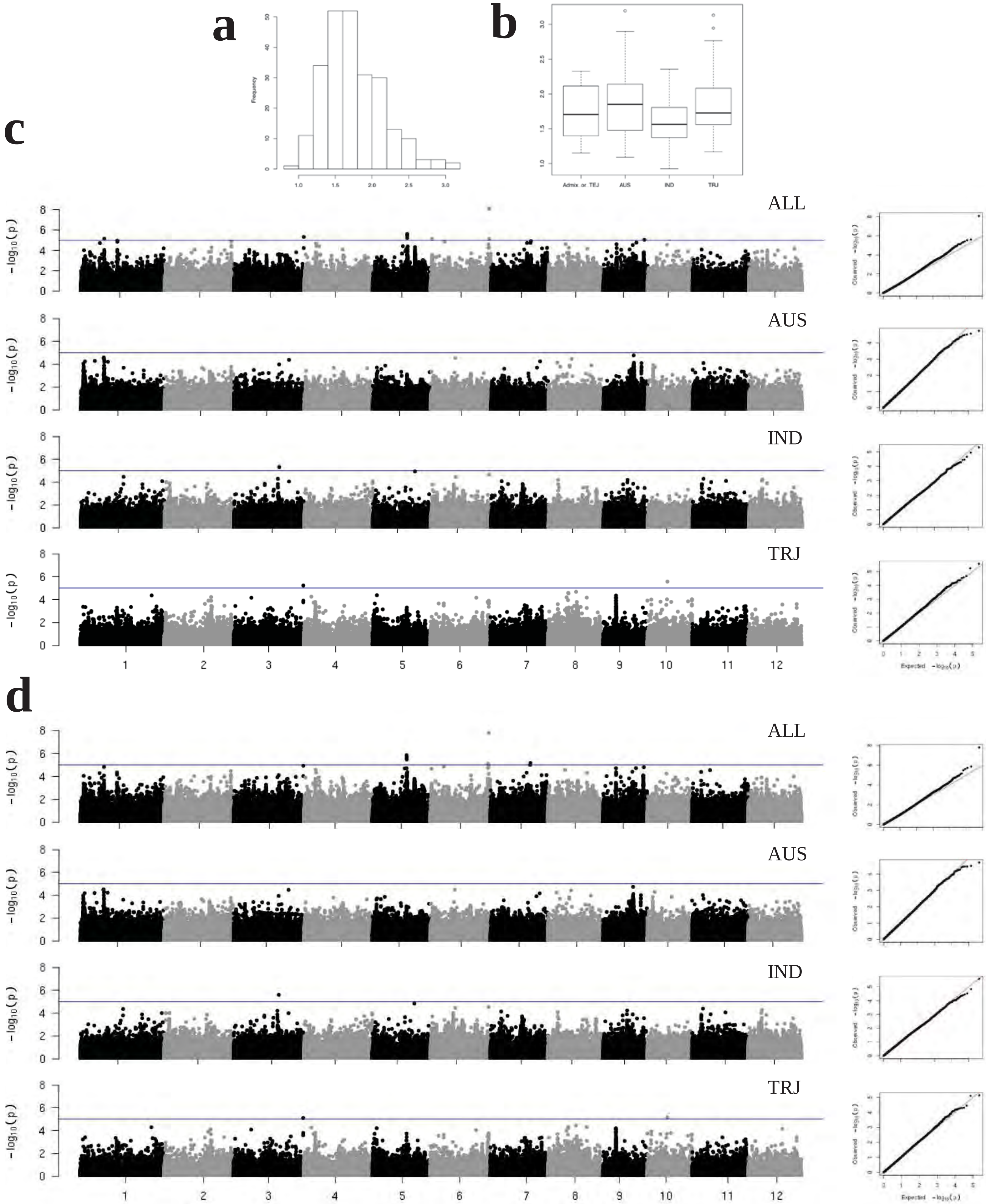

Supplementary Figure 27. Summary of GWAS results for Primary Branch Length Standard Deviation (cm). (**a, b**) Phenotypic distributions. (**c**) Mixed model controlling for subpopulation structure across all varieties (ALL) and within individual subpopulations (AUS = *aus*, IND = *indica*, TRJ = *tropical japonica*). (**d**) Mixed model with heading date as a covariate.

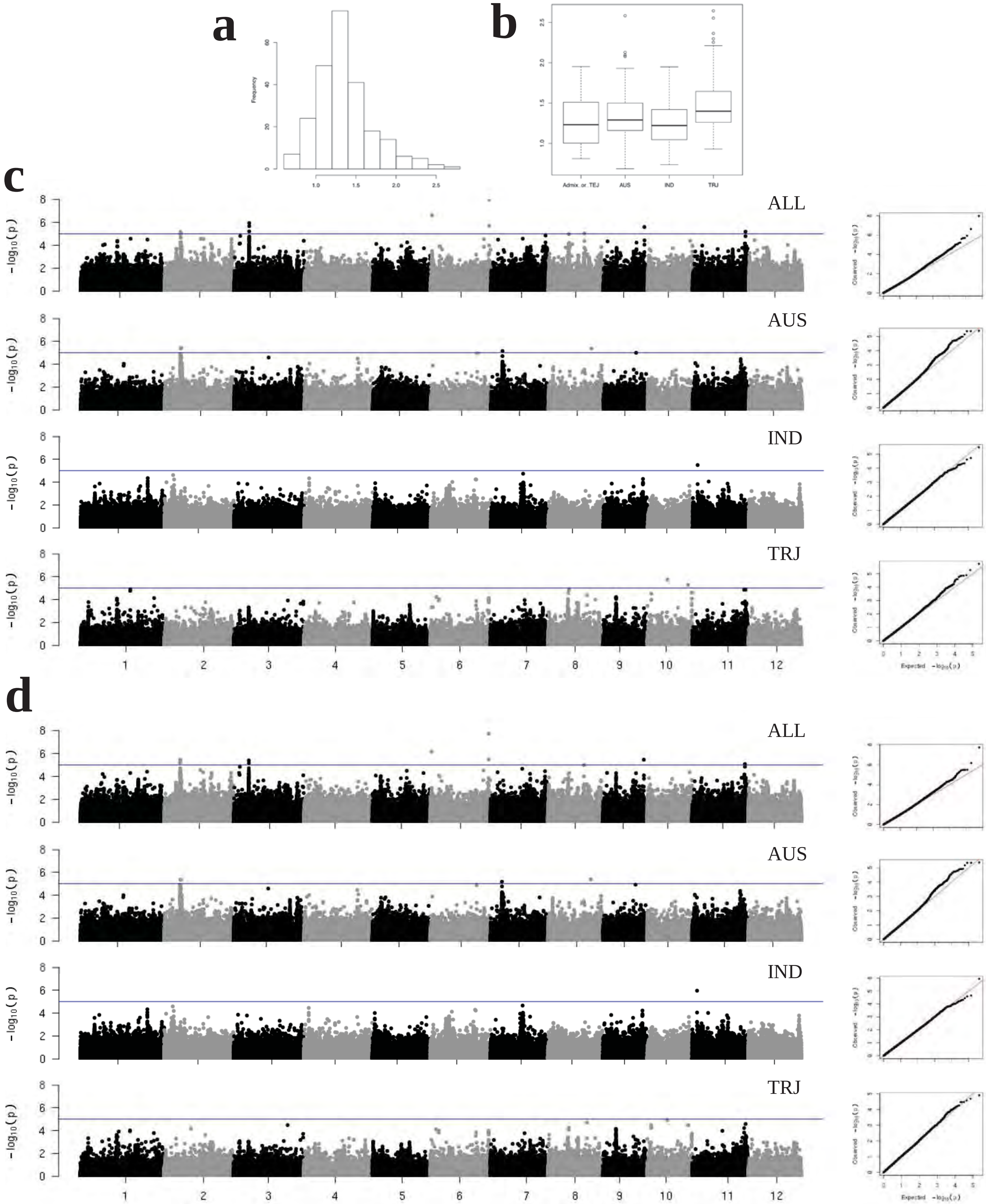

Supplementary Figure 28. Summary of GWAS results for Primary Branch Length Standard Deviation in the Upper Half of the Panicle (cm). (**a, b**) Phenotypic distributions. (**c**) Mixed model controlling for subpopulation structure across all varieties (ALL) and within individual subpopulations (AUS = *aus*, IND = *indica*, TRJ = *tropical japonica*). (**d**) Mixed model with heading date as a covariate.

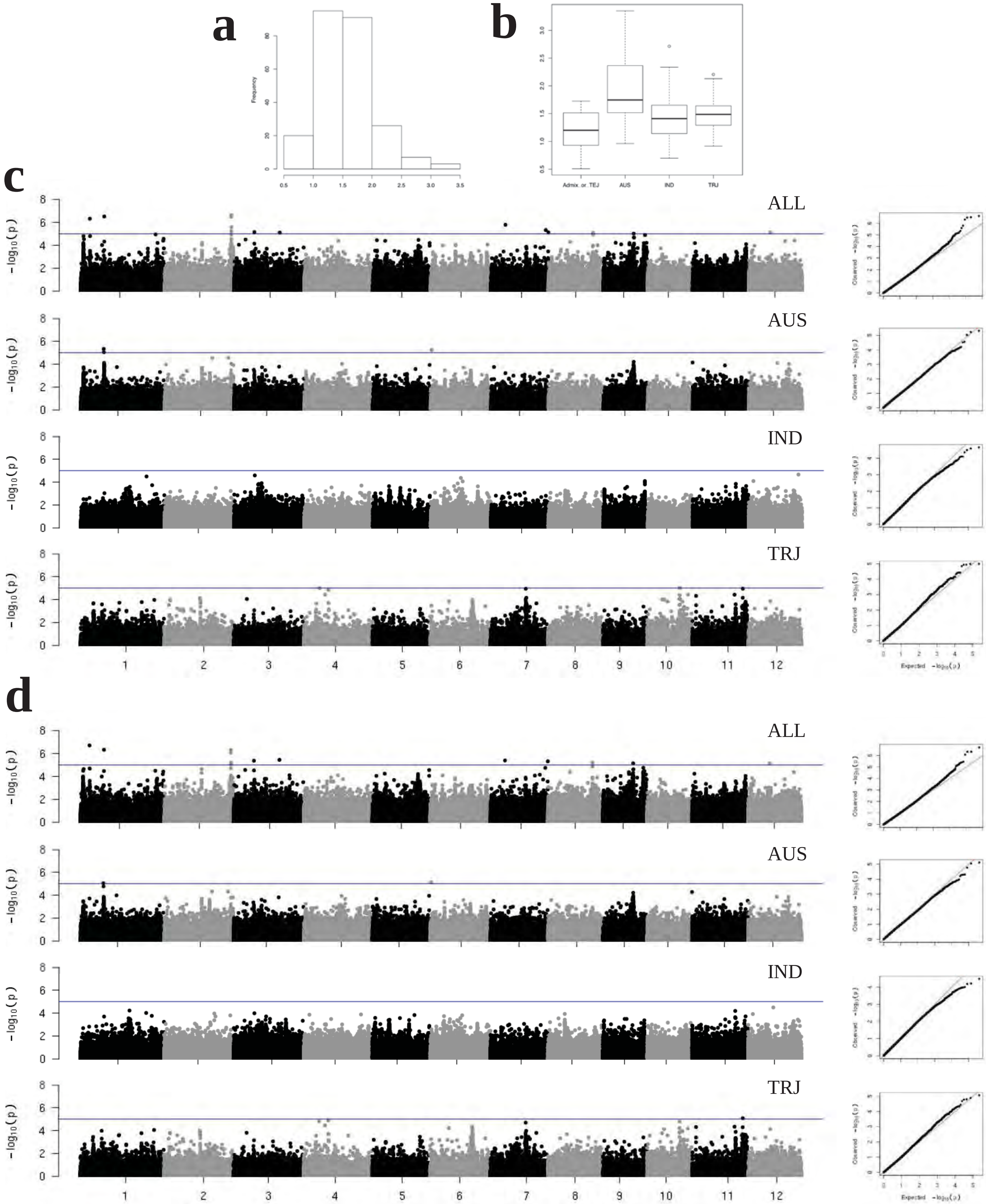

Supplementary Figure 29. Summary of GWAS results for Primary Branch Length Standard Deviation in the Lower Half of the Panicle (cm). (**a, b**) Phenotypic distributions. (**c**) Mixed model controlling for subpopulation structure across all varieties (ALL) and within individual subpopulations (AUS = *aus*, IND = *indica*, TRJ = *tropical japonica*). (**d**) Mixed model with heading date as a covariate.

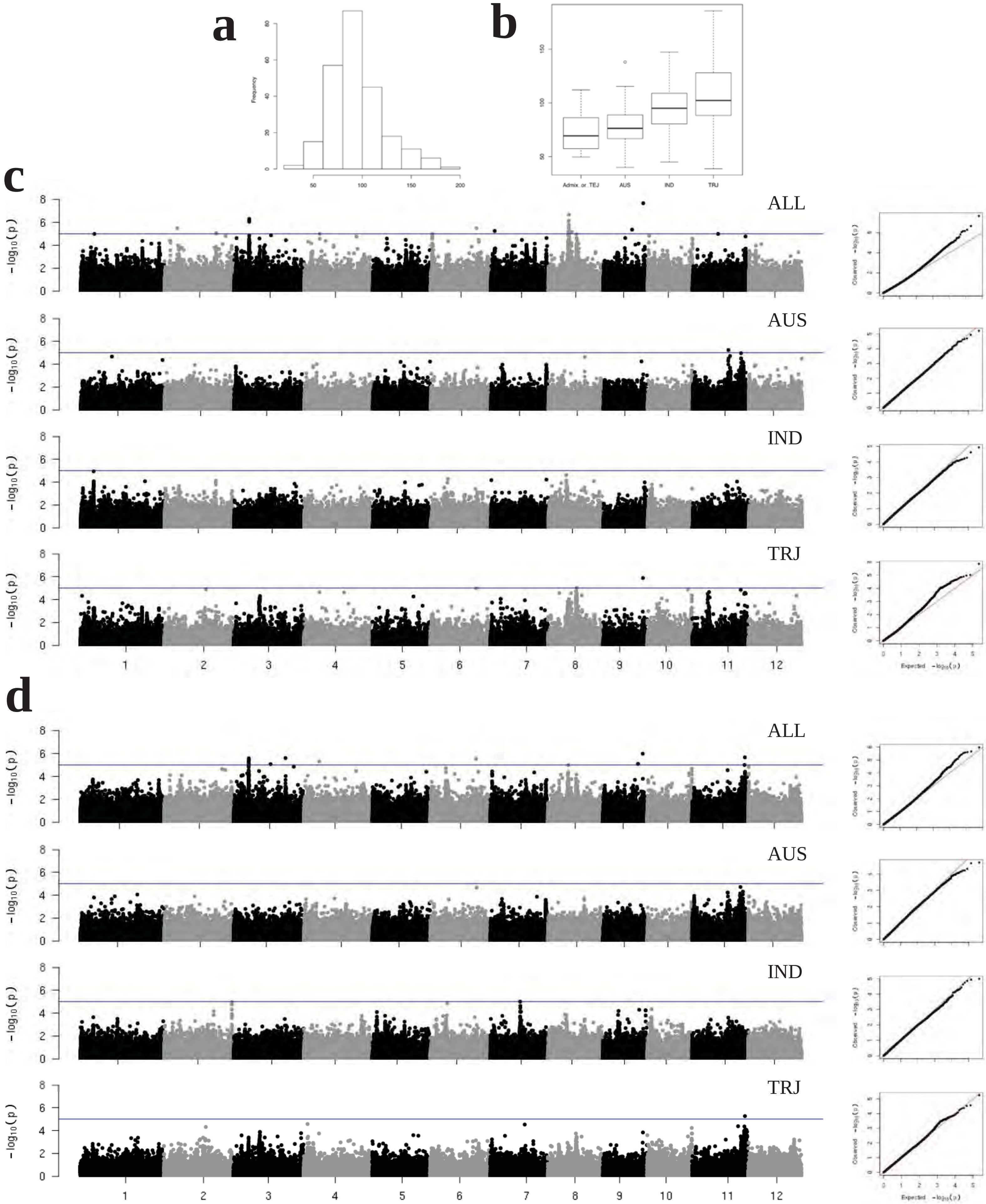

Supplementary Figure 30. Summary of GWAS results for Total Primary Branch Length (cm). (**a, b**) Phenotypic distributions. (**c**) Mixed model controlling for subpopulation structure across all varieties (ALL) and within individual subpopulations (AUS = *aus*, IND = *indica*, TRJ = *tropical japonica*). (**d**) Mixed model with heading date as a covariate.

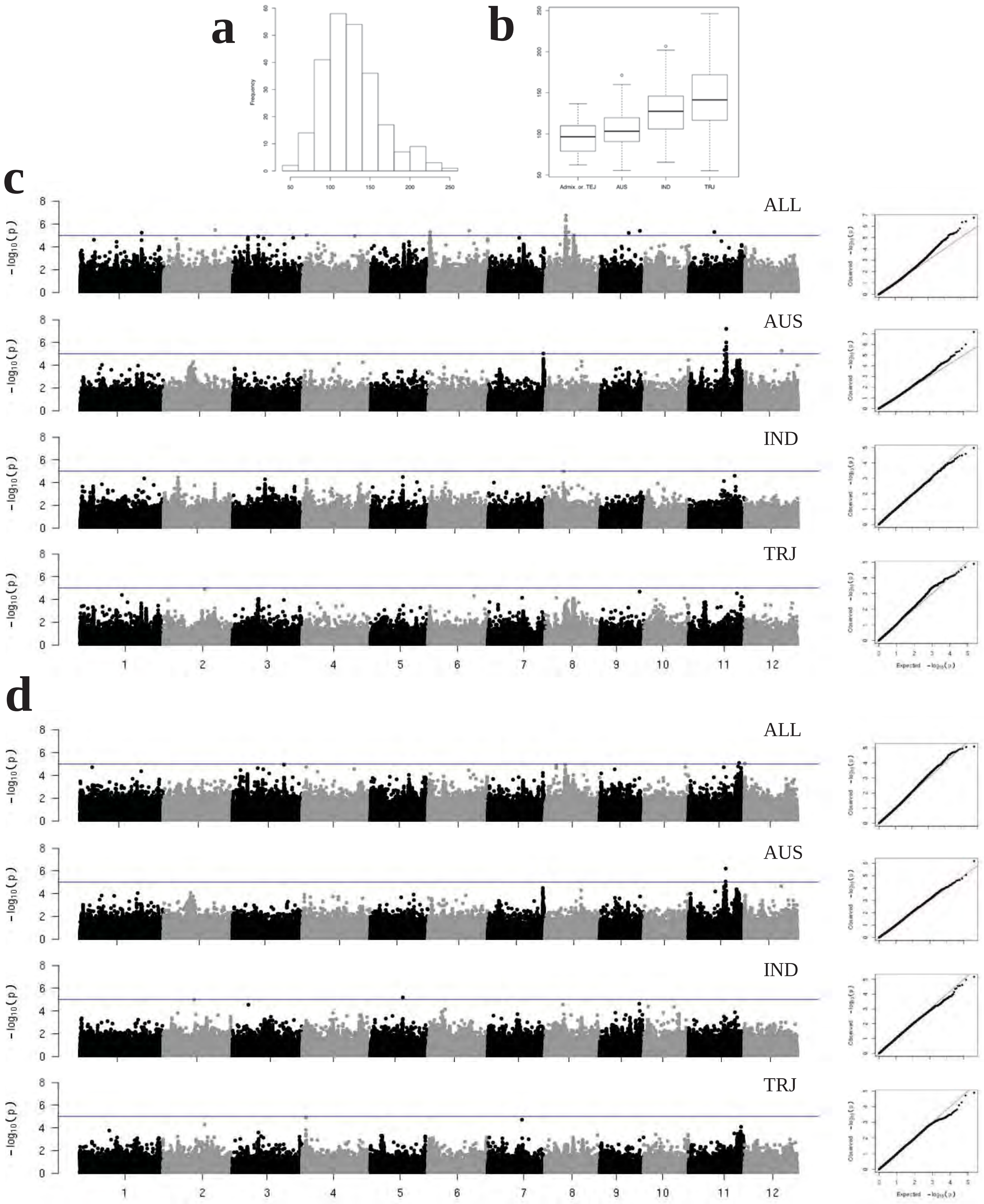

Supplementary Figure 31. Summary of GWAS results for Maximum Total Primary Branch Length (cm). (**a, b**) Phenotypic distributions. (**c**) Mixed model controlling for subpopulation structure across all varieties (ALL) and within individual subpopulations (AUS = *aus*, IND = *indica*, TRJ = *tropical japonica*). (**d**) Mixed model with heading date as a covariate.

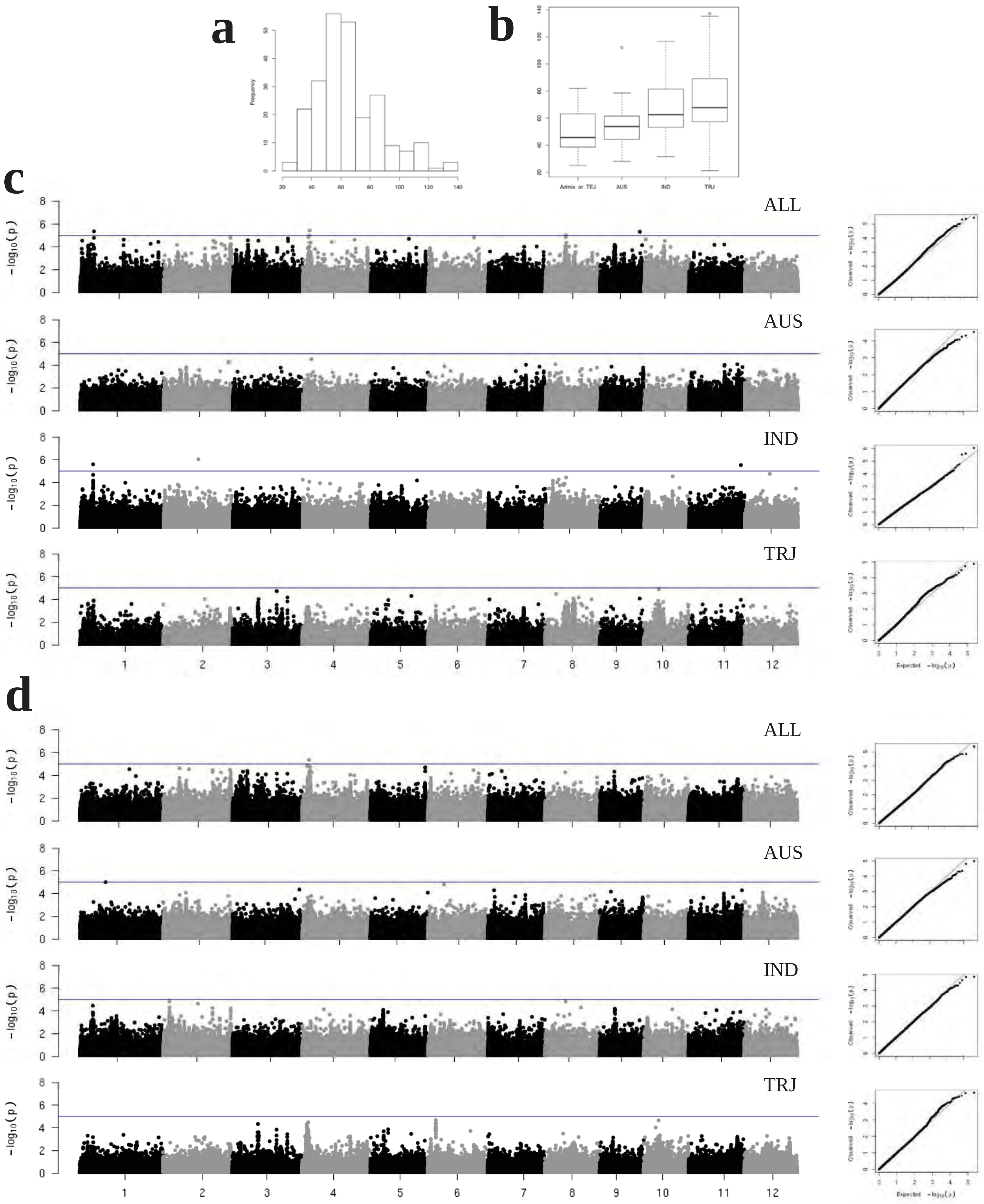

Supplementary Figure 32. Summary of GWAS results for Minimum Total Primary Branch Length (cm). (**a, b**) Phenotypic distributions. (**c**) Mixed model controlling for subpopulation structure across all varieties (ALL) and within individual subpopulations (AUS = *aus*, IND = *indica*, TRJ = *tropical japonica*). (**d**) Mixed model with heading date as a covariate.

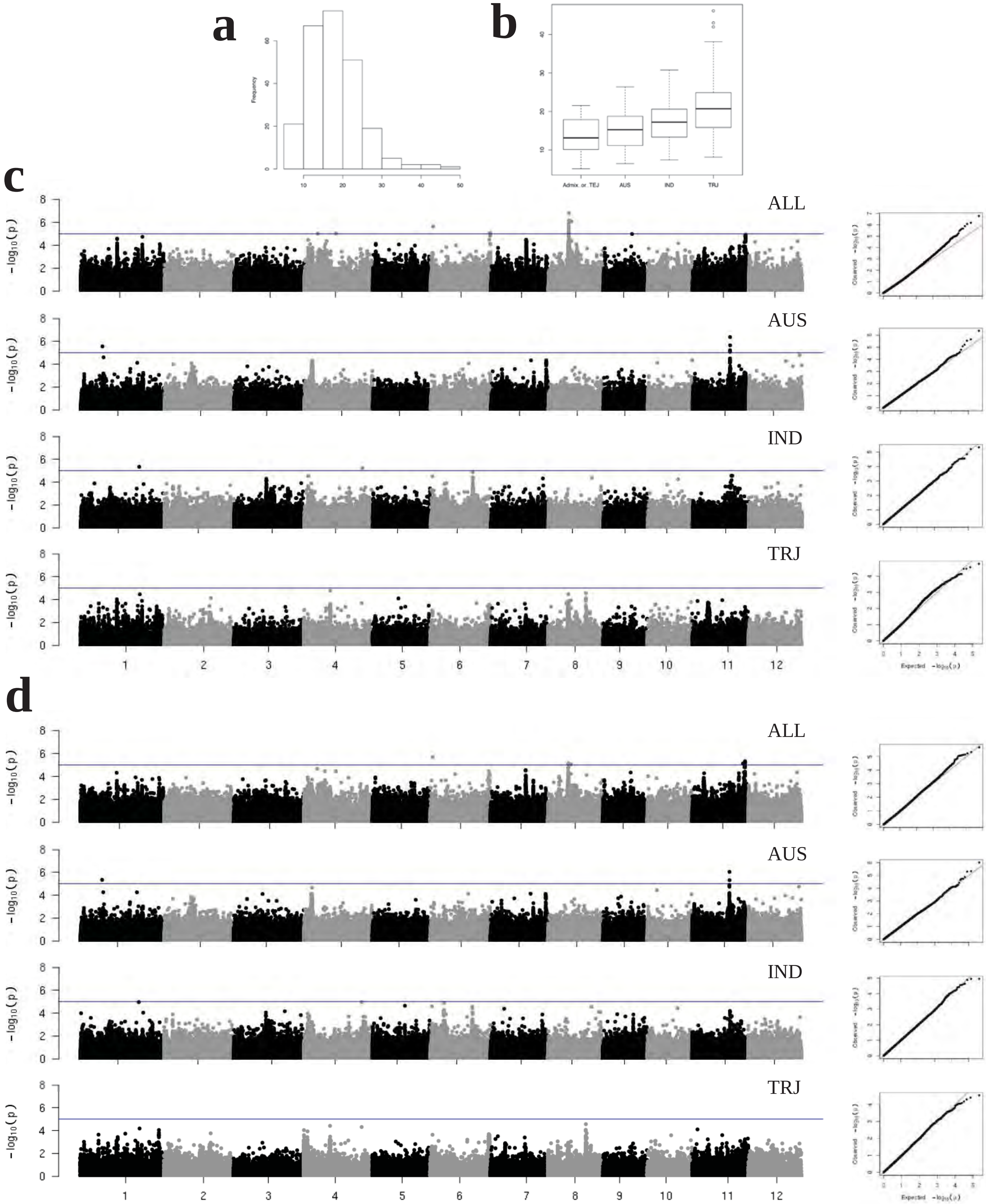

Supplementary Figure 33. Summary of GWAS results for Total Primary Branch Length Standard Deviation (cm). (**a, b**) Phenotypic distributions. (**c**) Mixed model controlling for subpopulation structure across all varieties (ALL) and within individual subpopulations (AUS = *aus*, IND = *indica*, TRJ = *tropical japonica*). (**d**) Mixed model with heading date as a covariate.

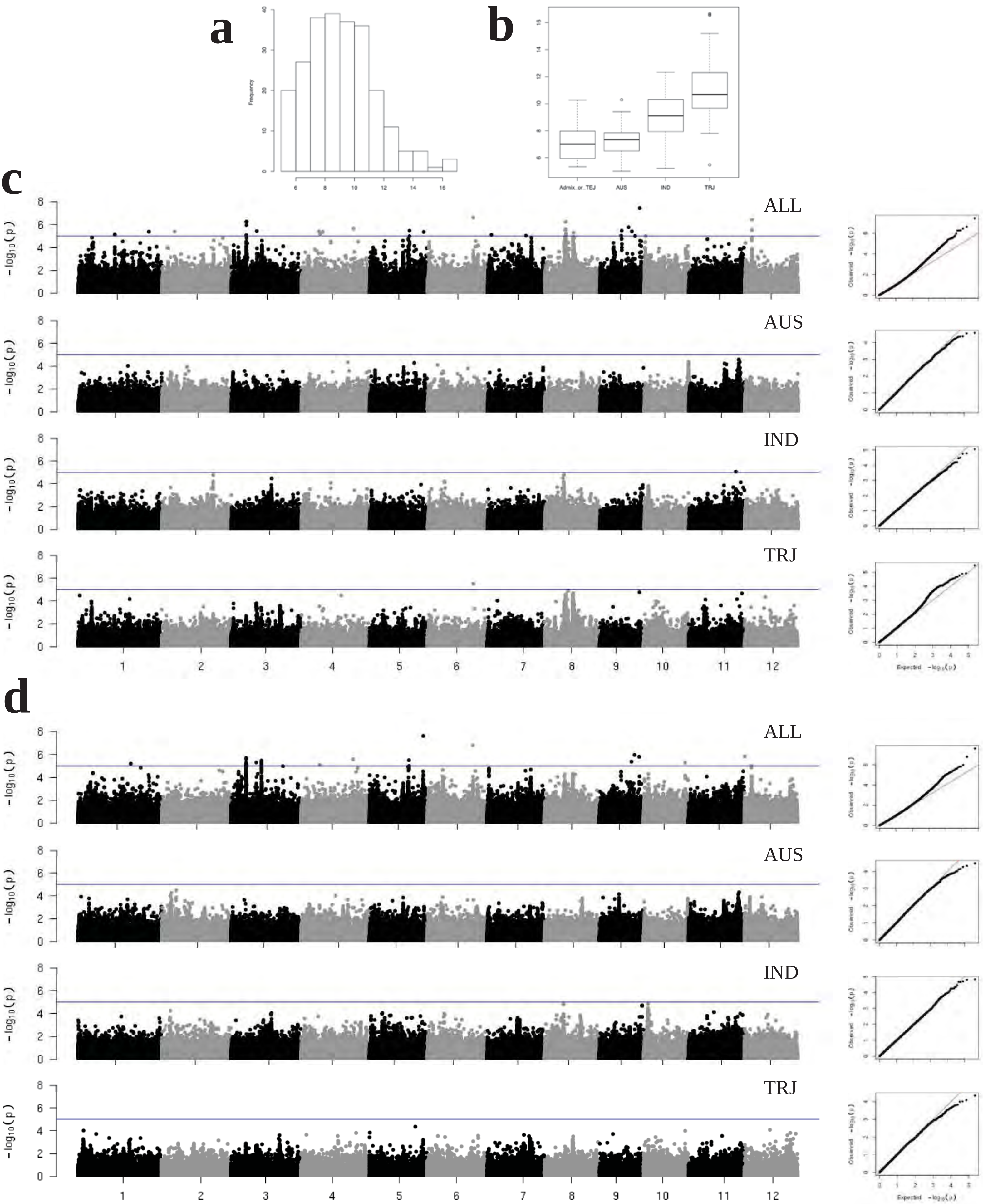

Supplementary Figure 34. Summary of GWAS results for Primary Branch Number. (**a, b**) Phenotypic distributions. (**c**) Mixed model controlling for subpopulation structure across all varieties (ALL) and within individual subpopulations (AUS = *aus*, IND = *indica*, TRJ = *tropical japonica*). (**d**) Mixed model with heading date as a covariate.

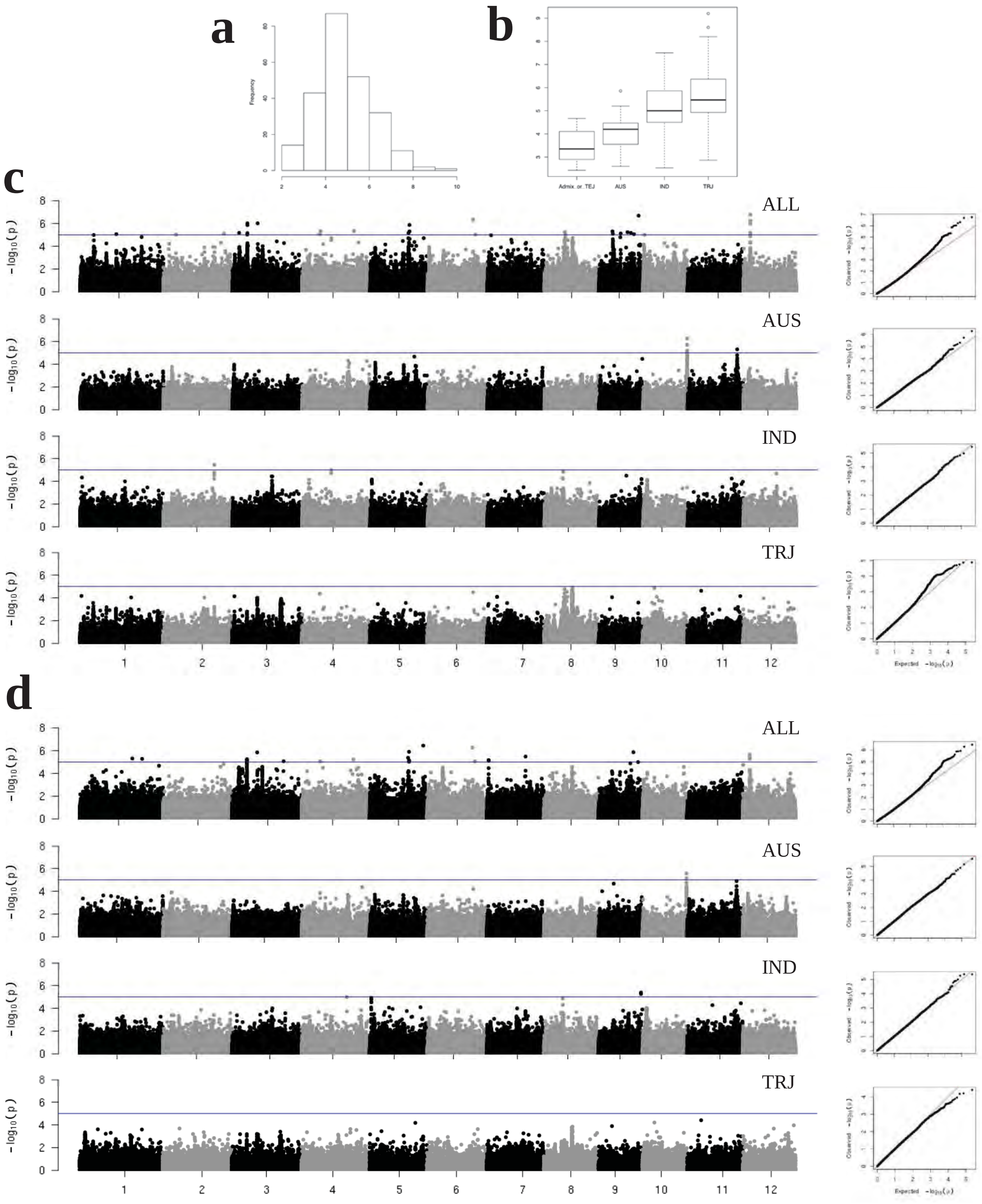

Supplementary Figure 35. Summary of GWAS results Primary Branch Number in the Lower Half of the Panicle. (**a, b**) Phenotypic distributions. (**c**) Mixed model controlling for subpopulation structure across all varieties (ALL) and within individual subpopulations (AUS = *aus*, IND = *indica*, TRJ = *tropical japonica*). (**d**) Mixed model with heading date as a covariate.

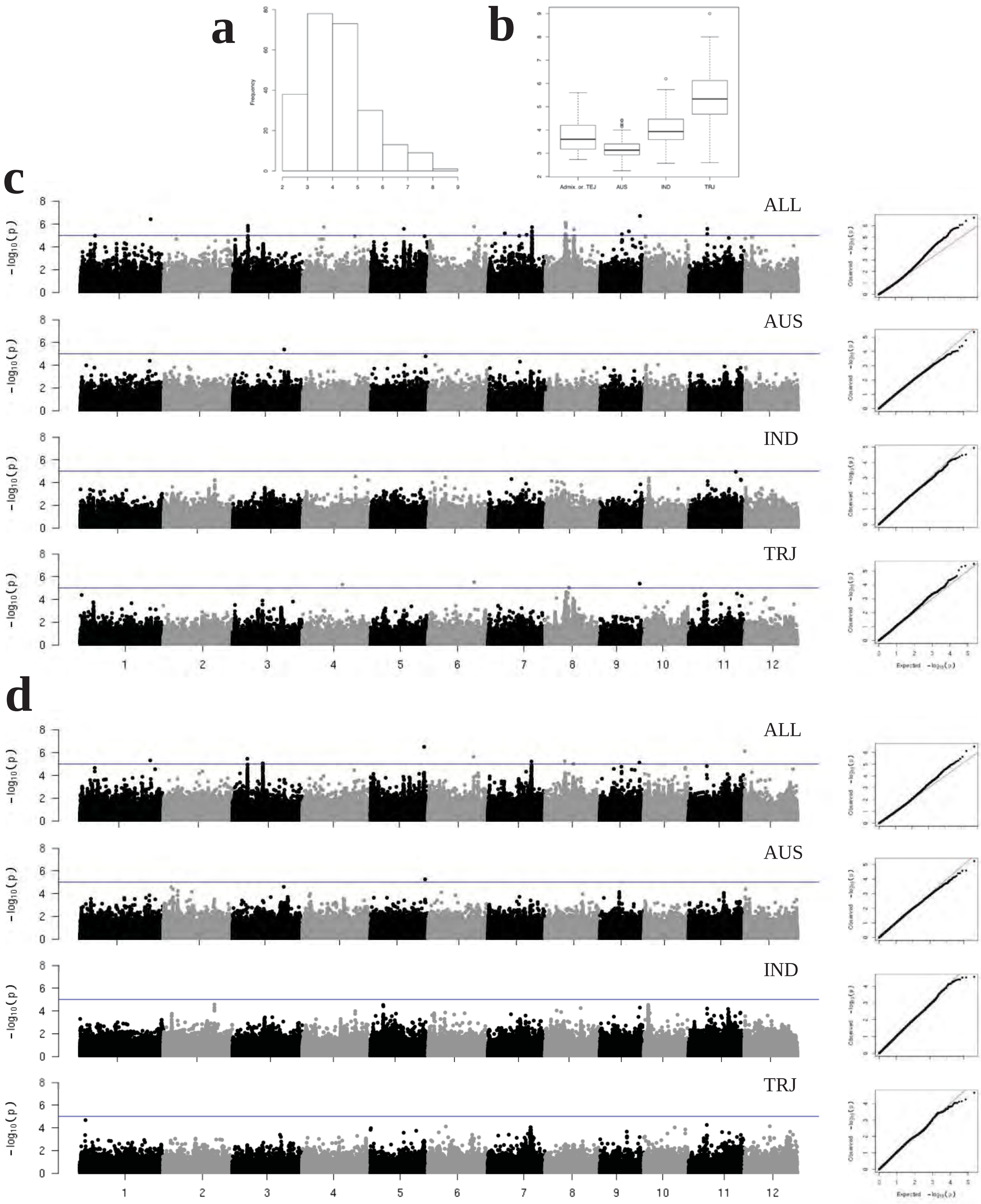

Supplementary Figure 36. Summary of GWAS results for Primary Branch Number in the Upper Half of the Panicle. (**a, b**) Phenotypic distributions. (**c**) Mixed model controlling for subpopulation structure across all varieties (ALL) and within individual subpopulations (AUS = *aus*, IND = *indica*, TRJ = *tropical japonica*). (**d**) Mixed model with heading date as a covariate.

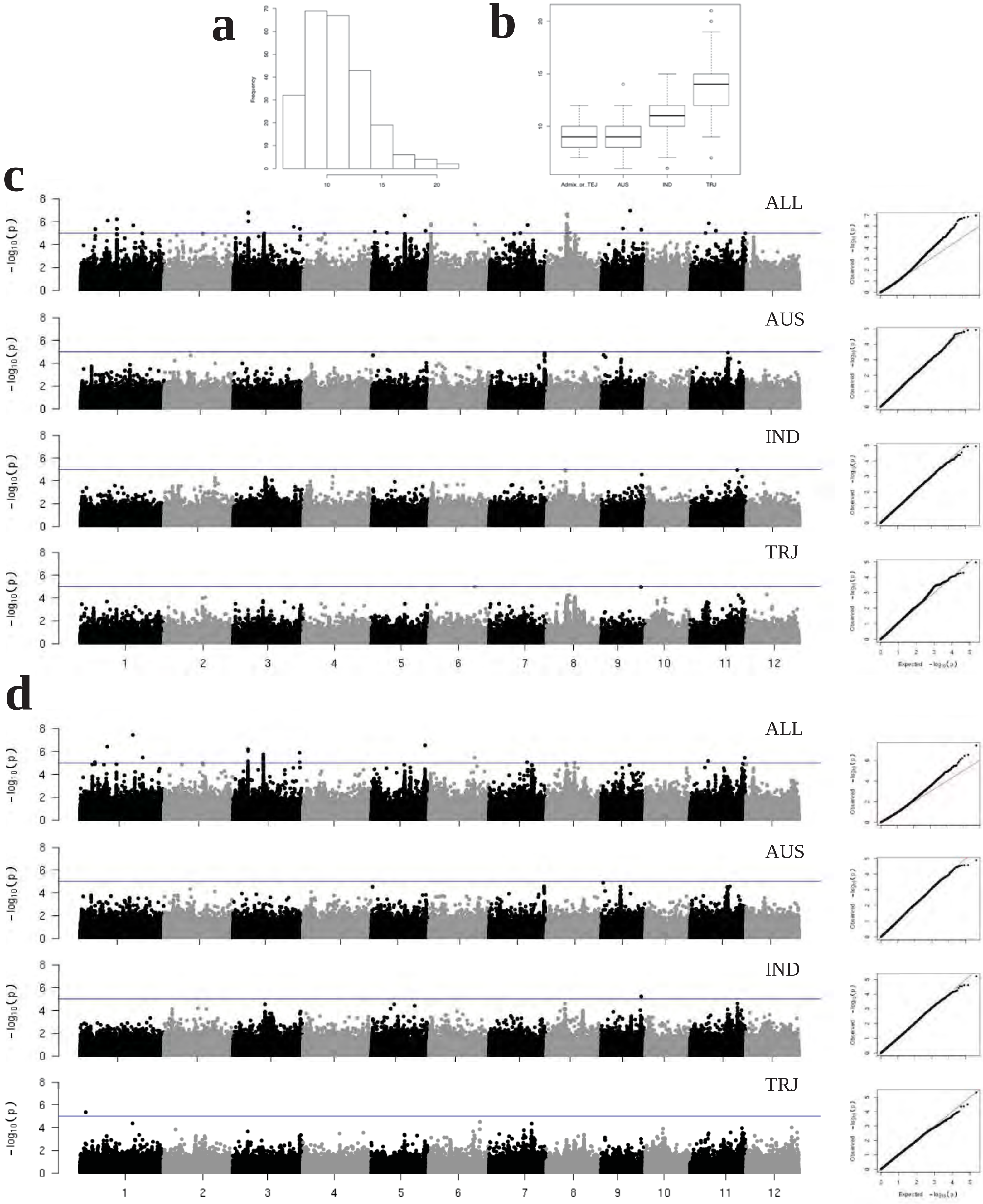

Supplementary Figure 37. Summary of GWAS results for Maximum Primary Branch Number. (**a, b**) Phenotypic distributions. (**c**) Mixed model controlling for subpopulation structure across all varieties (ALL) and within individual subpopulations (AUS = *aus*, IND = *indica*, TRJ = *tropical japonica*). (**d**) Mixed model with heading date as a covariate.

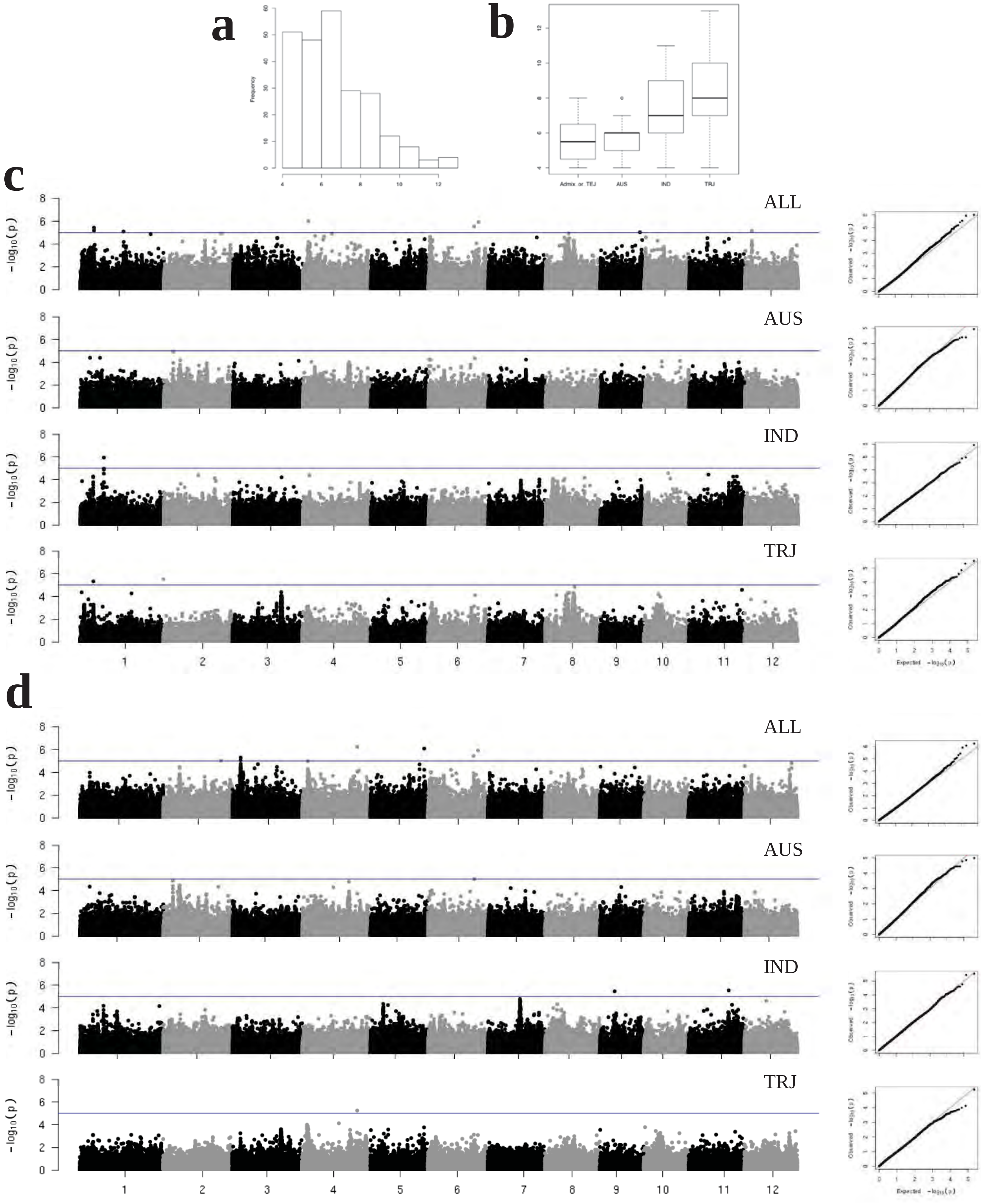

Supplementary Figure 38. Summary of GWAS results for Minimum Primary Branch Number. (**a, b**) Phenotypic distributions. (**c**) Mixed model controlling for subpopulation structure across all varieties (ALL) and within individual subpopulations (AUS = *aus*, IND = *indica*, TRJ = *tropical japonica*). (**d**) Mixed model with heading date as a covariate.

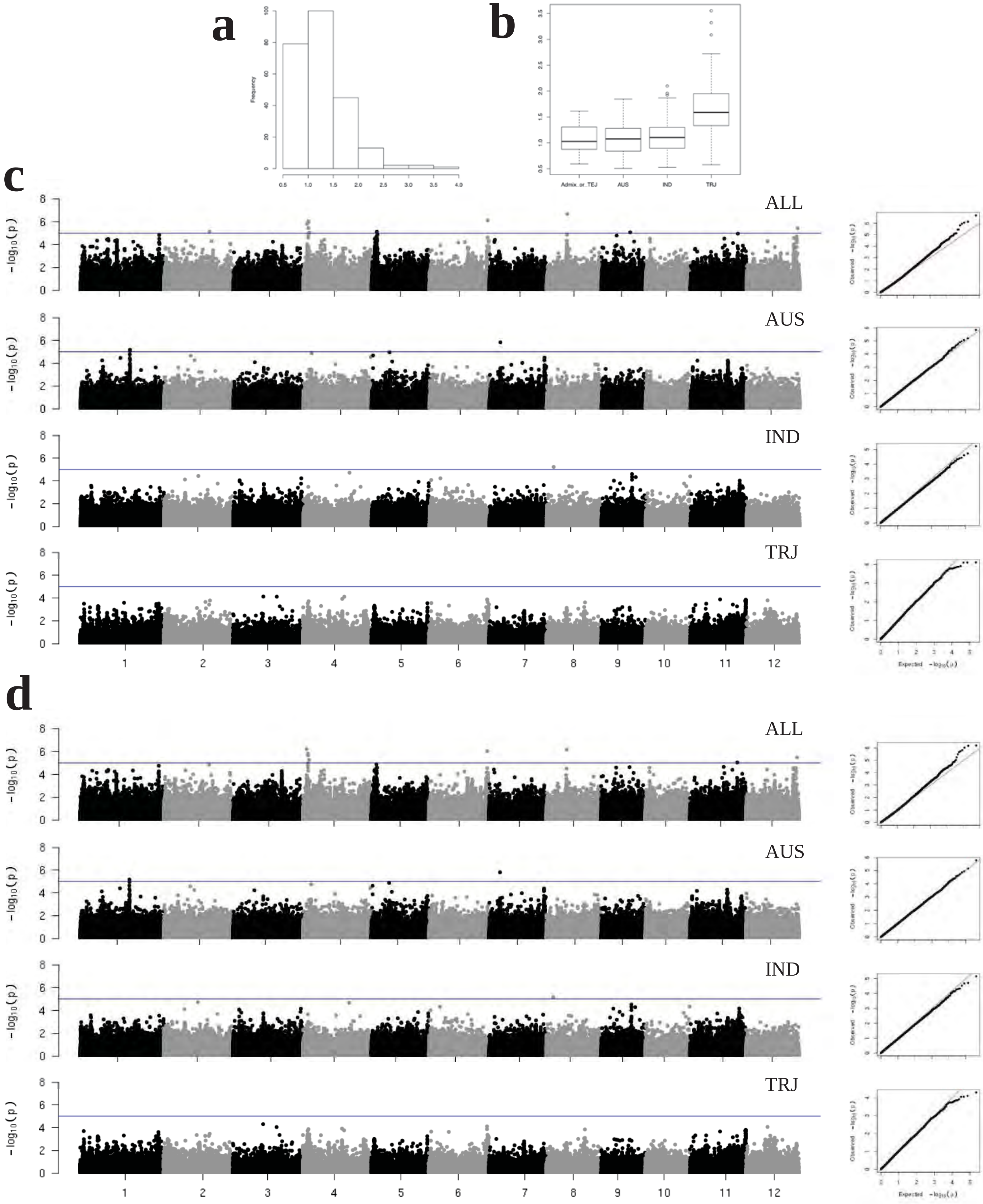

Supplementary Figure 39. Summary of GWAS results for Primary Branch Number Standard Deviation. (**a, b**) Phenotypic distributions. (**c**) Mixed model controlling for subpopulation structure across all varieties (ALL) and within individual subpopulations (AUS = *aus*, IND = *indica*, TRJ = *tropical japonica*). (**d**) Mixed model with heading date as a covariate.

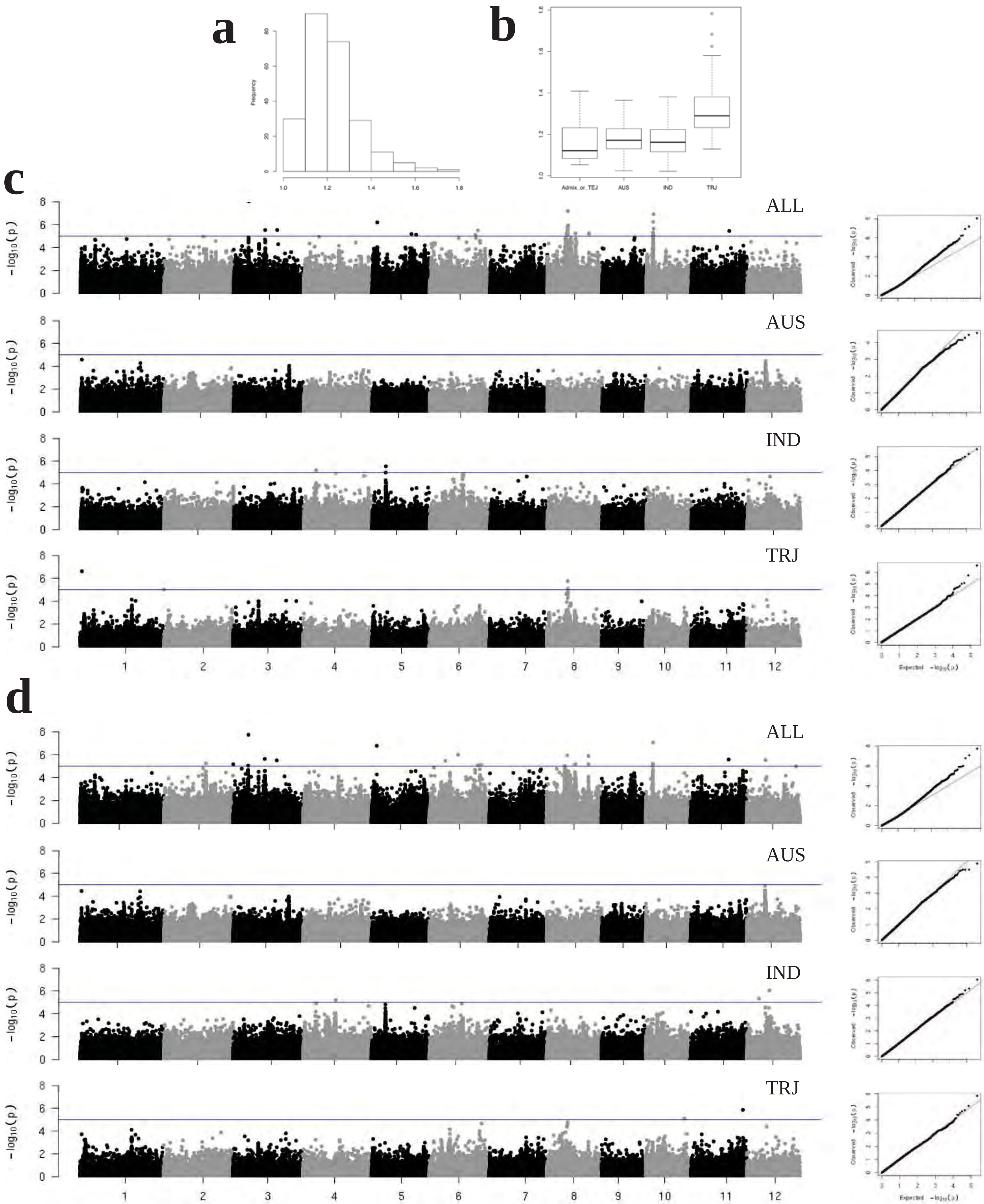

Supplementary Figure 40. Summary of GWAS results for Number of Primary Branches per Internode. (**a, b**) Phenotypic distributions. (**c**) Mixed model controlling for subpopulation structure across all varieties (ALL) and within individual subpopulations (AUS = *aus*, IND = *indica*, TRJ = *tropical japonica*). (**d**) Mixed model with heading date as a covariate.

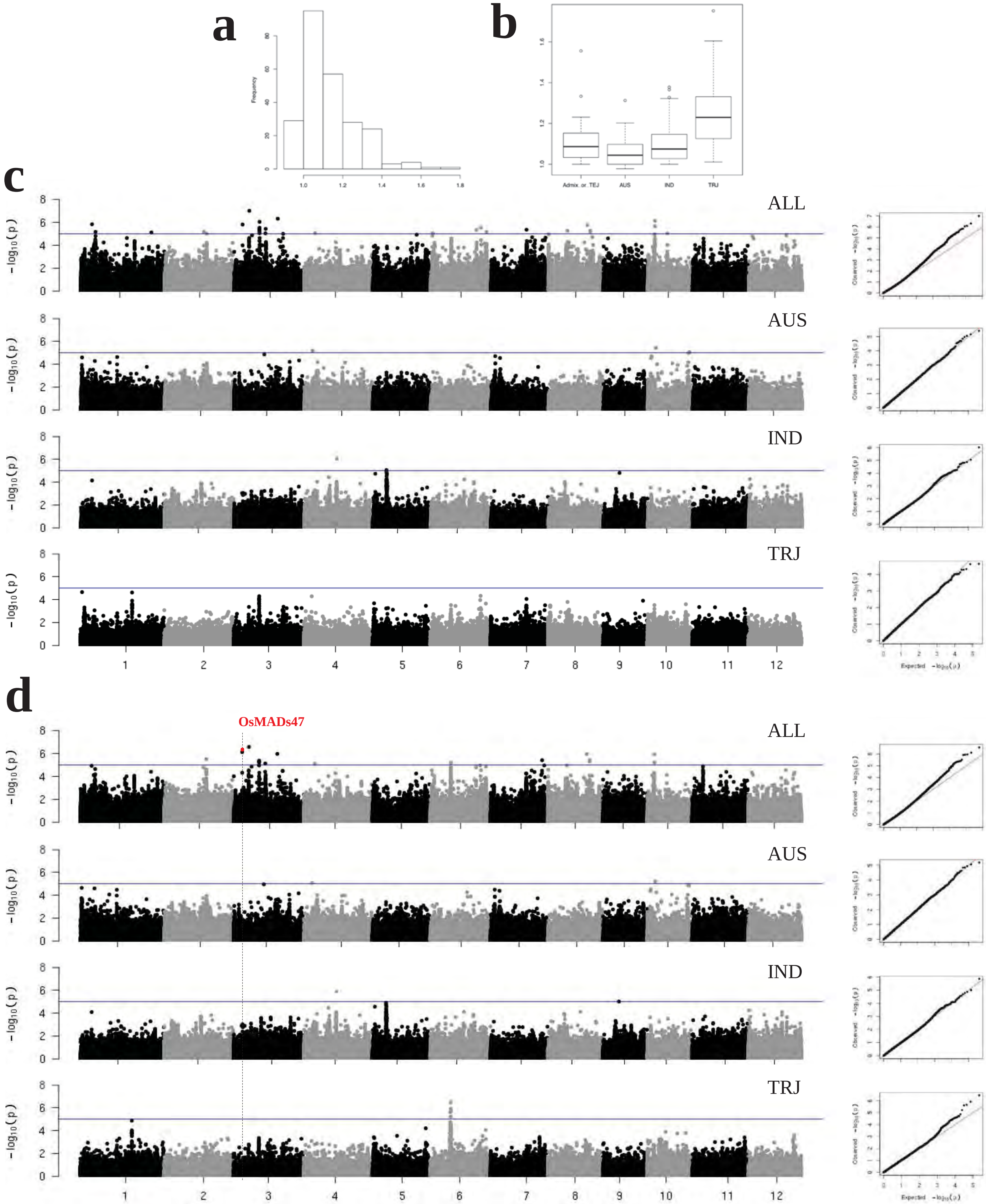

Supplementary Figure 41. Summary of GWAS results for Number of Primary Branches per Internode in the Upper Half of the Panicle. (**a**, **b**) Phenotypic distributions. (**c**) Mixed model controlling for subpopulation structure across all varieties (ALL) and within individual subpopulations (AUS = *aus*, IND = *indica*, TRJ = *tropical japonica*). (**d**) Mixed model with heading date as a covariate.

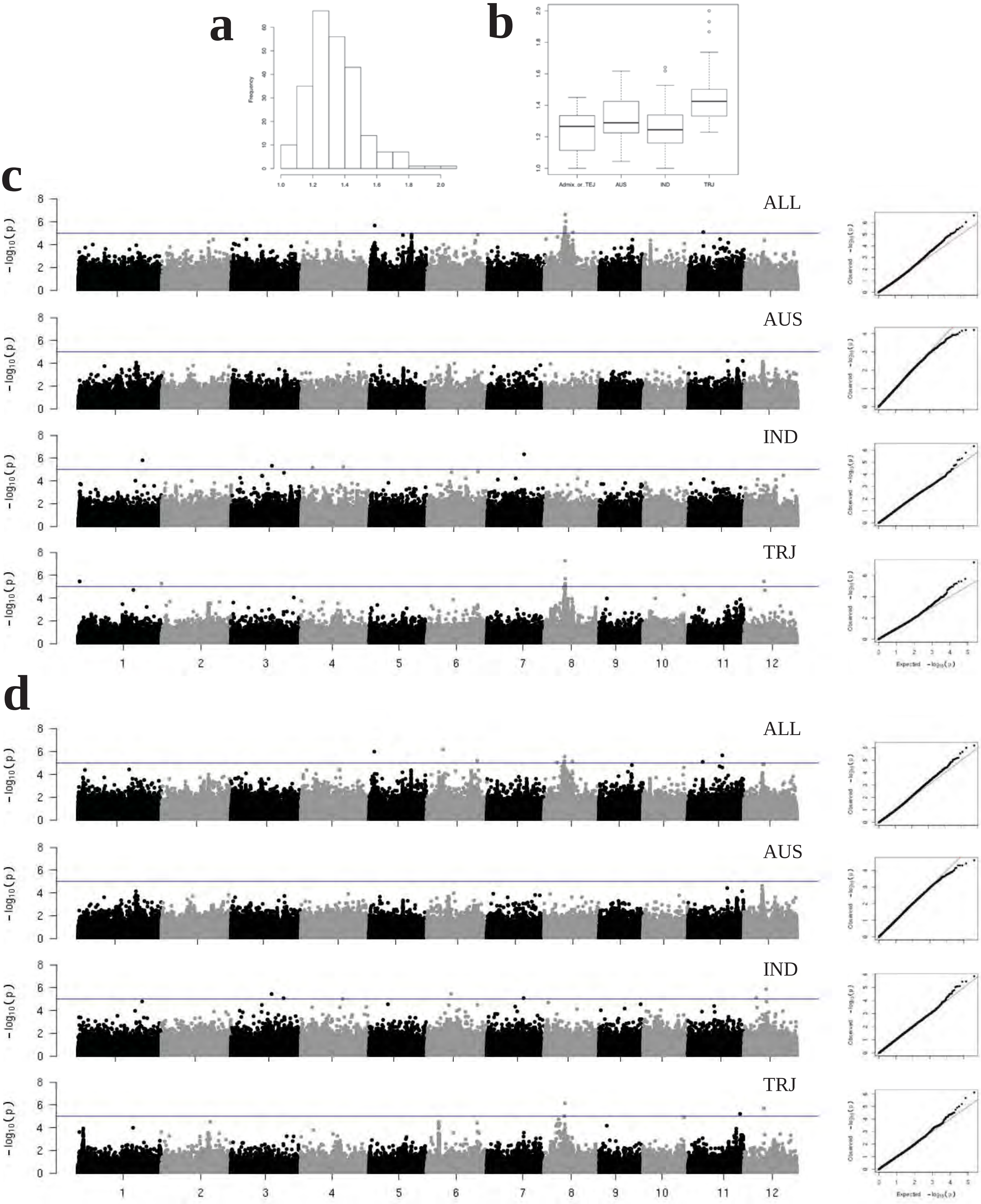

Supplementary Figure 42. Summary of GWAS results for Number of Primary Branches per Internode in the Lower Half of the Panicle. (**a, b**) Phenotypic distributions. (**c**) Mixed model controlling for subpopulation structure across all varieties (ALL) and within individual subpopulations (AUS = *aus*, IND = *indica*, TRJ = *tropical japonica*). (**d**) Mixed model with heading date as a covariate.

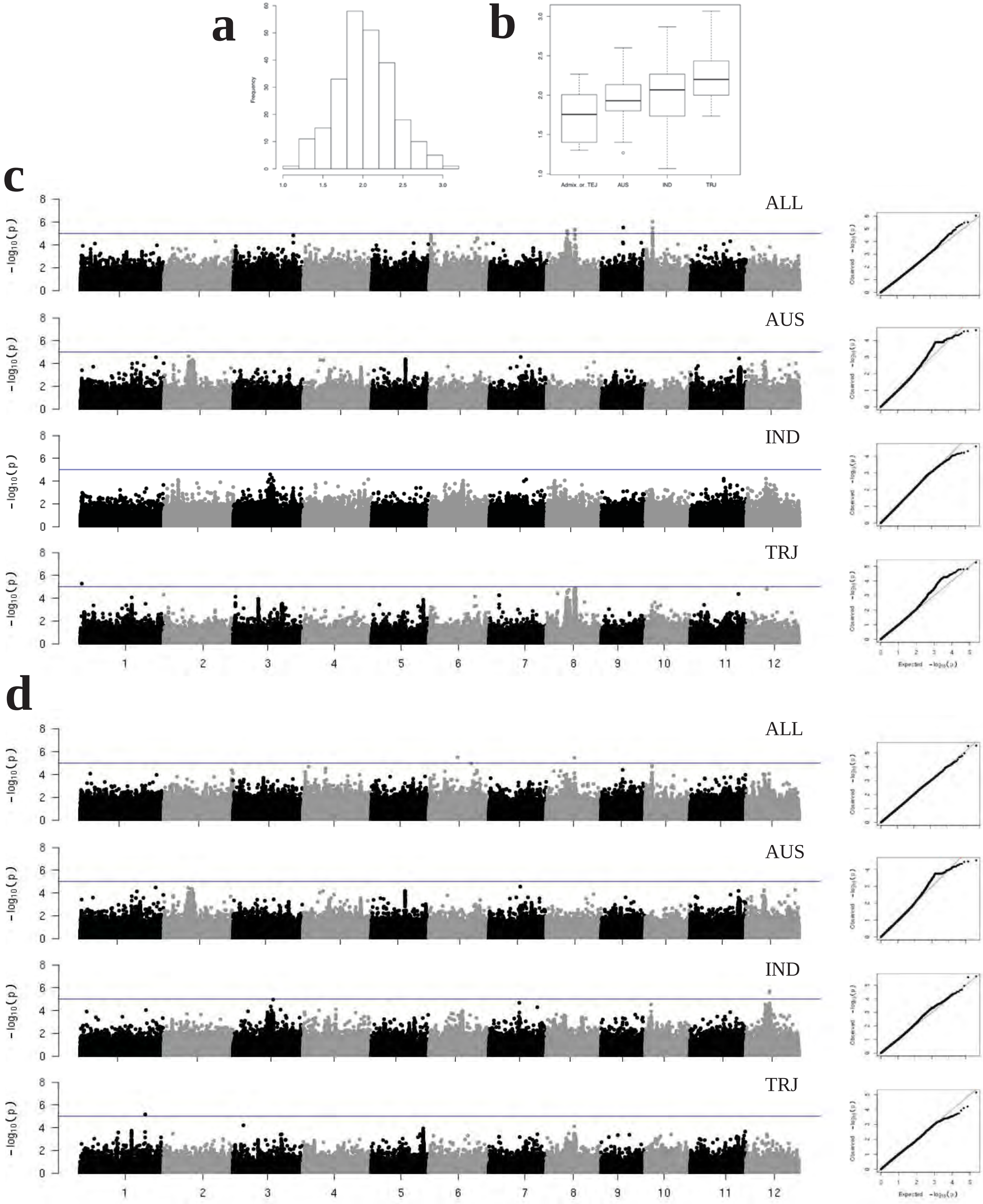

Supplementary Figure 43. Summary of GWAS results for Maximum Number of Primary Branches per Internode. (**a, b**) Phenotypic distributions. (**c**) Mixed model controlling for subpopulation structure across all varieties (ALL) and within individual subpopulations (AUS = *aus*, IND = *indica*, TRJ = *tropical japonica*). (**d**) Mixed model with heading date as a covariate.

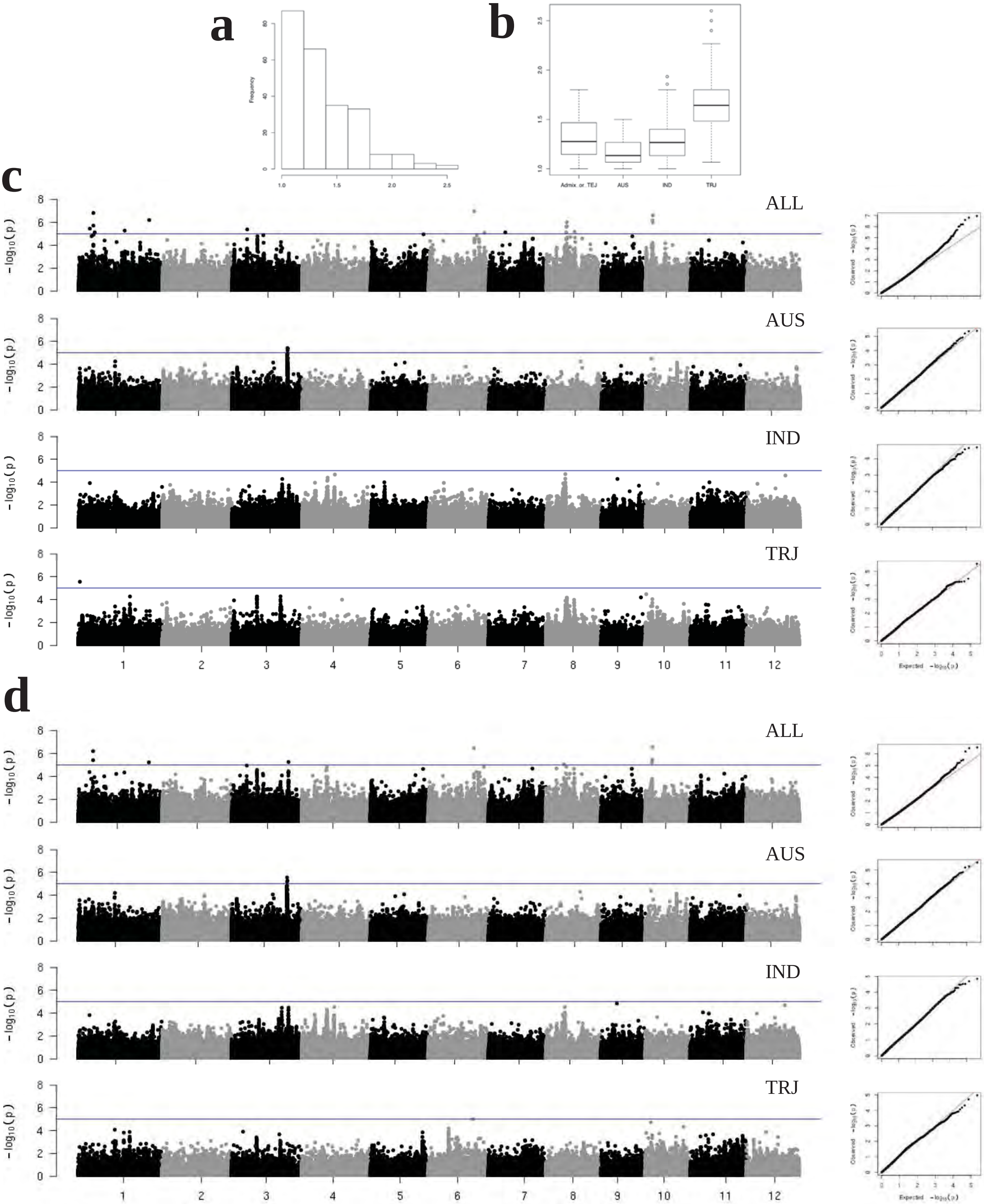

Supplementary Figure 44. Summary of GWAS results for Maximum Number of Primary Branches per Internode in the Upper Half of the Panicle. (**a, b**) Phenotypic distributions. (**c**) Mixed model controlling for subpopulation structure across all varieties (ALL) and within individual subpopulations (AUS = *aus*, IND = *indica*, TRJ = *tropical japonica*). (**d**) Mixed model with heading date as a covariate.

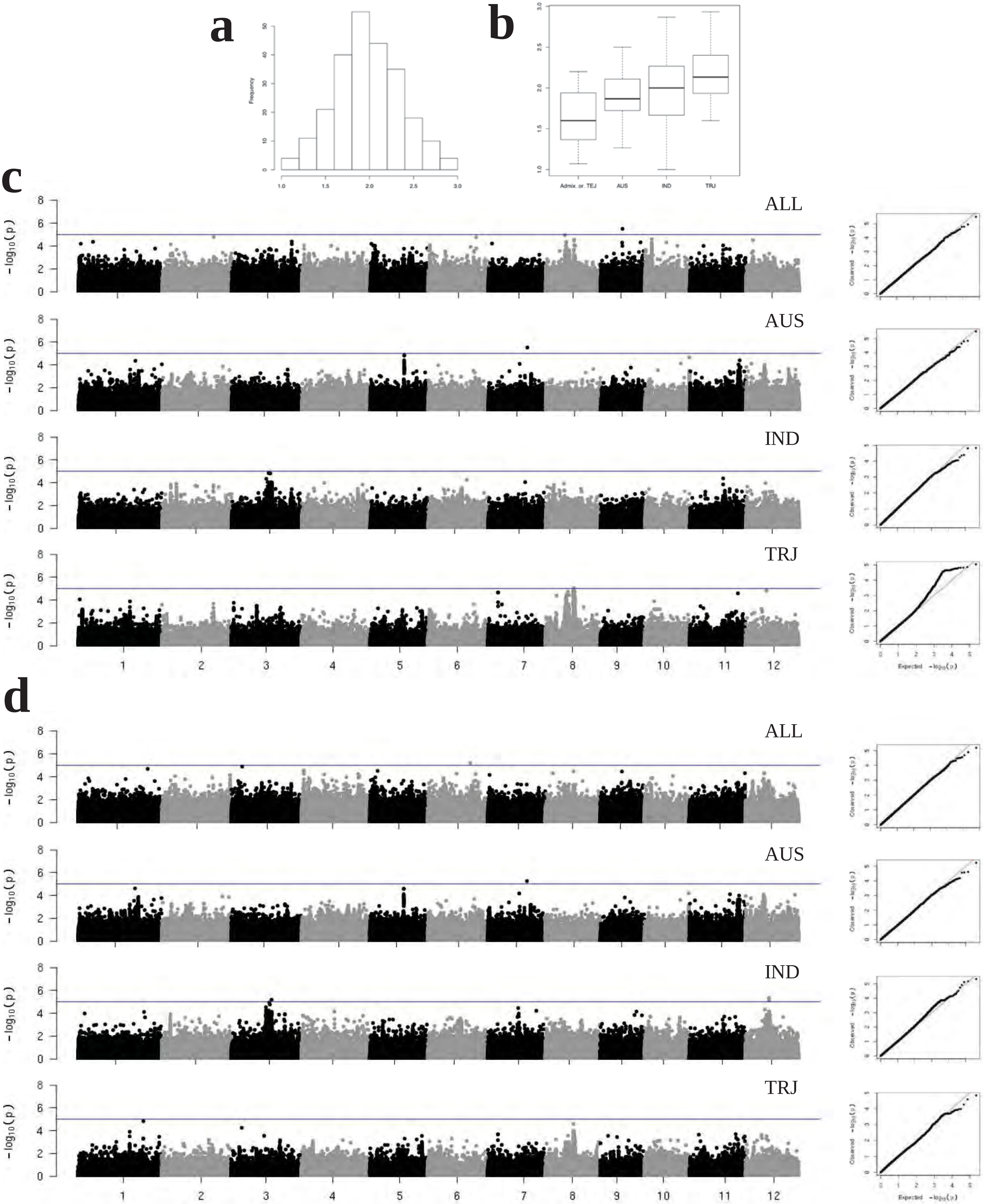

Supplementary Figure 45. Summary of GWAS results for Maximum Number of Primary Branches per Internode in the Lower Half of the Panicle. (**a, b**) Phenotypic distributions. (**c**) Mixed model controlling for subpopulation structure across all varieties (ALL) and within individual subpopulations (AUS = *aus*, IND = *indica*, TRJ = *tropical japonica*). (**d**) Mixed model with heading date as a covariate.

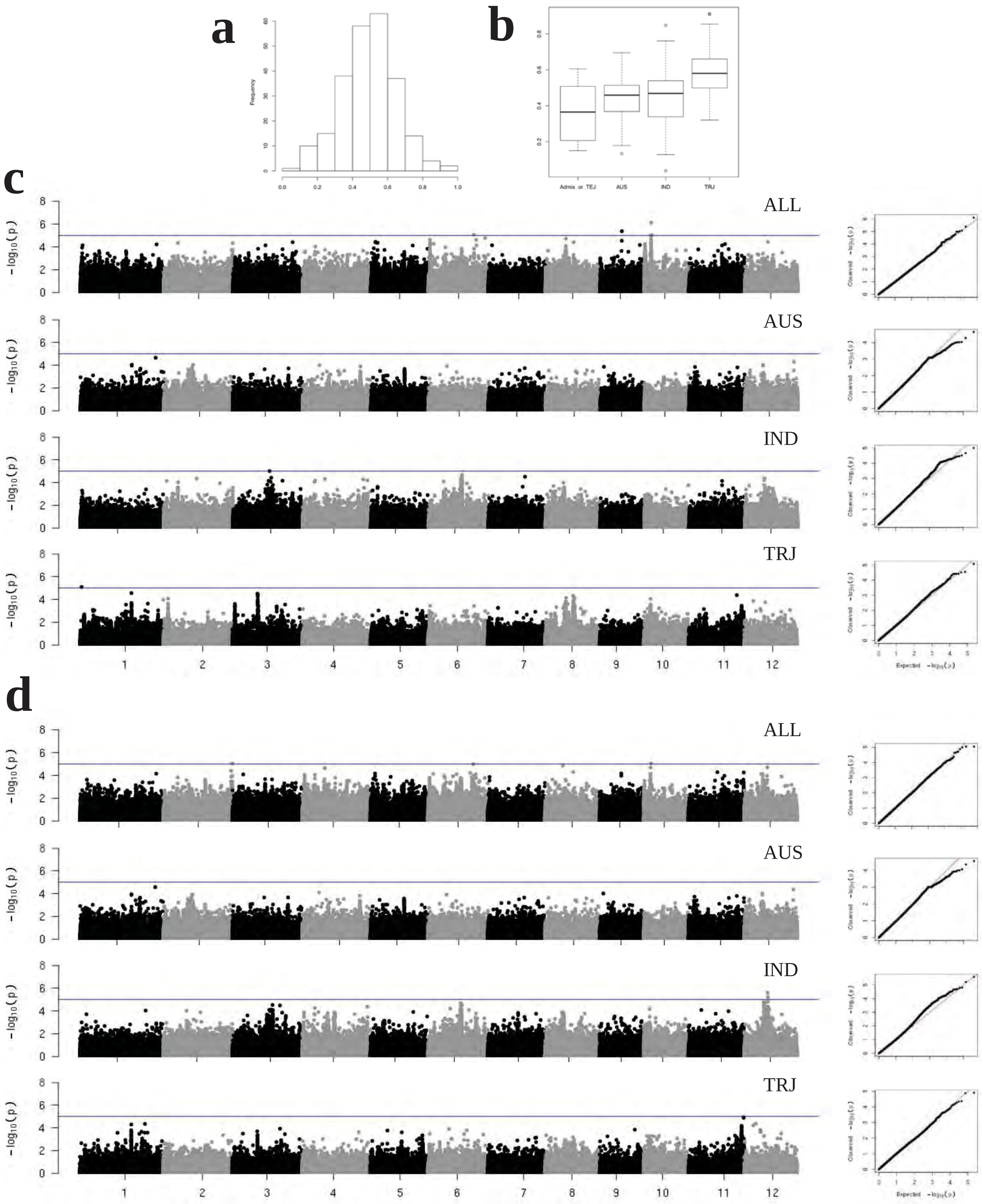

Supplementary Figure 46. Summary of GWAS results for Number of Primary Branches per Internode Standard Deviation. (**a, b**) Phenotypic distributions. (**c**) Mixed model controlling for subpopulation structure across all varieties (ALL) and within individual subpopulations (AUS = *aus*, IND = *indica*, TRJ = *tropical japonica*). (**d**) Mixed model with heading date as a covariate.

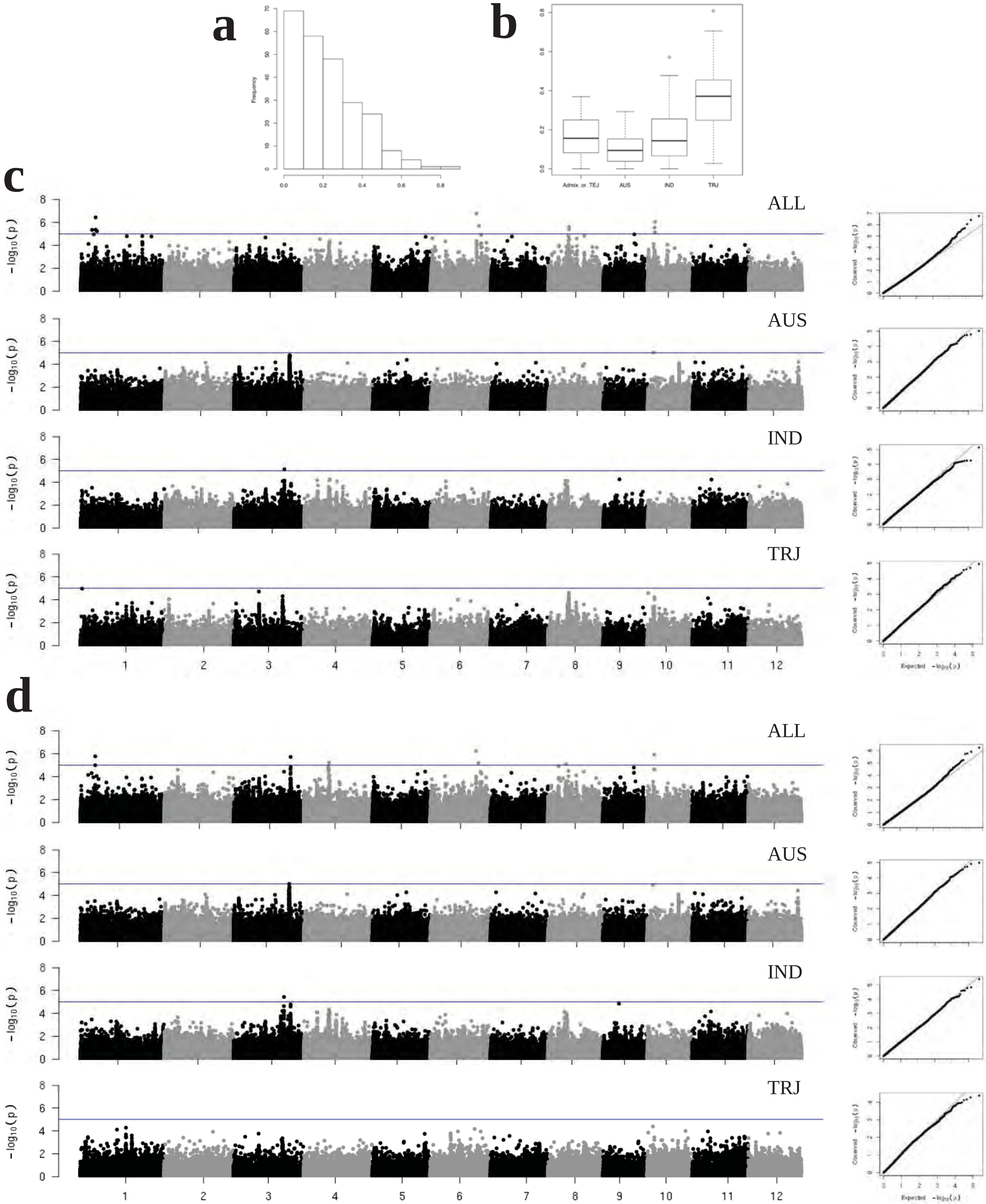

Supplementary Figure 47. Summary of GWAS results for Number of Primary Branches per Internode in the Upper Half of the Panicle Standard Deviation. (**a, b**) Phenotypic distributions. (**c**) Mixed model controlling for subpopulation structure across all varieties (ALL) and within individual subpopulations (AUS = *aus*, IND = *indica*, TRJ = *tropical japonica*). (**d**) Mixed model with heading date as a covariate.

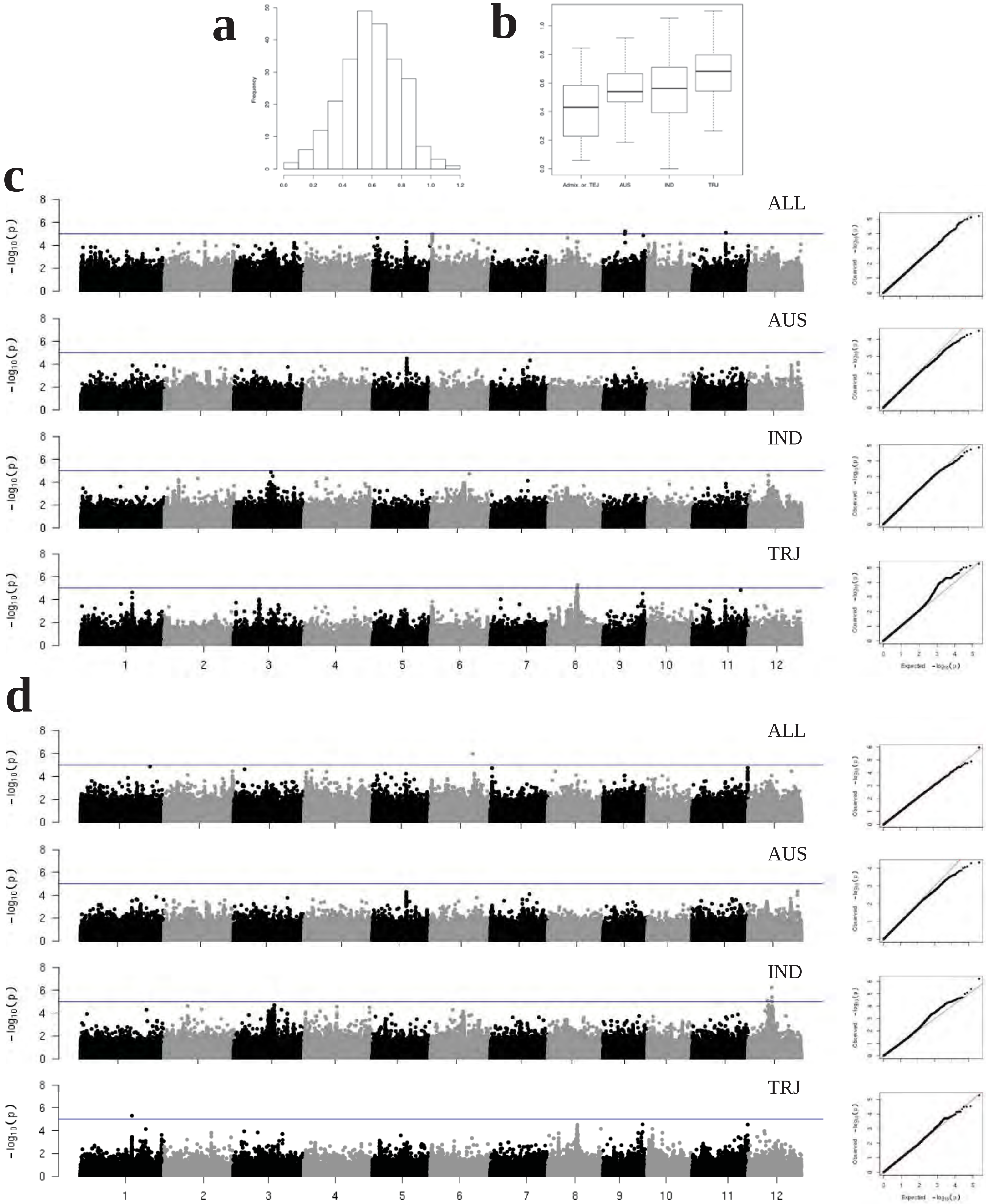

Supplementary Figure 48. Summary of GWAS results for Number of Primary Branches per Internode in the Lower Half of the Panicle Standard Deviation. (**a, b**) Phenotypic distributions. (**c**) Mixed model controlling for subpopulation structure across all varieties (ALL) and within individual subpopulations (AUS = *aus*, IND = *indica*, TRJ = *tropical japonica*). (**d**) Mixed model with heading date as a covariate.

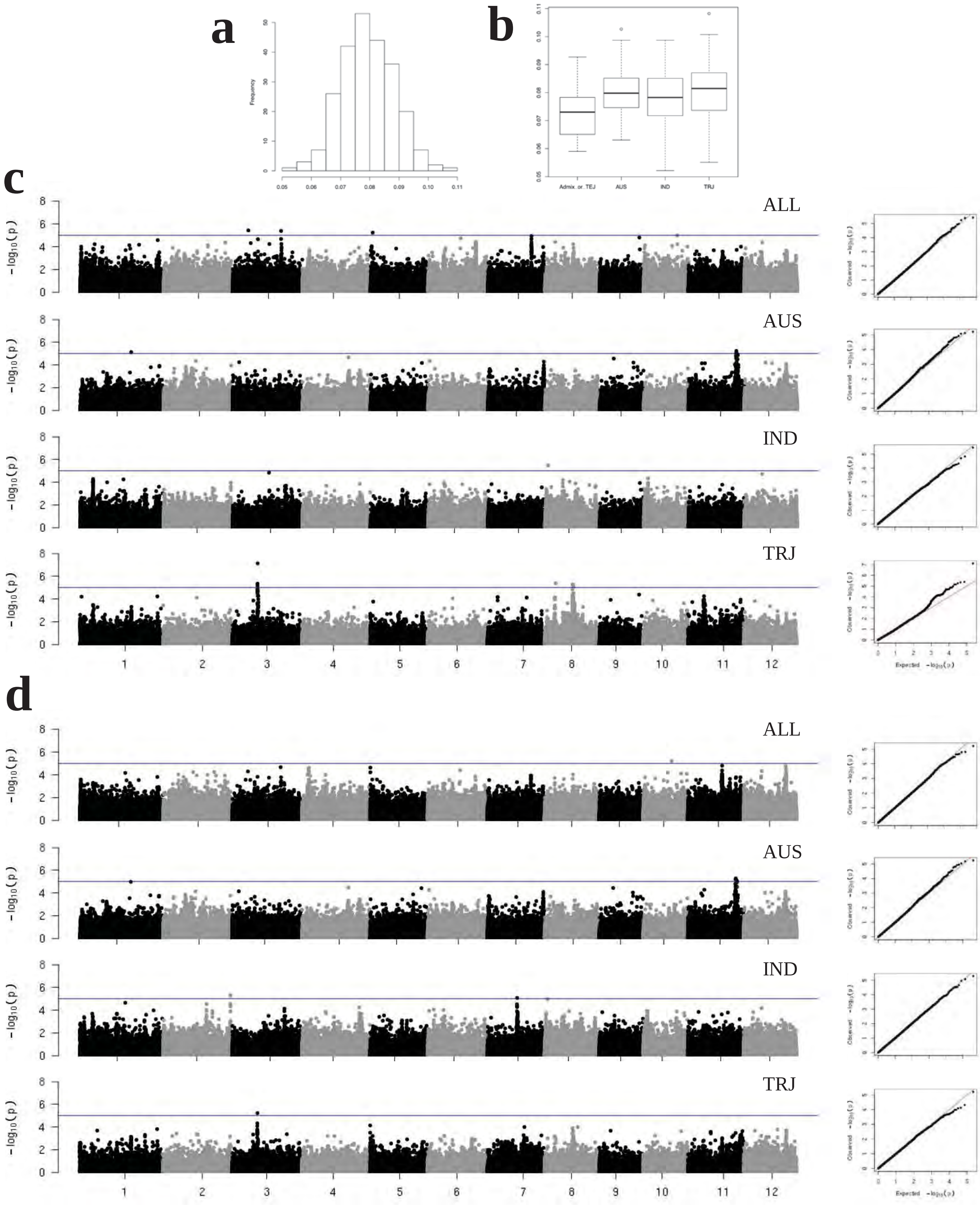

Supplementary Figure 49. Summary of GWAS results for Thickness of the Rachis (cm). (**a, b**) Phenotypic distributions. (**c**) Mixed model controlling for subpopulation structure across all varieties (ALL) and within individual subpopulations (AUS = *aus*, IND = *indica*, TRJ = *tropical japonica*). (**d**) Mixed model with heading date as a covariate.

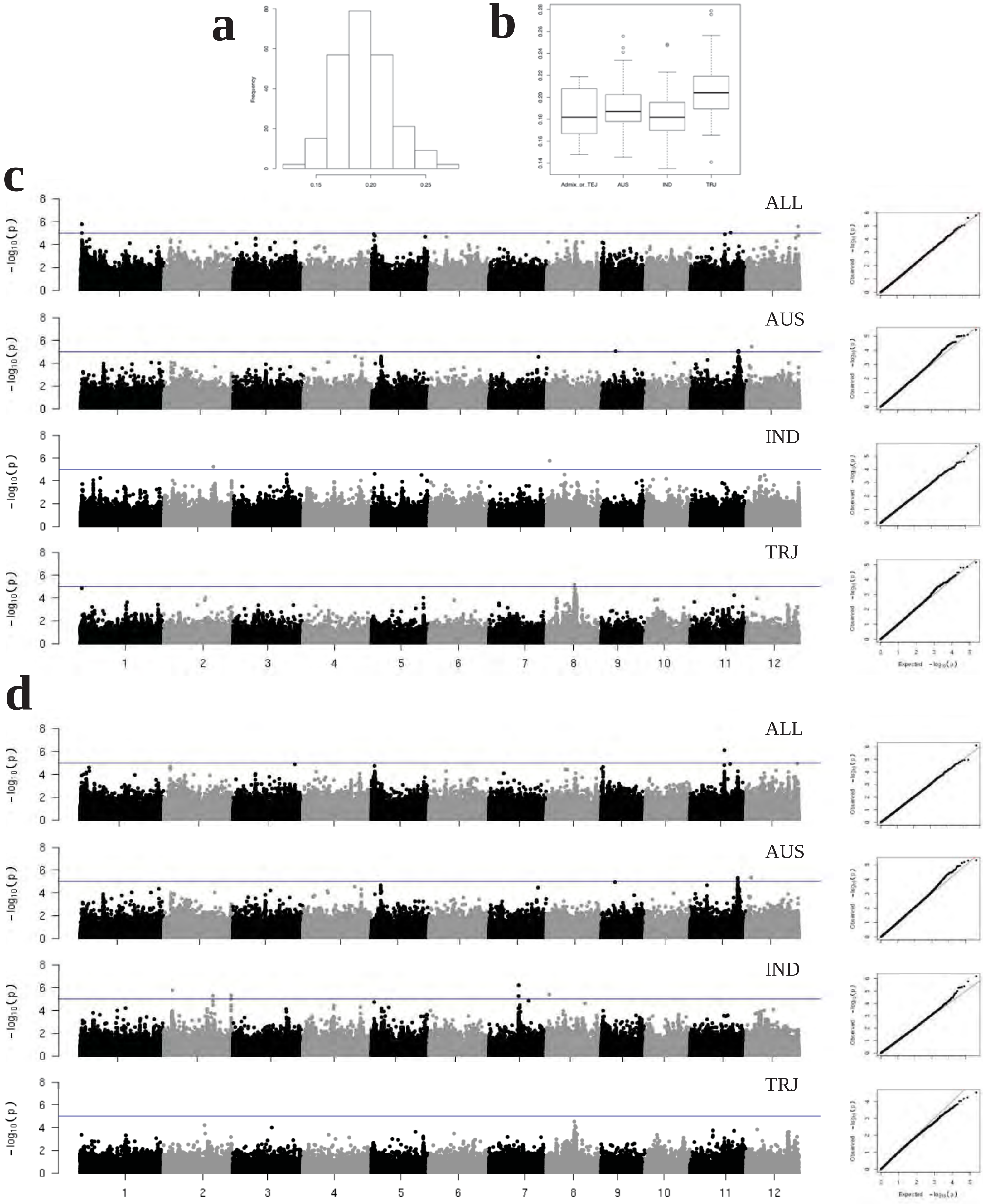

Supplementary Figure 50. Summary of GWAS results for Maximum Thickness of the Rachis (cm). (**a, b**) Phenotypic distributions. (**c**) Mixed model controlling for subpopulation structure across all varieties (ALL) and within individual subpopulations (AUS = *aus*, IND = *indica*, TRJ = *tropical japonica*). (**d**) Mixed model with heading date as a covariate.

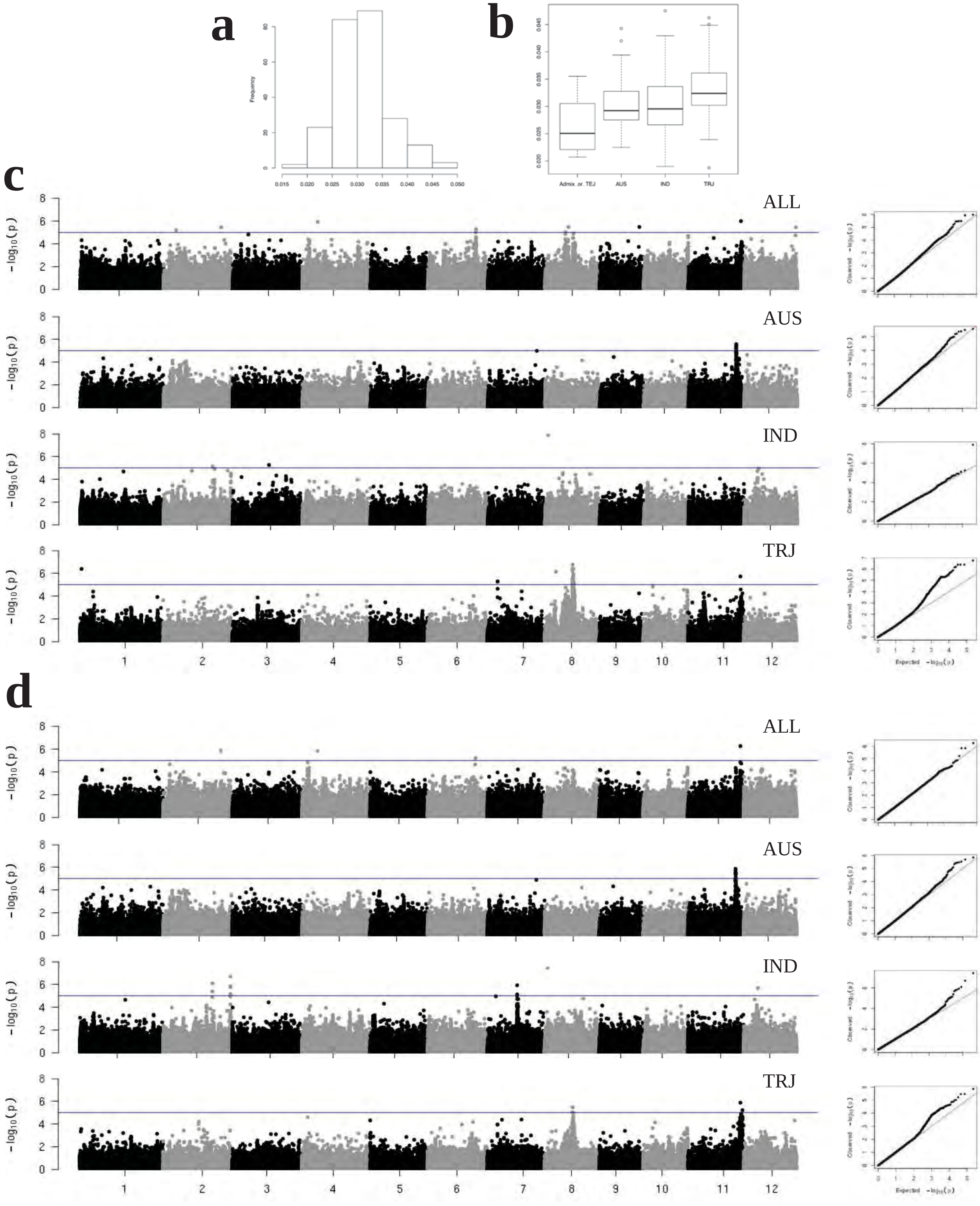

Supplementary Figure 51. Summary of GWAS results for Thickness of the Rachis Standard Deviation (cm). (**a, b**) Phenotypic distributions. (**c**) Mixed model controlling for subpopulation structure across all varieties (ALL) and within individual subpopulations (AUS = *aus*, IND = *indica*, TRJ = *tropical japonica*). (**d**) Mixed model with heading date as a covariate.

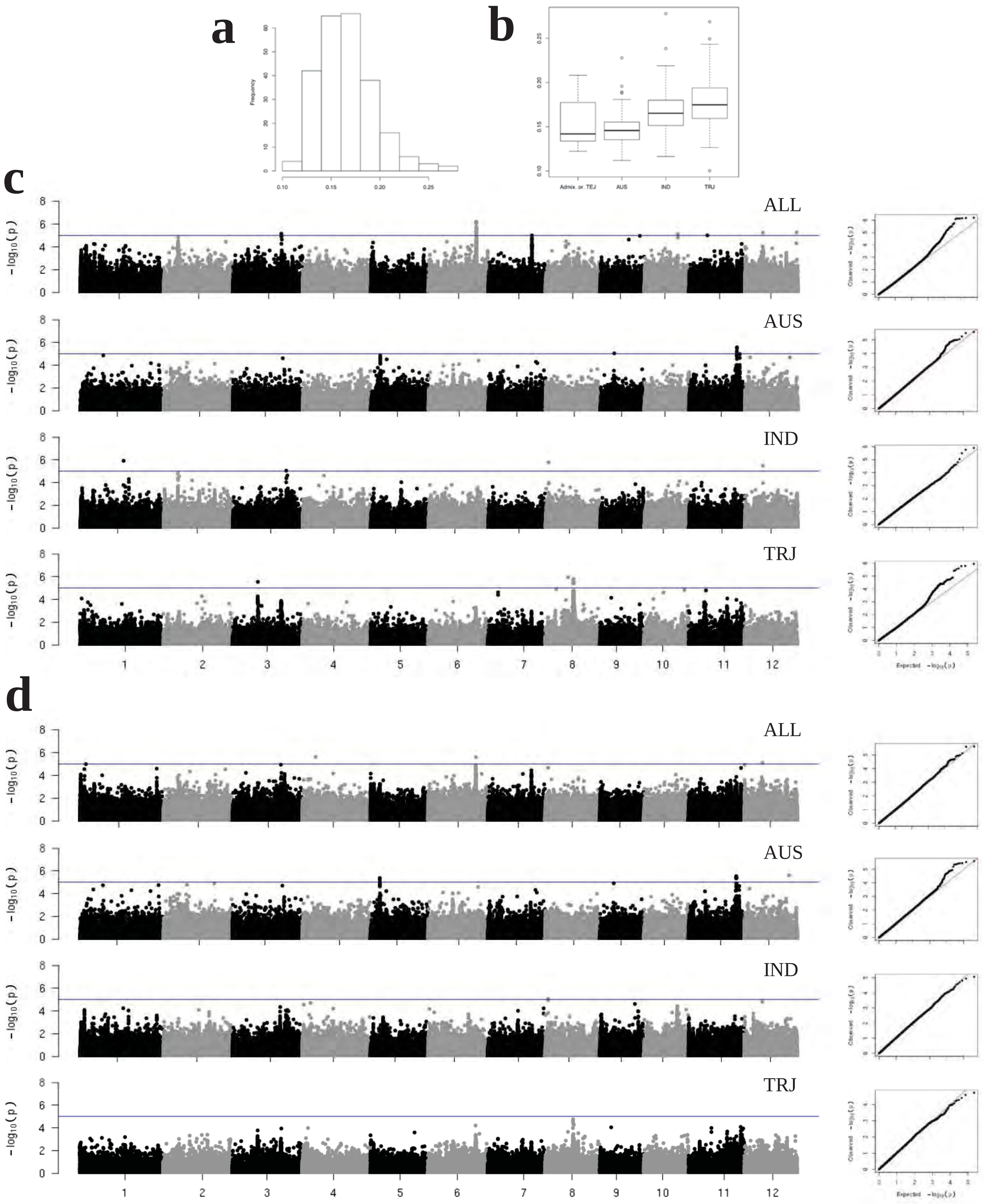

Supplementary Figure 52. Summary of GWAS results for Thickness of Exsertion (cm). (**a, b**) Phenotypic distributions. (**c**) Mixed model controlling for subpopulation structure across all varieties (ALL) and within individual subpopulations (AUS = *aus*, IND = *indica*, TRJ = *tropical japonica*). (**d**) Mixed model with heading date as a covariate.

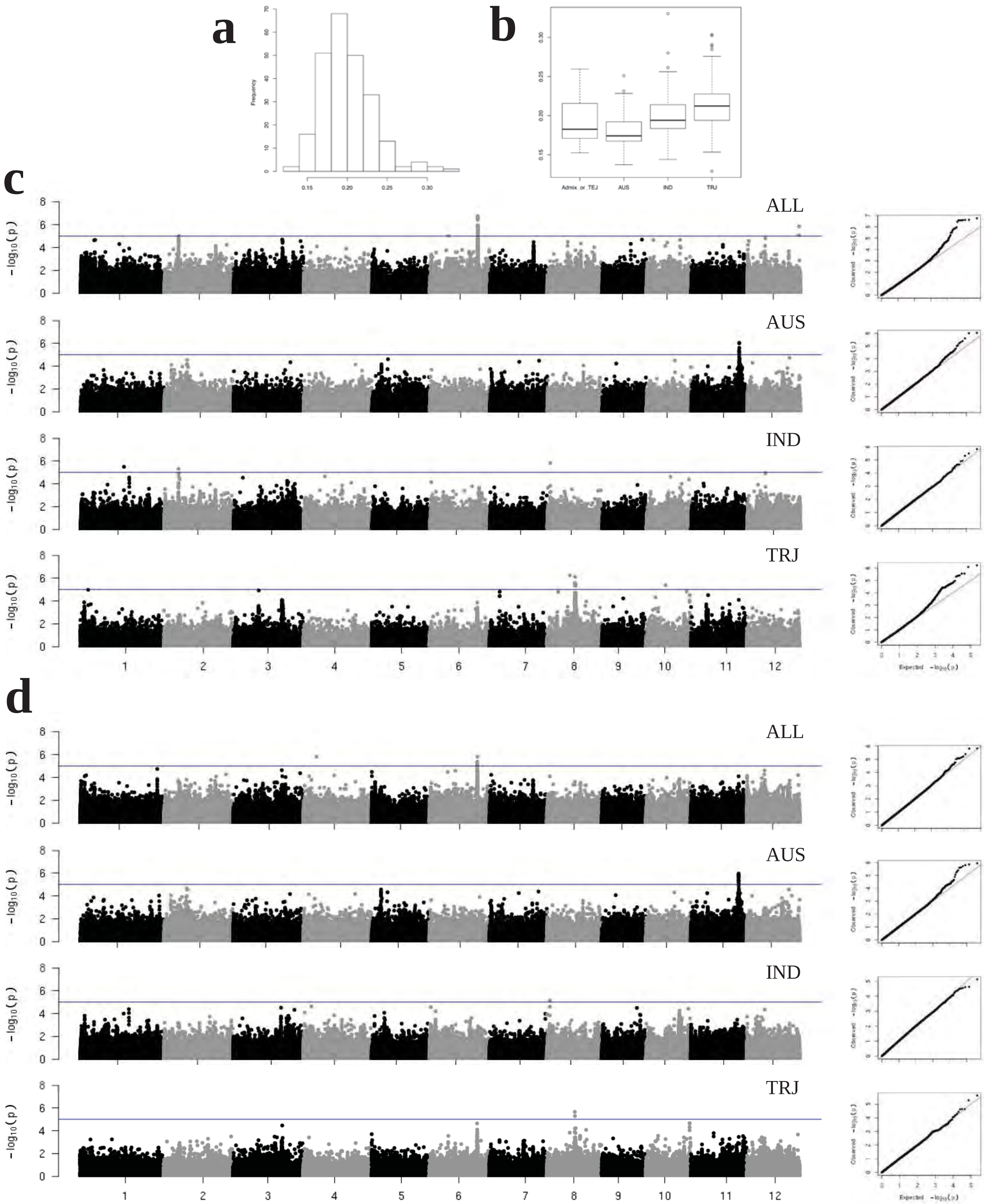

Supplementary Figure 53. Summary of GWAS results for Maximum Thickness of Exsertion (cm). (**a, b**) Phenotypic distributions. (**c**) Mixed model controlling for subpopulation structure across all varieties (ALL) and within individual subpopulations (AUS = *aus*, IND = *indica*, TRJ = *tropical japonica*). (**d**) Mixed model with heading date as a covariate.

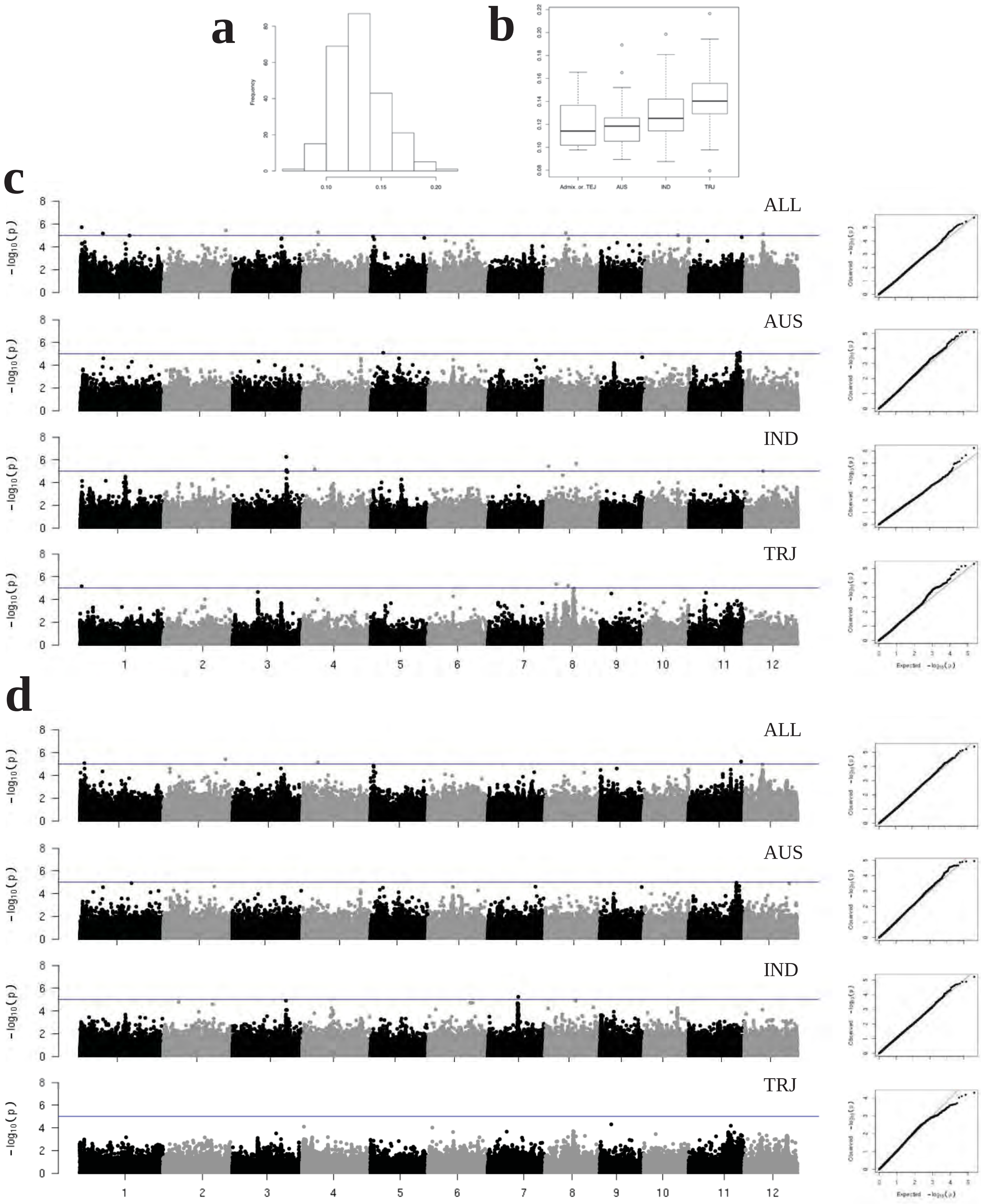

Supplementary Figure 54. Summary of GWAS results for Minimum Thickness of Exsertion (cm). (**a, b**) Phenotypic distributions. (**c**) Mixed model controlling for subpopulation structure across all varieties (ALL) and within individual subpopulations (AUS = *aus*, IND = *indica*, TRJ = *tropical japonica*). (**d**) Mixed model with heading date as a covariate.

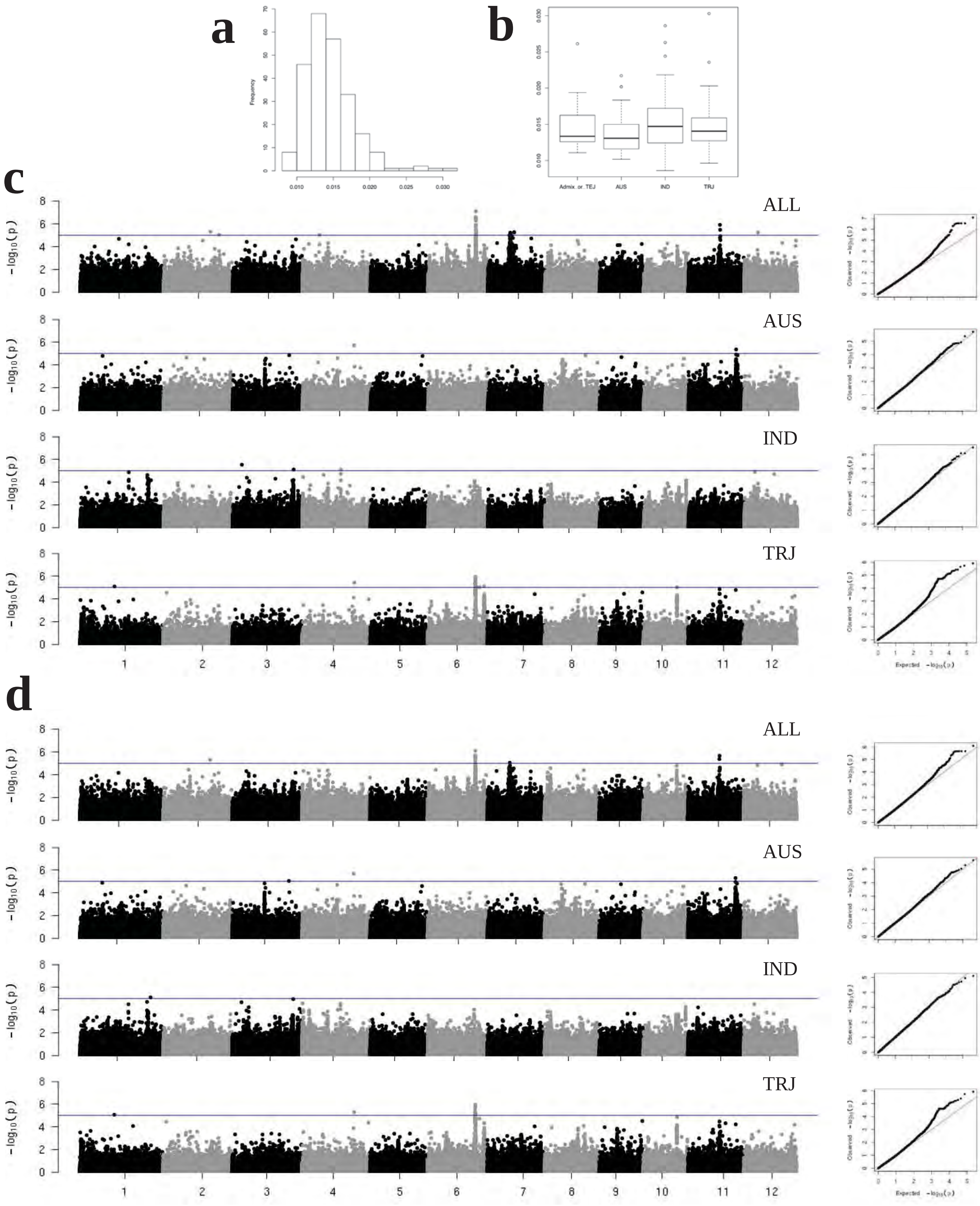

Supplementary Figure 55. Summary of GWAS results for Thickness of Exsertion Standard Deviation (cm). (**a, b**) Phenotypic distributions. (**c**) Mixed model controlling for subpopulation structure across all varieties (ALL) and within individual subpopulations (AUS = *aus*, IND = *indica*, TRJ = *tropical japonica*). (**d**) Mixed model with heading date as a covariate.

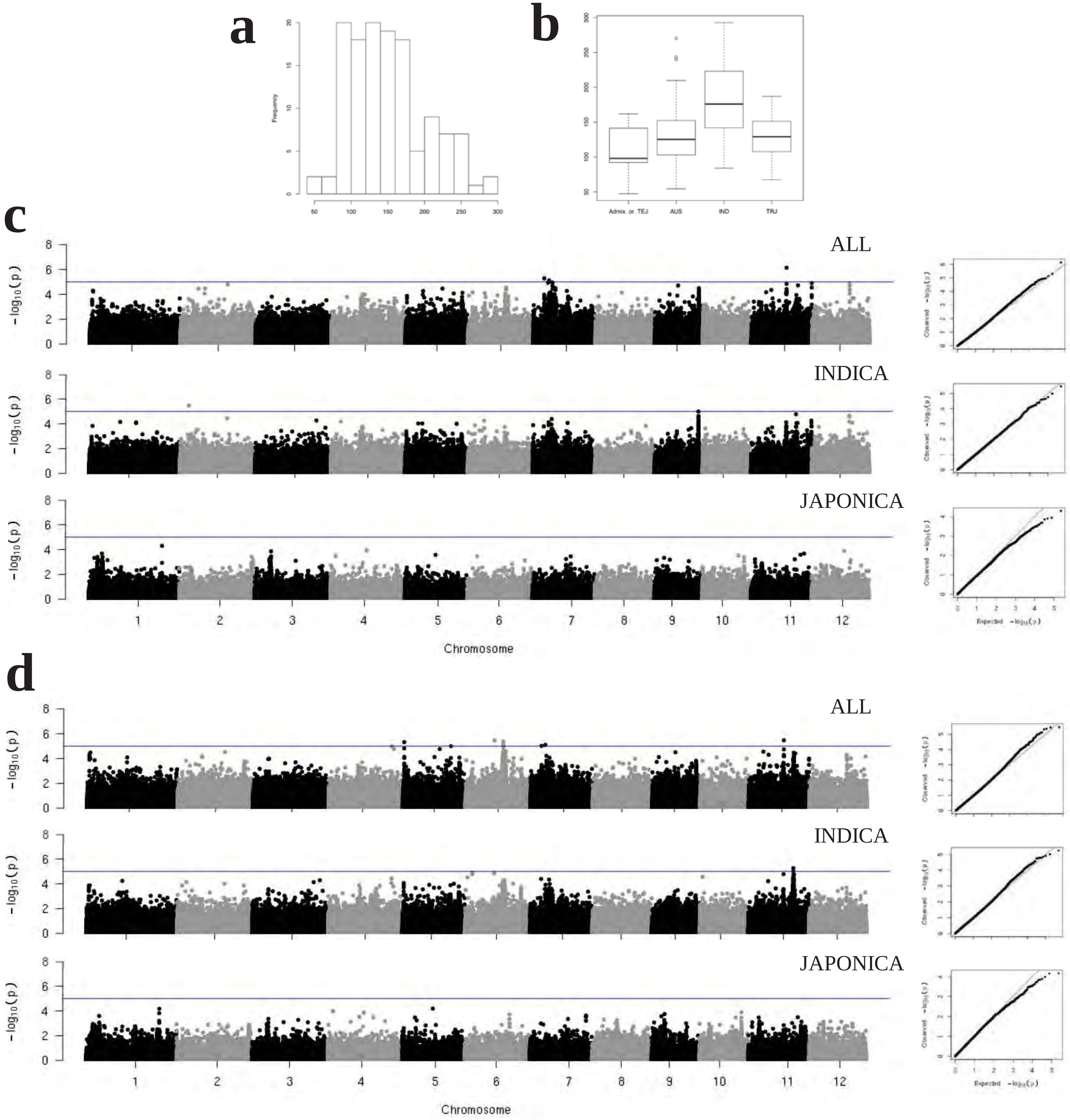

Supplementary Figure 56. Summary of GWAS results for Panicle Weight (g). (**a, b**) Phenotypic distributions. (**c**) Mixed model controlling for subpopulation structure across all varieties (ALL) and within the two major varietal groups *Indica* (INDICA) and *Japonica* (JAPONICA). (**d**) Mixed model with heading date as a covariate.

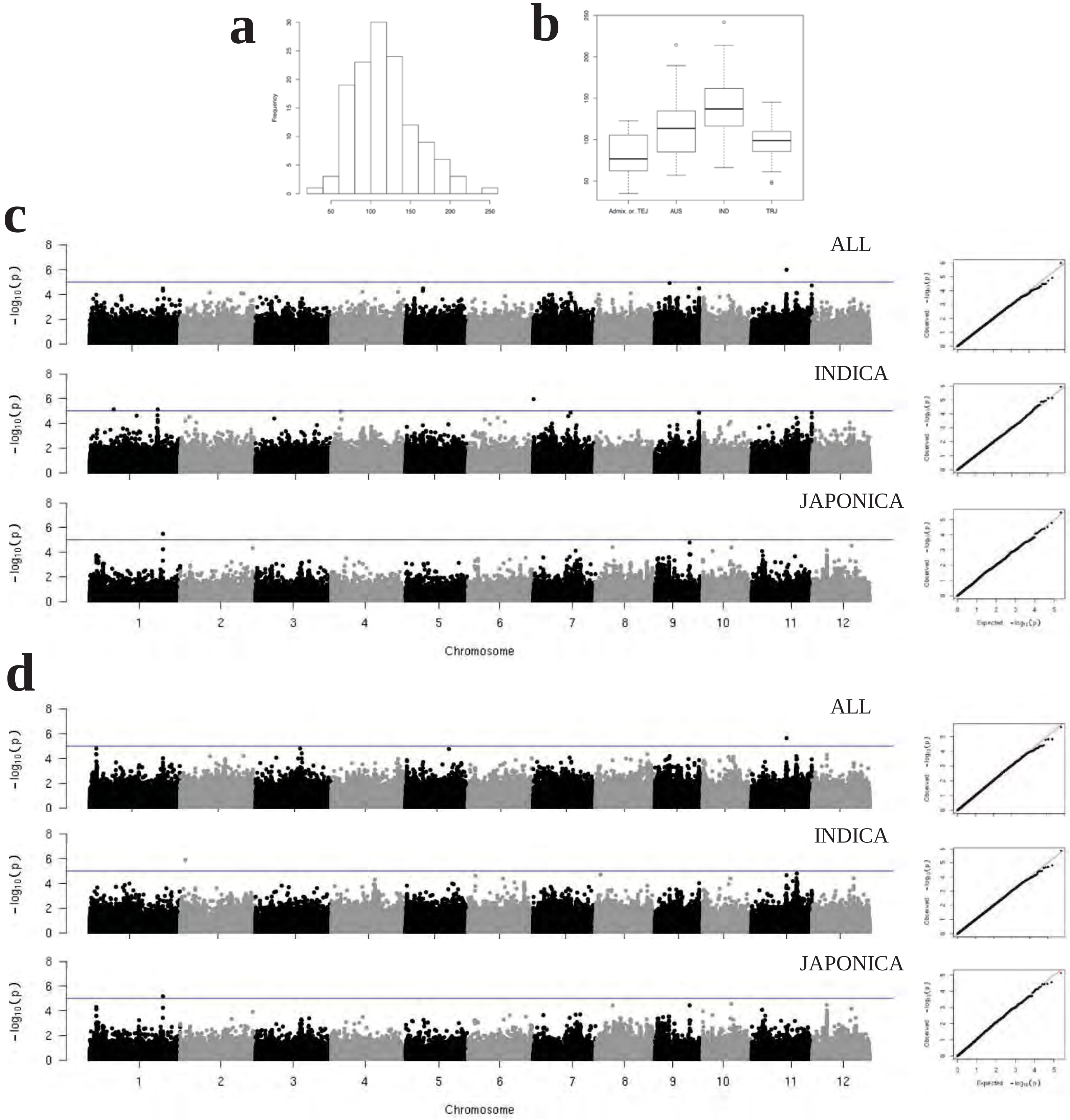

Supplementary Figure 57. Summary of GWAS results for Total Grain Weight (g). (**a, b**) Phenotypic distributions. (**c**) Mixed model controlling for subpopulation structure across all varieties (ALL) and within the two major varietal groups *Indica* (INDICA) and *Japonica* (JAPONICA). (**d**) Mixed model with heading date as a covariate.

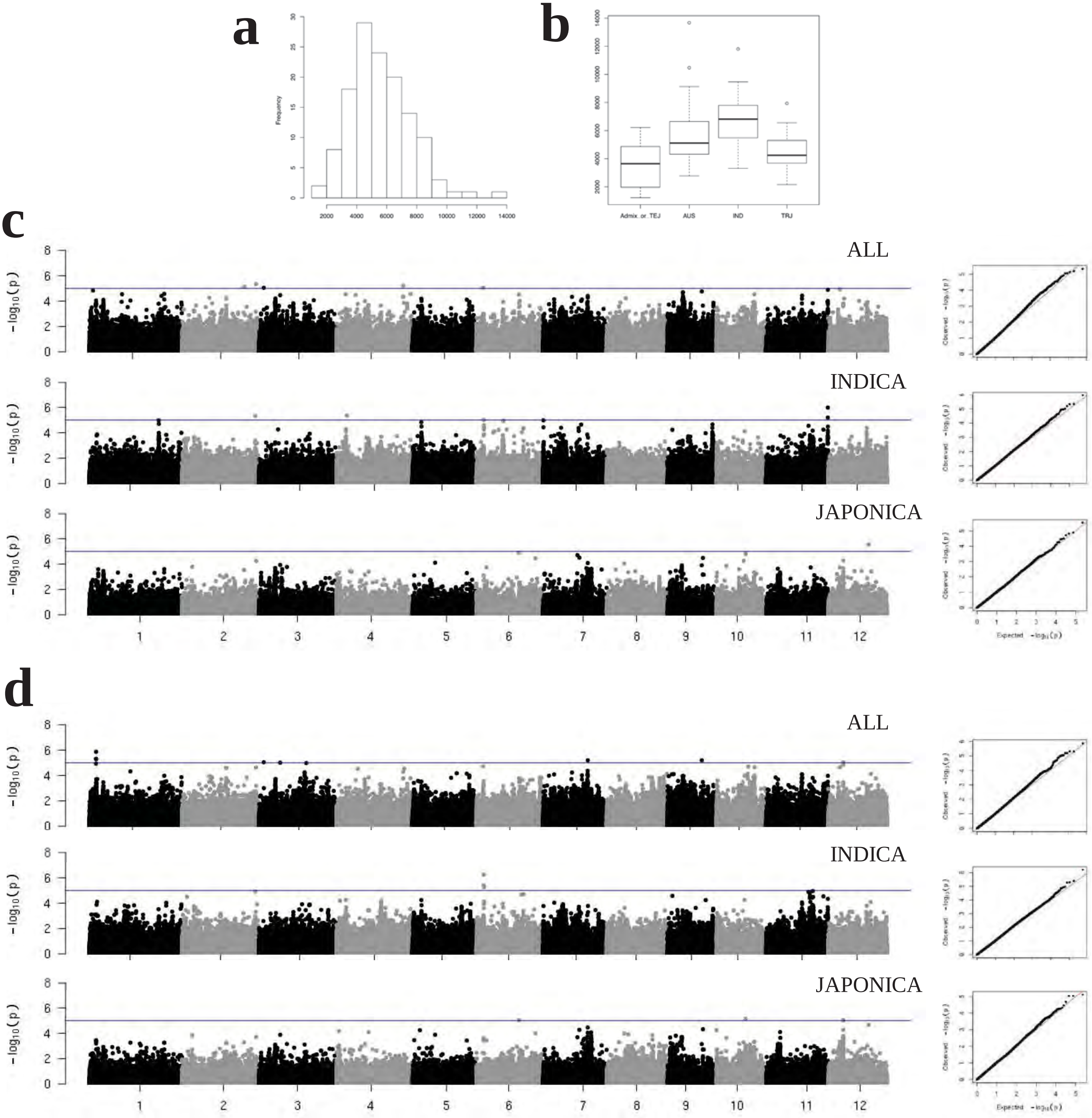

Supplementary Figure 58. Summary of GWAS results for Grain Number. (**a, b**) Phenotypic distributions. (**c**) Mixed model controlling for subpopulation structure across all varieties (ALL) and within the two major varietal groups *Indica* (INDICA) and *Japonica* (JAPONICA). (**d**) Mixed model with heading date as a covariate.

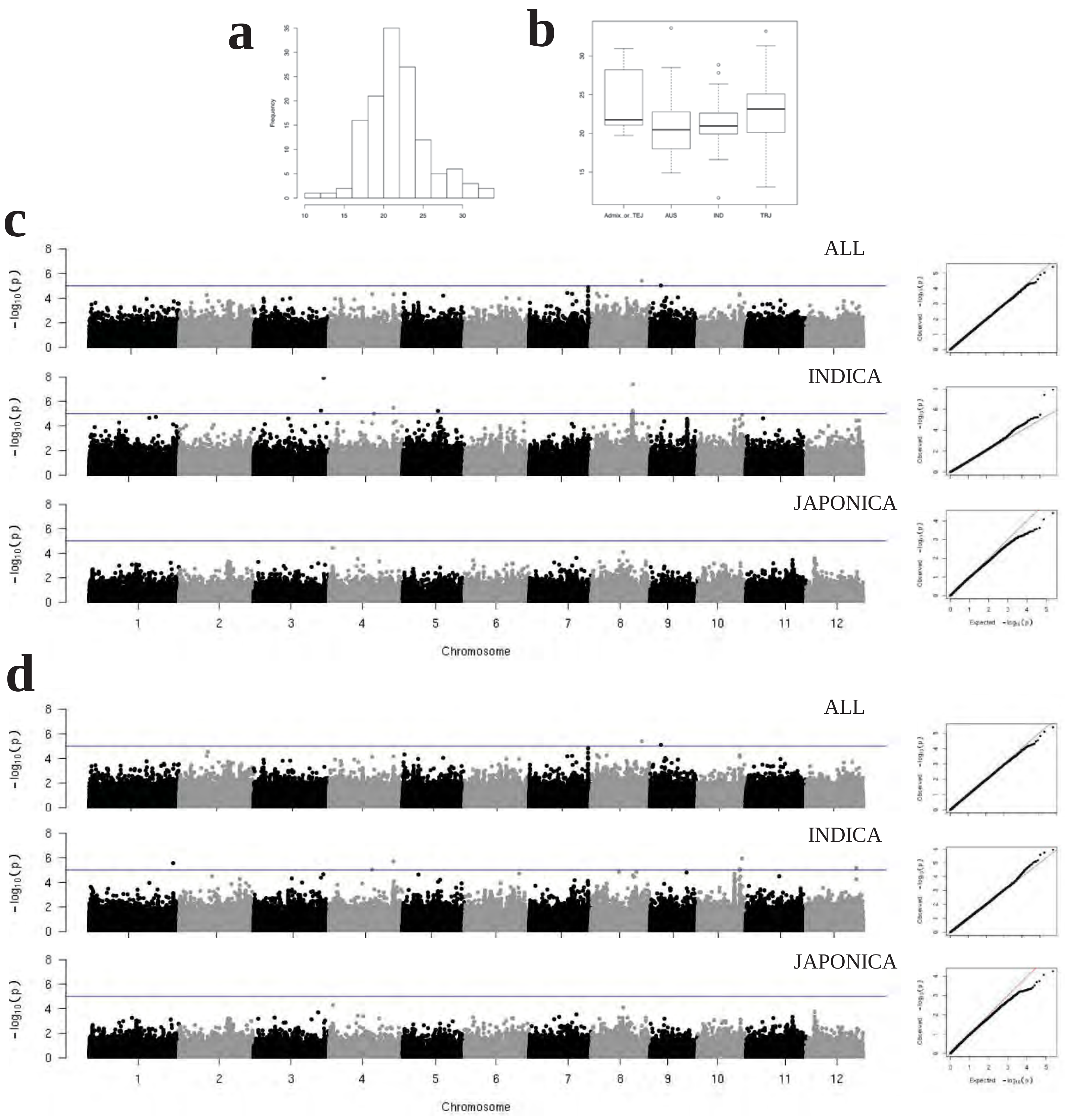

Supplementary Figure 59. Summary of GWAS results for 1000 Grain Weight (g). (**a, b**) Phenotypic distributions. (**c**) Mixed model controlling for subpopulation structure across all varieties (ALL) and within the two major varietal groups *Indica* (INDICA) and *Japonica* (JAPONICA). (**d**) Mixed model with heading date as a covariate.

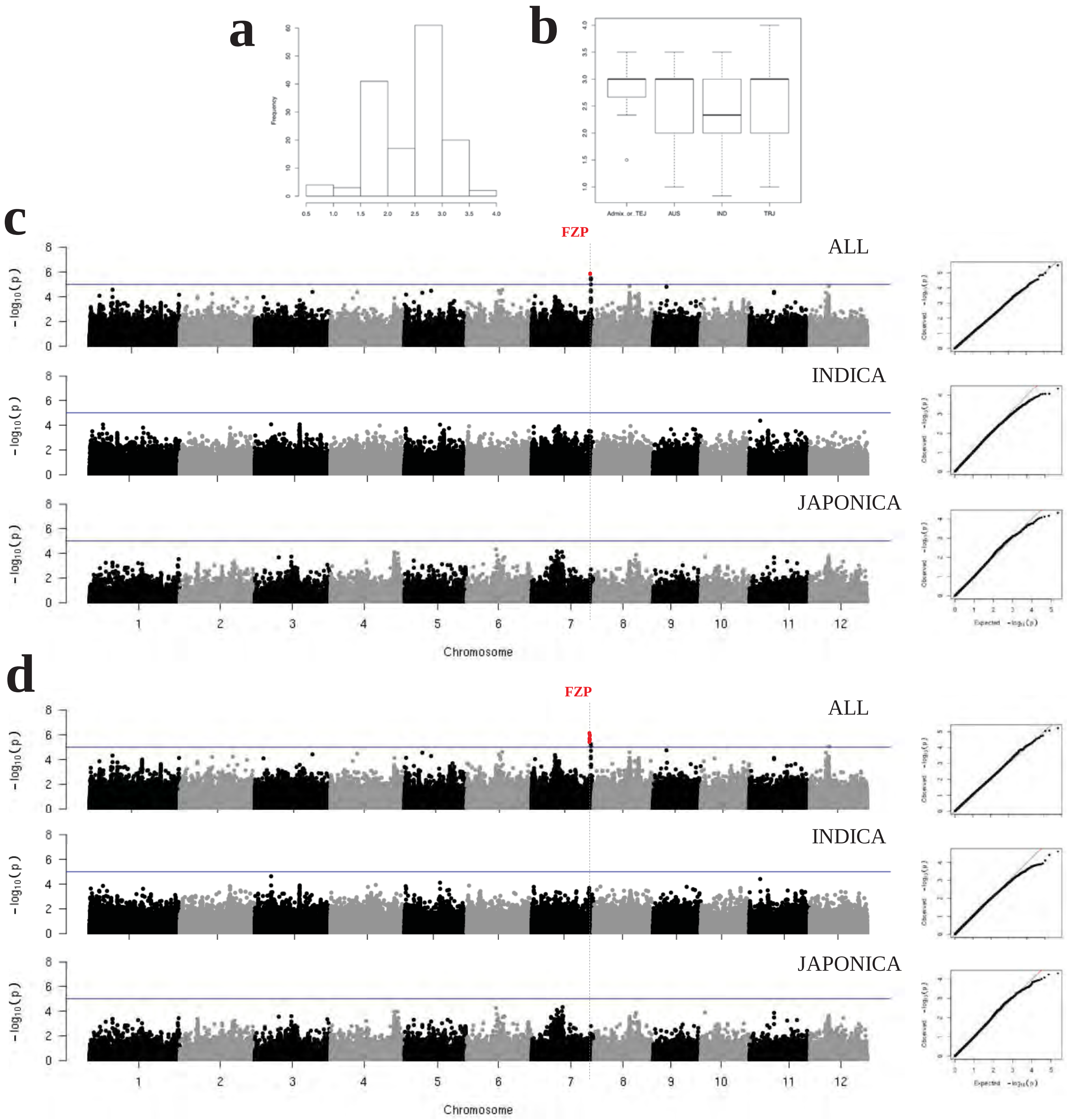

Supplementary Figure 60. Summary of GWAS results for Secondary Panicle Branching. (**a, b**) Phenotypic distributions. (**c**) Mixed model controlling for subpopulation structure across all varieties (ALL) and within the two major varietal groups *Indica* (INDICA) and *Japonica* (JAPONICA). (**d**) Mixed model with heading date as a covariate.

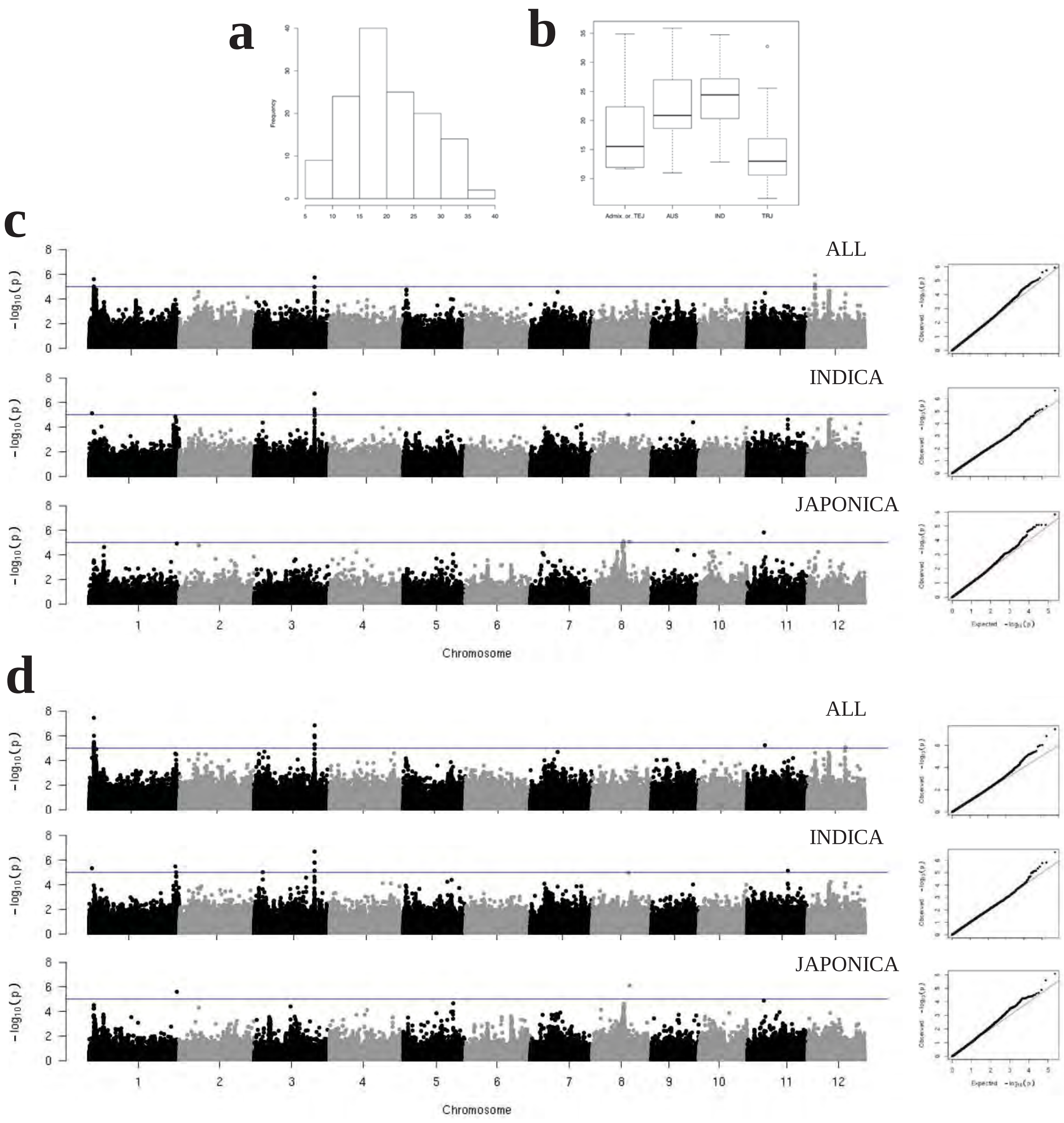

Supplementary Figure 61. Summary of GWAS results for Panicle Number. (**a, b**) Phenotypic distributions. (**c**) Mixed model controlling for subpopulation structure across all varieties (ALL) and within the two major varietal groups *Indica* (INDICA) and *Japonica* (JAPONICA). (**d**) Mixed model with heading date as a covariate.

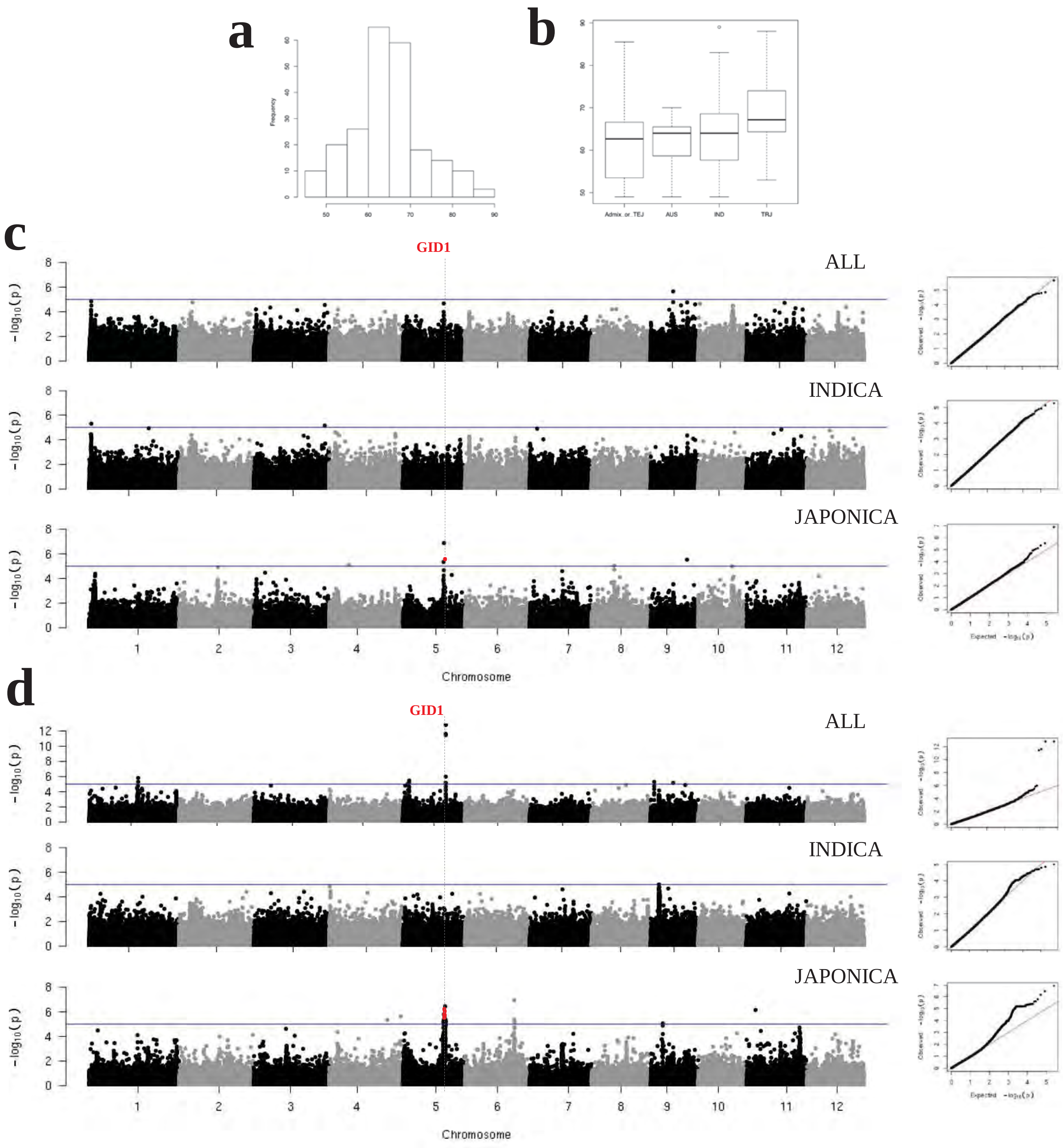

Supplementary Figure 62. Summary of GWAS results for Booting Date (days after transplanting). (**a, b**) Phenotypic distributions. (**c**) Mixed model controlling for subpopulation structure across all varieties (ALL) and within the two major varietal groups *Indica* (INDICA) and *Japonica* (JAPONICA). (**d**) Mixed model with heading date as a covariate.

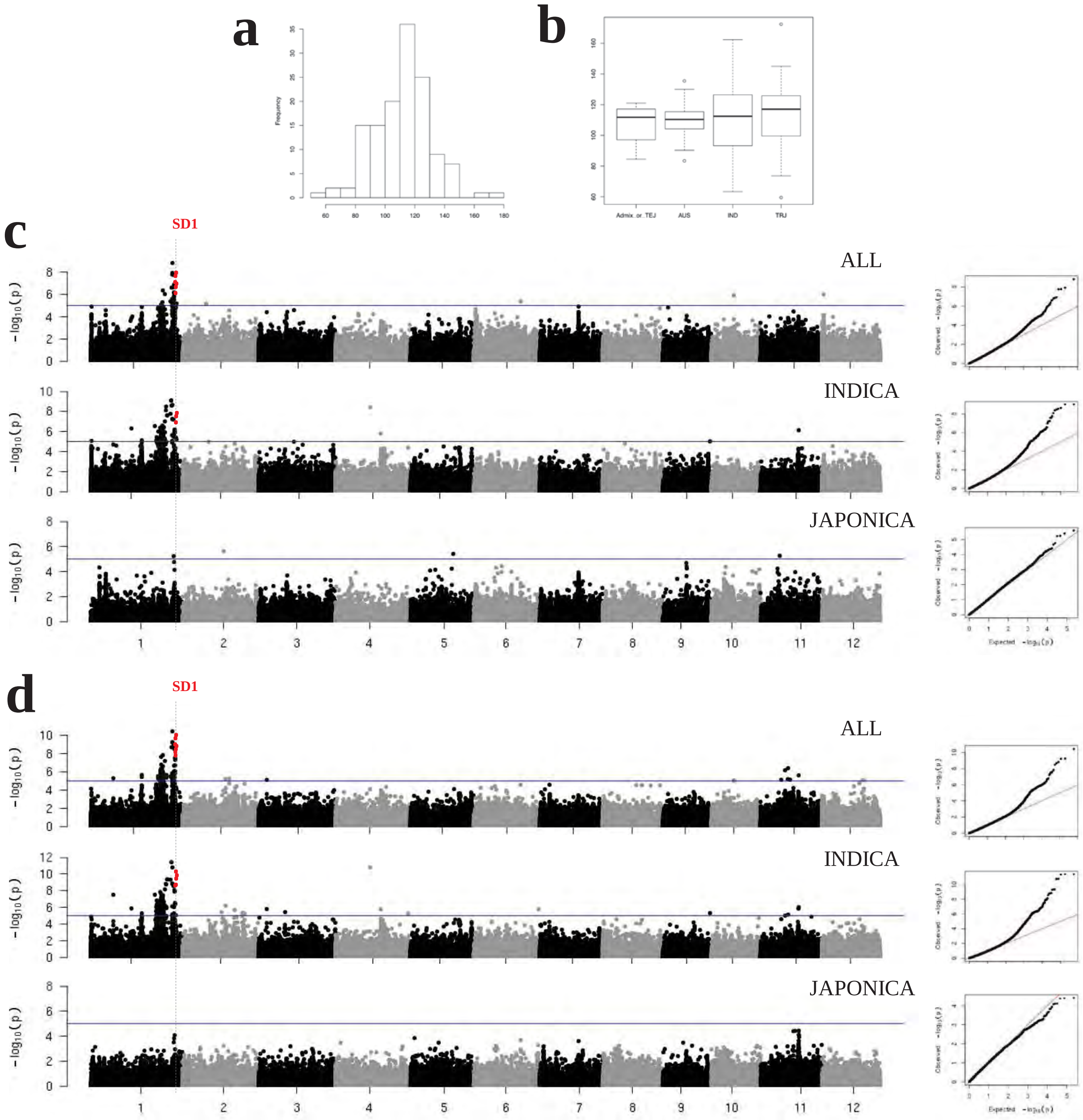

Supplementary Figure 63. Summary of GWAS results for Plant Height (cm). (**a, b**) Phenotypic distributions. (**c**) Mixed model controlling for subpopulation structure across all varieties (ALL) and within the two major varietal groups *Indica* (INDICA) and *Japonica* (JAPONICA). (**d**) Mixed model with heading date as a covariate.

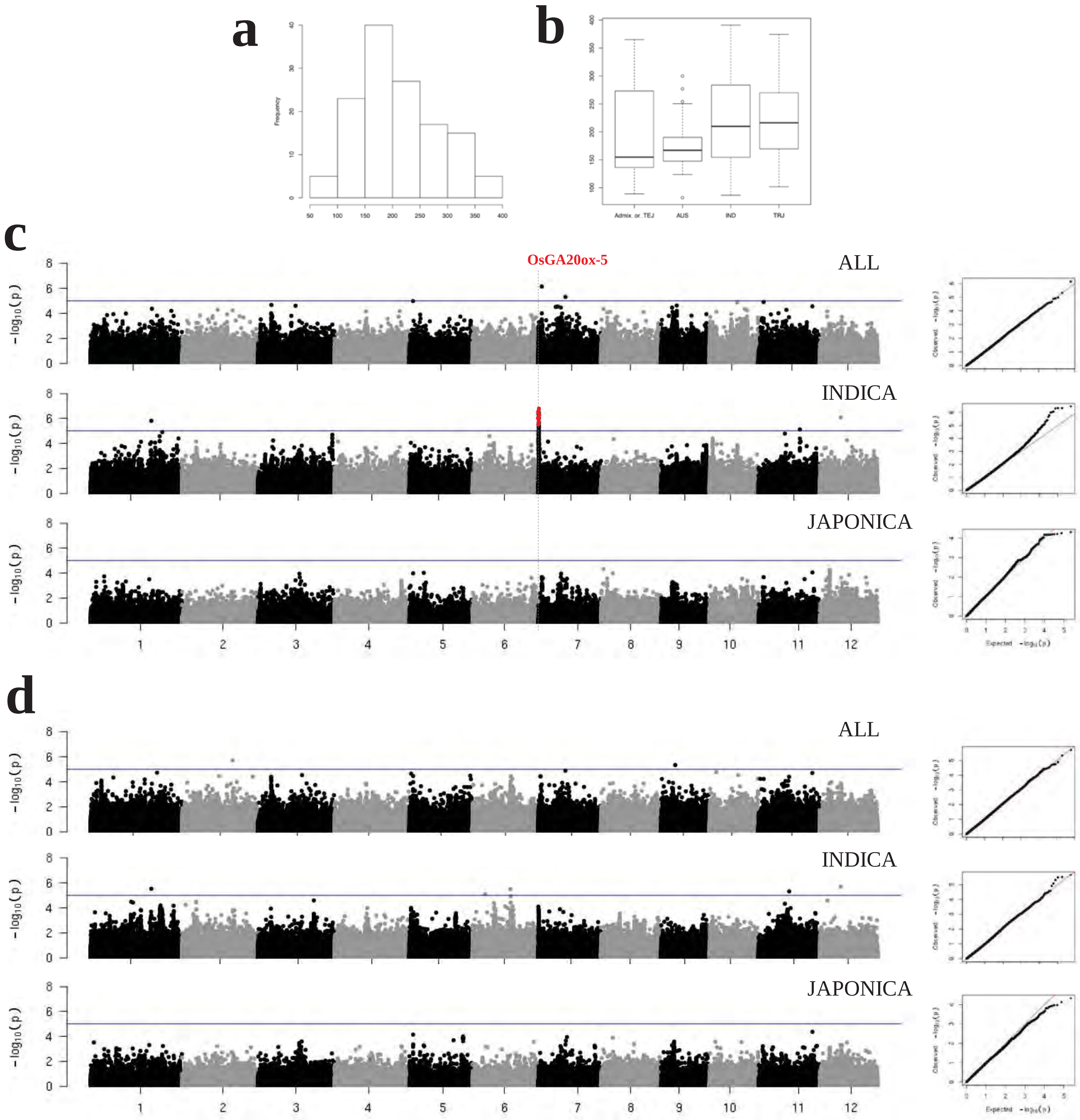

Supplementary Figure 64. Summary of GWAS results for Total Vegetative Biomass (g). (**a, b**) Phenotypic distributions. (**c**) Mixed model controlling for subpopulation structure across all varieties (ALL) and within the two major varietal groups *Indica* (INDICA) and *Japonica* (JAPONICA). (**d**) Mixed model with heading date as a covariate.

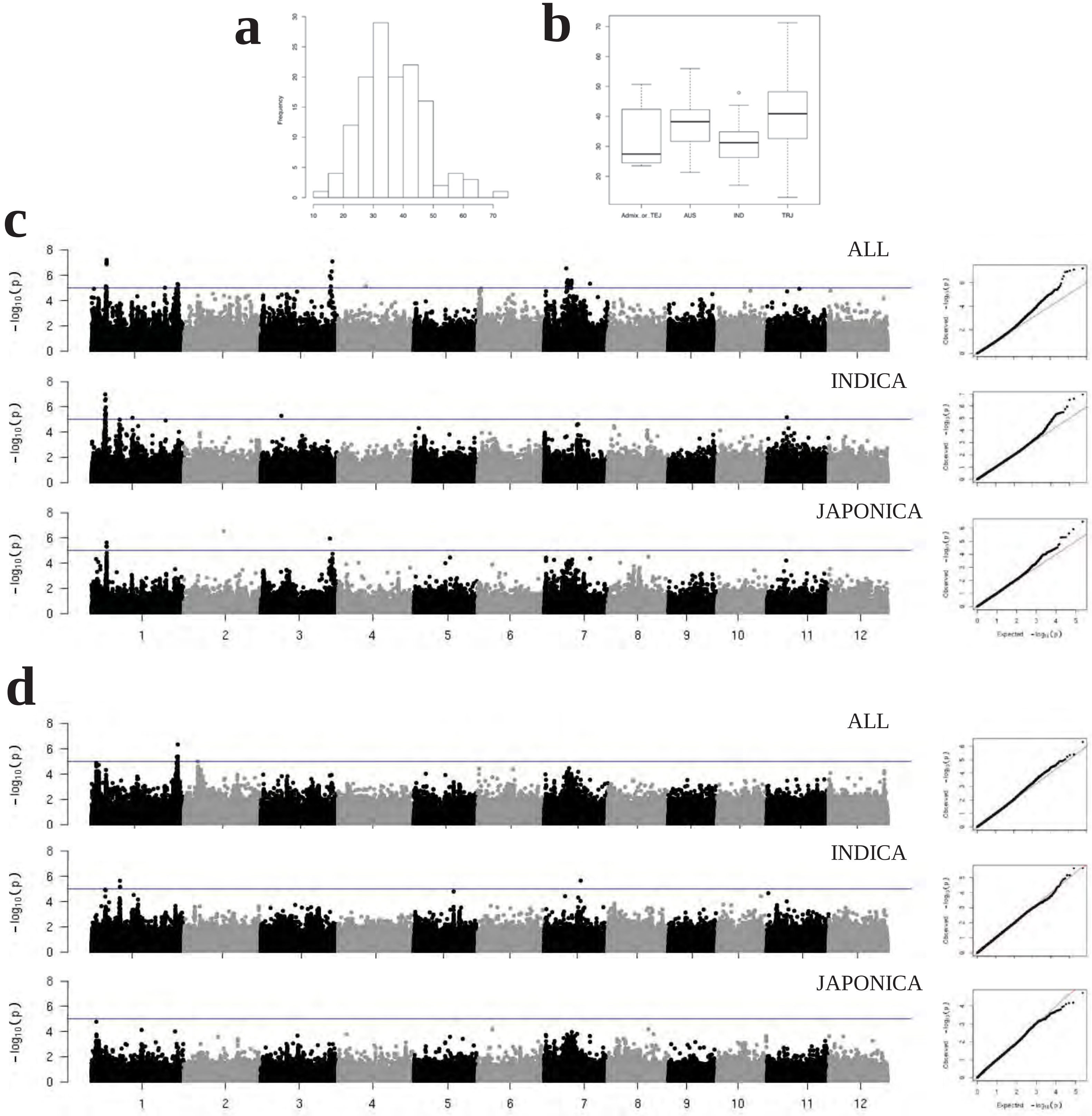

Supplementary Figure 65. Summary of GWAS results for Flag Leaf Area (cm^2). (**a, b**) Phenotypic distributions. (**c**) Mixed model controlling for subpopulation structure across all varieties (ALL) and within the two major varietal groups *Indica* (INDICA) and *Japonica* (JAPONICA). (**d**) Mixed model with heading date as a covariate.

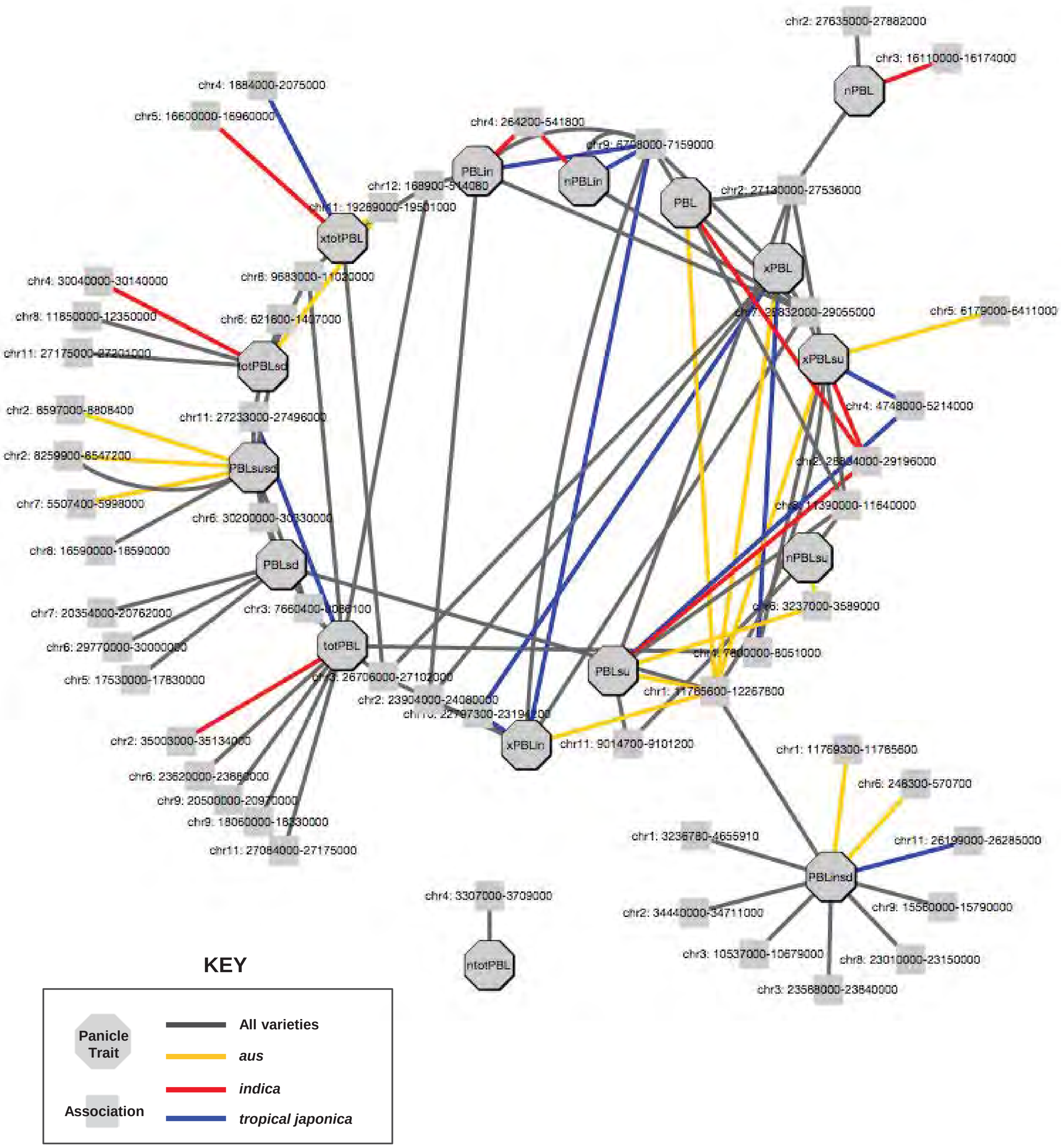

Supplementary Figure 66. Association Network Summarizing GWAS results for Primary Branch length traits. Primary branch length traits show an enrichment for subpopulation specific associations, when compared to primary branch number traits. Trait abbreviations can be found in Supplementary Table 2.

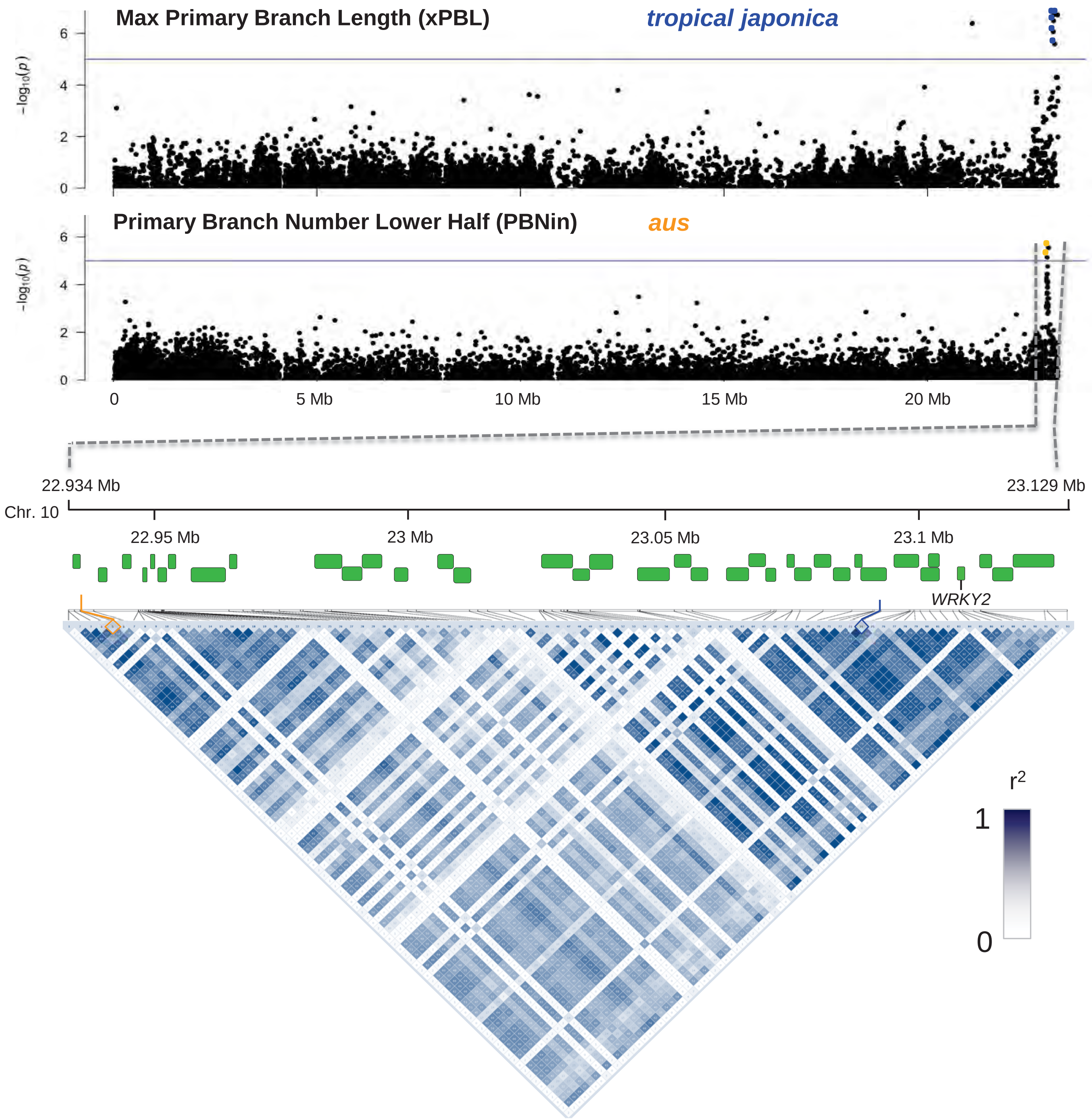

Supplementary Figure 67. Overlapping subpopulation-specific panicle trait peaks. Manhattan plots depicting significant associations for panicle traits in two subpopulations on the distal end of chromosome 10: maximum primary branch length (*tropical japonica* panel), and primary branch number in the lower half of the panicle (*aus* panel). The x-axis depicts the physical location of SNPs across chromosome 10, and the y-axis depicts the $-\log_{10}(P \text{ value})$. Significant SNPs ($P < 1 \times 10^{-5}$) are depicted as colored dots, labeled to match the group in which they were identified: *tropical japonica* (blue) and *aus* (yellow). The zoom-in plot depicts ~195kb region (grey dotted lines), with gene models in green. The heatmap depicts linkage disequilibrium (LD), measured as r^2, between SNPs calculated using *aus* varieties. The most significant SNP for the *aus* and *tropical japonica* peaks are labeled in yellow and blue. The candidate gene, *WRKY2*, is located ~30kb of the peak SNP for the maximum primary branch length. These two peaks were the only peaks for panicle traits that are in an LD block and were identified in two separate subpopulations.

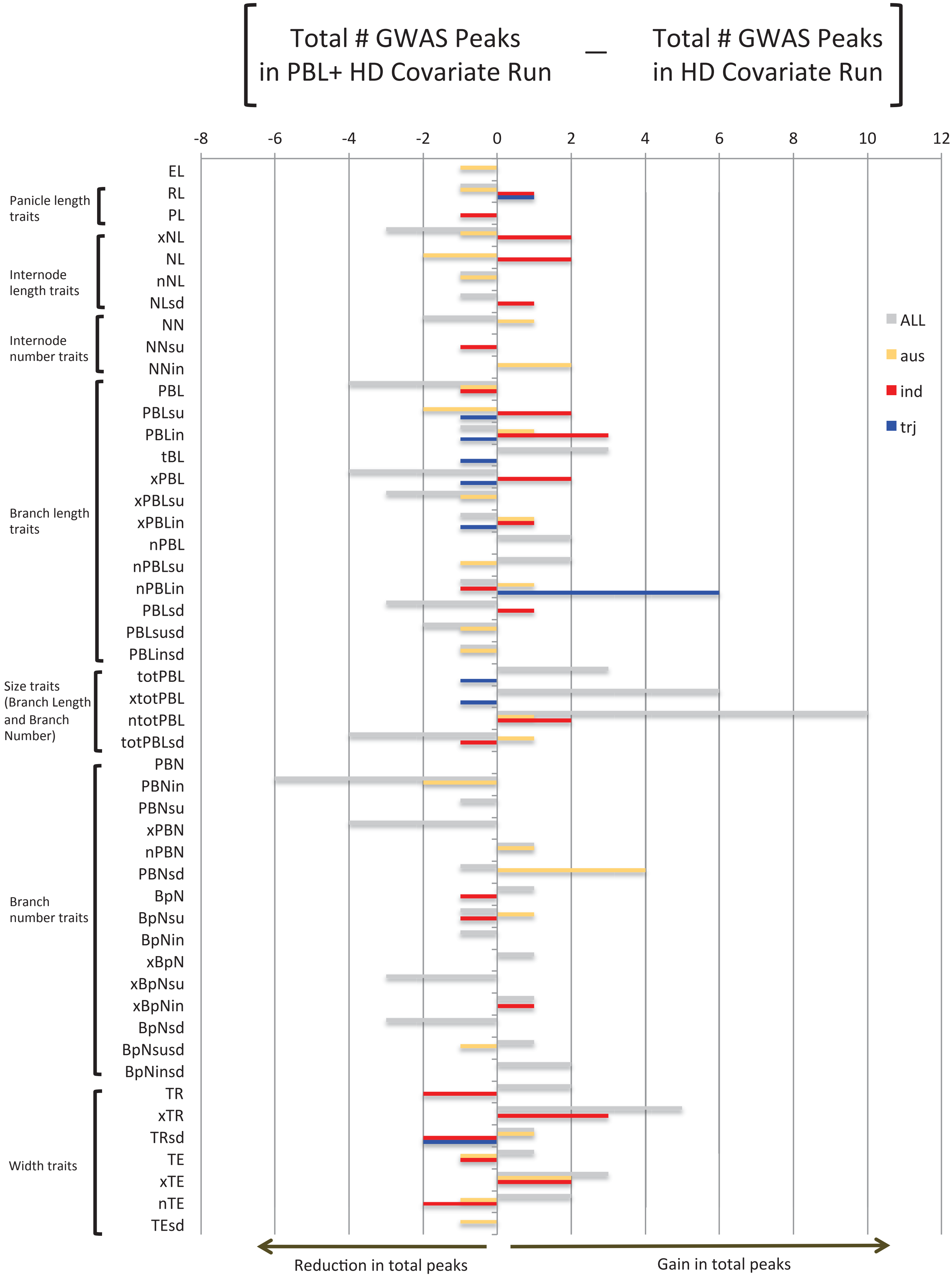

Supplementary Figure 68. PBL covariate impacts GWAS peaks. Bar graph depicts the difference between the total number of GWAS peaks, identified in the mixed model incorporating primary branch length (PBL) as a covariate vs. the mixed model only containing the heading date (HD) covariate.

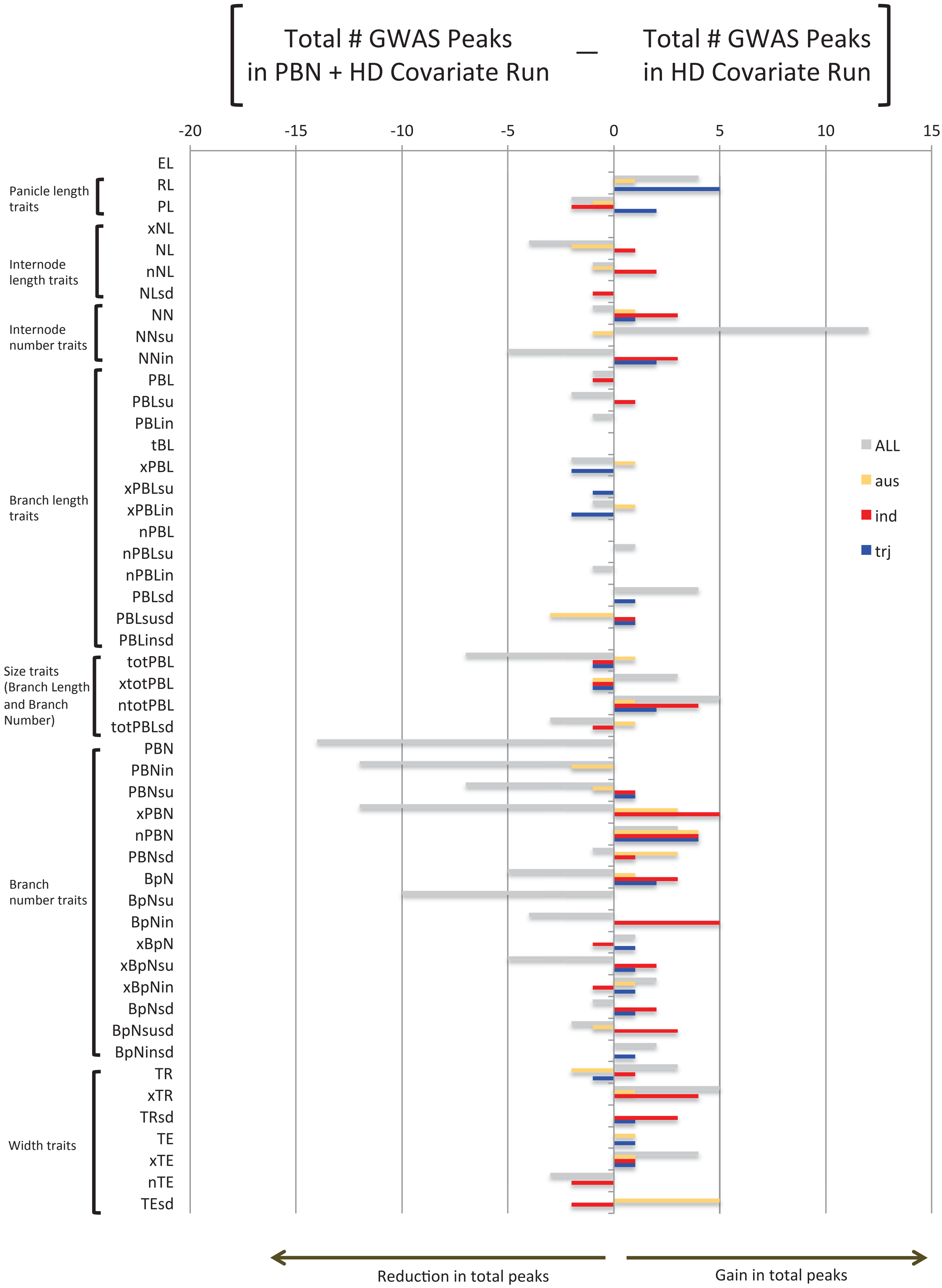

Supplementary Figure 69. PBN covariate impacts GWAS peaks. Bar graph depicts the difference between the total number of GWAS peaks, identified in the mixed model incorporating primary branch number (PBN) as a covariate vs. the mixed model only containing the heading date (HD) covariate.

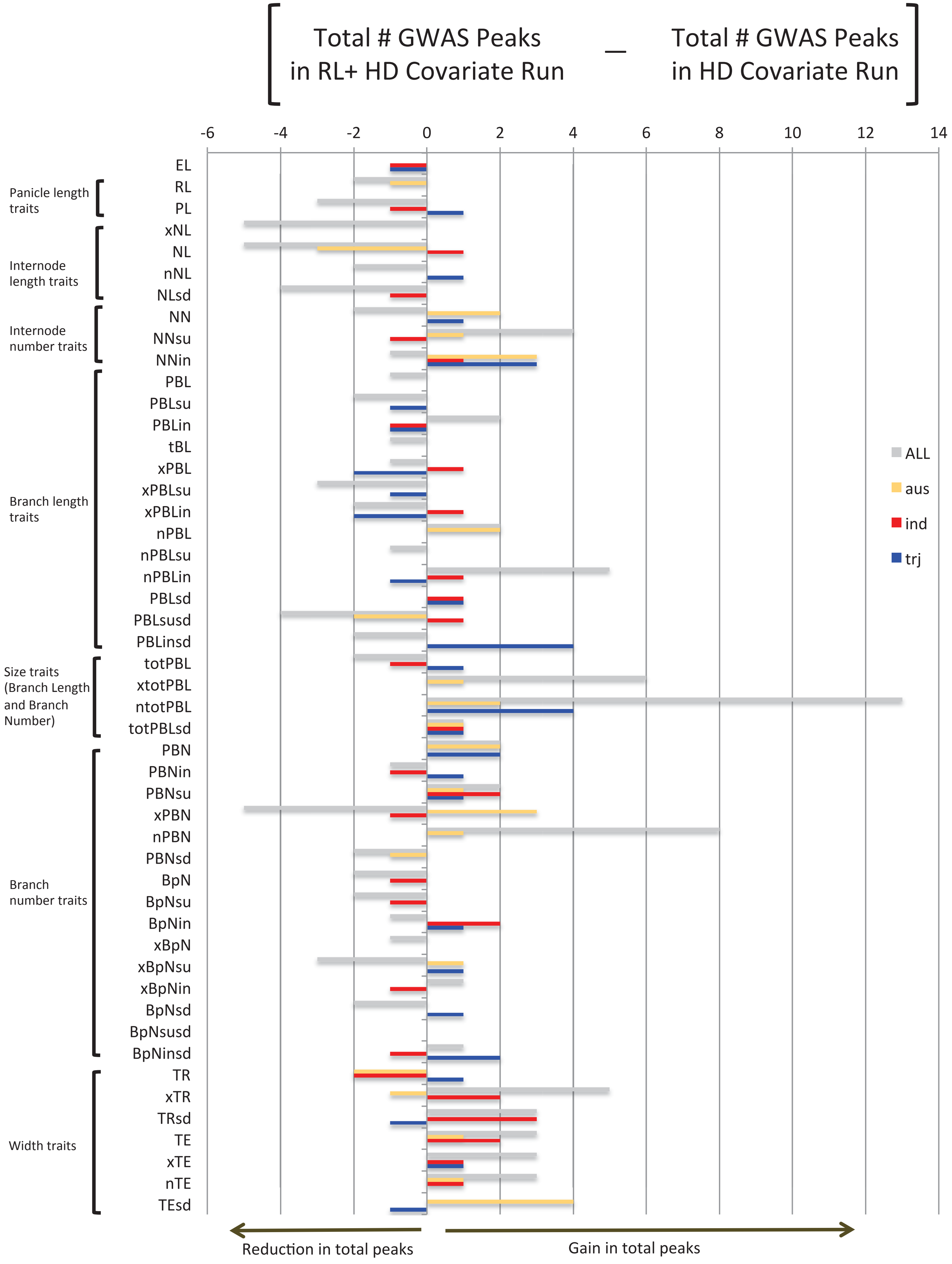

Supplementary Figure 70. RL covariate impacts GWAS peaks. Bar graph depicts the difference between the total number of GWAS peaks, identified in the mixed model incorporating rachis length (RL) as a covariate vs. the mixed model only containing the heading date (HD) covariate.

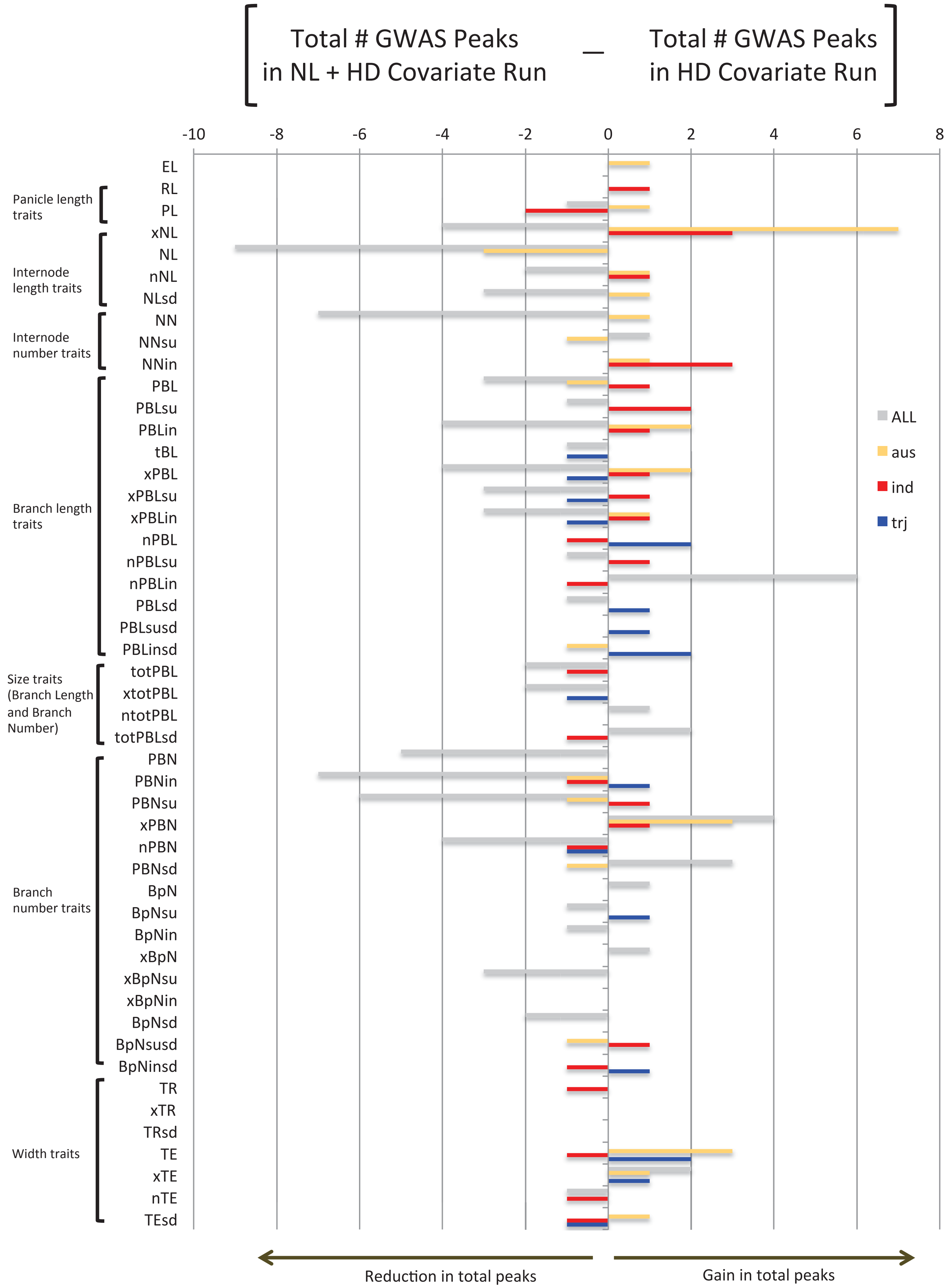

Supplementary Figure 71. NL covariate impacts GWAS peaks. Bar graph depicts the difference between the total number of GWAS peaks, identified in the mixed model incorporating internode length (RL) as a covariate vs. the mixed model only containing the heading date (HD) covariate.

Supplementary Figure 72. Panicle trait covariates and yield associations. Peaks detected using (**a**) the heading date (HD) covariate within the GWAS mixed model; (**b**) HD and primary branch length (PBL) covariate; (**c**) HD and primary branch number (PBN) covariate; (**d**) HD and rachis length (RL) covariate; or (**e**) HD and internode length (NL) covariate. Association networks where traits and linkage disequilibrium (LD) blocks containing significant SNPs ($P < 1 \times 10^{-5}$) are treated as nodes, and are connected if an LD block contains a significant association for the trait of interest. LD blocks are labeled with chromosome number and coordinates. The color and style of the edges connecting the trait and associations indicate the subpopulation or subspecies in which the association was detected. When multiple edges are present between a trait and LD block, a significant association was detected in more than one GWAS. To simplify networks, acronyms are used. Count traits: primary branch number (PBN), internode number (NN), and number of branches per internode (BpN). Length traits: panicle length (PL), rachis length (RL), primary branch length (PBL), and internode length (NL). Width traits: thickness of rachis (TR) and thickness of exsertion (TE). In addition to overall trait averages, sub-traits are depicted using acronyms with prefixes and/or suffixes: maximum (x-), minimum (n-), standard deviation (-sd), inferior (-in), and superior (-su). For example, maximum primary branch length in the inferior half of the panicle (xPBLin). Agronomic traits: booting (BT), total shoot biomass (totBM), flag leaf area (FLA), panicle weight (PW), grain number (GN), 1000-grain weight (1000GW), and panicle number (PN).

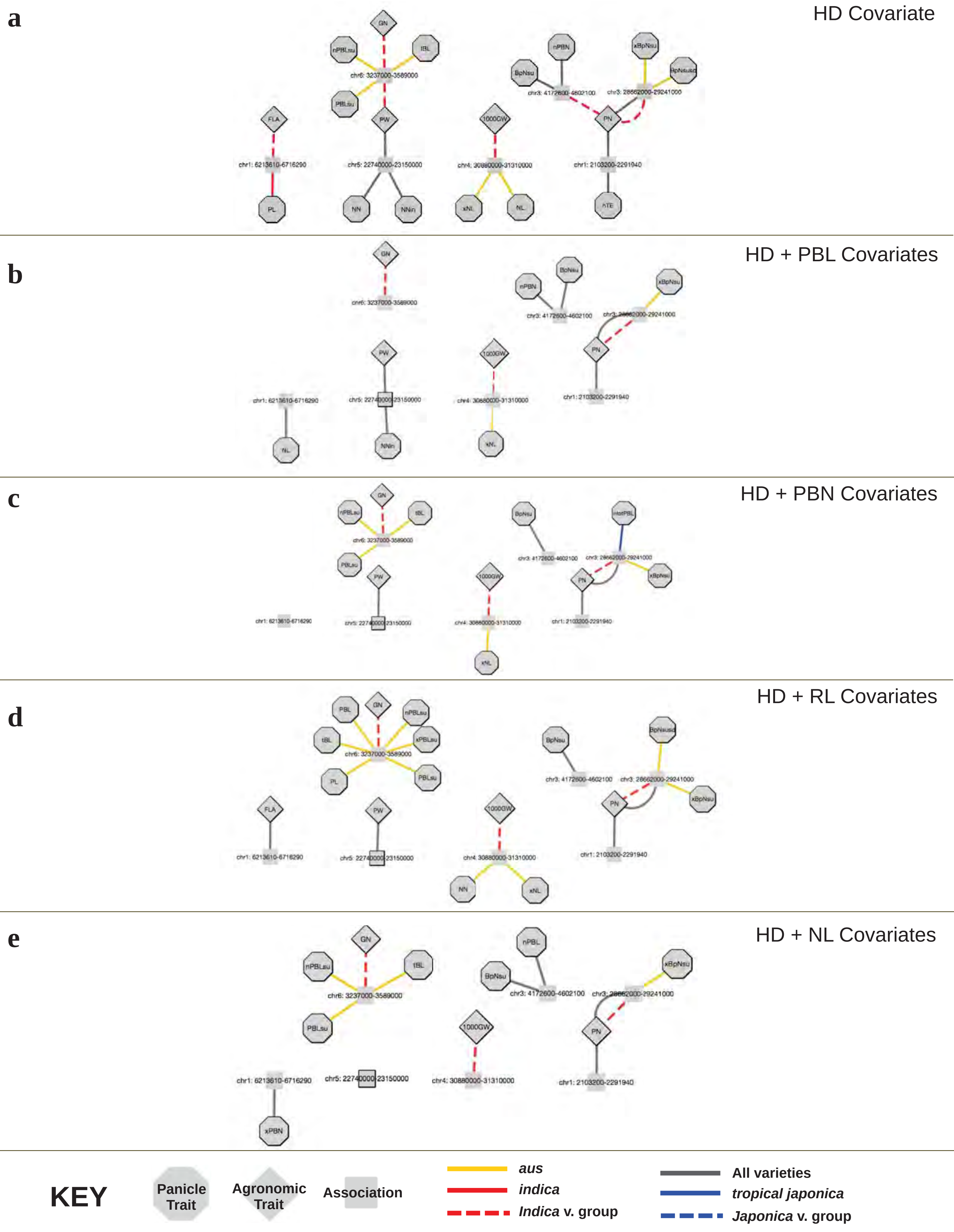
a
HD Covariate
b
HD + PBL Covariates
c
HD + PBN Covariates
d
HD + RL Covariates
e
HD + NL Covariates
KEY
Panicle Trait
Agronomic Trait
Association
aus
indica
Indica v. group
All varieties
tropical japonica
Japonica v. group

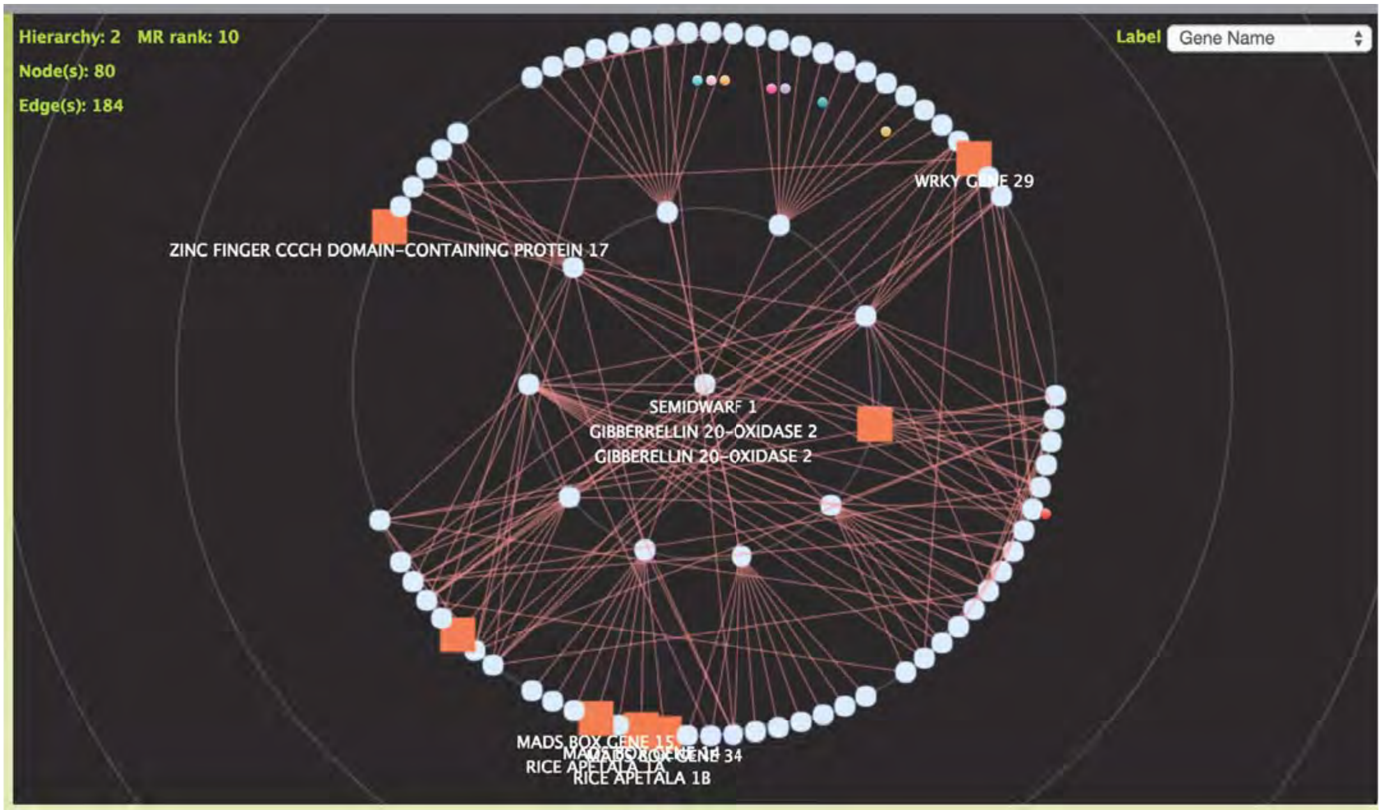

Os01g0883800
Hierarchy:2
MR / MR rank

Hierarchy	Node (Edge)	MR rank 1	2	3	4	5	10	15	50
	1	2 (1)	3 (2)	4 (3)	5 (7)	6 (8)	11 (19)	16 (30)	51 (205)
	2	3 (2)	7 (7)	13 (15)	18 (24)	27 (39)	80 (184)	-	-
	3	3 (2)	11 (11)	30 (36)	55 (75)	-	-	-	-
	4	3 (2)	16 (16)	58 (68)	-	-	-	-	-
	5	3 (2)	22 (23)	-	-	-	-	-	-

Locus ID	Os01g0883800 MR List HyperTree
Description	Gibberellin 20 oxidase 2 (EC 1.14.11.-) (Gibberellin C-20 oxidase 2) (GA 20-oxidase 2) (Os20ox2) (Semidwarf-1 protein).
GO BP	GO:0017000 : antibiotic biosynthesis
GO SLM BP	GO:0009058 : biosynthetic process<br>GO:0009987 : cellular process
GO CC	
GO SLM CC	
GO MF	GO:0005506 : iron ion binding<br>GO:0016216 : isopenicillin-N synthase activity
GO SLM MF	GO:0003824 : catalytic activity<br>GO:0005488 : binding
KEGG Pathway	
Transcription Factor	
Gene Symbol	SD1 (SEMIDWARF 1)<br>OS20OX2 (GIBBERELLIN 20-OXIDASE 2)<br>GA20OX2 (GIBBERELLIN 20-OXIDASE 2)
WoLF PSORT	nucl:3, cyto:3, plas:3, cyto_nucl:3, nucl_plas:3, cyto_plas:3
MSU ID	LOC_Os01g66100

Supplementary Figure 73. Coexpression network for *SEMIDWARF1*. The gene coexpression network was constructed on the RiceFREND database (http://ricefrend.dna.affrc.go.jp/) using *SD1* as a single gene guide.

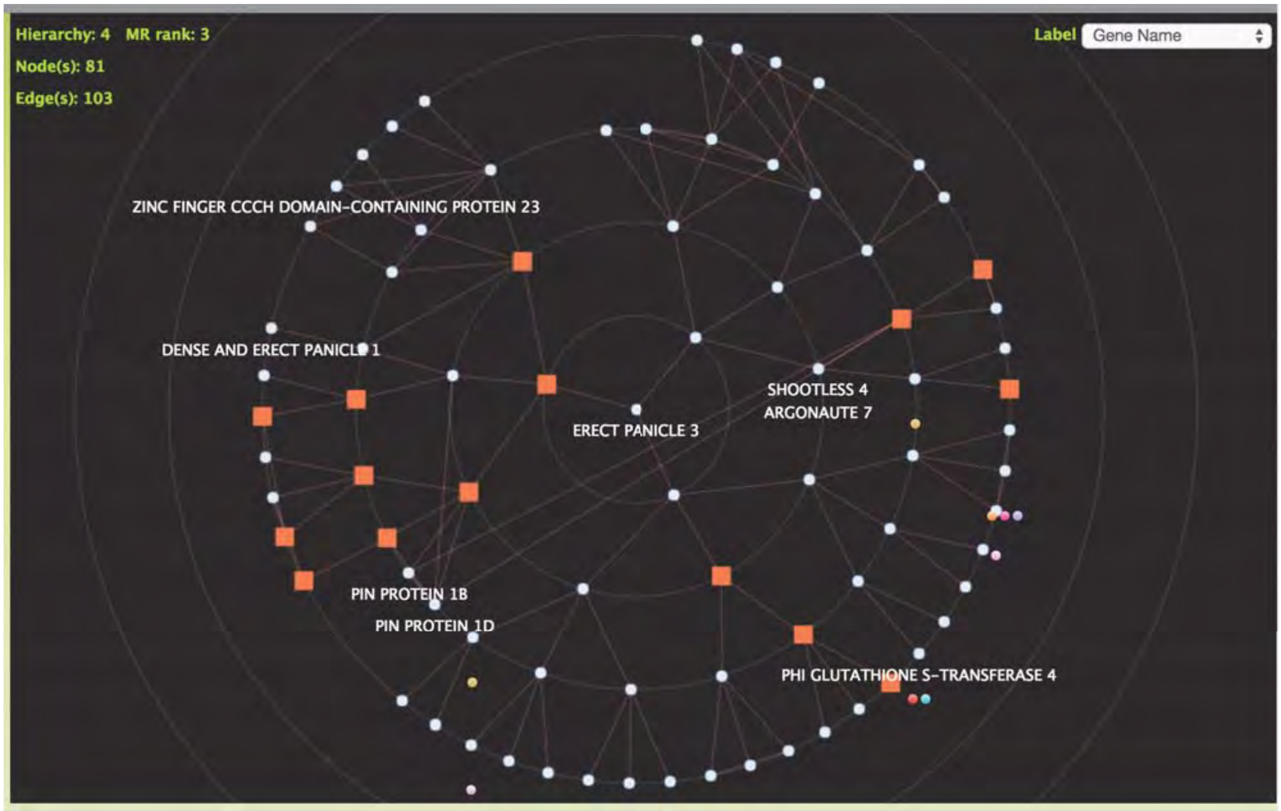

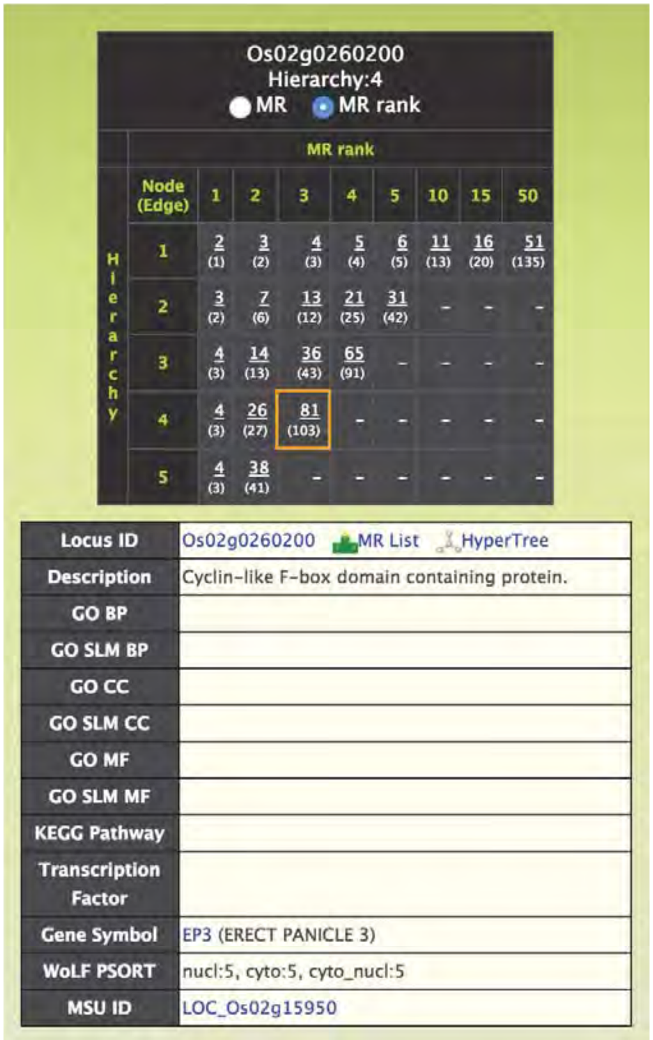

Os02g0260200
Hierarchy:4
MR MR rank

Hierarchy	MR rank: Node (Edge)	1	2	3	4	5	10	15	50
1		2 (1)	3 (2)	4 (3)	5 (4)	6 (5)	11 (13)	16 (20)	51 (135)
2		3 (2)	7 (6)	13 (12)	21 (25)	31 (42)	-	-	-
3		4 (3)	14 (13)	36 (43)	65 (91)	-	-	-	-
4		4 (3)	26 (27)	81 (103)	-	-	-	-	-
5		4 (3)	38 (41)	-	-	-	-	-	-

Locus ID	Os02g0260200 MR List HyperTree
Description	Cyclin-like F-box domain containing protein.
GO BP	
GO SLM BP	
GO CC	
GO SLM CC	
GO MF	
GO SLM MF	
KEGG Pathway	
Transcription Factor	
Gene Symbol	EP3 (ERECT PANICLE 3)
WoLF PSORT	nucl:5, cyto:5, cyto_nucl:5
MSU ID	LOC_Os02g15950

Supplementary Figure 74. Coexpression network for *ERECT PANICLE 3*. The gene coexpression network was constructed on the RiceFREND database (http://ricefrend.dna.affrc.go.jp/) using *EP3* as a single gene guide.

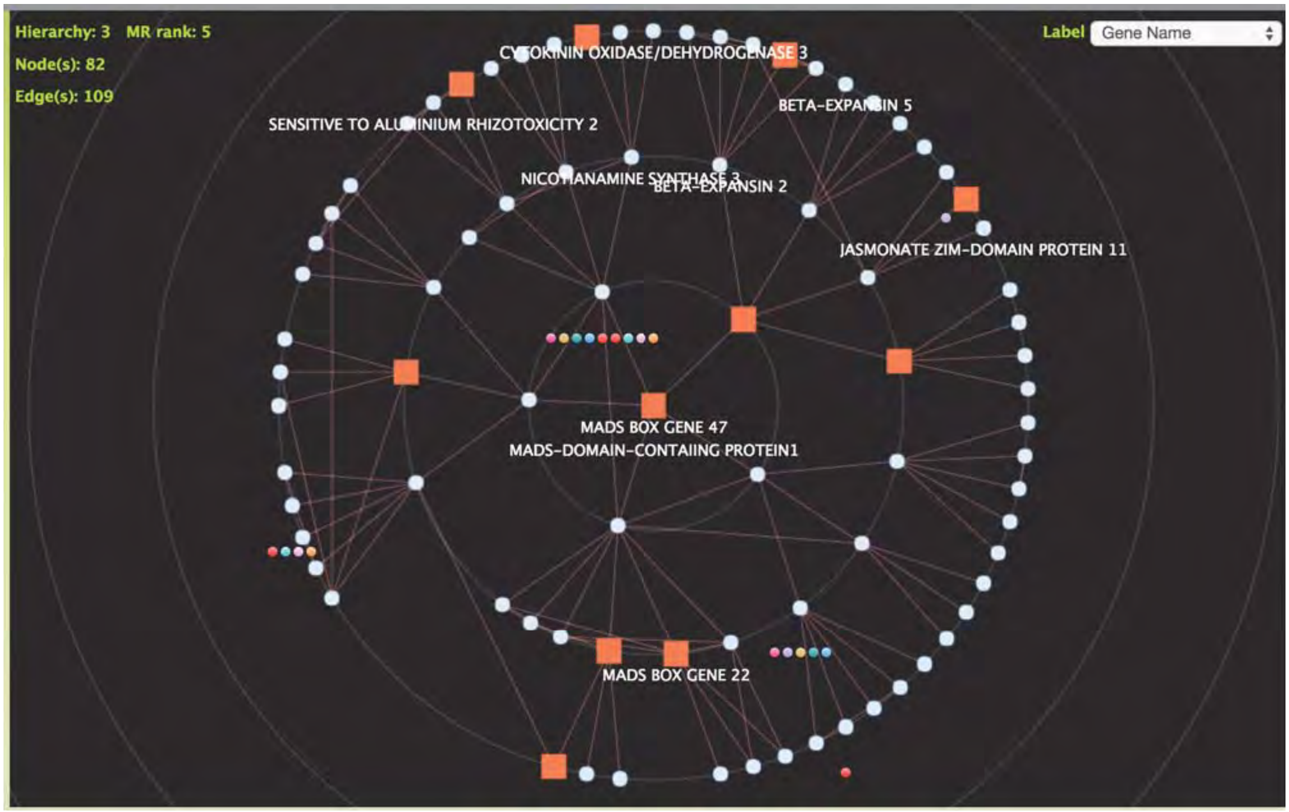

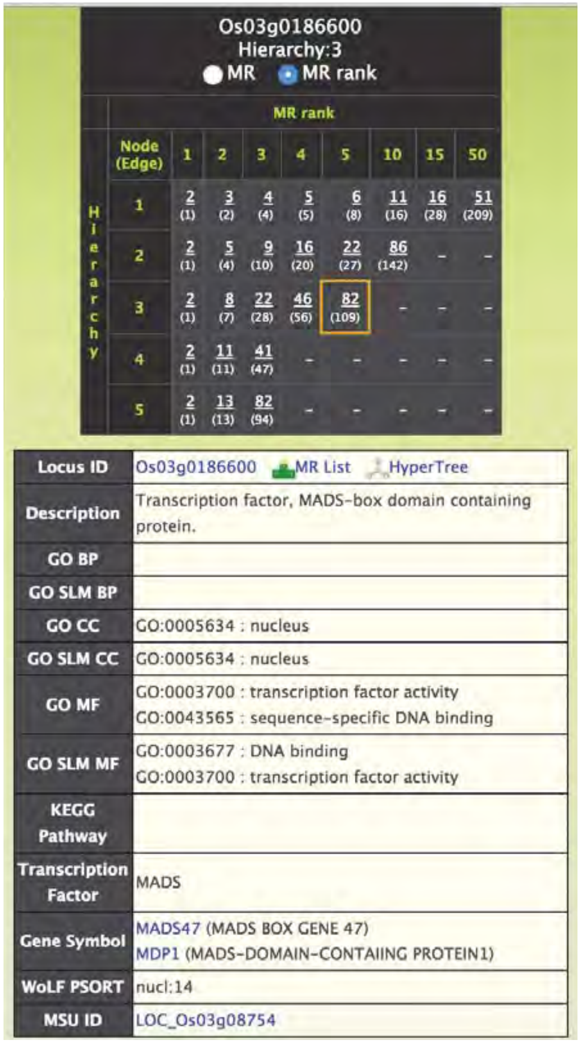

Os03g0186600
Hierarchy:3
MR MR rank

Hierarchy \ Node (Edge)	MR rank 1	2	3	4	5	10	15	50
1	2 (1)	3 (2)	4 (4)	5 (5)	6 (8)	11 (16)	16 (28)	51 (209)
2	2 (1)	5 (4)	9 (10)	16 (20)	22 (27)	86 (142)	-	-
3	2 (1)	8 (7)	22 (28)	46 (56)	82 (109)	-	-	-
4	2 (1)	11 (11)	41 (47)	-	-	-	-	-
5	2 (1)	13 (13)	82 (94)	-	-	-	-	-

Locus ID	Os03g0186600 MR List HyperTree
Description	Transcription factor, MADS-box domain containing protein.
GO BP	
GO SLM BP	
GO CC	GO:0005634 : nucleus
GO SLM CC	GO:0005634 : nucleus
GO MF	GO:0003700 : transcription factor activity<br>GO:0043565 : sequence-specific DNA binding
GO SLM MF	GO:0003677 : DNA binding<br>GO:0003700 : transcription factor activity
KEGG Pathway	
Transcription Factor	MADS
Gene Symbol	MADS47 (MADS BOX GENE 47)<br>MDP1 (MADS-DOMAIN-CONTAIING PROTEIN1)
WoLF PSORT	nucl:14
MSU ID	LOC_Os03g08754

Supplementary Figure 75. Coexpression network for *OsMADS47*. The gene coexpression network was constructed on the RiceFREND database (http://ricefrend.dna.affrc.go.jp/) using *OsMADs47* as a single gene guide.

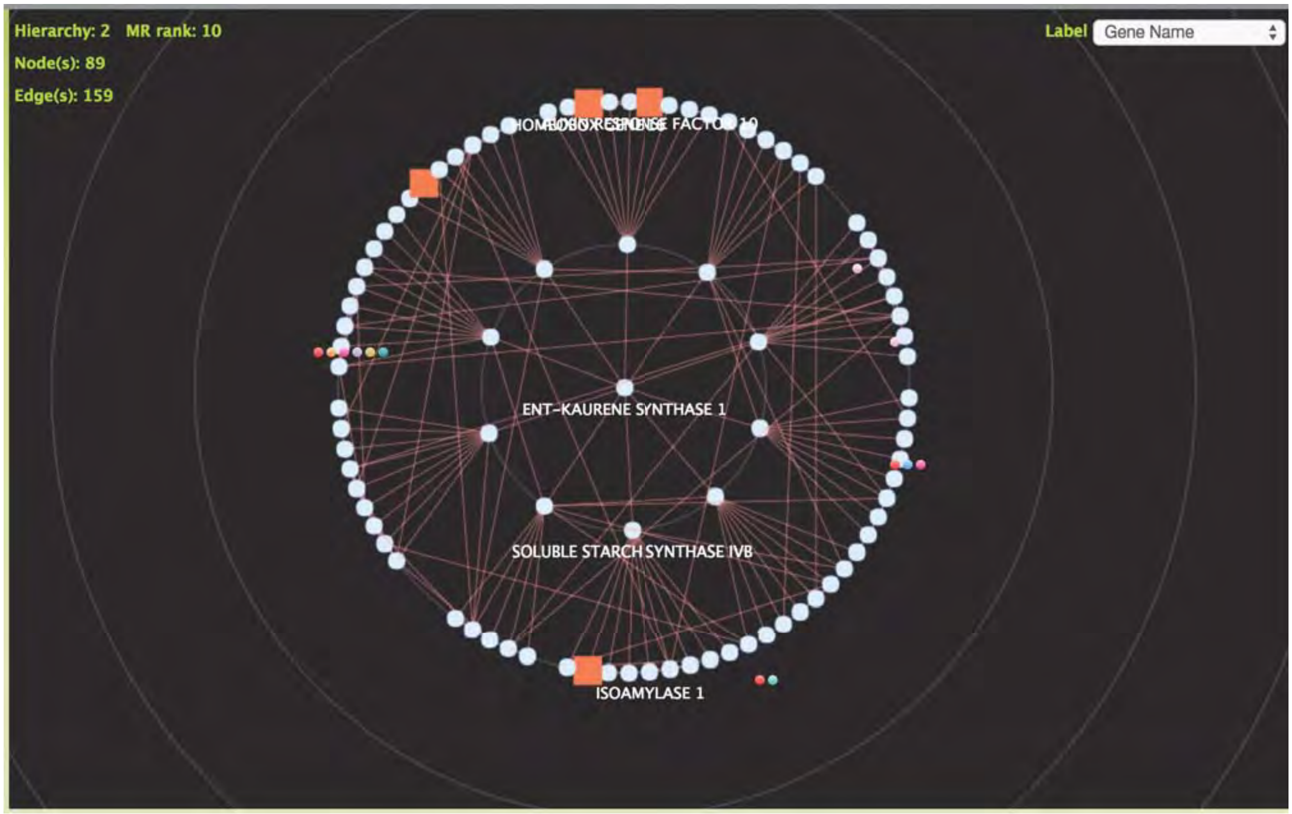

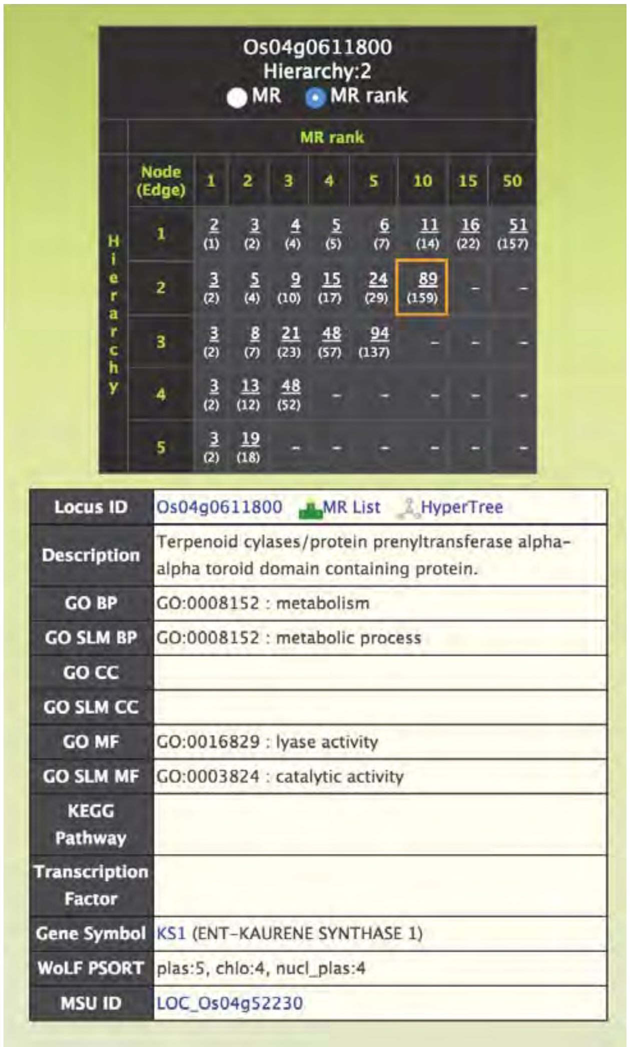

Os04g0611800
Hierarchy:2
MR MR rank

Hierarchy	Node (Edge)	MR rank 1	2	3	4	5	10	15	50
	1	2 (1)	3 (2)	4 (4)	5 (5)	6 (7)	11 (14)	16 (22)	51 (157)
	2	3 (2)	5 (4)	9 (10)	15 (17)	24 (29)	89 (159)	–	–
	3	3 (2)	8 (7)	21 (23)	48 (57)	94 (137)	–	–	–
	4	3 (2)	13 (12)	48 (52)	–	–	–	–	–
	5	3 (2)	19 (18)	–	–	–	–	–	–

Locus ID	Os04g0611800 MR List HyperTree
Description	Terpenoid cylases/protein prenyltransferase alpha-alpha toroid domain containing protein.
GO BP	GO:0008152 : metabolism
GO SLM BP	GO:0008152 : metabolic process
GO CC	
GO SLM CC	
GO MF	GO:0016829 : lyase activity
GO SLM MF	GO:0003824 : catalytic activity
KEGG Pathway	
Transcription Factor	
Gene Symbol	KS1 (ENT-KAURENE SYNTHASE 1)
WoLF PSORT	plas:5, chlo:4, nucl_plas:4
MSU ID	LOC_Os04g52230

Supplementary Figure 76. Coexpression network for *ent-Kaurene synthase (OsKS1).* The gene coexpression network was constructed on the RiceFREND database (http://ricefrend.dna.affrc.go.jp/) using *OsKS1* as a single gene guide.

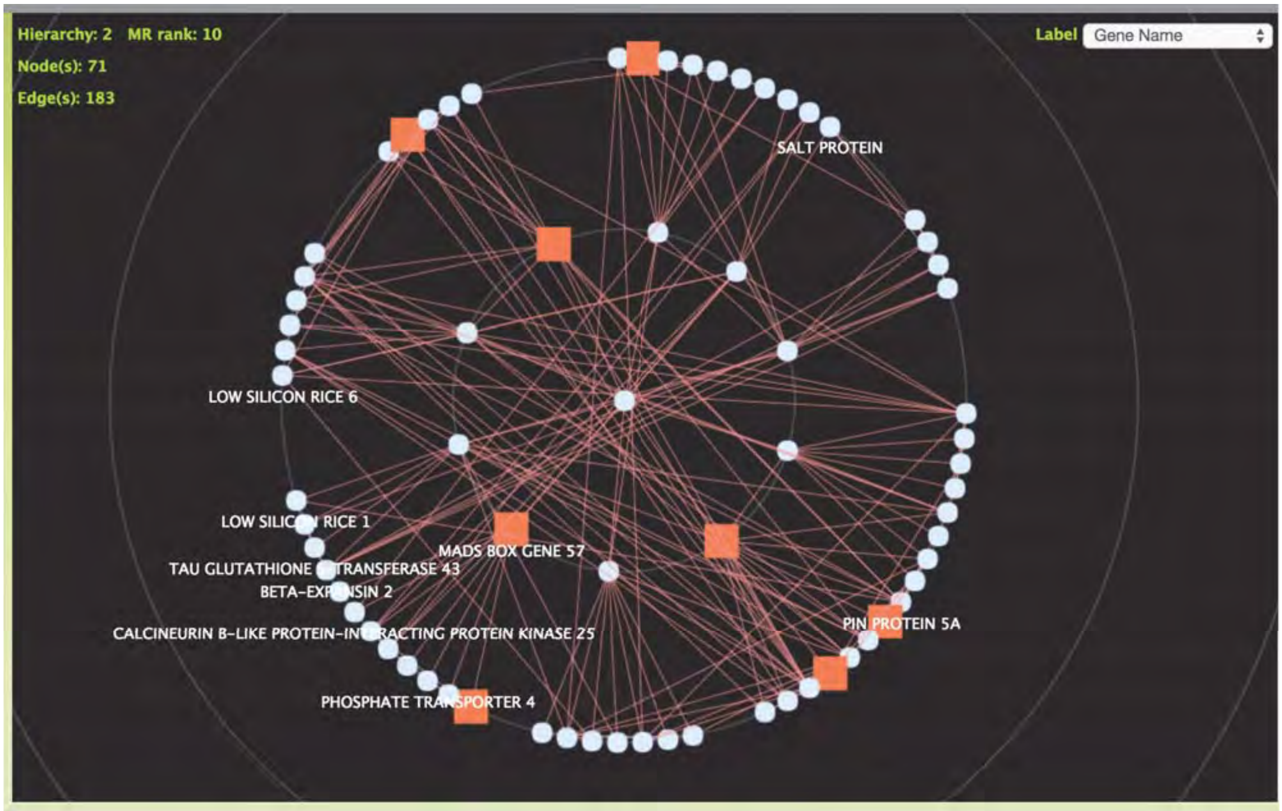

Os05g0200400
Hierarchy:2
MR MR rank

Hierarchy	Node (Edge)	MR rank 1	2	3	4	5	10	15	50
1		2 (1)	3 (2)	4 (3)	5 (5)	6 (8)	11 (18)	16 (35)	51 (315)
2		2 (1)	5 (4)	9 (9)	16 (23)	21 (31)	71 (183)	-	-
3		2 (1)	8 (8)	21 (23)	42 (57)	71 (101)	-	-	-
4		2 (1)	10 (10)	40 (46)	-	-	-	-	-
5		2 (1)	14 (14)	76 (89)	-	-	-	-	-

Locus ID	Os05g0200400 MR List HyperTree
Description	Cytochrome P450 family protein.
GO BP	GO:0006118 : electron transport
GO SLM BP	
GO CC	
GO SLM CC	
GO MF	GO:0004497 : monooxygenase activity<br>GO:0005506 : iron ion binding<br>GO:0020037 : heme binding
GO SLM MF	GO:0003824 : catalytic activity<br>GO:0005488 : binding
KEGG Pathway	
Transcription Factor	
Gene Symbol	
WoLF PSORT	cyto:6, extr:3, cyto_pero:3, cyto_E.R.:3
MSU ID	LOC_Os05g11130

Supplementary Figure 77. Coexpression network for *CYP90D3*. The gene coexpression network was constructed on the RiceFREND database (http://ricefrend.dna.affrc.go.jp/) using CYP90D3 as a single gene guide.

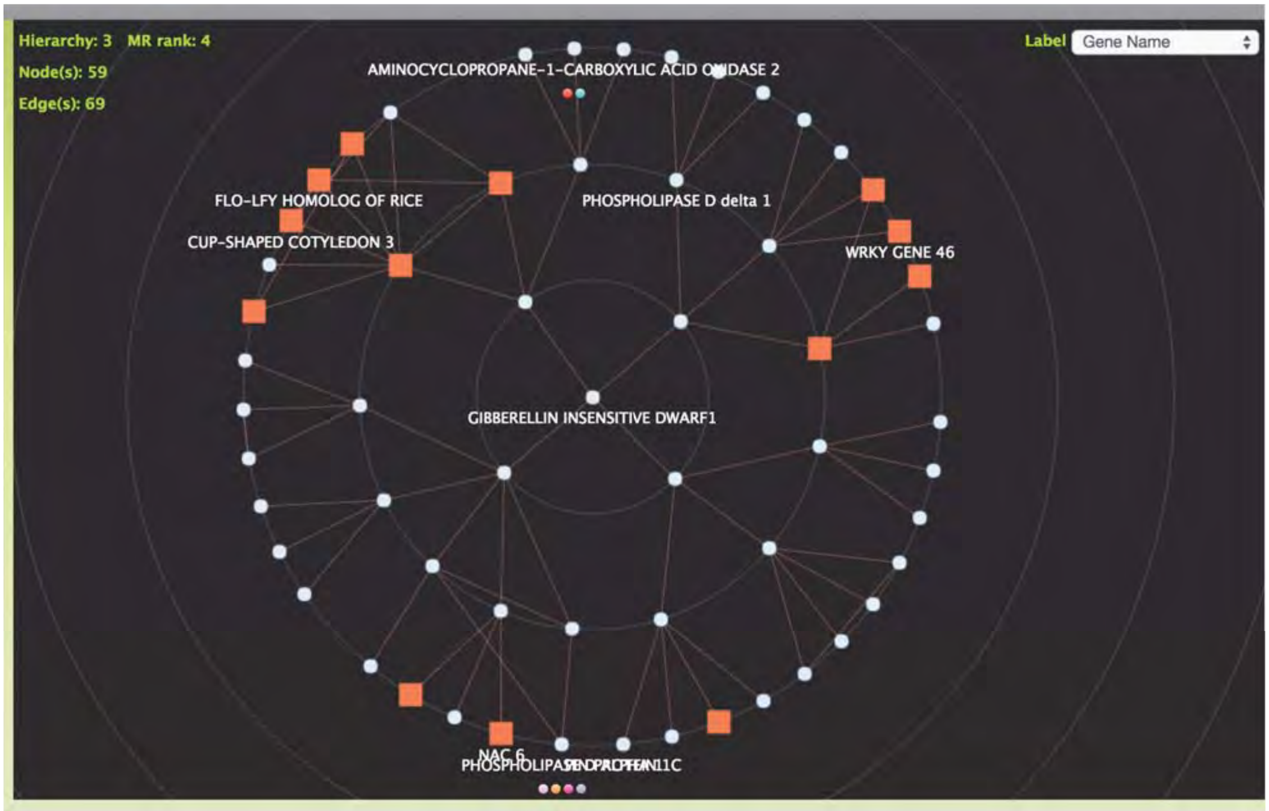

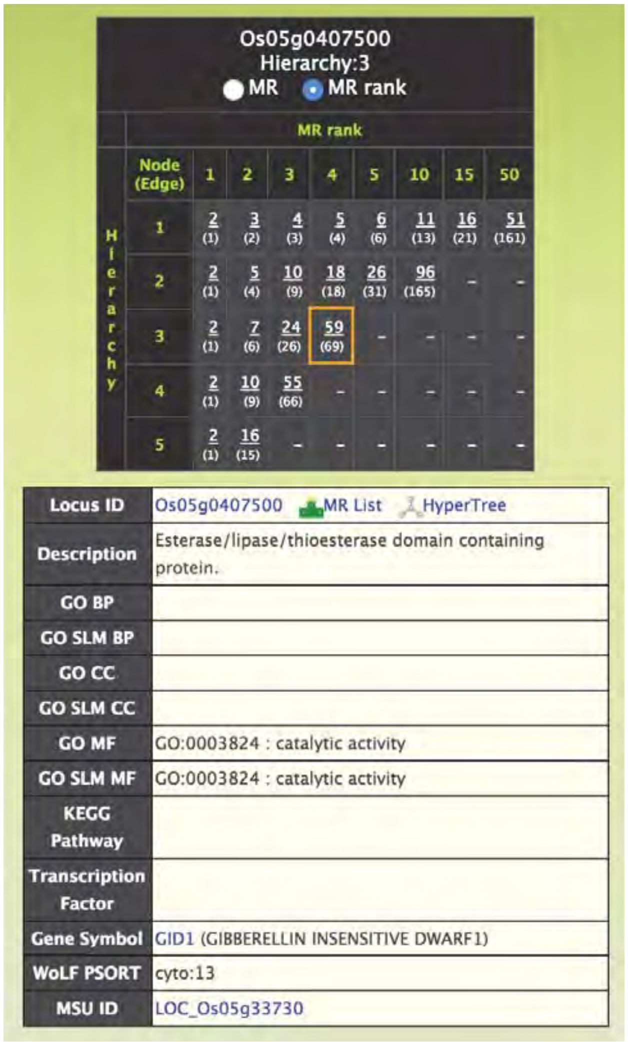

Os05g0407500
Hierarchy:3
MR MR rank

Hierarchy	Node (Edge)	MR rank 1	2	3	4	5	10	15	50
	1	2 (1)	3 (2)	4 (3)	5 (4)	6 (6)	11 (13)	16 (21)	51 (161)
	2	2 (1)	5 (4)	10 (9)	18 (18)	26 (31)	96 (165)	-	-
	3	2 (1)	7 (6)	24 (26)	59 (69)	-	-	-	-
	4	2 (1)	10 (9)	55 (66)	-	-	-	-	-
	5	2 (1)	16 (15)	-	-	-	-	-	-

Locus ID	Os05g0407500 MR List HyperTree
Description	Esterase/lipase/thioesterase domain containing protein.
GO BP	
GO SLM BP	
GO CC	
GO SLM CC	
GO MF	GO:0003824 : catalytic activity
GO SLM MF	GO:0003824 : catalytic activity
KEGG Pathway	
Transcription Factor	
Gene Symbol	GID1 (GIBBERELLIN INSENSITIVE DWARF1)
WoLF PSORT	cyto:13
MSU ID	LOC_Os05g33730

Supplementary Figure 78. Coexpression network for *GIBBERELLIN INSENSITIVE DWARF 1*. The gene coexpression network was constructed on the RiceFREND database (http://ricefrend.dna.affrc.go.jp/) using *GID1* as a single gene guide.

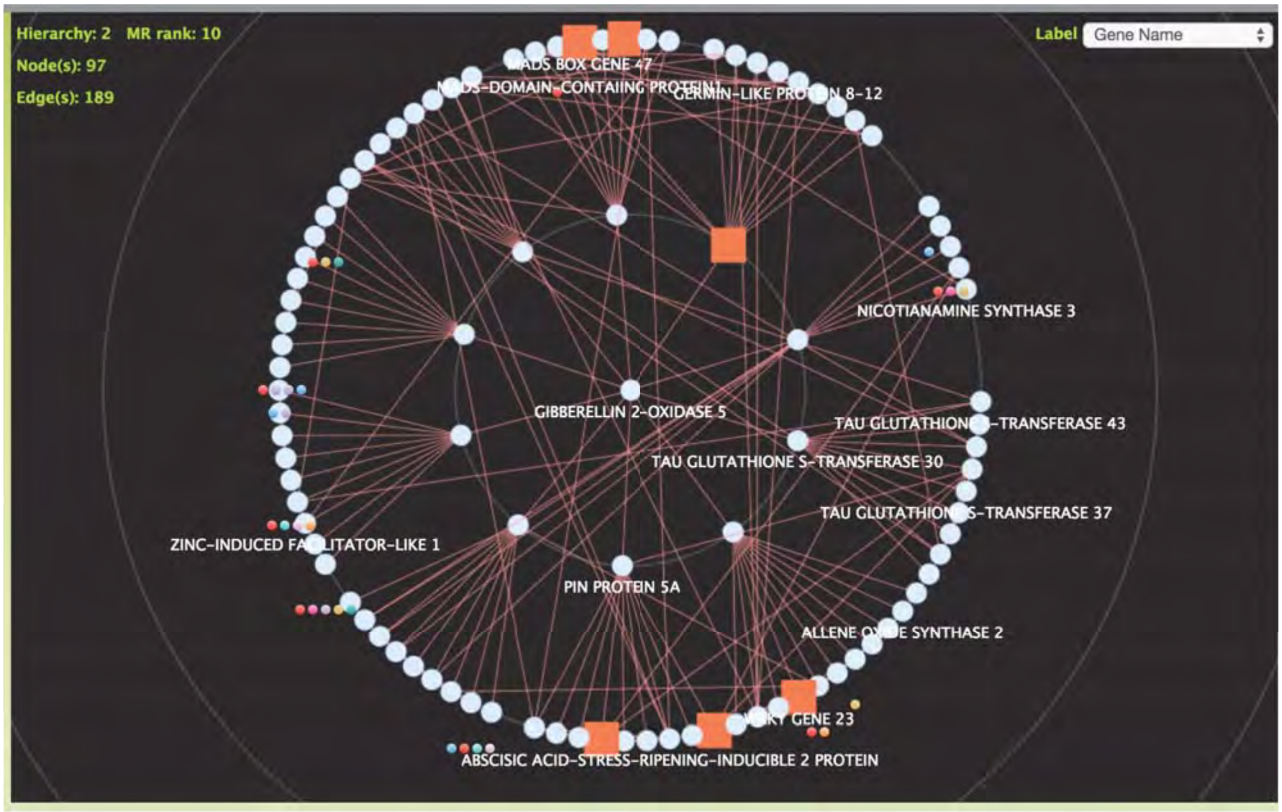

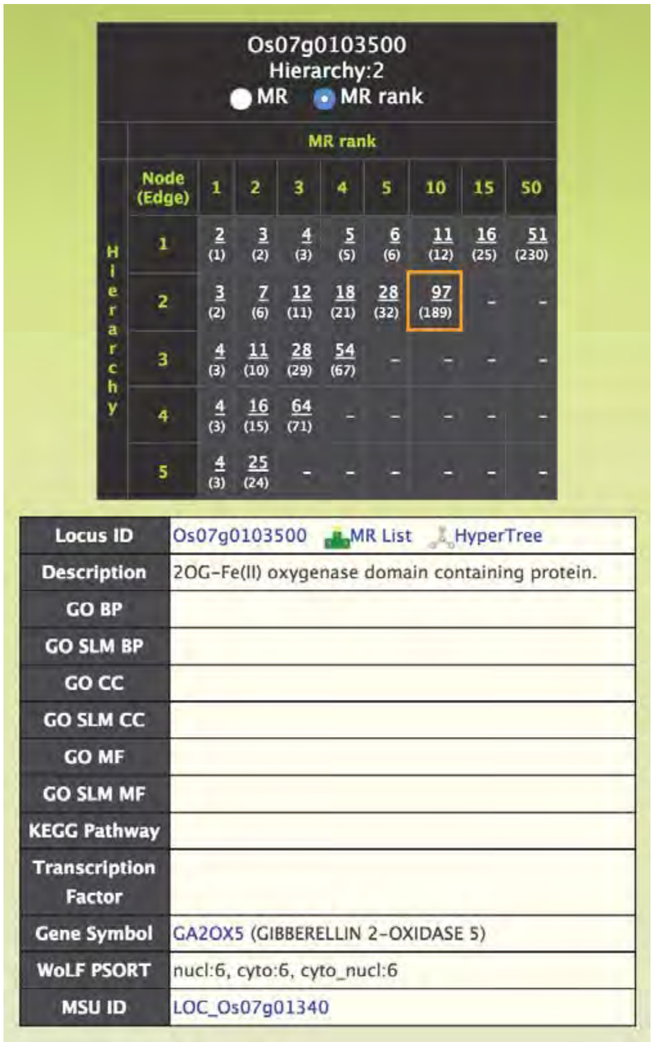

Os07g0103500
Hierarchy:2
MR MR rank

Hierarchy	Node (Edge)	MR rank 1	2	3	4	5	10	15	50
	1	2 (1)	3 (2)	4 (3)	5 (5)	6 (6)	11 (12)	16 (25)	51 (230)
	2	3 (2)	7 (6)	12 (11)	18 (21)	28 (32)	97 (189)	-	-
	3	4 (3)	11 (10)	28 (29)	54 (67)	-	-	-	-
	4	4 (3)	16 (15)	64 (71)	-	-	-	-	-
	5	4 (3)	25 (24)	-	-	-	-	-	-

Locus ID	Os07g0103500 MR List HyperTree
Description	2OG-Fe(II) oxygenase domain containing protein.
GO BP	
GO SLM BP	
GO CC	
GO SLM CC	
GO MF	
GO SLM MF	
KEGG Pathway	
Transcription Factor	
Gene Symbol	GA2OX5 (GIBBERELLIN 2-OXIDASE 5)
WoLF PSORT	nucl:6, cyto:6, cyto_nucl:6
MSU ID	LOC_Os07g01340

Supplementary Figure 79. Coexpression network for *gibberellin oxidase OsGA2ox-5*. The gene coexpression network was constructed on the RiceFREND database (http://ricefrend.dna.affrc.go.jp/) using *OsGA2ox-5* as a single gene guide.

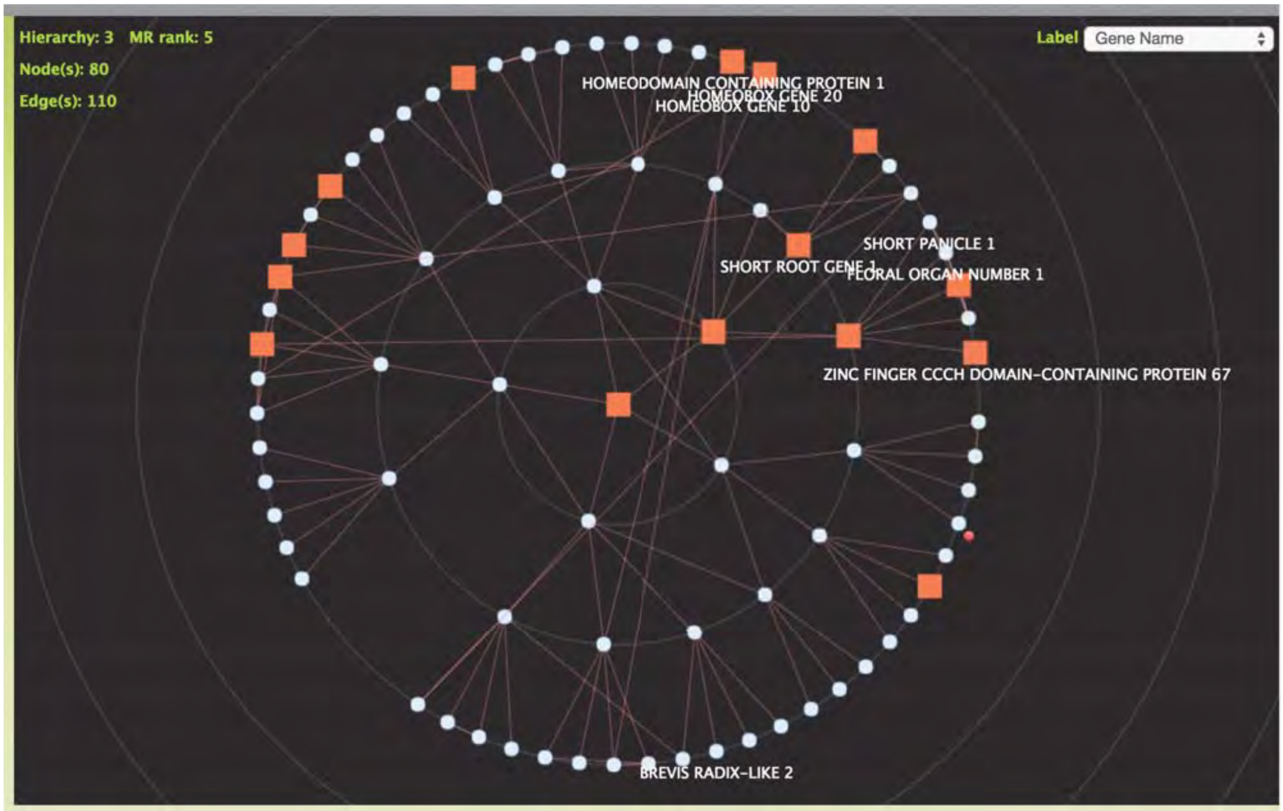

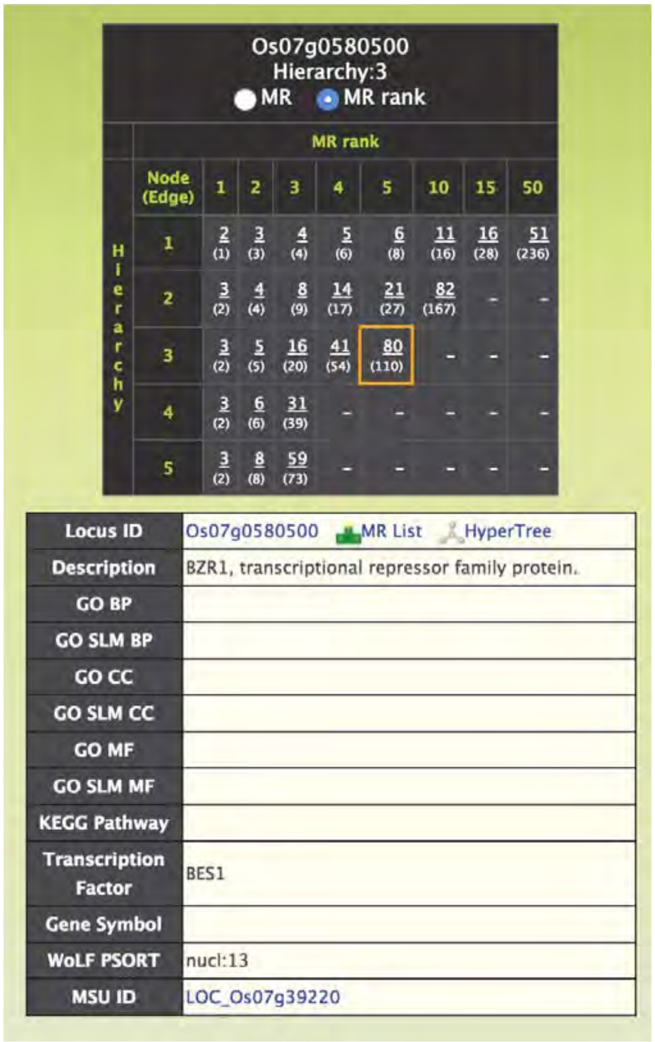

Os07g0580500
Hierarchy:3
MR MR rank

Hierarchy	Node (Edge) MR rank: 1	2	3	4	5	10	15	50
1	2 (1)	3 (3)	4 (4)	5 (6)	6 (8)	11 (16)	16 (28)	51 (236)
2	3 (2)	4 (4)	8 (9)	14 (17)	21 (27)	82 (167)	-	-
3	3 (2)	5 (5)	16 (20)	41 (54)	80 (110)	-	-	-
4	3 (2)	6 (6)	31 (39)	-	-	-	-	-
5	3 (2)	8 (8)	59 (73)	-	-	-	-	-

Locus ID	Os07g0580500 MR List HyperTree
Description	BZR1, transcriptional repressor family protein.
GO BP	
GO SLM BP	
GO CC	
GO SLM CC	
GO MF	
GO SLM MF	
KEGG Pathway	
Transcription Factor	BES1
Gene Symbol	
WoLF PSORT	nucl:13
MSU ID	LOC_Os07g39220

Supplementary Figure 80. Coexpression network for *BRASSINAZOLE RESISTANT 1*. The gene coexpression network was constructed on the RiceFREND database (http://ricefrend.dna.affrc.go.jp/) using *BZR1* as a single gene guide.

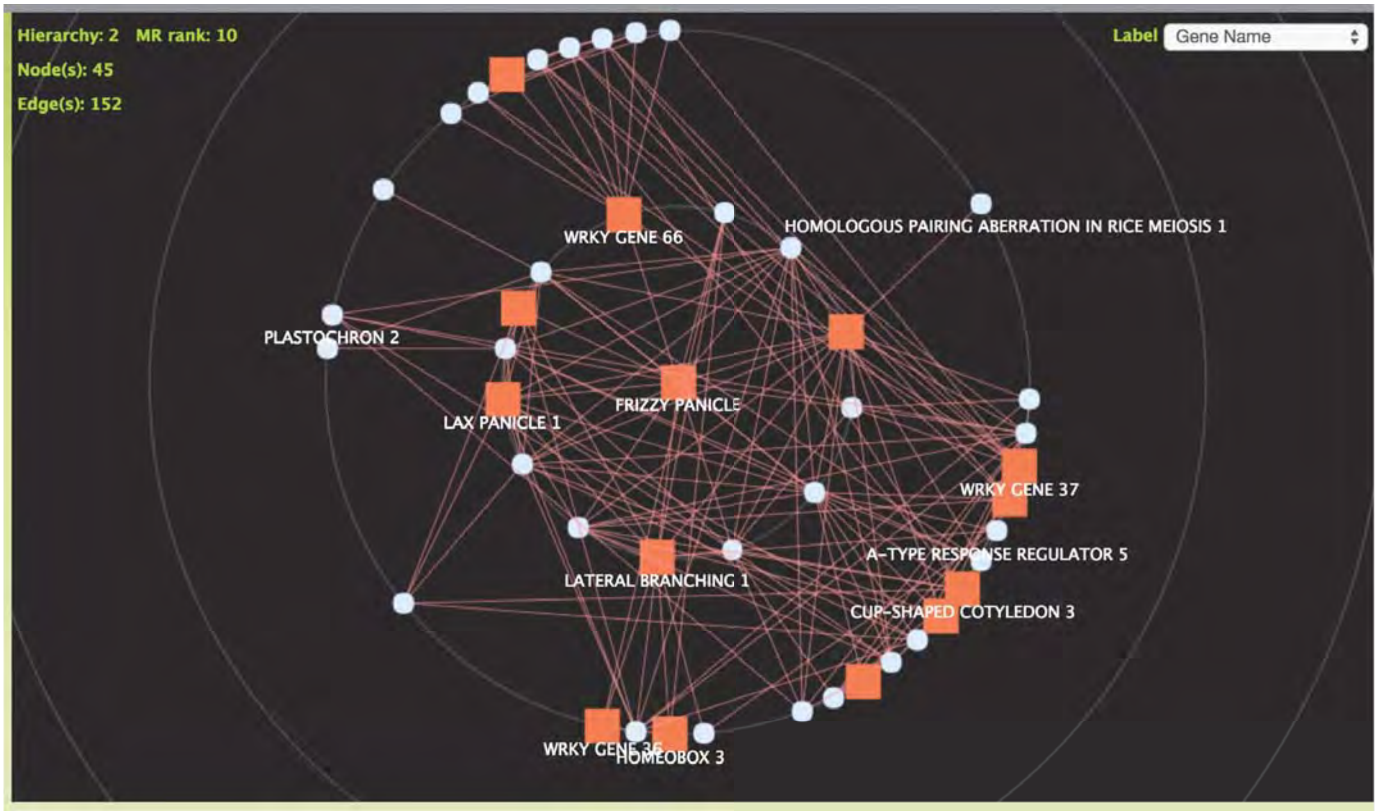

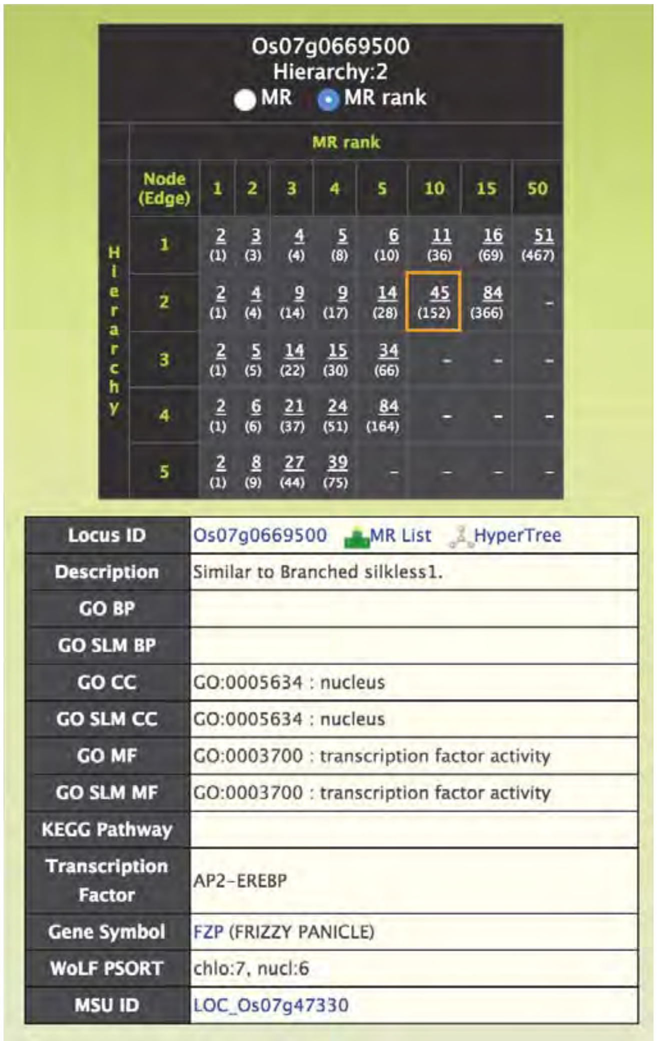

Os07g0669500
Hierarchy:2
MR   MR rank

Hierarchy	Node (Edge)	1	2	3	4	5	10	15	50
		MR rank							
1		2 (1)	3 (3)	4 (4)	5 (8)	6 (10)	11 (36)	16 (69)	51 (467)
2		2 (1)	4 (4)	9 (14)	9 (17)	14 (28)	45 (152)	84 (366)	-
3		2 (1)	5 (5)	14 (22)	15 (30)	34 (66)	-	-	-
4		2 (1)	6 (6)	21 (37)	24 (51)	84 (164)	-	-	-
5		2 (1)	8 (9)	27 (44)	39 (75)	-	-	-	-

Locus ID	Os07g0669500 MR List HyperTree
Description	Similar to Branched silkless1.
GO BP	
GO SLM BP	
GO CC	GO:0005634 : nucleus
GO SLM CC	GO:0005634 : nucleus
GO MF	GO:0003700 : transcription factor activity
GO SLM MF	GO:0003700 : transcription factor activity
KEGG Pathway	
Transcription Factor	AP2-EREBP
Gene Symbol	FZP (FRIZZY PANICLE)
WoLF PSORT	chlo:7, nucl:6
MSU ID	LOC_Os07g47330

Supplementary Figure 81. Coexpression network for *FRIZZY PANICLE 1.* The gene coexpression network was constructed on the RiceFREND database (http://ricefrend.dna.affrc.go.jp/) using *FZP1* as a single gene guide.

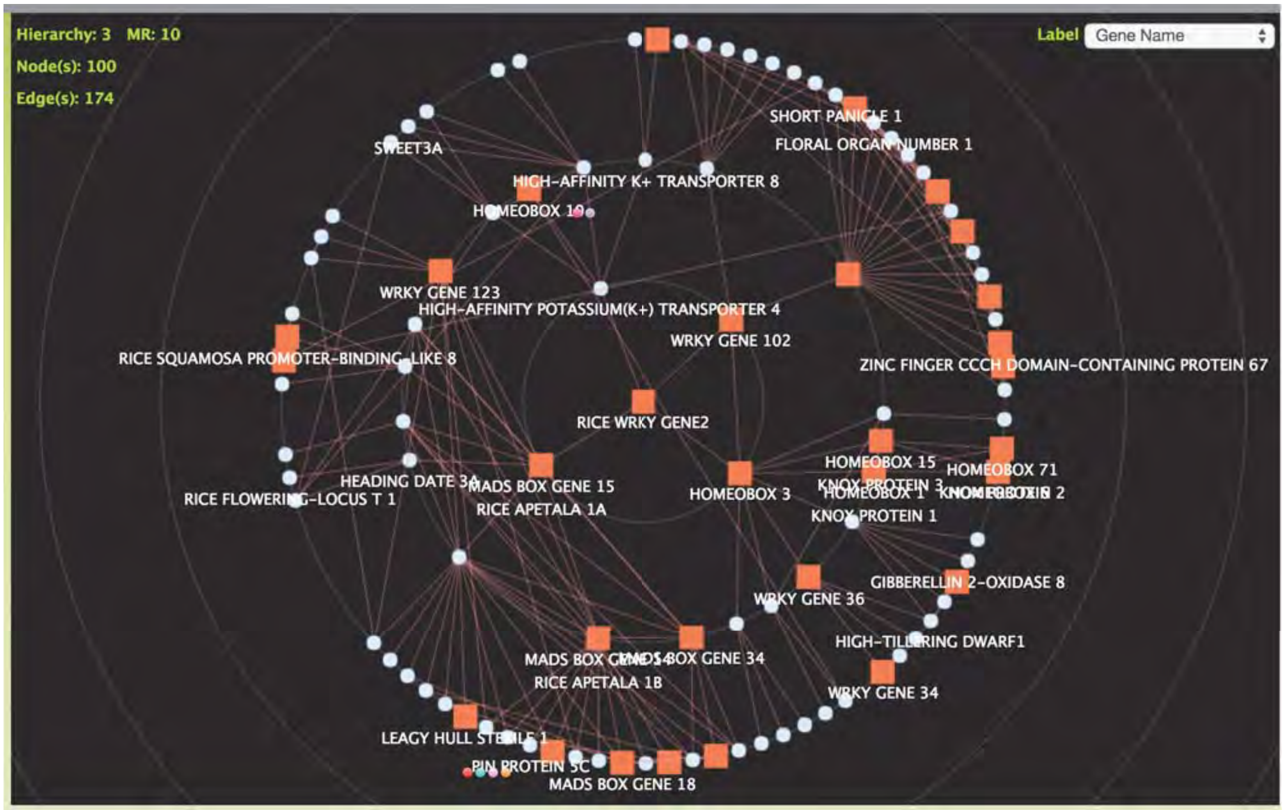

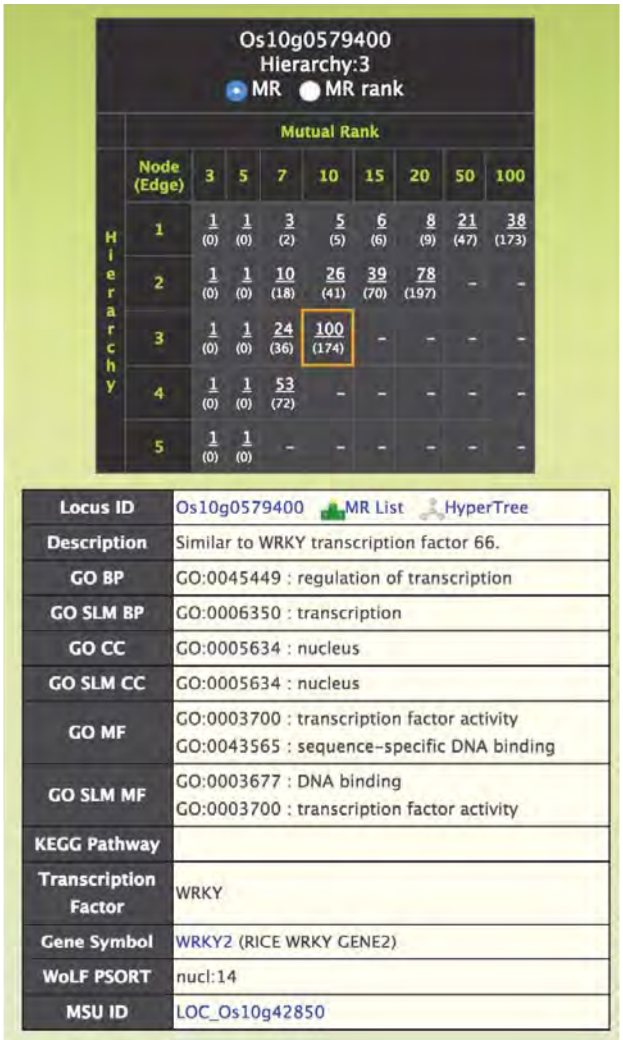

Os10g0579400
Hierarchy:3
MR MR rank

Hierarchy	Node (Edge)	3	5	7	10	15	20	50	100
		Mutual Rank							
1		1 (0)	1 (0)	3 (2)	5 (5)	6 (6)	8 (9)	21 (47)	38 (173)
2		1 (0)	1 (0)	10 (18)	26 (41)	39 (70)	78 (197)	-	-
3		1 (0)	1 (0)	24 (36)	100 (174)	-	-	-	-
4		1 (0)	1 (0)	53 (72)	-	-	-	-	-
5		1 (0)	1 (0)	-	-	-	-	-	-

Locus ID	Os10g0579400 MR List HyperTree
Description	Similar to WRKY transcription factor 66.
GO BP	GO:0045449 : regulation of transcription
GO SLM BP	GO:0006350 : transcription
GO CC	GO:0005634 : nucleus
GO SLM CC	GO:0005634 : nucleus
GO MF	GO:0003700 : transcription factor activity<br>GO:0043565 : sequence-specific DNA binding
GO SLM MF	GO:0003677 : DNA binding<br>GO:0003700 : transcription factor activity
KEGG Pathway	
Transcription Factor	WRKY
Gene Symbol	WRKY2 (RICE WRKY GENE2)
WoLF PSORT	nucl:14
MSU ID	LOC_Os10g42850

Supplementary Figure 82. Coexpression network for rice *WRKY2*. The gene coexpression network was constructed on the RiceFREND database (http://ricefrend.dna.affrc.go.jp/) using *WRKY2* as a single gene guide.

Chromosome 1

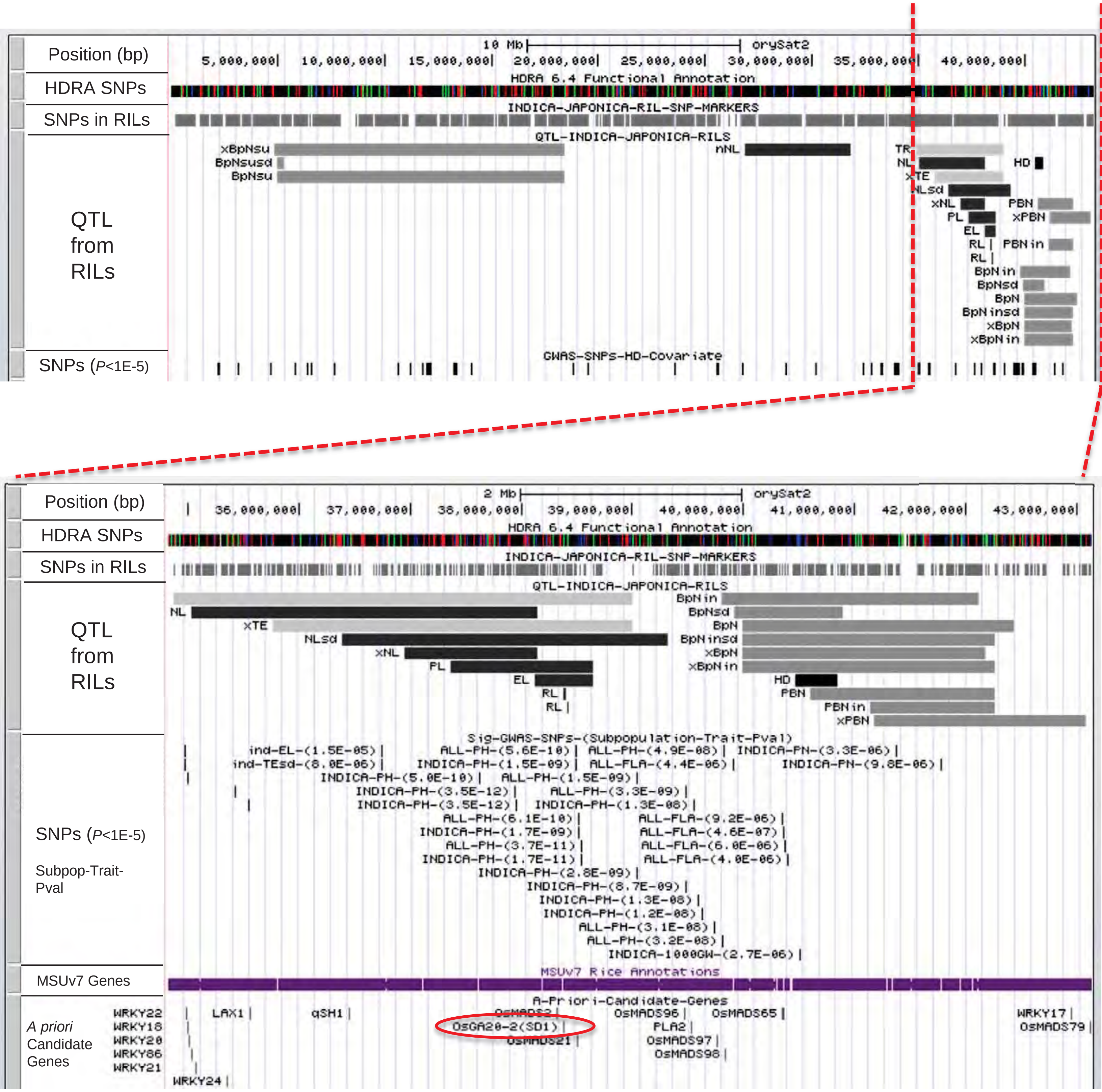

Supplementary Figure 83. Chromosome 1 GWAS and QTL results. Screenshots from the UCSC Browser with tracks depicting: high-density rice array (HDRA) SNPs; SNPs used in the recombinant inbred line (RIL) population; QTL identified for traits in the RILs; and significant SNPs associated with traits from the GWAS panel. The zoom in includes gene annotations from MSUv7, as well as *a priori* candidate genes from Supplementary Table 5. Candidates <30kb from a GWAS SNP are circled in red. Trait abbreviations correspond to Supplementary Table 2.

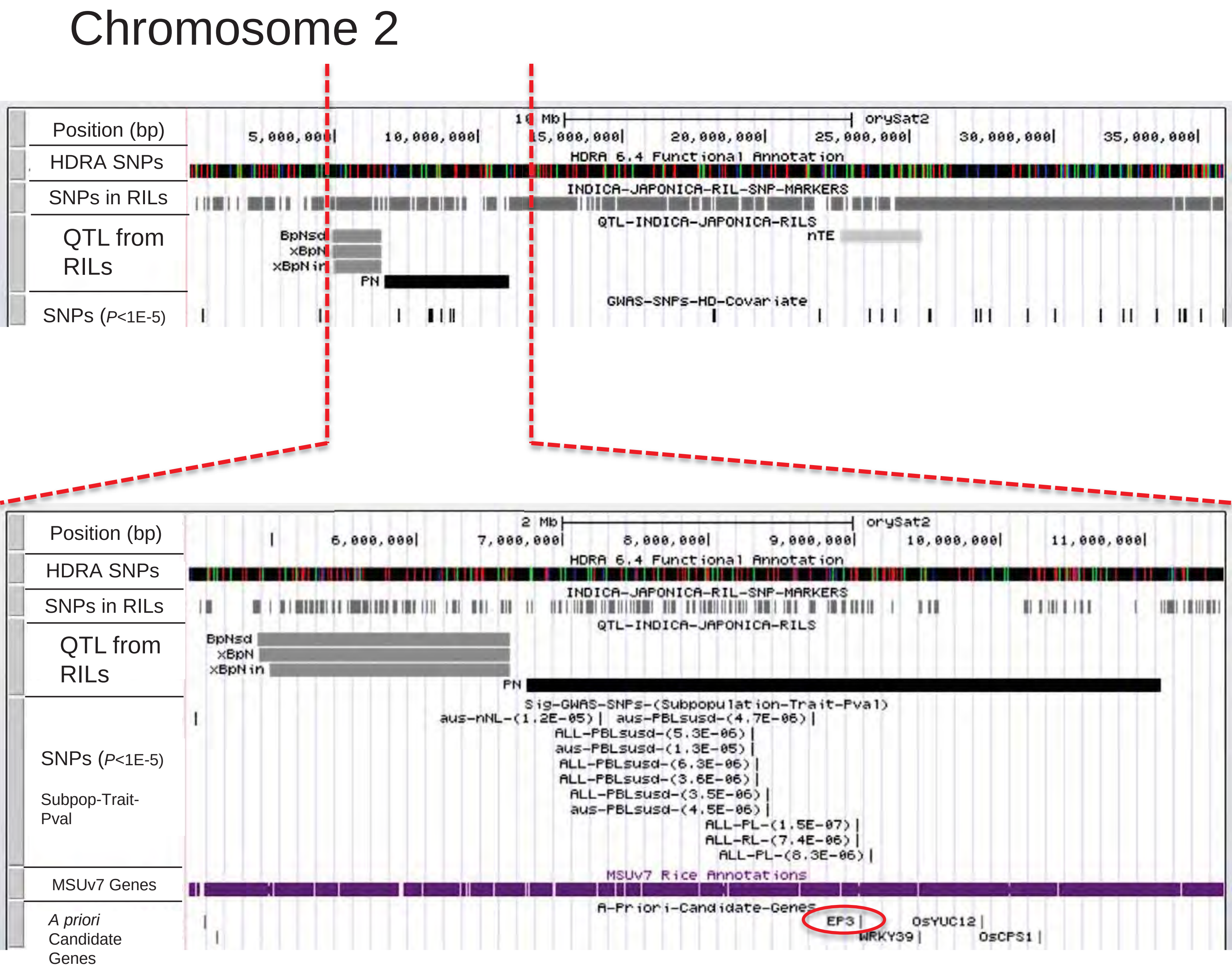

Supplementary Figure 84. Chromosome 2 GWAS and QTL results. Screenshots from the UCSC Browser with tracks depicting: high-density rice array (HDRA) SNPs; SNPs used in the recombinant inbred line (RIL) population; QTL identified for traits in the RILs; and significant SNPs associated with traits from the GWAS panel. The zoom in includes gene annotations from MSUv7, as well as *a priori* candidate genes from Supplementary Table 5. Candidates <30kb from a GWAS SNP are circled in red. Trait abbreviations correspond to Supplementary Table 2.

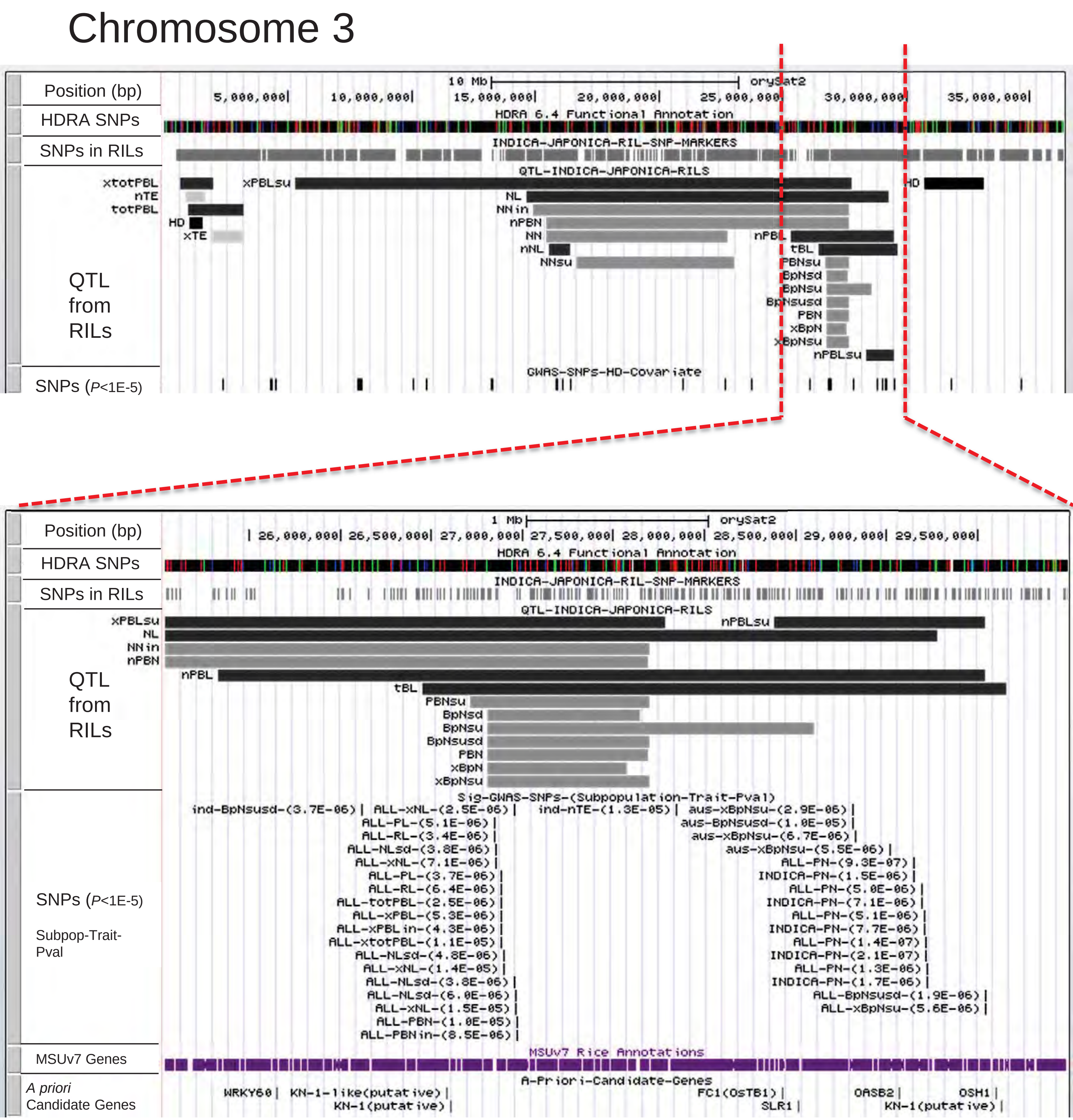

Supplementary Figure 85. Chromosome 3 GWAS and QTL results. Screenshots from the UCSC Browser with tracks depicting: high-density rice array (HDRA) SNPs; SNPs used in the recombinant inbred line (RIL) population; QTL identified for traits in the RILs; and significant SNPs associated with traits from the GWAS panel. The zoom in includes gene annotations from MSUv7, as well as *a priori* candidate genes from Supplementary Table 5. Candidates <30kb from a GWAS SNP are circled in red. Trait abbreviations correspond to Supplementary Table 2.

Chromosome 4

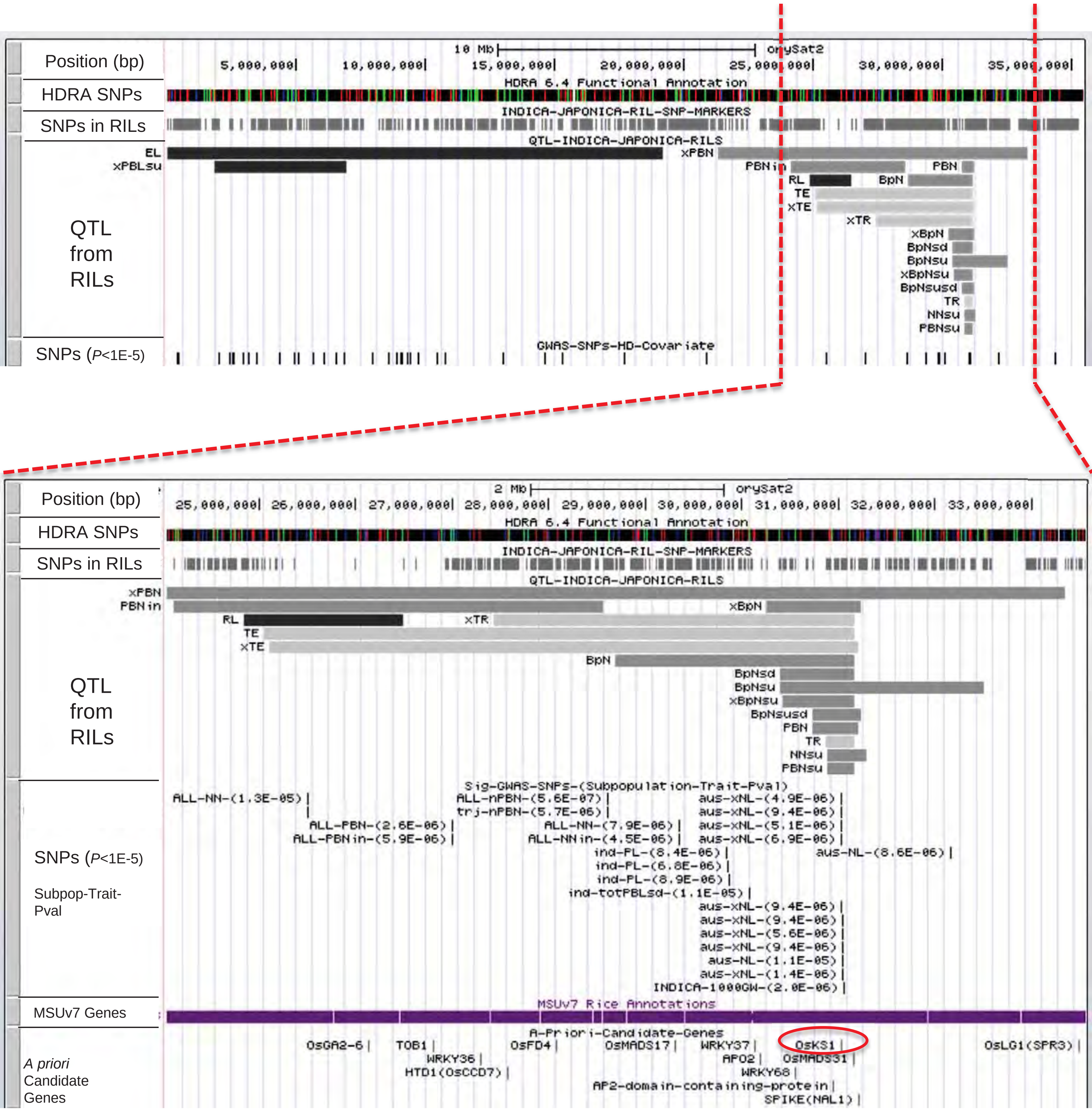

Supplementary Figure 86. Chromosome 4 GWAS and QTL results. Screenshots from the UCSC Browser with tracks depicting: high-density rice array (HDRA) SNPs; SNPs used in the recombinant inbred line (RIL) population; QTL identified for traits in the RILs; and significant SNPs associated with traits from the GWAS panel. The zoom in includes gene annotations from MSUv7, as well as *a priori* candidate genes from Supplementary Table 5. Candidates <30kb from a GWAS SNP are circled in red. Trait abbreviations correspond to Supplementary Table 2.

Chromosome 5

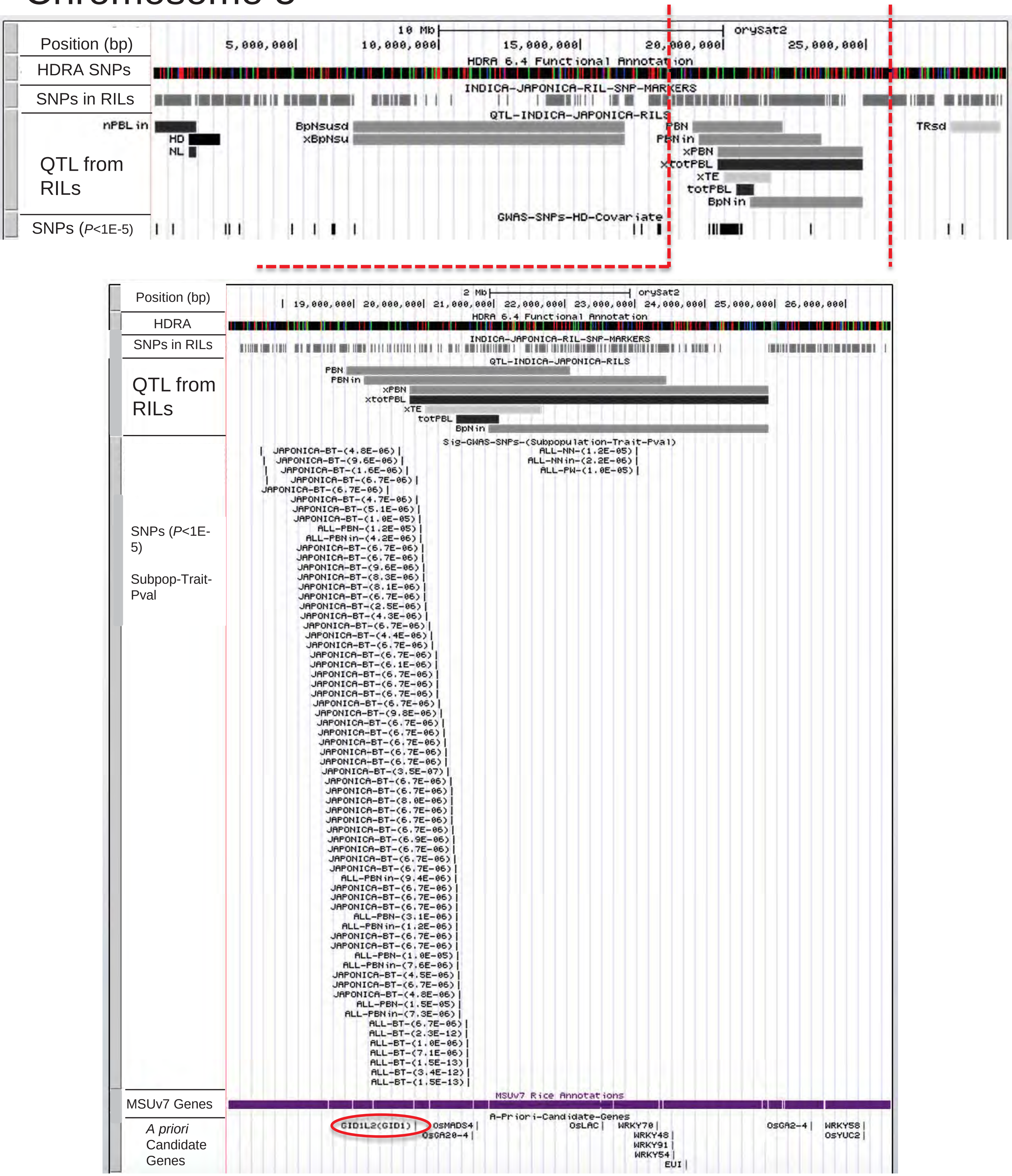

Supplementary Figure 87. Chromosome 5 GWAS and QTL results. Screenshots from the UCSC Browser with tracks depicting: high-density rice array (HDRA) SNPs; SNPs used in the recombinant inbred line (RIL) population; QTL identified for traits in the RILs; and significant SNPs associated with traits from the GWAS panel. The zoom in includes gene annotations from MSUv7, as well as *a priori* candidate genes from Supplementary Table 5. Candidates <30kb from a GWAS SNP are circled in red. Trait abbreviations correspond to Supplementary Table 2.

Chromosome 6

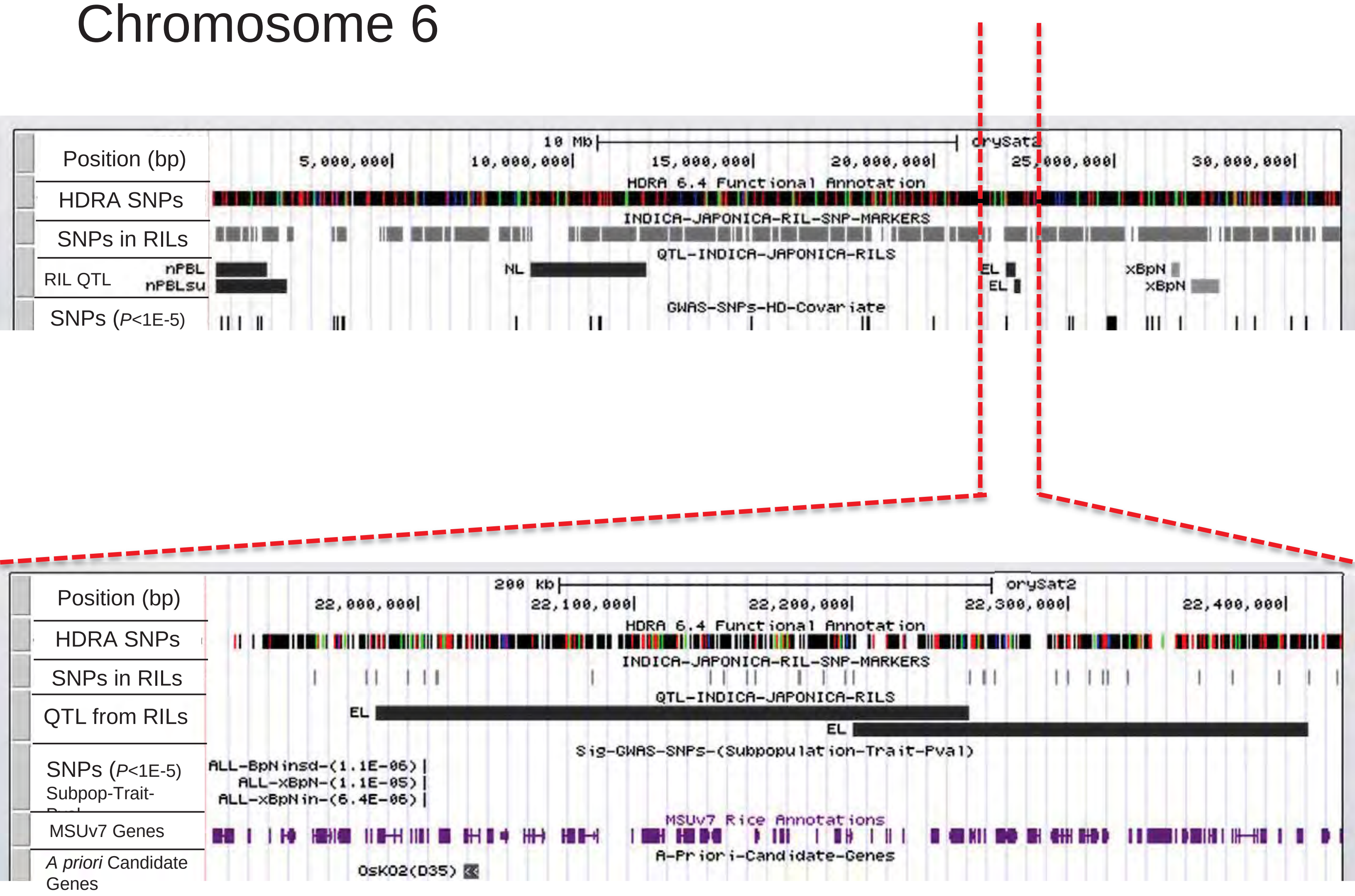

Supplementary Figure 88. Chromosome 6 GWAS and QTL results. Screenshots from the UCSC Browser with tracks depicting: high-density rice array (HDRA) SNPs; SNPs used in the recombinant inbred line (RIL) population; QTL identified for traits in the RILs; and significant SNPs associated with traits from the GWAS panel. The zoom in includes gene annotations from MSUv7, as well as *a priori* candidate genes from Supplementary Table 5. Candidates <30kb from a GWAS SNP are circled in red. Trait abbreviations correspond to Supplementary Table 2.

Chromosome 7

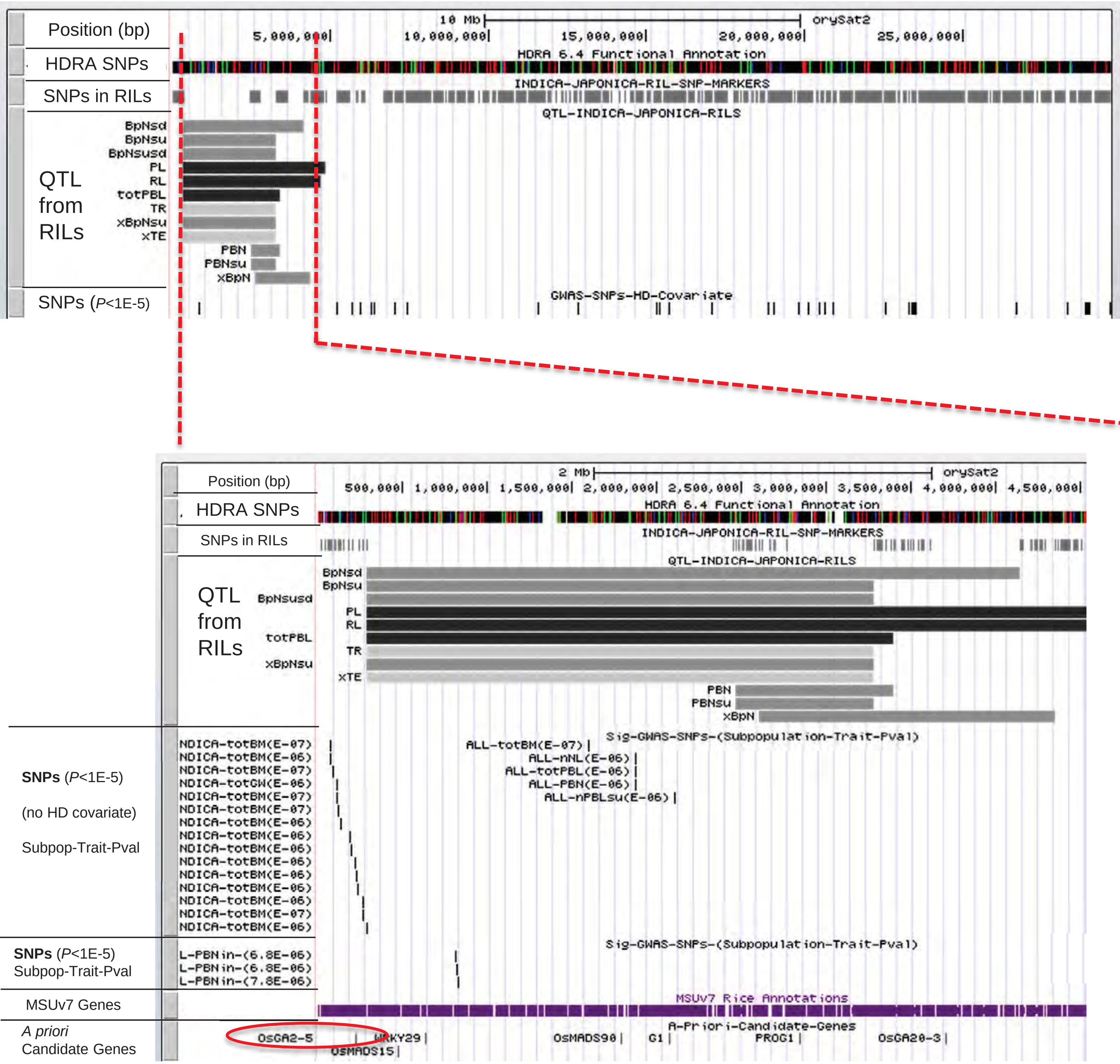

Supplementary Figure 89. Chromosome 7 GWAS and QTL results. Screenshots from the UCSC Browser with tracks depicting: high-density rice array (HDRA) SNPs; SNPs used in the recombinant inbred line (RIL) population; QTL identified for traits in the RILs; and significant SNPs associated with traits from the GWAS panel. The zoom in includes gene annotations from MSUv7, as well as *a priori* candidate genes from Supplementary Table 5. Candidates <30kb from a GWAS SNP are circled in red. Trait abbreviations correspond to Supplementary Table 2.

Chromosome 8

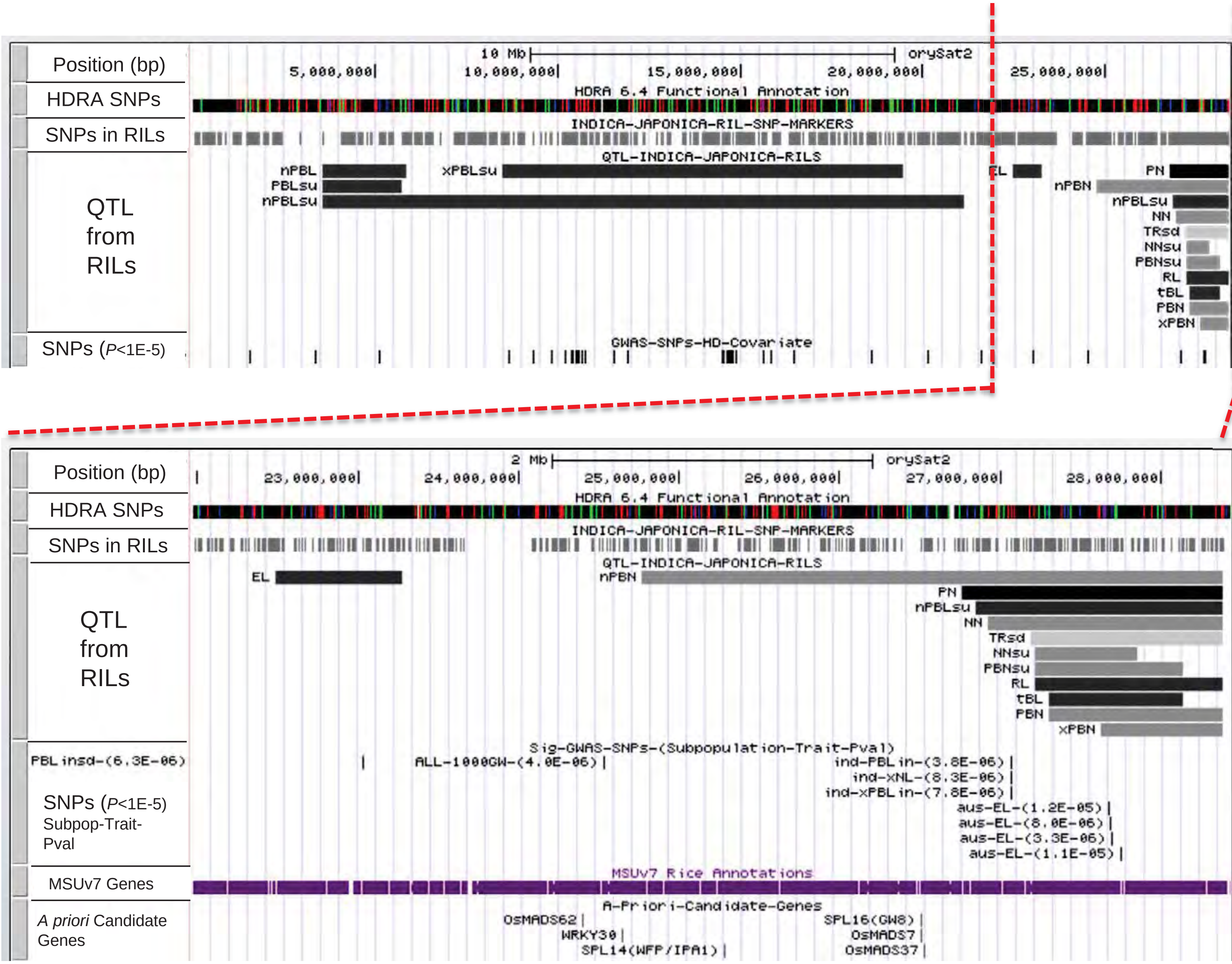

Supplementary Figure 90. Chromosome 8 GWAS and QTL results. Screenshots from the UCSC Browser with tracks depicting: high-density rice array (HDRA) SNPs; SNPs used in the recombinant inbred line (RIL) population; QTL identified for traits in the RILs; and significant SNPs associated with traits from the GWAS panel. The zoom in includes gene annotations from MSUv7, as well as *a priori* candidate genes from Supplementary Table 5. Candidates <30kb from a GWAS SNP are circled in red. Trait abbreviations correspond to Supplementary Table 2.

Chromosome 9

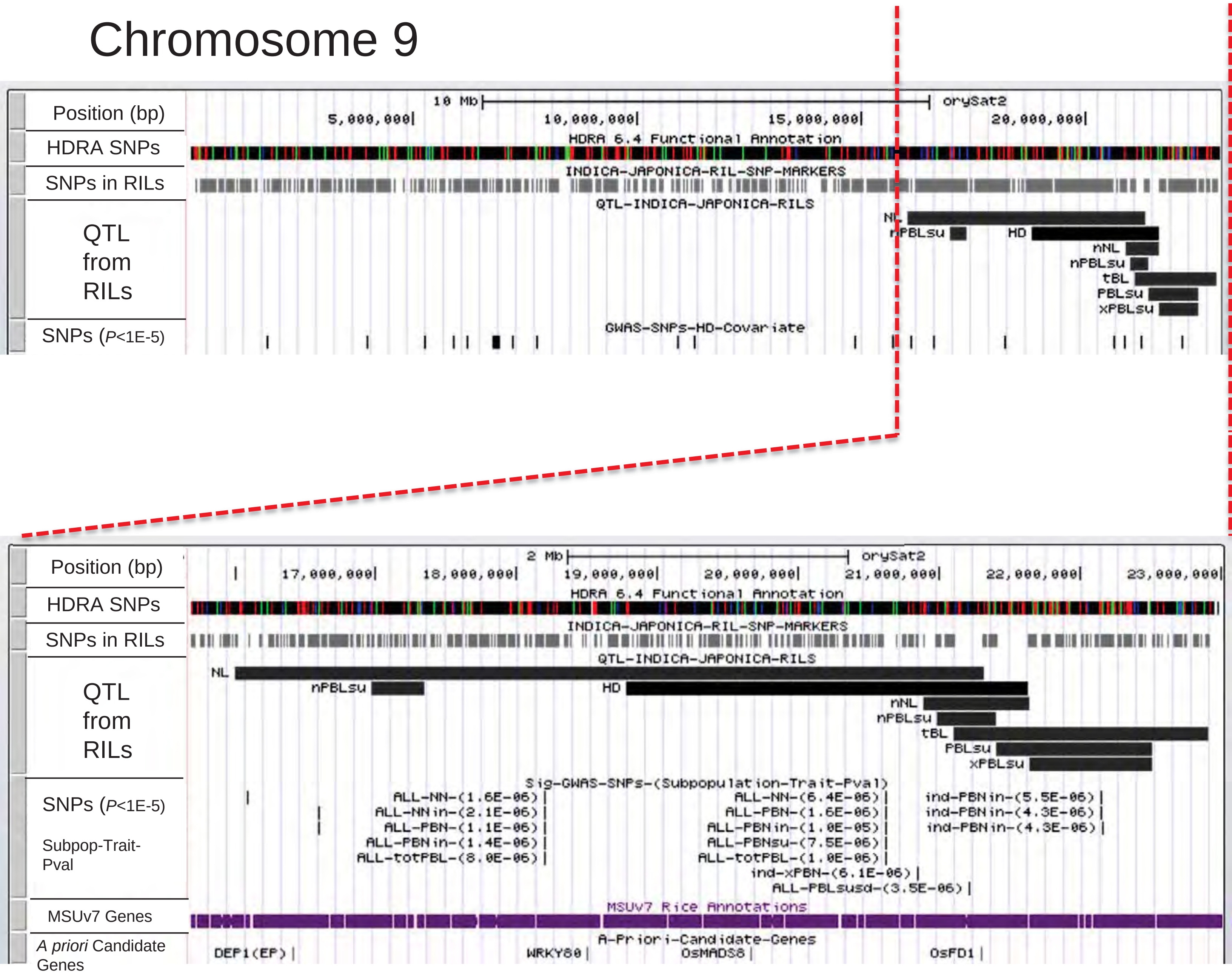

Supplementary Figure 91. Chromosome 9 GWAS and QTL results. Screenshots from the UCSC Browser with tracks depicting: high-density rice array (HDRA) SNPs; SNPs used in the recombinant inbred line (RIL) population; QTL identified for traits in the RILs; and significant SNPs associated with traits from the GWAS panel. The zoom in includes gene annotations from MSUv7, as well as *a priori* candidate genes from Supplementary Table 5. Candidates <30kb from a GWAS SNP are circled in red. Trait abbreviations correspond to Supplementary Table 2.

Chromosome 10

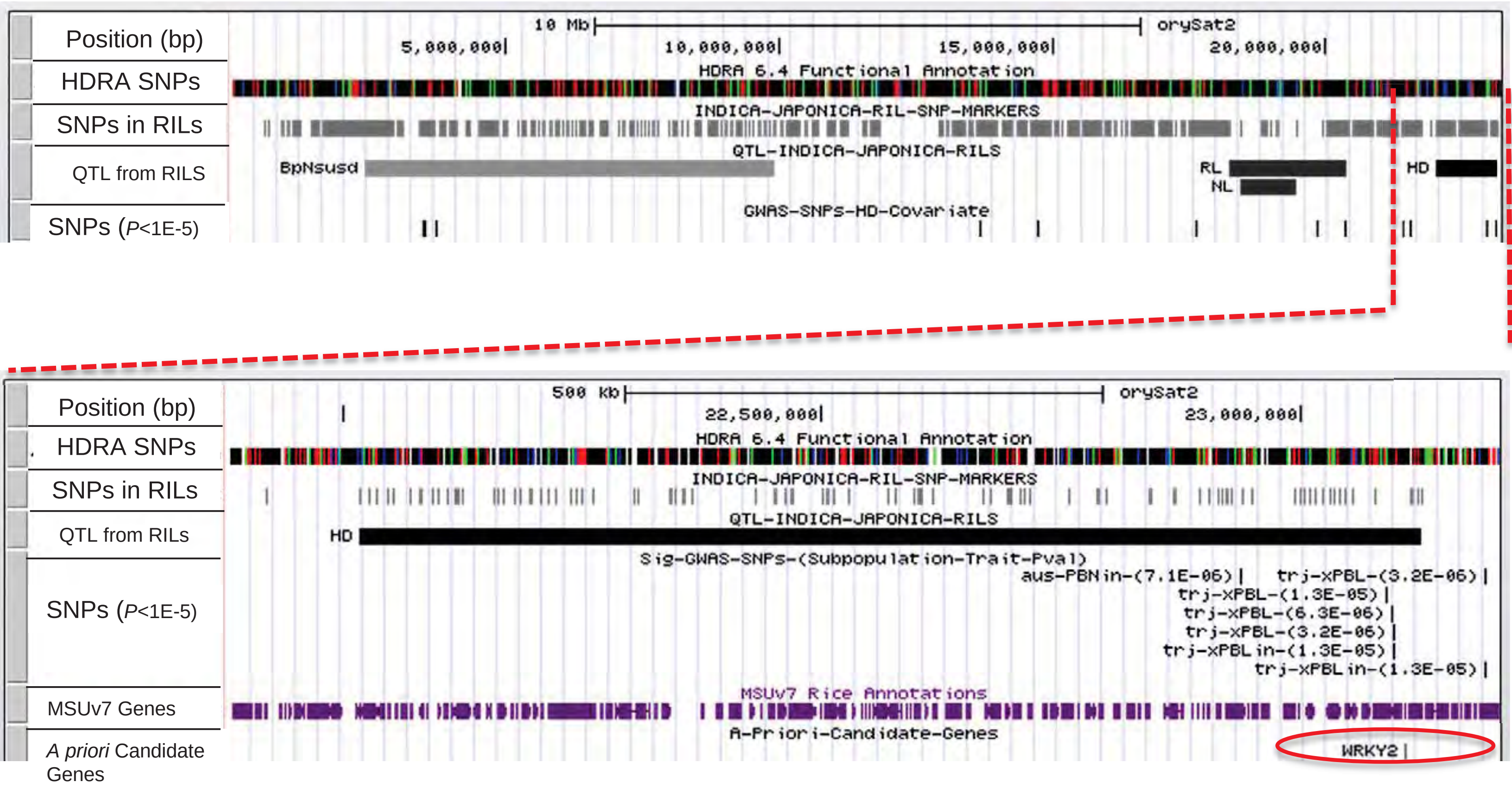

Supplementary Figure 92. Chromosome 10 GWAS and QTL results. Screenshots from the UCSC Browser with tracks depicting: high-density rice array (HDRA) SNPs; SNPs used in the recombinant inbred line (RIL) population; QTL identified for traits in the RILs; and significant SNPs associated with traits from the GWAS panel. The zoom in includes gene annotations from MSUv7, as well as *a priori* candidate genes from Supplementary Table 5. Candidates <30kb from a GWAS SNP are circled in red. Trait abbreviations correspond to Supplementary Table 2.

Chromosome 11

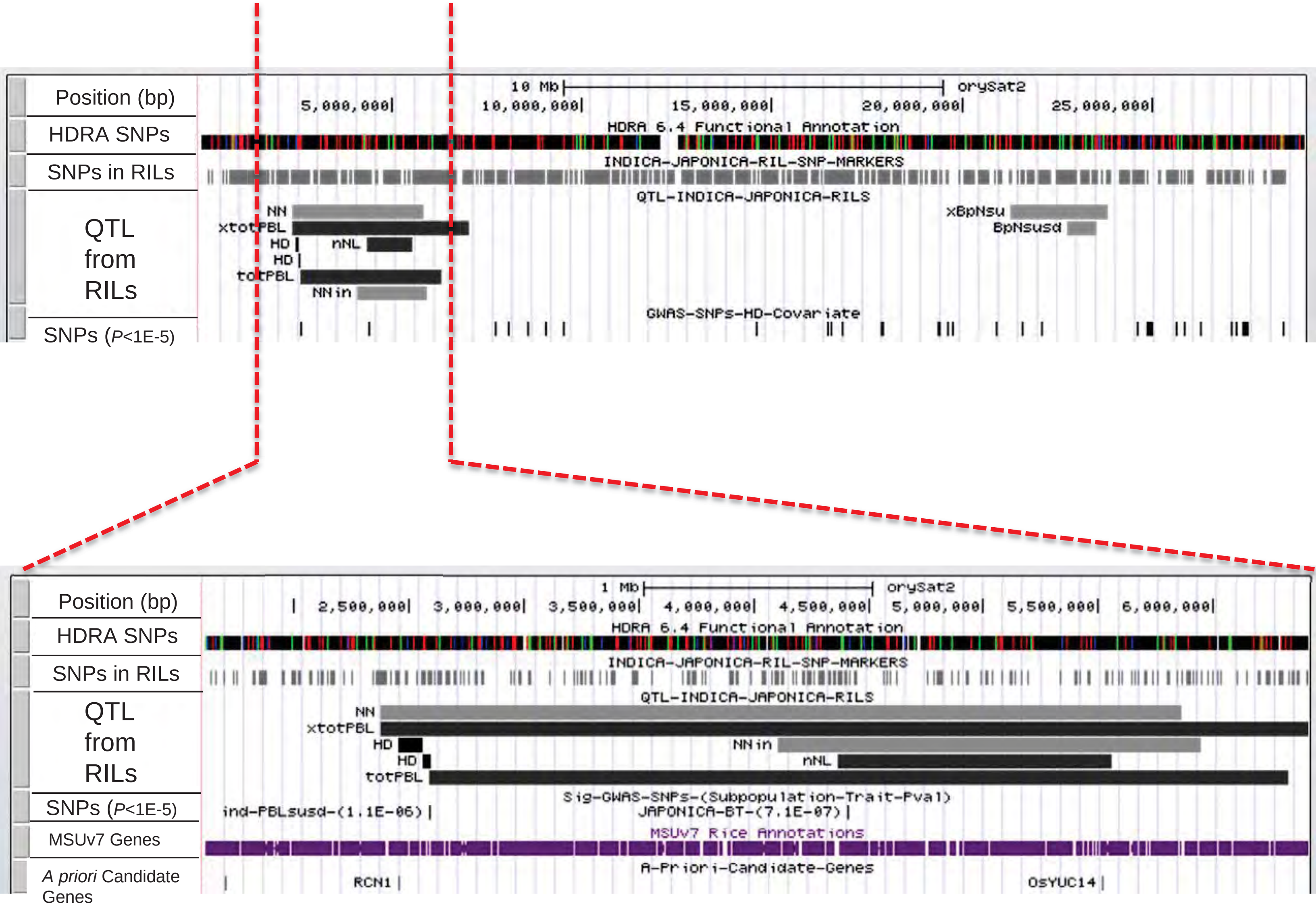

Supplementary Figure 93. Chromosome 11 GWAS and QTL results. Screenshots from the UCSC Browser with tracks depicting: high-density rice array (HDRA) SNPs; SNPs used in the recombinant inbred line (RIL) population; QTL identified for traits in the RILs; and significant SNPs associated with traits from the GWAS panel. The zoom in includes gene annotations from MSUv7, as well as *a priori* candidate genes from Supplementary Table 5. Candidates <30kb from a GWAS SNP are circled in red. Trait abbreviations correspond to Supplementary Table 2.

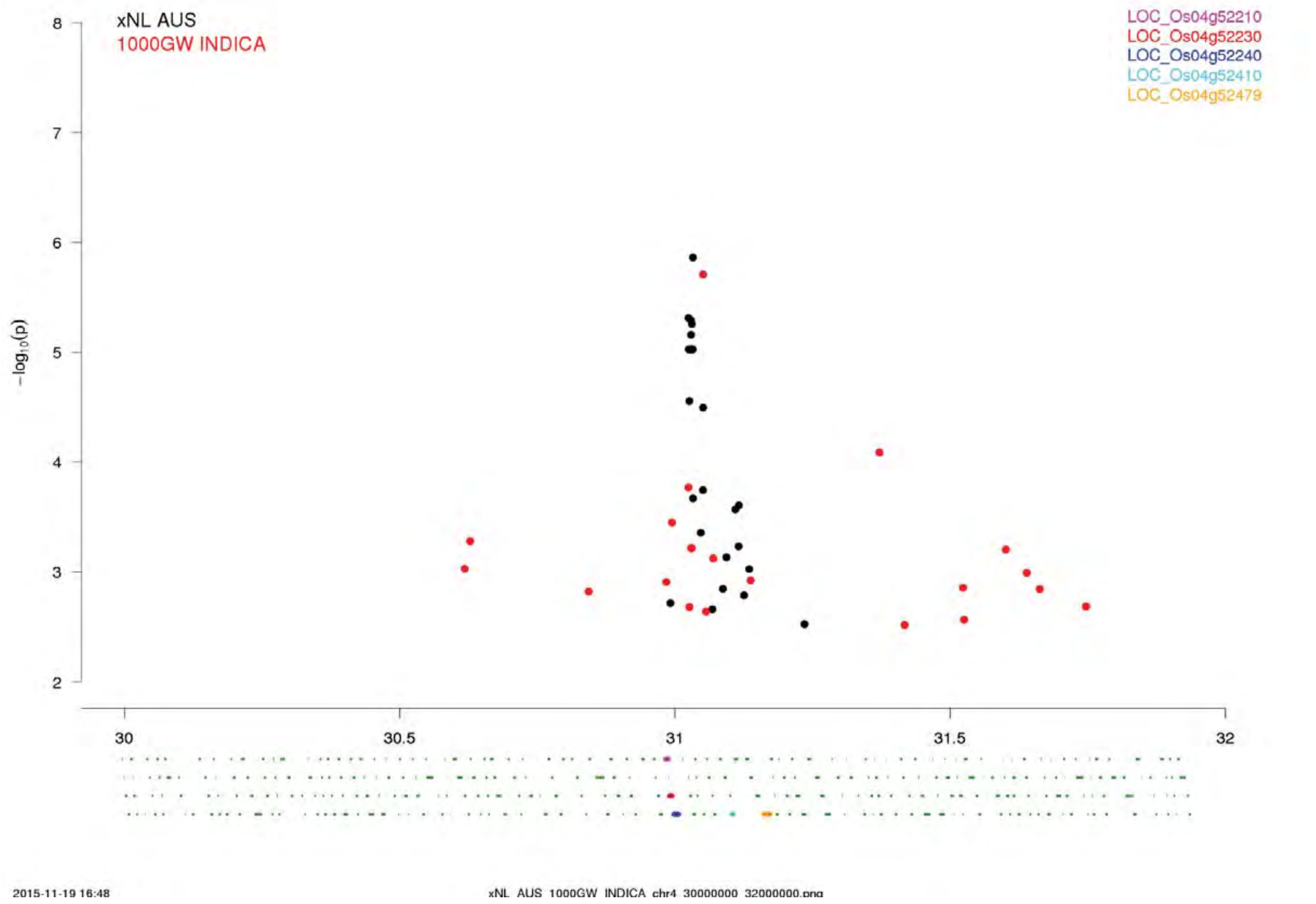

Supplementary Figure 94: Composite Manhattan Plot for xNL and 1000GW on Chromosome 4. Composite Manhattan Plot showing position of GWAS-QTL associated with maximum internode length (xNL) detected in the *aus* subpopulation (black) and 1000 grain weight (GW) detected in the *Indica* varietal group (*indica + aus*) (red) across a 2 Mb region between 30-32 Mb on chromosome 4; five candidate genes (*OsKS3*= LOC_Os04g52210, *OsKS1*= LOC_Os04g52230, *OsKS2*= LOC_Os04g52240, *OsMADS31*= LOC_Os04g52410, and *NAL1*= LOC_Os04g52479*)* are highlighted as colored bars below the Manhattan plot; the candidate genes fall within the mega-locus where these two GWAS-QTL overlap with 14 NIL-QTLs (shown in Fig. 7).

Supplementary Tables

Supplementary Table 1. Germplasm comprising the GWAS diversity panel.

GSaccno	HDRA_Sample_ID	IRGC_Name	IRGC	Origin Country	Subpopulation	Subspecies	Varieties with yield component data
122128	122128	Kakani 2	KAKANI 2	NPL	Admixed	-	y
121493	121493	Sampog	SAMPOG	MYS	Admixed	-	
121450	121450	Neng Nah	NENG NAH	THA	Admixed-Japonica	Japonica	y
121469	121469	Peek	Peek	LAO	Admixed-Japonica	Japonica	y
122004	122004	Chay Loy Oe	CHA LOY OE	THA	Admixed-Japonica	Japonica	y
121208	121208	Khao Do Ngoi	Khao do ngoi	LAO	Admixed-Japonica	Japonica	
121386	121386	Khao Mum	KHAO' MUM	THA	Admixed-Japonica	Japonica	
121405	121405	Lai	LAI	THA	Admixed-Japonica	Japonica	
121444	121444	Na Gui Zhan Gu	NA GUI ZHAN GU	CHN	Admixed-Japonica	Japonica	
121498	121498	Seng	SENG	THA	Admixed-Japonica	Japonica	
121985	121985	Boda 148-3	BODA 148-3	MAD	Admixed-Japonica	Japonica	
122130	122130	Karasukara Surankasu	KARASUKARA SURANKASU	TWN	Admixed-Japonica	Japonica	
122281	122281	Vietnam 1	VIETNAM 1	VNM	Admixed-Japonica	Japonica	
117266	NSFTV651	Dular	Dular	IND	aus	Indica	y
117605	NSFTV13	Basmati 1	Basmati 1	PAK	aus	Indica	y
117617	NSFTV85	Kasalath	Kasalath	IND	aus	Indica	y
117620	NSFTV105	Mehr	Mehr	IRN	aus	Indica	y
117661	NSFTV18	BJ1	BJ1	IND	aus	Indica	y
117673	NSFTV228	CA_902.B.2.1	CA_902.B.2.1	TCD	aus	Indica	y
117692	NSFTV370	Coarse	Coarse	PAK	aus	Indica	y
117697	NSFTV314	CTG 1516	CTG_1516	BGD	aus	Indica	y
117704	NSFTV316	DD 62	DD 62	BGD	aus	Indica	y
117711	NSFTV317	DJ 123	DJ123	BGD	aus	Indica	y
117715	NSFTV320	DM 43	DM 43	BGD	aus	Indica	y
117726	NSFTV345	DZ 193	DZ_193	BGD	aus	Indica	y
117740	NSFTV58	Ghati Kamma Nangarhar	Ghati_Kamma_Nangarhar	AFG	aus	Indica	y
117741	NSFTV326	Ghorbhai	Ghorbhai	BGD	aus	Indica	y

GSaccno	HDRA_Sample_ID	IRGC_Name	IRGC	Origin Country	Subpopulation	Subspecies	Varieties with yield component data
117743	NSFTV327	Goria	Goria	BGD	aus	Indica	y
117747	NSFTV262	Halwa Gose Red	Halwa Gose Red	IRQ	aus	Indica	y
117765	NSFTV328	Jamir	Jamir	BGD	aus	Indica	y
117769	NSFTV78	Jhona 349	Jhona349	IND	aus	Indica	y
117772	NSFTV329	Kachilon	Kachilon	BGD	aus	Indica	y
117773	NSFTV81	Kalamkati	Kalamkati	IND	aus	Indica	y
117778	NSFTV346	Karkati 87	Karkati_87	BGD	aus	Indica	y
117783	NSFTV331	Khao Tot Long 227	Khao Tot Long 227	THA	aus	Indica	y
117847	NSFTV336	Paung Malaung	Paung_Malaung	BUR	aus	Indica	y
117850	NSFTV131	Phudugey	Phudugey NSFTV131	BTN	aus	Indica	y
117856	NSFTV360	PTB 30	PTB_30 NSFTV360	IND	aus	Indica	y
117878	NSFTV369	Sathi	Sathi	PAK	aus	Indica	y
117885	NSFTV341	Shirkati	Shirkati	AFG	aus	Indica	y
117899	NSFTV359	Surjamkuhi	Surjamkuhi	IND	aus	Indica	y
117903	NSFTV153	T 26	T26	IND	aus	Indica	y
120896	120896	Biranj	BIRANJ	IND	aus	Indica	y
121013	121013	Kasapur	KASAPUR	IND	aus	Indica	y
121078	121078	Pankhiraj	PANKHIRAJ	BGD	aus	Indica	y
121114	121114	Seenetti	SEENETTI	LKA	aus	Indica	y
121155	121155	Weda Heenati	WEDA HEENATI	LKA	aus	Indica	y
120864	120864	Angalais	ANGALAIS	SEN	aus	Indica	
120920	120920	Cross 4-244	CROSS 4-244	IND	aus	Indica	
120931	120931	Dhala Bagdar 2	DHALA BAGDAR 2	BGD	aus	Indica	
121017	121017	Kele Bardhan	KELE BARDHAN	IND	aus	Indica	
121026	121026	Koyra	KOYRA	BGD	aus	Indica	
121060	121060	Moyna Moti	MOYNA MOTI	BGD	aus	Indica	
121335	121335	Etym	ETYM	SUN	aus	Indica	
117902	NSFTV152	T 1	T 1	IND	aus	Indica	
117602	NSFTV178	ARC 6578	ARC_6578	IND	aus	Indica	

GSaccno	HDRA_Sample_ID	IRGC_Name	IRGC	Origin Country	Subpopulation	Subspecies	Varieties with yield component data
117662	NSFTV19	Black Gora	Black_Gora	IND	aus	Indica	
117834	NSFTV200	P737	P 737	PAK	aus	Indica	
117877	NSFTV246	Saraya	Saraya	FJI	aus	Indica	
117883	NSFTV261	Shim Balte	Shim_Balte	IRQ	aus	Indica	
117779	NSFTV276	Kaukau	Kaukau	MLI	aus	Indica	
117712	NSFTV318	DJ 24	DJ_24	BGD	aus	Indica	
117714	NSFTV319	DK 12	DK 12	BGD	aus	Indica	
117716	NSFTV321	DM 56	DM 56	BGD	aus	Indica	
117717	NSFTV322	DM 59	DM_59	BGD	aus	Indica	
117718	NSFTV323	DNJ 140	DNJ 140	BGD	aus	Indica	
117724	NSFTV324	DV 123	DV 123	BGD	aus	Indica	
121653	NSFTV330	Khao Pahk Maw	Khao Pahk Maw	THA	aus	Indica	
117642	NSFTV353	ARC 10376	ARC_10376	IND	aus	Indica	
117634	NSFTV357	9524	9524	IND	aus	Indica	
117876	NSFTV371	Santhi Sufaid	Santhi Sufaid	PAK	aus	Indica	
117896	NSFTV372	Sufaid	Sufaid	PAK	aus	Indica	
117774	NSFTV378	Kalubala Vee	Kalubala_Vee	LKA	aus	Indica	
117710	NSFTV44	Dhala Shaitta	Dhala_Shaitta	IND	aus	Indica	
117725	NSFTV49	DV 85	Dv85	BGD	aus	Indica	
117603	NSFTV6	ARC 7229	ARC_7229	IND	aus	Indica	
117268	NSFTV612	IR 64	IR64	PHL	indica	Indica	y
117276	NSFTV636	Sadu Cho	Sadu Cho	KOR	indica	Indica	y
117447	117447	Chitraj (DA 23)	CHITRAJ (DA 23)	BGD	indica	Indica	y
117460	117460	DA 9	DA 9	BGD	indica	Indica	y
117478	117478	Gie 57	GIE 57	VNM	indica	Indica	y
117559	117559	Peta	PETA	IDN	indica	Indica	y
117587	117587	TD 25	TD 25	THA	indica	Indica	y
117600	NSFTV234	Aijiaonante	Aijiaonante	CHN	indica	Indica	y
117623	NSFTV313	BR24	BR24	BGD	indica	Indica	y

GSaccno	HDRA_Sample_ID	IRGC_Name	IRGC	Origin Country	Subpopulation	Subspecies	Varieties with yield component data
117625	NSFTV299	SML 242	SML_242	SUR	indica	Indica	y
117636	NSFTV3	Ai-Chiao-Hong	Ai-Chiao-Hong	CHN	indica	Indica	y
117671	NSFTV21	Byakkoku Y 5006 Seln	Byakkoku Y 5006 Seln	AUS	indica	Indica	y
117681	NSFTV349	Chang Ch'Sang Hsu Tao	Chang_Ch'Sang_Hsu_Tao	CHN	indica	Indica	y
117682	NSFTV29	Chau	Chau	VNM	indica	Indica	y
117684	NSFTV30	Chiem Chanh	Chiem_Chanh	VNM	indica	Indica	y
117691	NSFTV35	CO18	CO18	IND	indica	Indica	y
117703	NSFTV315	Dawebyan	Dawebyan	BUR	indica	Indica	y
117705	NSFTV43	Dee Geo Woo Gen	Dee_Geo_Woo_Gen	TWN	indica	Indica	y
117713	NSFTV252	Djimoron	Djimoron	GNB	indica	Indica	y
117728	NSFTV241	ECIA76-S89-1	ECIA76-S89-1	CUB	indica	Indica	y
117751	NSFTV66	Hsia-Chioh-Keh-Tu	Hsia-Chioh-Keh-Tu	TWN	indica	Indica	y
117753	NSFTV231	Hunan Early Dwarf No. 3	Hunan Early Dwarf No. 3	CHN	indica	Indica	y
117757	NSFTV71	IR 36	IR_36	PHL	indica	Indica	y
117763	NSFTV74	IRGA 409	IRGA_409	BRA	indica	Indica	y
117766	NSFTV76	Jaya	Jaya	IND	indica	Indica	y
117767	NSFTV356	JC 117	JC_117	IND	indica	Indica	y
117770	NSFTV196	JM 70	JM70	MLI	indica	Indica	y
117784	NSFTV90	Kiang-Chou-Chiu	Kiang-Chou-Chiu	TWN	indica	Indica	y
117829	NSFTV119	Oryzica Llanos 5	Oryzica Llanos 5	COL	indica	Indica	y
117837	NSFTV123	Pagaiyahan	Pagaiyahan	TWN	indica	Indica	y
117838	NSFTV255	Pai Hok Glutinous	Pai Hok Glutinous	HKG	indica	Indica	y
117841	NSFTV126	Pappaku	Pappaku	TWN	indica	Indica	y
117842	NSFTV222	Paraiba Chines Nova	Paraiba Chines Nova	BRA	indica	Indica	y
117848	NSFTV129	Peh-Kuh	Peh-Kuh	TWN	indica	Indica	y
117871	NSFTV337	Sabharaj	Sabharaj	BGD	indica	Indica	y
117881	NSFTV142	Shai-Kuh	Shai-Kuh	CHN	indica	Indica	y
117889	NSFTV207	Sigadis	Sigadis	IDN	indica	Indica	y
117891	NSFTV148	Sintane Diofor	Sintane_Diofor	HVO	indica	Indica	y

GSaccno	HDRA_Sample_ID	IRGC_Name	IRGC	Origin Country	Subpopulation	Subspecies	Varieties with yield component data
117894	NSFTV208	Slo 17	SLO_17	IND	indica	Indica	y
117898	NSFTV269	Sundensis	Sundensis	KAZ	indica	Indica	y
117901	NSFTV235	Sze Guen Zim	Sze_Guen_Zim	CHN	indica	Indica	y
117906	NSFTV163	Taducan	Taducan	PHL	indica	Indica	y
117911	NSFTV209	Tchiganga	Tchibanga	GAB	indica	Indica	y
117912	NSFTV161	Teqing	Teqing	CHN	indica	Indica	y
117915	NSFTV162	TKM 6	TKM6	IND	indica	Indica	y
117940	NSFTV339	Yodanya	Yodanya	BUR	indica	Indica	y
120904	120904	Byat Kyar	BYAT KYAR	BUR	indica	Indica	y
120970	120970	Hong Mi Dong Mao Zhan	HONG MI DONG MAO ZHAN	CHN	indica	Indica	y
120988	120988	IR 77384-12-35-3-12-1-B	IR 77384-12-35-3-12-1-B	PHL	indica	Indica	y
121044	121044	Ma Gui Zi He	MA GU ZI HE	CHN	indica	Indica	y
121663	121663	Gajpati	GAJPATI	NPL	indica	Indica	y
120914	120914	Chua Dau	CHUA DAU	CHN	indica	Indica	
120952	120952	Ea Houm	Ea houm	LAO	indica	Indica	
121019	121019	Khao Daw Tai	KHAO DAW TAI	THA	indica	Indica	
121020	121020	Khao Pon	KHAO PON	THA	indica	Indica	
121045	121045	Ma Waine Ohn	MA WAINE OHN	BUR	indica	Indica	
121080	121080	Pawhtun	PAWHTUN	BUR	indica	Indica	
121087	121087	Pinursigi	PINURSIGI	PHL	indica	Indica	
121092	121092	Qing Gu	QING GU	CHN	indica	Indica	
121104	121104	RTS 5	RTS 5	VNM	indica	Indica	
121642	121642	Som Cau 70A	SOM CAU 70 A	VNM	indica	Indica	
121889	121889	Pelita Janggut	PELITA JANGGUT	IDN	indica	Indica	
124447	AFY08610786	Padi Oro	PADI ORO	IDN	indica	Indica	
117619	NSFTV102	Leung Pratew	Leung Pratew	THA	indica	Indica	
117814	NSFTV106	Minghui 63	Minghui_63	CHN	indica	Indica	

GSaccno	HDRA_Sample_ID	IRGC_Name	IRGC	Origin Country	Subpopulation	Subspecies	Varieties with yield component data
117818	NSFTV110	Mudgo	Mudgo	IND	indica	Indica	
117826	NSFTV117	O-Luen-Cheung	O-Luen-Cheung	TWN	indica	Indica	
117840	NSFTV125	Pao-Tou-Hung	Pao-Tou-Hung	CHN	indica	Indica	
117849	NSFTV130	Peh-Kuh-Tsao-Tu	Peh-Kuh-Tsao-Tu	TWN	indica	Indica	
117859	NSFTV132	Rathuwee	Rathuwee	LKA	indica	Indica	
117622	NSFTV137	RTS14	RTS14	VNM	indica	Indica	
117880	NSFTV141	Seratoes Hari	Seratoes_Hari	IDN	indica	Indica	
117888	NSFTV145	Short Grain	Short Grain	THA	indica	Indica	
117907	NSFTV156	Taichung Native 1	Taichung_Native_1	TWN	indica	Indica	
117944	NSFTV172	Zhenshan 2	Zhenshan 2	CHN	indica	Indica	
117858	NSFTV203	Radin Ebos 33	Radin_Ebos_33	MYS	indica	Indica	
117759	NSFTV284	IR-44595	IR-44595	NPL	indica	Indica	
117893	NSFTV294	SL 22-613	SL 22-613	SLE	indica	Indica	
117731	NSFTV325	EMATA A 16-34	EMATA A 16-34	BUR	indica	Indica	
117685	NSFTV348	China 1039	China_1039	CHN	indica	Indica	
117280	NSFTV642	Zhenshan 97B	Zhenshan 97B	CHN	indica	Indica	
117271	NSFTV643	Minghui 63	Minghui_63	CHN	indica	Indica	
117758	NSFTV72	IR 8	IR_8	PHL	indica	Indica	
117768	NSFTV77	JC149	JC149	IND	indica	Indica	
121923	121923	Nep Me Hoa Binh	NEP ME HOA BINH	VNM	temperate japonica	Japonica	y
121340	121340	Gao Gan Da Nuo	GAO GAN DA NUO	CHN	temperate japonica	Japonica	
121924	121924	Nep Vang Ong	NEP VANG ONG	VNM	temperate japonica	Japonica	
117264	NSFTV174	Azucena	Azucena	PHL	tropical japonica	Japonica	y
117272	NSFTV108	Moroberekan	Moroberekan	GIN	tropical japonica	Japonica	y
117517	117517	Kinandang Patong	KINANDANG PATONG	PHL	tropical japonica	Japonica	y
117560	117560	Pin Kaeo	PIN KAEO	THA	tropical japonica	Japonica	y
117599	NSFTV242	27	27	DOM	tropical japonica	Japonica	y
117607	NSFTV24	Carolina Gold	CarolinaGold	USA	tropical japonica	Japonica	y
117626	NSFTV214	Improved Bluebonnet	Improved Bluebonnet	HND	tropical japonica	Japonica	y

GSaccno	HDRA_Sample_ID	IRGC_Name	IRGC	Origin Country	Subpopulation	Subspecies	Varieties with yield component data
117629	NSFTV384	318	318	TUR	tropical japonica	Japonica	y
117632	NSFTV176	583	583	ECU	tropical japonica	Japonica	y
117664	NSFTV183	Boa Vista	Boa_Vista	SLV	tropical japonica	Japonica	y
117693	NSFTV396	Cocodrie	Cocodrie	USA	tropical japonica	Japonica	y
117698	NSFTV37	Cuba 65	Cuba 65	CUB	tropical japonica	Japonica	y
117707	NSFTV190	Delrex	Delrex	USA	tropical japonica	Japonica	y
117723	NSFTV46	Dourado Agulha	Dourado_Agulha	BRA	tropical japonica	Japonica	y
117736	NSFTV54	Fortuna	Fortuna	USA	tropical japonica	Japonica	y
117755	NSFTV70	Iguape Cateto	Iguape_Cateto	HTI	tropical japonica	Japonica	y
117760	NSFTV195	IRAT 13	IRAT_13	CIV	tropical japonica	Japonica	y
117762	NSFTV226	IRAT 44	IRAT_44	HVO	tropical japonica	Japonica	y
117776	NSFTV84	Kaniranga	Kaniranga	IDN	tropical japonica	Japonica	y
117786	NSFTV92	Kinastano	Kinastano	PHL	tropical japonica	Japonica	y
117798	NSFTV394	Lady Wright Seln	Lady Wright Seln	USA	tropical japonica	Japonica	y
117804	NSFTV350	Ligerito	Ligerito	COL	tropical japonica	Japonica	y
117805	NSFTV308	Llanero 501	Llanero 501	VEN	tropical japonica	Japonica	y
117835	NSFTV122	Padi Kasalle	Padi_Kasalle	IDN	tropical japonica	Japonica	y
117843	NSFTV201	Pate Blanc Mn 1	Pate_Blanc_Mn_1	CIV	tropical japonica	Japonica	y
117853	NSFTV377	PR 304	PR 304	PRI	tropical japonica	Japonica	y
117854	NSFTV202	Pratao	Pratao	BRA	tropical japonica	Japonica	y
117857	NSFTV310	R 101	R 101	ZAR	tropical japonica	Japonica	y
117897	NSFTV150	Sultani	Sultani	EGY	tropical japonica	Japonica	y
117914	NSFTV258	Tia Bura	Tia_Bura	IDN	tropical japonica	Japonica	y
117920	NSFTV285	Tox 782-20-1	Tox_782-20-1	NGA	tropical japonica	Japonica	y
117932	NSFTV239	WAB 502-13-4-1	WAB_502-13-4-1	CIV	tropical japonica	Japonica	y
117935	NSFTV213	WC 3397	Wc 3397	JAM	tropical japonica	Japonica	y
121193	121193	Bakung (H)	BAKUNG (H)	MYS	tropical japonica	Japonica	y
121195	121195	Dinolores	DINOLORES	PHL	tropical japonica	Japonica	y
121635	121635	Ranau Kadai	RANAU KADAI	MYS	tropical japonica	Japonica	y

GSaccno	HDRA_Sample_ID	IRGC_Name	IRGC	Origin Country	Subpopulation	Subspecies	Varieties with yield component data
121652	NSFTV251	H256-76-1-1-1	H256-76-1-1-1	ARG	tropical japonica	Japonica	y
121905	121905	Cina	CINA	IDN	tropical japonica	Japonica	y
121926	121926	Padi Teras	PADI TERAS	IDN	tropical japonica	Japonica	y
121939	121939	Ubak Bakong	UBAK BAKONG	MYS	tropical japonica	Japonica	y
122052	122052	Ganigi	GANIGI	IDN	tropical japonica	Japonica	y
122095	122095	IR 47686-09-01-B-1	IR 47686-09-01-B-1	PHL	tropical japonica	Japonica	y
122108	122108	IR 68704-145-1-1-B	IR 68704-145-1-1-B	PHL	tropical japonica	Japonica	y
122120	122120	IRAT 335	IRAT 335	BOL	tropical japonica	Japonica	y
124490	124490	Padi Kompai	PADI KOMPAI	IDN	tropical japonica	Japonica	y
121385	121385	Khao Kap Sang	KHAO KAP SANG	LAO	tropical japonica	Japonica	
121391	121391	Kikilong	KIKILONG	MYS	tropical japonica	Japonica	
121475	121475	Popong	POPONG	MYS	tropical japonica	Japonica	
121523	121523	Toang	TOANG	IDN	tropical japonica	Japonica	
121672	121672	MUT IAC 25-44-807	MUT IAC 25-44-807	GUY	tropical japonica	Japonica	
122031	122031	Davao	DAVAO	PHL	tropical japonica	Japonica	
122063	122063	Gogo	GOGO	IDN	tropical japonica	Japonica	
122134	122134	Kendinga 5H	KENDINGA 5 H	MYS	tropical japonica	Japonica	
117830	NSFTV120	OS6	OS6	LBR	tropical japonica	Japonica	
117890	NSFTV147	Sinampaga Selection	Sinampaga_Selection	PHL	tropical japonica	Japonica	
117918	NSFTV164	Tondok	Tondok	IDN	tropical japonica	Japonica	
117921	NSFTV165	Trembese	Trembese	IDN	tropical japonica	Japonica	
117668	NSFTV185	British Honduras Creole	British_Honduras_Creole	BLZ	tropical japonica	Japonica	
117694	NSFTV188	Coppocina	Coppocina	BGR	tropical japonica	Japonica	
117934	NSFTV212	WC 2810	Wc 2810	FSM	tropical japonica	Japonica	
117936	NSFTV215	WC 4443	WC_4443	BOL	tropical japonica	Japonica	
117674	NSFTV22	Caawa/Fortuna 6-103-15	Caawa/Fortuna_6-103-15	TWN	tropical japonica	Japonica	
117855	NSFTV223	Priano Guaira	Priano_Guaira	BRA	tropical japonica	Japonica	
117675	NSFTV23	Canella De Ferro	Canella_De_Ferro	BRA	tropical japonica	Japonica	
117931	NSFTV240	Wab 501-11-5-1	Wab 501-11-5-1	CIV	tropical japonica	Japonica	

GSaccno	HDRA_Sample_ID	IRGC_Name	IRGC	Origin Country	Subpopulation	Subspecies	Varieties with yield component data
117836	NSFTV274	Padi Pagalong	Padi_Pagalong	MYS	tropical japonica	Japonica	
117874	NSFTV280	Saku	Saku	MNG	tropical japonica	Japonica	
117756	NSFTV286	IITA 135	IITA 135	NGA	tropical japonica	Japonica	
124372	NSFTV309	Manzano	Manzano	ZAR	tropical japonica	Japonica	
117678	NSFTV342	Cenit	Cenit	ARG	tropical japonica	Japonica	
117695	NSFTV347	Creole	Creole	BLZ	tropical japonica	Japonica	
117706	NSFTV391	Della	Della	USA	tropical japonica	Japonica	
117699	NSFTV397	Cybonnet	Cybonnet	USA	tropical japonica	Japonica	
117742	NSFTV59	Gogo Lempuk	Gogo Lempuk	IDN	tropical japonica	Japonica	
117761	NSFTV73	IRAT 177	IRAT 177	CIV	tropical japonica	Japonica	
117764	NSFTV75	Jambu	Jambu	IDN	tropical japonica	Japonica	
117816	NSFTV770	Mojito Colorado	Mojito Colorado	BOL	tropical japonica	Japonica	
117643	NSFTV8	Asse Y Pung	Asse_Y_Pung	PHL	tropical japonica	Japonica	
117796	NSFTV99	LAC 23	LAC_23	LBR	tropical japonica	Japonica	

Supplementary Table 2. List of phenotypes used for GWAS.

	Trait Name	PANorama Software Trait Identifier	Unit	Logarithmic Transformation	Agronomic vs. PANorama	Detailed Description of Trait Collection
HD	Heading Date	NA	days	Y	Agronomic	Number of days after transplanting when ~50% of plants from a plot replicate show emerging panicles
PH	Plant Height	NA	cm	y	Agronomic	Distance from base of the culm to the tip of the longest panicle
PN	Panicle Number	NA	count	-	Agronomic	Number of reproductive tillers per plant
BT	Booting	NA	days	y	Agronomic	Number of days after transplanting when ~50% of plants from a plot replicate reach booting stage (visible flag leaf)
SBN	Secondary Branch Number	NA	categorical	-	Agronomic	Scored on a 0-4 scale. 0=no spikelets born on secondary branches; 1=25% spikelets born on secondary branches; 2=50% of spikelets born on secondary branches; 3=75% spikelets born on secondary branches; 4=100% of spikelets born on secondary branches in clustered formation
FLA	Flag Leaf Area	NA	mm squared	y	Agronomic	Scored using Li-Cor LI3100 leaf area meter
totBM	Total Shoot Biomass	NA	grams	-	Agronomic	Dry shoot biomass; 5 plants amassed for replicate
PW	Panicle Weight	NA	grams	-	Agronomic	Total dry panicle weight; 5 plants amassed for replicate; corrected for seed moisture content
totGW	Grain Weight	NA	grams	y	Agronomic	Weight of threshed grains; 5 plants amassed for replicate; corrected for seed moisture content
1000GW	1000-Grain Weight	NA	grams	y	Agronomic	Weight of 1000, randomly sampled grains; Counted from total grain weight; corrected for seed moisture content
GN	Grain Number	NA	count	y	Agronomic	Total Grain Weight divided by 1000-grain weight
EL	Exsertion Length	LengthOfExtrusioncm (Initial Main Axis Point - Extrustion Point)	cm	y	PANorama	Distance from the flag leaf ligule to the panicle notch; Automatically implemented in PANorama (IMAP - EP)

RL	Rachis Length	LengthOfMaixiscm (Final Main Axis Point - Intial Main Axis Point)	cm	y	PANorama	Distance from the panicle notch to the inflorescence meristem abortion point; Automatically implemented in PANorama (FAMP-IMAP)
PL	Panicle Length	InflorescenceLengthcm	cm	y	PANorama	Distance from the panicle notch to the tip of the terminal panicle primary branch; Automatically implemented in PANorama, when IMAP is selected at the panicle notch.
tBL	Tip Branch Length	NA	cm	-	PANorama	Length of the terminal primary branch; PL-RL
NL	Internode Length	MeanLengthBetweenNodescm	cm	y	PANorama	The distance between two nodes, after collapsing of primary branches. NL is the average length of all internodes in an image.
NLsd	Internode Length Standard Deviation	StdevLengthBetweenNodescm	cm	y	PANorama	The standard deviation of NL.
xNL	Maximum Internode Length	MaxLengthBetweenNodescm	cm	y	PANorama	The maximum distance between two nodes within a panicle, after primary branches have been collapsed.
nNL	Minimum Internode Length	MinLengthBetweenNodescm	cm	y	PANorama	The minimum distance between two nodes within a panicle, after primary branches have been collapsed.
PBL	Primary Branch Length	MeanLengthOfPrimaryBranchescm	cm	-	PANorama	The distance from the first pixel in a primary branch to neighbor the rachis skeleton, to the 8- neighbor pixel at the tip of a terminal seed. PBL is the average of all primary branches within a panicle.
PBLsd	Primary Branch Length Standard Deviation	StdevLengthOfPrimaryBranchescm	cm	-	PANorama	The standard deviation of MeanLengthOfPrimaryBranches.
xPBL	Maximum Primary Branch Length	MaxLengthOfPrimaryBranchescm	cm	-	PANorama	The maximum length of a primary branch within a panicle.
nPBL	Minimum Primary Branch Length	MinLengthOfPrimaryBranchescm	cm	-	PANorama	The minimum length of a primary branch within a panicle.
PBLin	Primary Branch Length in the Lower Half of the Panicle	MeanLengthOfPrimaryBranchesInInferiorSlabcm	cm	-	PANorama	The average length of all primary branches below the JP.
PBLinsd	Primary Branch Length in the Lower Half of the Panicle Standard Deviation	StdevLengthOfPrimaryBranchesInInferiorSlabcm	cm	-	PANorama	The standard deviation of all primary branch lengths within a panicle below the JP.

xPBLin	Maximum Primary Branch Length in the Lower Half of the Panicle	MaxLengthOfPrimaryBranchesInInferiorSlabcm	cm	-	PANorama	The maximum length of a primary branch within a panicle below the JP.
nPBLin	Minimum Primary Branch Length in the Lower Half of the Panicle	MinLengthOfPrimaryBranchesInInferiorSlabcm	cm	-	PANorama	The minimum length of a primary branch within a panicle below the JP.
PBLsu	Primary Branch Length in the Upper Half of the Panicle	MeanLengthOfPrimaryBranchesInSuperiorSlabcm	cm	-	PANorama	The average length of all primary branches above the JP.
PBLsusd	Primary Branch Length in the Upper Half of the Panicle Standard Deviation	StdevLengthOfPrimaryBranchesInSuperiorSlabcm	cm	-	PANorama	The standard deviation of all primary branch lengths within a panicle above the JP.
xPBLsu	Maximum Primary Branch Length in the Upper Half of the Panicle	MaxLengthOfPrimaryBranchesInSuperiorSlabcm	cm	-	PANorama	The maximum length of a primary branch within a panicle above the JP.
nPBLsu	Minimum Primary Branch Length in the Upper Half of the Panicle	MinLengthOfPrimaryBranchesInSuperiorSlabcm	cm	-	PANorama	The minimum length of a primary branch within a panicle above the JP.
TR	Thickness of the Rachis	MeanThicknessOfMaixiscm	cm	-	PANorama	Thickness is calculated as 2x the distance between a skeleton point and its closest pixel on the image contour. Distances are obtained directly from the Euclidean distance map of the panicle mask during the skeletonization step. MeanThicknessOfMainAxis is the average of all thickness measures between the IMAP and FMAP. In rice, these points are selected to reflect mean thickness of the rachis (TR).
TRsd	Thickness of the Rachis Standard Deviation	StdevThicknessOfMaixiscm	cm	-	PANorama	The standard deviation of TR.
xTR	Maximum Thickness of Rachis	MaxThicknessOfMaixiscm	cm	-	PANorama	The maximum thickness between the IMAP and the FMAP.

TE	Thickness of Exsertion	MeanThicknessOfExtrusioncm	cm	-	PANorama	Thickness is calculated as 2x the distance between a skeleton point and its closest pixel on the image contour. Distances are obtained directly from the Euclidean distance map of the panicle mask during the skeletonization step. MeanThicknessOfExtrusion is the average of all thickness measures between the EP and IMAP. In rice, these points are selected to reflect mean thickness of exsertion (TE).
TEsd	Thickness of Exsertion Standard Deviation	StdevThicknessOfExtrusioncm	cm	-	PANorama	The standard deviation of TE.
xTE	Maximum Thickness of Exsertion	MaxThicknessOfExtrusioncm	cm	-	PANorama	The maximum of all thickness measures between the EP and the IMAP. In rice, these points are selected to reflect max thickness of exsertion (xTE).
nTE	Minimum Thickeness of Exsertion	MinThicknessOfExtrusioncm	cm	-	PANorama	The minimum of all thickness measures between the EP and the IMAP. In rice, these points are selected to reflect min thickness of exsertion (xTE).
PBN	Primary Branch Number	NumberOfPrimaryBranches	count	y	PANorama	A primary branch is any distinct skeleton segment that attaches (i.e. shares a neighboring pixel with the main axis) between the IMAP and FMAP. The MINBRANCSZ parameter within iftPANorama.h defines the minimum length requirement of a primary branch.
PBNsu	Primary Branch Number in the Upper Half of the Panicle	NumberOfPrimaryBranchesInSuperiorSlab	count	y	PANorama	Number of primary branches above the JP.
PBNin	Primary Branch Number in the Lower Half of the Panicle	NumberOfPrimaryBranchesInInferiorSlab	count	y	PANorama	Number of primary branches below the JP.
NN	Internode Number	NumberOfNodesOnMaixis	count	-	PANorama	Nodes are defined as anywhere a meristem transition has occurred. The pixels at which primary branches attach are considered nodes. If a branch forms at the IMAP and FMAP, they are also treated as nodes. Branches that attach within a specified distance of one another are automatically collapsed into a single internode point using the MINJCTDIST parameter in iftPANorama.h, and the pixel closest to the geometric center of the cluster is considered the true internode point. NN is defined is as the total number of nodes in an image.

NNsu	Internode Number in the Upper Half of the Panicle	NumberOfNodesOnMaixisInSuperiorSlab	count	-	PANorama	Number of nodes above the JP.
NNin	Internode Number in the Lower Half of the Panicle	NumberOfNodesOnMaixisInInferiorSlab	count	-	PANorama	Number of nodes below the JP.
BpN	Number of Primary Branches per Internode	MeanNumberOfBranchesPerNode	count	y	PANorama	BpN is the average number of branches per node for all nodes within a panicle. Branches are collapsed into single nodes using the MINJCTDIST parameter in iftPANorama.h, and the pixel closest to the geometric center of the cluster is considered the true node.
BpNsd	Number of Primary Branches per Internode Standard Deviation	StdevNumberOfBranchesPerNode	count	-	PANorama	The standard deviation when calculating NumberOfBrancesPerNode.
BpNsu	Number of Primary Branches per Internode in the Upper Half of the Panicle	MeanNumberOfBranchesPerNodeInSuperiorSlab	count	y	PANorama	Number of branches per internode above the JP.
BpNsusd	Number of Primary Branches per Internode in the Upper Half of the Panicle Standard Deviation	StdevNumberOfBranchesPerNodeInSuperiorSlab	count	y	PANorama	The standard deviation when calculating BpNsu.
BpNin	Number of Primary Branches per Internode in the Lower Half of the Panicle	MeanNumberOfBranchesPerNodeInInferiorSlab	count	y	PANorama	Number of branches per internode below the JP.
BpNinsd	Number of Primary Branches per Internode in the Lower Half of the Panicle Standard Deviation	StdevNumberOfBranchesPerNodeInInferiorSlab	count	-	PANorama	The standard deviation when calculation BpNin.
xBpN	Maximum Number of Primary Branches per Internode	MaxNumberOfBranchesPerNode	count	-	PANorama	xBpN is the maximum number of branches to occur at a single node within a panicle. Branches are collapsed into single nodes using the MINJCTDIST parameter in iftPANorama.h, and the pixel closest to the geometric center of the cluster is considered the true node.

xBpNsu	Maximum Number of Primary Branches per Internode in the Upper Half of the Panicle	MaxNumberOfBranchesPerNodeInSuperiorSlab	count	y	PANorama	xBpN aove the JP.
xBpNin	Maximum Number of Primary Branches per Internode in the Lower Half of the Panicle	MaxNumberOfBranchesPerNodeInInferiorSlab	count	y	PANorama	xBpN below the JP.
totPBL	Total Primary Branch Length	NA	cm	y	PANorama	The sum of all primary branches, averaged for all panicles in an accession; Calculated from the PANorama ExtraPanicleMeasures.csv file
xPBN	Maximum Primary Branch Number	NA	count	-	PANorama	The maximum number of panicle branches among all panicle replicates for an accession; Calculated from the PANorama ExtraPanicleMeasures.csv file
xtotPBL	Maximum Total Primary Branch Length	NA	cm	y	PANorama	The maximum sum length of all primary panicle branches among all panicle replicates for an accession; Calculated from the PANorama ExtraPanicleMeasures.csv file
nPBN	Minimum Primary Branch Number	NA	count	y	PANorama	The minimum number of panicle branches among all panicle replicates for a line; Calculated from the PANorama ExtraPanicleMeasures.csv file
ntotPBL	Minimum Total Primary Branch Length	NA	cm	y	PANorama	The minimum sum length of all primary panicle branches among all panicle replicates for an accession; Calculated from the PANorama ExtraPanicleMeasures.csv file
PBNsd	Primary Branch Number Standard Deviation	NA	count	y	PANorama	The standard deviation for primary panicle branch number among all panicles in an accession; Calculated from the PANorama ExtraPanicleMeasures.csv file
totPBLsd	Total Primary Branch Length Standard Deviation	NA	cm	y	PANorama	The standard deviation of the sum length of all primary panicle branches among all panicles in an accession; Calculated from the PANorama ExtraPanicleMeasures.csv file

Notes:
1. To collect PANorama phenotypes, five panicles per plot were randomly harvested from different but genetically identical plants and photographed.

2. Plant height and panicle number were measured on five randomly selected plants per plot replicate. Vegetative and reproductive plant matter was amassed per plot and dried in ovens. Dry samples were used to calculate total biomass, panicle weight, and grain weight traits.

3. Panicle weight, grain weight, and total grain weight were corrected by moisture content. Grain moisture percentage was measured using a digital grain moisture tester. Seeds from dry panicle samples were amassed, tested three times, and the percent moisture content was averaged. The following equation was used to correct total weight measurements: Weight measurement * [1 - (Average%Moisture/100)]

4. Secondary branch number and flag leaf area traits using the same randomly harvested panicles used in PANorama imaging. For secondary branch number, the average categorical score per panicle (0-4) was calculated using every primary branch.

5. The PANorama Software Trait Identifier Column indicates how traits are labeled within the PANorama2.0 software package.

6. EP (Exsertion Point), IMAP (Initial Main Axis Point), and FMAP (Final Main Axis Point) are selected for every image using a graphical user interface within PANorama2.0. Junction Point (JP) is defined by the MINFSTJCTLEN parameter within the iftPANorama.h file. In order to calculate traits in the upper and lower halves of the panicle, the parameter was set to 0.5 (i.e. one-half).

Supplementary Table 3. GWAS results for panicle traits.

**Subpopulation**	**Mixed model**	**Mixed Model + HD**	**Mixed Model + HD + NL**	**Mixed Model + HD + PBL**	**Mixed Model + HD + PBN**	**Mixed Model + HD + RL**
All	709 (358)	496 (256)	283 (196)	376 (251)	445 (198)	477 (258)
aus	148 (52)	117 (44)	111 (60)	52 (42)	89 (56)	82 (60)
indica	48 (38)	54 (39)	57 (45)	57 (48)	95 (76)	51 (46)
tropical japonica	132 (41)	49 (24)	50 (29)	44 (22)	96 (45)	70 (41)
Total associations	1037 (489)	716 (363)	501 (330)	529 (363)	725 (375)	680 (405)

The number of significant peaks is in parentheses, and is defined by binning significant SNPs using a sliding window of linkage disequilbrium (LD). Covariate abbreviations are as follows: heading date (HD); internode length (NL); primary branch length (PBL); primary branch number (PBN); rachis length (RL).

Supplementary Table 4. Overlap between panicle trait GWAS results.

**Proportion of shared GWAS results**	**Mixed Model + HD + RL**	**Mixed Model + HD + NL**	**Mixed Model + HD + PBN**	**Mixed Model + HD + PBL**
Number of SNPs shared with HD run Total number of SNPs identified in run	537/682 (78.7%)	422/501 (84.2%)	546/725 (75.3%)	439/529 (83%)
Number of peaks shared with HD run Total number of peaks identified in run	292/405 (72.1%)	259/330 (78.5%)	236/375 (62.9%)	285/363 (78.5%)

The number of significant peaks is defined by binning significant SNPs using a sliding window of linkage disequilbrium (LD). Percentage of overlap between each covariate and the HD covariate is indicated in parentheses. Covariate abbreviations are as follows: heading date (HD); internode length (NL); primary branch length (PBL); primary branch number (PBN); rachis length (RL).

Supplementary Table 5. QTL identified in the RIL population.

Trait Name	Trait Acronym	Chr	Peak (cM)	Peak Marker	Left Marker, Right Marker	PVE	LOD	Add. Eff.
Maximum Number of Primary Branches per Internode in the Upper Half of the Panicle	xBpNsu	1	26.78	S1_5015805	S1_4826553, S1_18455172	5.68	3.84	-0.04*
Number of Primary Branches per Internode in the Upper Half of the Panicle Standard Deviation	BpNsusd	1	26.78	S1_5015819	S1_4978128, S1_5261761	5.76	4.46	-0.04*
Number of Primary Branches per Internode in the Upper Half of the Panicle	BpNsu	1	74.27	S1_12555138	S1_4978128, S1_18455172	9.21	4.67	-0.04^{np}
Minimum Internode Length	nNL	1	157.41	S1_30018773	S1_26869417, S1_31817994	6.41	3.77	-0.04*
Internode Length Standard Deviation	NLsd	1	189.65	S1_37521762	S1_36389924, S1_39320602	17.51	7.02	-0.16cm^{np}
Internode Length	NL	1	190.56	S1_37780914	S1_35032514, S1_38150186	22.2	14.09	-0.06*
Maximum Internode Length	xNL	1	190.56	S1_37780914	S1_36953664, S1_38150306	20.2	8.23	-0.52cm^{np}
Thickness of the Rachis	TR	1	191.47	S1_37946665	S1_34874035, S1_38996012	7.92	3.88	-0.0021cm^{np}
Panicle Length	PL	1	192.69	S1_38150186	S1_37370036, S1_38642939	18.08	8.05	-1.71cm
Rachis Length	RL	1	193.01	S1_38383505	S1_38372528, S1_38397011	7.73	6.2	-1.62cm
Rachis Length	RL	1	193.01	S1_38411487	S1_38410392, S1_38412702	7.73	6.2	-1.62cm
Exsertion Length	EL	1	193.32	S1_38527519	S1_38116619, S1_38642939	18.04	10.91	-1.21cm^{np}
Maximum Exsertion Thickness	xTE	1	195.73	S1_38690482	S1_35764131, S1_38996012	8.8	4.81	-0.01cm^{np}

Trait Name	Trait Acronym	Chr	Peak (cM)	Peak Marker	Left Marker, Right Marker	PVE	LOD	Add. Eff.
Number of Primary Branches per Internode in the Lower Half of the Panicle	BpNin	1	207.04	S1_40263574	S1_39800289, S1_42108167	15.22	6.68	0.1np
Heading Date	HD	1	209.76	S1_40706234	S1_40468832, S1_40838070	9.11	8.47	-4.71 days^{np}
Number of Primary Branches per Internode	BpN	1	210.66	S1_40792028	S1_39989656, S1_42432186	17.35	7.61	0.02*
Number of Primary Branches per Internode Standard Deviation	BpNsd	1	210.66	S1_40792028	S1_39910384, S1_40896859	11.21	6.61	0.06
Number of Primary Branches per Internode in the Lower Half of the Panicle Standard Deviation	BpNinsd	1	216.44	S1_41271407	S1_39989656, S1_42256041	14.95	5.91	0.09
Primary Branch Number	PBN	1	220.09	S1_42038034	S1_40596824, S1_42256041	5.5	3.94	0.8
Primary Branch Number in the Lower Half of the Panicle	PBNin	1	220.69	S1_42050495	S1_41133234, S1_42256041	16.15	7.8	0.72
Maximum Number of Primary Branches per Internode in the Lower Half of the Panicle	xBpNin	1	220.69	S1_42050495	S1_39989656, S1_42256041	16.95	7.44	0.22
Maximum Number of Primary Branches per Internode	xBpN	1	220.99	S1_42054294	S1_39989656, S1_42171085	10.1	6.92	0.17
Maximum Primary Branch Number	xPBN	1	223.43	S1_42197808	S1_41172542, S1_43076281	9.95	5.54	0.72
Maximum Number of Primary Branches per Internode	xBpN	2	42.35	S2_5425842	S2_4910917, S2_6638269	6.32	4.48	0.13
Number of Primary Branches per Internode Standard Deviation	BpNsd	2	42.49	S2_5466966	S2_4908277, S2_6638269	7.39	4.49	0.05

**Trait Name**	**Trait Acronym**	**Chr**	**Peak (cM)**	**Peak Marker**	**Left Marker, Right Marker**	**PVE**	**LOD**	**Add. Eff.**
Maximum Number of Primary Branches per Internode in the Lower Half of the Panicle	xBpNin	2	44.47	S2_5592612	S2_4982707, S2_6638269	8.94	4.11	0.15
Panicle Number	PN	2	65.16	S2_10189314	S2_6749562, S2_11101262	12.66	5.29	1.37
Minimum Exsertion Thickness	nTE	2	123.71	S2_24587075	S2_22561101, S2_25420045	14.55	6.17	-0.01cm^{np}
Minimum Exsertion Thickness	nTE	3	4.56	S3_1007975	S3_867099, S3_1681663	8.31	3.65	0.01cm^{np}
Maximum Total Primary Branch Length	xtotPBL	3	4.86	S3_1033363	S3_665284, S3_1989193	9.06	4.19	10.77cm
Heading Date	HD	3	6.07	S3_1174570	S3_1033363, S3_1529264	14.85	12.96	-4.53 days^{np}
Maximum Exsertion Thickness	xTE	3	17.26	S3_2546716	S3_1938374, S3_3204141	7.92	4.36	0.01cm^{np}
Total Primary Branch Length	totPBL	3	18.16	S3_2615175	S3_955311, S3_3204141	8.92	4.65	6.91cm
Maximum Primary Branch Length in the Upper Half of the Panicle	xPBLsu	3	43.32	S3_5602318	S3_5310718, S3_27782699	7.82	4.06	0.04*
Minimum Internode Length	nNL	3	95.3	S3_15829528	S3_15551732, S3_16438463	12.63	7.08	0.07*
Internode Number	NN	3	95.3	S3_15829528	S3_15474234, S3_22775738	12.09	6.1	-0.65
Internode Number in the Lower Half of the Panicle	NNin	3	95.3	S3_15829528	S3_14937446, S3_27698909	10.36	4.63	-0.4
Minimum Primary Branch Number	nPBN	3	98.02	S3_16066102	S3_15454097, S3_27693150	11.72	5.08	-1.09^{np}
Internode Number in the Upper Half of the Panicle	NNsu	3	111.07	S3_20968119	S3_16658639, S3_23048829	4.1	4.1	-0.26

**Trait Name**	**Trait Acronym**	**Chr**	**Peak (cM)**	**Peak Marker**	**Left Marker, Right Marker**	**PVE**	**LOD**	**Add. Eff.**
Minimum Primary Branch Length	nPBL	3	142.28	S3_26493127	S3_25325717, S3_29541959	8.21	3.82	0.05*
Internode Length	NL	3	143.79	S3_26730228	S3_14655869, S3_29281809	5.14	3.78	0.03*
Primary Branch Number in the Upper Half of the Panicle	PBNsu	3	145.92	S3_27147027	S3_26714008, S3_27698909	9.84	6.08	-0.04*
Number of Primary Branches per Internode Standard Deviation	BpNsd	3	146.52	S3_27206391	S3_26805000, S3_27646885	8.83	5.3	-0.04
Maximum Number of Primary Branches per Internode	xBpN	3	146.52	S3_27206391	S3_26805000, S3_27574658	6.71	4.74	-0.13
Primary Branch Number	PBN	3	146.59	S3_27287420	S3_26805000, S3_27693150	8.83	6.12	-0.84
Number of Primary Branches per Internode in the Upper Half of the Panicle Standard Deviation	BpNsusd	3	147.43	S3_27441141	S3_26805000, S3_27698909	15.3	10.82	-0.04*
Maximum Number of Primary Branches per Internode in the Upper Half of the Panicle	xBpNsu	3	147.43	S3_27498202	S3_26805000, S3_27698909	13.04	8.28	-0.04*
Number of Primary Branches per Internode in the Upper Half of the Panicle	BpNsu	3	147.43	S3_27514086	S3_26805000, S3_28601847	10.61	5.33	-0.03[np]
Tip Branch Length	tBL	3	148.93	S3_27698909	S3_26448604, S3_29656367	8.45	4.82	0.08*
Minimum Primary Branch Length in the Upper Half of the Panicle	nPBLsu	3	158.02	S3_29359583	S3_28383390, S3_29541959	11.29	7.49	0.08*
Heading Date	HD	3	178.72	S3_32851774	S3_30719621, S3_33127721	3.95	3.92	3.52 days[np]

Trait Name	Trait Acronym	Chr	Peak (cM)	Peak Marker	Left Marker, Right Marker	PVE	LOD	Add. Eff.
Exsertion Length	EL	4	2.14	S4_405005	S4_10622, S4_19203350	6.11	4.07	0.81cm^{np}
Maximum Primary Branch Length in the Upper Half of the Panicle	xPBLsu	4	22.42	S4_5672520	S4_1814589, S4_6948320	7.68	3.99	0.03*
Rachis Length	RL	4	98.04	S4_25363835	S4_24846658, S4_26493364	5.47	4.49	-0.93cm
Primary Branch Number in the Lower Half of the Panicle	PBNin	4	98.04	S4_25363835	S4_24116694, S4_28557463	7.84	4	-0.43
Maximum Primary Branch Number	xPBN	4	100.17	S4_25983969	S4_21310144, S4_33337908	6.57	3.75	-0.6
Maximum Exsertion Thickness	xTE	4	108.52	S4_27482637	S4_25112152, S4_31208138	8.74	4.78	-0.01cm^{np}
Number of Primary Branches per Internode	BpN	4	122.47	S4_29895055	S4_28694937, S4_31157231	10.4	4.75	-0.02*
Number of Primary Branches per Internode in the Upper Half of the Panicle	BpNsu	4	127.4	S4_30865838	S4_30398628, S4_32506016	9.06	4.6	-0.03np
Maximum Thickness of the Rachis	xTR	4	128	S4_30887947	S4_27432714, S4_31157231	15.05	5.95	-0.01*
Number of Primary Branches per Internode Standard Deviation	BpNsd	4	128	S4_30889916	S4_30398628, S4_31157231	14.09	8.12	-0.05
Maximum Number of Primary Branches per Internode	xBpN	4	128	S4_30894887	S4_30255555, S4_31237412	10.94	7.44	-0.13
Thickness of the Rachis	TR	4	128	S4_30894887	S4_30865838, S4_31157231	18.25	8.4	-0.003cm^{np}
Exsertion Thickness	TE	4	128	S4_30894887	S4_25050908, S4_31157231	9.88	3.8	-0.01cm^{np}
Primary Branch Number in the Upper Half of the Panicle	PBNsu	4	128.9	S4_31020320	S4_30885454, S4_31157231	21.36	12.11	-0.07*

**Trait Name**	**Trait Acronym**	**Chr**	**Peak (cM)**	**Peak Marker**	**Left Marker, Right Marker**	**PVE**	**LOD**	**Add. Eff.**
Primary Branch Number	PBN	4	128.9	S4_31020320	S4_30735893, S4_31208138	11.26	7.65	-0.82
Number of Primary Branches per Internode in the Upper Half of the Panicle Standard Deviation	BpNsusd	4	128.91	S4_31157132	S4_30735893, S4_31237412	14.11	10.08	-0.04*
Maximum Number of Primary Branches per Internode in the Upper Half of the Panicle	xBpNsu	4	128.91	S4_31157132	S4_30428412, S4_31157231	16.59	10.24	-0.04*
Internode Number in the Upper Half of the Panicle	NNsu	4	128.91	S4_31157132	S4_30885454, S4_31289624	4.61	4.61	-0.29
Minimum Primary Branch Length in the Lower Half of the Panicle	nPBLin	5	8.94	S5_1236478	S5_32898, S5_1507074	10.27	3.95	-0.06*
Internode Length	NL	5	11.98	S5_1442539	S5_1247072, S5_1507074	7.62	5.47	-0.04*
Heading Date	HD	5	13	S5_1456951	S5_1237260, S5_2312488	5.4	5.25	2.52 days^{np}
Number of Primary Branches per Internode in the Upper Half of the Panicle Standard Deviation	BpNsusd	5	52.52	S5_7661534	S5_6991580, S5_16544219	6.29	4.85	-0.03*
Maximum Number of Primary Branches per Internode in the Upper Half of the Panicle	xBpNsu	5	52.52	S5_7661534	S5_6991580, S5_16559773	5.52	3.74	-0.02*
Maximum Total Primary Branch Length	xtotPBL	5	85.52	S5_20687384	S5_19798154, S5_24906893	10.1	4.65	11.39cm
Primary Branch Number	PBN	5	87.03	S5_20954473	S5_18908122, S5_22079772	5.99	4.26	0.83
Total Primary Branch Length	totPBL	5	87.03	S5_20986553	S5_20464081, S5_21080155	12.49	6.36	8.09cm

Trait Name	Trait Acronym	Chr	Peak (cM)	Peak Marker	Left Marker, Right Marker	PVE	LOD	Add. Eff.
Maximum Exsertion Thickness	xTE	5	87.03	S5_20986553	S5_20023184, S5_21677168	7.7	4.24	0.01cm^{np}
Maximum Primary Branch Number	xPBN	5	88.24	S5_21080332	S5_19798154, S5_24906893	8.47	4.77	0.85
Primary Branch Number in the Lower Half of the Panicle	PBNin	5	94.26	S5_21684588	S5_19152649, S5_23445666	9	4.55	0.64
Number of Primary Branches per Internode in the Lower Half of the Panicle	BpNin	5	96.09	S5_22096397	S5_20923388, S5_24906893	8.59	3.92	0.08^{np}
Thickness of the Rachis Standard Deviation	TRsd	5	142.93	S5_29271828	S5_27985480, S5_29744120	7.74	3.37	-0.0015cm^{np}
Minimum Primary Branch Length	nPBL	6	0.3	S6_216858	S6_107598, S6_1501183	8.84	4.1	-0.05*
Minimum Primary Branch Length in the Upper Half of the Panicle	nPBLsu	6	0.6	S6_285160	S6_107598, S6_2074873	7.59	5.2	-0.05*
Internode Length	NL	6	66.69	S6_10107446	S6_8821624, S6_12033156	7.85	5.62	-0.03*
Exsertion Length	EL	6	121.49	S6_22141171	S6_21980490, S6_22254152	6.23	4.15	-0.28cm^{np}
Exsertion Length	EL	6	123.31	S6_22260180	S6_22200268, S6_22410496	10.47	6.72	-0.55cm^{np}
Maximum Number of Primary Branches per Internode	xBpN	6	145.99	S6_26691211	S6_26570815, S6_26815270	6.35	4.5	0.01
Maximum Number of Primary Branches per Internode	xBpN	6	150.57	S6_27612798	S6_27114480, S6_27899598	8.9	6.17	0.09
Number of Primary Branches per Internode Standard Deviation	BpNsd	7	21.2	S7_2464872	S7_286127, S7_4138443	6.6	4.03	0.03

Trait Name	Trait Acronym	Chr	Peak (cM)	Peak Marker	Left Marker, Right Marker	PVE	LOD	Add. Eff.
Number of Primary Branches per Internode in the Upper Half of the Panicle	BpNsu	7	21.95	S7_2502087	S7_286127, S7_3274717	7.07	3.63	0.03np
Maximum Exsertion Thickness	xTE	7	22.26	S7_2614196	S7_286127, S7_3274717	8.46	4.64	0.01cm^{np}
Rachis Length	RL	7	22.56	S7_2664270	S7_286127, S7_4683204	4.41	3.66	0.97cm
Primary Branch Number in the Upper Half of the Panicle	PBNsu	7	23.77	S7_2759545	S7_2464872, S7_3274717	6.19	3.94	0.03*
Number of Primary Branches per Internode in the Upper Half of the Panicle Standard Deviation	BpNsusd	7	23.77	S7_2759545	S7_286127, S7_3274717	9.49	7.08	0.02*
Maximum Number of Primary Branches per Internode in the Upper Half of the Panicle	xBpNsu	7	23.77	S7_2759545	S7_286127, S7_3274717	8.18	5.41	0.02*
Panicle Length	PL	7	23.77	S7_2759545	S7_286127, S7_4851150	7.6	3.6	1.14cm
Primary Branch Number	PBN	7	23.77	S7_2759545	S7_2464872, S7_3396611	6.18	4.39	0.38
Total Primary Branch Length	totPBL	7	23.77	S7_2759545	S7_286127, S7_3396611	12.35	6.3	7.47cm
Thickness of the Rachis	TR	7	23.77	S7_2759545	S7_286127, S7_3274717	7.12	3.51	0.0019cm^{np}
Maximum Number of Primary Branches per Internode	xBpN	7	29.05	S7_3274717	S7_2601679, S7_4348779	5.78	4.12	0.09
Primary Branch Length in the Upper Half of the Panicle	PBLsu	8	34.81	S8_4177366	S8_3537285, S8_5708455	11.15	4.88	0.04*
Minimum Primary Branch Length	nPBL	8	39.68	S8_5141914	S8_3537285, S8_5852658	8.91	4.13	0.05*

Trait Name	Trait Acronym	Chr	Peak (cM)	Peak Marker	Left Marker, Right Marker	PVE	LOD	Add. Eff.
Maximum Primary Branch Length in the Upper Half of the Panicle	xPBLsu	8	56.07	S8_9627773	S8_8465714, S8_19468327	8.31	4.3	0.04*
Minimum Primary Branch Length in the Upper Half of the Panicle	nPBLsu	8	69.57	S8_19832020	S8_3537285, S8_21148406	7.1	4.88	0.03*
Exsertion Length	EL	8	83.23	S8_22603837	S8_22484223, S8_23270731	10.59	6.79	-0.88cm^{np}
Minimum Primary Branch Length in the Upper Half of the Panicle	nPBLsu	8	113.08	S8_26929901	S8_26844525, S8_28389376	11.75	7.77	0.06*
Rachis Length	RL	8	115.82	S8_27314672	S8_27213374, S8_28389376	11.05	8.57	-1.68cm
Internode Number in the Upper Half of the Panicle	NNsu	8	115.82	S8_27314672	S8_27213374, S8_27854290	8.07	8.07	-0.36
Primary Branch Number in the Upper Half of the Panicle	PBNsu	8	116.42	S8_27330669	S8_27213374, S8_28134956	10.26	6.32	-0.07*
Internode Number	NN	8	116.73	S8_27381639	S8_26926770, S8_28389376	8.71	4.49	-0.58
Panicle Number	PN	8	117.33	S8_27456412	S8_26762931, S8_28389376	16.08	6.59	1.63
Minimum Primary Branch Number	nPBN	8	120.04	S8_27893736	S8_24768030, S8_28389376	8.73	3.85	-0.85^{np}
Tip Branch Length	tBL	8	120.19	S8_27910097	S8_27301277, S8_28134956	20.5	10.74	0.09*
Primary Branch Number	PBN	8	120.95	S8_28133070	S8_27301277, S8_28389376	6.51	4.61	-0.89
Maximum Primary Branch Number	xPBN	8	122.17	S8_28134956	S8_27626827, S8_28389376	15.61	8.36	-1.06
Thickness of the Rachis Standard Deviation	TRsd	8	122.34	S8_28267215	S8_27185076, S8_28389376	11.77	5.01	-0.0018cm^{np}

Trait Name	Trait Acronym	Chr	Peak (cM)	Peak Marker	Left Marker, Right Marker	PVE	LOD	Add. Eff.
Minimum Primary Branch Length in the Upper Half of the Panicle	nPBLsu	9	87.09	S9_17134585	S9_16971736, S9_17348654	10.17	6.81	0.02*
Internode Length	NL	9	106.66	S9_18985817	S9_16001454, S9_21316402	5.13	3.77	-0.03*
Heading Date	HD	9	119.72	S9_21312838	S9_18772960, S9_21630557	4.6	4.52	-3.43 days^{np}
Minimum Primary Branch Length in the Upper Half of the Panicle	nPBLsu	9	120.02	S9_21375539	S9_20979971, S9_21397964	19.34	12.03	-0.06*
Minimum Internode Length	nNL	9	120.84	S9_21390653	S9_20887955, S9_21635970	12.08	6.8	-0.04*
Primary Branch Length in the Upper Half of the Panicle	PBLsu	9	124.88	S9_21635970	S9_21397964, S9_22503375	13.56	5.85	-0.05*
Maximum Primary Branch Length in the Upper Half of the Panicle	xPBLsu	9	124.88	S9_21635970	S9_21633972, S9_22503375	14.87	7.37	-0.04*
Tip Branch Length	tBL	9	124.9	S9_21841937	S9_21093487, S9_22903307	8.59	4.89	-0.06*
Number of Primary Branches per Internode in the Upper Half of the Panicle Standard Deviation	BpNsusd	10	10.94	S10_2733712	S10_2410677, S10_9903943	4.4	3.45	-0.03*
Rachis Length	RL	10	68.4	S10_18811147	S10_18223900, S10_20363426	5.07	4.18	1.06cm
Internode Length	NL	10	68.4	S10_18813938	S10_18448365, S10_19468486	8.27	5.9	0.03*
Heading Date	HD	10	99.8	S10_22841510	S10_22017290, S10_23124787	8.3	7.8	-3.6 days^{np}
Heading Date	HD	11	15.44	S11_2531514	S11_2457120, S11_2558011	6.98	6.67	2.43 days^{np}

Trait Name	Trait Acronym	Chr	Peak (cM)	Peak Marker	Left Marker, Right Marker	PVE	LOD	Add. Eff.
Heading Date	HD	11	16.36	S11_2561195	S11_2557885, S11_2597906	5.01	4.9	1.69 days^{np}
Minimum Internode Length	nNL	11	35.27	S11_4920784	S11_4362683, S11_5556815	14.16	7.85	0.04*
Internode Number	NN	11	35.27	S11_4920784	S11_2378191, S11_5859540	8.18	4.23	-0.51
Total Primary Branch Length	totPBL	11	36.49	S11_5459296	S11_2588493, S11_6327532	10.52	5.43	-7.93cm
Maximum Total Primary Branch Length	xtotPBL	11	36.49	S11_5529639	S11_2378191, S11_7032038	7.64	3.57	-9.68cm
Internode Number in the Lower Half of the Panicle	NNin	11	38.29	S11_5754289	S11_4103134, S11_5943371	13.19	5.8	-0.37
Number of Primary Branches per Internode in the Upper Half of the Panicle Standard Deviation	BpNsusd	11	115.07	S11_23262087	S11_22736967, S11_23509404	6.58	5.05	-0.03*
Maximum Number of Primary Branches per Internode in the Upper Half of the Panicle	xBpNsu	11	115.07	S11_23262087	S11_21270458, S11_23829029	6.3	4.23	-0.03*

Supplementary Table 6. A priori candidate gene list.

Gene name	chr.	MSUv7_LocID	MSUv7_coordL	MSUv7_coordR
OsYUC4	1	LOC_Os01g12490	6857032	6860962
OsYUC1; OsYUCCA1	1	LOC_Os01g45760	25994669	25996948
OsPIN1c	6	LOC_Os06g12610	6866393	6869521
OsGH3-2	7	LOC_Os07g47490	28400307	28391698
ASP1 (aberrant spikelet and panicle 1)	8	LOC_Os08g06480	3667092	3675813
OsJAG (OPB1; OPEN BEAK)	1	LOC_Os01g03840	1625159	1626771
OsRA2	1	LOC_Os01g07480	3552990	3551044
OsPID-like	1	LOC_Os01g07940	3838211	3835129
OsGA3 oxidase-2 (D18)	1	LOC_Os01g08220	4004946	4003659
SSG4	1	LOC_Os01g08420	4133703	4140633
OsGI1 (GIGANTEA)	1	LOC_Os01g08700	4338485	4329152
D2 (dwarf 2, ebisu dwarf)	1	LOC_Os01g10040	5236623	5244011
GN1 (grain number 1; OsCKX2)	1	LOC_Os01g10110	5275678	5270103
d-h gene (semidwarf)	1	LOC_Os01g10460	5530497	5533609
OsYUC9	1	LOC_Os01g16714	9488825	9493964
OsYUC10	1	LOC_Os01g16750	9512985	9515102
OsGA2 oxidase-2	1	LOC_Os01g22920	12885551	12884474
OsCCD8a (d10-like)	1	LOC_Os01g38580	21669309	21665618
LOG1 (lonely guy-1)	1	LOC_Os01g40630	22959225	22954908
DWT1 (dwarf tiller 1)	1	LOC_Os01g47710	27292681	27288306
OsMAX1 ortholog 1	1	LOC_Os01g50520	29020844	29021337
OsMAX1 ortholog 2	1	LOC_Os01g50580	29039999	29041364
OsBRI1 (O. sativa BRASSINOSTEROID INSENSITIVE 1)	1	LOC_Os01g52050	29931487	29927543
OsYUC3	1	LOC_Os01g53200	30561145	30564493
OsCCD8b (d10)	1	LOC_Os01g54270	31228566	31220321
OsGA2 oxidase-3	1	LOC_Os01g55240	31795105	31797643
LAX1	1	LOC_Os01g61480	35558148	35559225
qSH1	1	LOC_Os01g62920	36449951	36445019
OsGA20 oxidase-2 (SD1)	1	LOC_Os01g66100	38382382	38385504
PLA2 (plastochron2)	1	LOC_Os01g68000	39523638	39527227
EP3 (erect panicle 3)	2	LOC_Os02g15950	9042076	9046141
LARGER PANICLE 1 (LP1)	2	LOC_Os02g15950	9042076	9046141
OsYUC12	2	LOC_Os02g17230	9877605	9879276
OsCPS1 (ent-copalyl diphosphate synthase 1)	2	LOC_Os02g17780	10283727	10273718
FOS1 (FON2 SPARE1)	2	LOC_Os02g21890	13014468	13015693
RCN2 (rice TFL1/CENTRORADIALIS (CEN)-like-	2	LOC_Os02g32950	19568968	19567632

Gene name	chr.	MSUv7_LocID	MSUv7_coordL	MSUv7_coordR
2) **not to be confused with Reducedculmnumber				
GID2	2	LOC_Os02g36974	22333281	22337713
OsMADS57	2	LOC_Os02g49840	30456666	30462759
BRITTLE CULM3 (BC3)	2	LOC_Os02g50550	30871171	30861491
OsPIN1; REH1	2	LOC_Os02g50960	31162062	31158903
COP1	2	LOC_Os02g53140	32533583	32528037
SMG1 (small grain 1)	2	LOC_Os02g54600	33443948	33442069
OsFD3	2	LOC_Os02g58670	35854731	35853347
EHD4 (early heading date 4)	3	LOC_Os03g02160	717447	720837
OsMADS50	3	LOC_Os03g03100	1300384	1298070
TAD; TE	3	LOC_Os03g03150	1327397	1331210
NAL7; OsYUC8	3	LOC_Os03g06654	3355041	3360485
D14	3	LOC_Os03g10620	5422148	5426577
DL (drooping leaf)	3	LOC_Os03g11600	6041245	6048687
LEAFY HULL STERILE 1 (LHS1)	3	LOC_Os03g11614	6061369	6052750
D4 (dwarf4)	3	LOC_Os03g12660	6737549	6744506
SSD1 (sword shape dwarf1)	3	LOC_Os03g19080	10684315	10688955
BRITTLE CULM1 (BC1)	3	LOC_Os03g30250	17260196	17262960
FC1 (FINE CULM1), also known as OsTB1 (O. sativa TEOSINTEBRANCHED1)	3	LOC_Os03g49880	28428504	28430462
SLR1 (SLENDER RICE 1)	3	LOC_Os03g49990	28512625	28515179
OASB2	3	LOC_Os03g50880	29071552	29067747
OSH1 (~7 OSH genes with overlapping functions)	3	LOC_Os03g51690	29606861	29596277
OsMADS14	3	LOC_Os03g54160	31041569	31031146
PAP2 (panicle phytomer-2) (OsMADS34)	3	LOC_Os03g54170	31055019	31048351
GASR1 (GA-stimulated transcript-related gene 1)	3	LOC_Os03g55290	31465625	31464840
HD6	3	LOC_Os03g55389	31514460	31508811
DST (DROUGHT AND SALT TOLERANCE)	3	LOC_Os03g57240	32647051	32645456
HD16 (early flowering1, EF1)	3	LOC_Os03g57940	32999502	33006898
OsIDS1 (O. sativa INDETERMINATE SPIKELET 1)	3	LOC_Os03g60430	34358193	34362334
OsGA20 oxidase-1	3	LOC_Os03g63970	36150664	36152517
OsYUC7	4	LOC_Os04g03980	1816073	1813950
OsRA1	4	LOC_Os04g08600	4678833	4674744
OsTS2 (rice ortholog tasselseed-2)	4	LOC_Os04g10000	5381565	5380095
An-1 (awn-1)	4	LOC_Os04g28280	16734806	16732393
LAX PANICLE2 (LAX2)	4	LOC_Os04g32510	19564239	19566320
TDD1 (tryptophan deficient dwarf); OASB1	4	LOC_Os04g38950	23142821	23139158
GASR2 (GA-stimulated transcript-related gene 2)	4	LOC_Os04g39110	23243202	23242283
DWARF11 (D11, shinkane-aikoku)	4	LOC_Os04g39430	23471594	23467128
OsGA2 oxidase-6	4	LOC_Os04g44150	26134356	26130707

Gene name	chr.	MSUv7_LocID	MSUv7_coordL	MSUv7_coordR
TOB1 (Tongari-Boushi 1)	4	LOC_Os04g45330	26800253	26797650
HTD1 (high-tillering dwarf 1); (also known as OsCCD7, D17)	4	LOC_Os04g46470	27567824	27570926
OsFD4	4	LOC_Os04g47270	28070371	28067096
APO2 (aberrant panicle organization-2; also known as RFL)	4	LOC_Os04g51000	30185852	30182589
AP2-domain containing protein	4	LOC_Os04g52090	30939177	30940275
OsKS1	4	LOC_Os04g52230	31026617	31031248
OsMADS31	4	LOC_Os04g52410	31143083	31144886
SPIKE (NAL1)	4	LOC_Os04g52479	31203525	31214741
OsLG1 (SPR3 locus)	4	LOC_Os04g56170	33488512	33492876
SHA1 (shattering 1)	4	LOC_Os04g57530	34233373	34231186
OsRR6	4	LOC_Os04g57720	34375978	34377357
OsGA2 oxidase-1	5	LOC_Os05g06670	3459616	3465991
OsGA3 oxidase-1	5	LOC_Os05g08540	4662589	4660662
D2 homolog (dwarf 2, ebisu dwarf)	5	LOC_Os05g11130	6267906	6273074
DWARF1 (dwarf 1, RGA)	5	LOC_Os05g26890	15613483	15609844
GID1L2 (gibberellin insensitive dwarf 1; GID1)	5	LOC_Os05g33730	19868419	19871283
OsGA20 oxidase-4	5	LOC_Os05g34854	20692274	20685346
OsLAC	5	LOC_Os05g38420	22533536	22536101
ELONGATED UPPERMOST INTERNODE 1 (EUI1)	5	LOC_Os05g40384	23728502	23738405
OsGA2 oxidase-4	5	LOC_Os05g43880	25516204	25515053
OsYUC2	5	LOC_Os05g45240	26263760	26261275
OsKAO1 (dwarf 3)	6	LOC_Os06g02019	580665	586450
DLT (D62, OsGRAS62)	6	LOC_Os06g03710	1468600	1465499
StarchSynthase		LOC_Os06g04200	1766194	1770656
D3 (dwarf 3, dwarf bunketsuwaito tillering)	6	LOC_Os06g06050	2780715	2785271
RFT1 (RICE FLOWERING LOCUS T 1, RFT1/FT-L3)	6	LOC_Os06g06300	2926823	2928474
HD3a (Heading date 3a)	6	LOC_Os06g06320	2940004	2942452
Hd1 (heading date 1)	6	LOC_Os06g16370	9336359	9338643
OsKO2 (D35)	6	LOC_Os06g37300	22028146	22020840
MOC1 (SPA1=allele)	6	LOC_Os06g40780	24311420	24316382
APO1 (aberrant panicle organization-1) (also known as UFO)	6	LOC_Os06g45460	27481387	27480082
DENSE AND ERECT PANICLE 3 (DEP3)	6	LOC_Os06g46350	28110979	28109236
OsLIC1	6	LOC_Os06g49080	29738880	29742503
SPW1 (SUPERWOMAN1); also known as OsMADS16	6	LOC_Os06g49840	30177996	30173485
FON1 (floral organ number 1)	6	LOC_Os06g50340	30474473	30470045
OsFD6	6	LOC_Os06g50480	30564131	30567036
OsFD2	6	LOC_Os06g50600	30624219	30620816
OsFD5	6	LOC_Os06g50830	30758412	30754644
OsGA2 oxidase-5	7	LOC_Os07g01340	213778	219544

Gene name	chr.	MSUv7_LocID	MSUv7_coordL	MSUv7_coordR
OsMADS15	7	LOC_Os07g01820	477655	471174
G1 (long sterile lemma)	7	LOC_Os07g04670	2068174	2067344
PROG1 (prostrate growth 1)	7	LOC_Os07g05900	2839979	2839476
OsGA20 oxidase-3	7	LOC_Os07g07420	3702287	3705030
SNB (SUPERNUMERARY BRACT)	7	LOC_Os07g13170	7545486	7550141
GHD7 (grain number, plant height, and heading date 7)	7	LOC_Os07g15770	9155185	9152402
OsYUC6	7	LOC_Os07g25540	14620501	14618203
Oshox1 (rice vrs1)	7	LOC_Os07g39280	23515632	23511899
OsMADS18	7	LOC_Os07g41370	24788476	24793884
DENSE AND ERECT PANICLE 2 (DEP2)	7	LOC_Os07g42410	25381698	25389547
SRA (sister of RA3)	7	LOC_Os07g43160	25867212	25871233
FZP (frizzy panicle)	7	LOC_Os07g47330	28301089	28299591
Ehd3	8	LOC_Os08g01420	276978	272854
GHD8	8	LOC_Os08g07740	4335434	4333717
OsBAK1	8	LOC_Os08g07760	4344171	4350502
SPL14 (SQUAMOSA PROMOTER BINDING PROTEIN-LIKE1, also known as WFP (wealthy farmer's panicle) and IPA1 (ideal plant architecture 1)	8	LOC_Os08g39890	25278696	25274449
SPL16 (QTL GW8)	8	LOC_Os08g41940	26501167	26506218
OsTRX1	9	LOC_Os09g04890	2615934	2601650
EMF2b (embryonic flower 2b)	9	LOC_Os09g13630	7938161	7925560
OsCPS3 ((ent-copalyl diphosphate synthase 3)	9	LOC_Os09g15050	9096555	9101314
DEP1 (DENSE AND ERECT PANICLE 1); also identified as EP	9	LOC_Os09g26999	16411151	16415862
OsFD1	9	LOC_Os09g36910	21291988	21293452
PLA1 (plastochron1)	10	LOC_Os10g26340	13660543	13658790
Ehd2; OsID1; RID1; Ghd10	10	LOC_Os10g28330	14742745	14739569
Ehd1	10	LOC_Os10g32600	17077979	17076098
TAW1 (TAWAWA 1)	10	LOC_Os10g33780	17889724	17888297
D53	11	LOC_Os11g01330	194176	199479
OsPIN1b	11	LOC_Os11g04190	1705477	1702178
RCN1 (rice TFL1/CENTRORADIALIS (CEN)-like-1) **not to be confused with Reducedculmnumber	11	LOC_Os11g05470	2454103	2452525
OsYUC14	11	LOC_Os11g10170	5514584	5516300
SP1 (short panicle-1)	11	LOC_Os11g12740	7198552	7193230
FON2 (floral organ number 2) (FON4 = allele)	11	LOC_Os11g38270	22664995	22663457
OsYUC11	12	LOC_Os12g08780	4517346	4515673
OsDEC1	12	LOC_Os12g27994	16504887	16500199
OsYUC5	12	LOC_Os12g32750	19779505	19786587
OsPID (BIF2)	12	LOC_Os12g42020	26055221	26057002
OsMADS3 - MADS-box family gene with MIKCc	1	LOC_Os01g10504	5559548	5568844

Gene name	chr.	MSUv7_LocID	MSUv7_coordL	MSUv7_coordR
type-box, expressed				
OsMADS91 - MADS-box family gene with M-beta type-box, expressed	1	LOC_Os01g11510	6191931	6193745
OsMADS88 - MADS-box family gene with M-gamma type-box, expressed	1	LOC_Os01g18420	10352429	10353151
OsMADS89 - MADS-box family gene with M-gamma type-box, expressed	1	LOC_Os01g18440	10366411	10367331
OsMADS92 - MADS-box family gene with M-beta type-box, expressed	1	LOC_Os01g23750	13360117	13361337
OsMADS93 - MADS-box family gene with M-beta type-box, expressed	1	LOC_Os01g23760	13363707	13364930
OsMADS94 - MADS-box family gene with M-beta type-box, expressed	1	LOC_Os01g23770	13366416	13367710
OsMADS95 - MADS-box family gene with M-beta type-box, expressed	1	LOC_Os01g23780	13373620	13374665
OsMADS32 - MADS-box family gene with MIKCc type-box, expressed	1	LOC_Os01g52680	30278492	30280577
OsMADS2 - MADS-box family gene with MIKCc type-box, expressed	1	LOC_Os01g66030	38320785	38324074
OsMADS21 - MADS-box family gene with MIKCc type-box, expressed	1	LOC_Os01g66290	38500880	38505127
OsMADS96 - MADS-box family gene with M-beta type-box, expressed	1	LOC_Os01g67890	39459599	39461050
OsMADS97 - MADS-box family gene with M-beta type-box, expressed	1	LOC_Os01g68420	39762896	39763714
OsMADS98 - MADS-box family gene with M-beta type-box, expressed	1	LOC_Os01g68560	39826794	39828732
OsMADS65 - MADS-box family gene with MIKC* type-box, expressed	1	LOC_Os01g69850	40344329	40364584
OsMADS79 - MADS-box family gene with M-alpha type-box, expressed	1	LOC_Os01g74440	43112781	43113407
OsMADS60 - MADS-box family gene with MIKCc type-box, expressed	2	LOC_Os02g01360	205424	207508
OsMADS80 - MADS-box family gene with M-alpha type-box, expressed	2	LOC_Os02g06860	3459445	3460305
OsMADS29 - MADS-box family gene with MIKCc type-box, expressed	2	LOC_Os02g07430	3833129	3837135
OsMADS27 - MADS-box family gene with MIKCc type-box, expressed	2	LOC_Os02g36924	22294657	22301808
OsMADS6 - MADS-box family gene with MIKCc type-box, expressed	2	LOC_Os02g45770	27875979	27884079
OsMADS57 - MADS-box family gene with MIKCc type-box, expressed	2	LOC_Os02g49840	30456666	30462759
OsMADS22 - MADS-box family gene with MIKCc type-box, expressed	2	LOC_Os02g52340	32038902	32045130
OsMADS50 - MADS-box family gene with MIKCc type-box, expressed	3	LOC_Os03g03100	1298070	1300384

Gene name	chr.	MSUv7_LocID	MSUv7_coordL	MSUv7_coordR
OsMADS47 - MADS-box family gene with MIKCc type-box, expressed	3	LOC_Os03g08754	4519405	4525778
OsMADS72 - MADS-box family gene with M-alpha type-box, expressed	3	LOC_Os03g14850	8096539	8098764
OsMADS86 - MADS-box family gene with M-gamma type-box, expressed	3	LOC_Os03g37670	20891504	20895054
OsMADS87 - MADS-box family gene with M-gamma type-box, expressed	3	LOC_Os03g38610	21428521	21429270
OsMADS14 - MADS-box family gene with MIKCc type-box, expressed	3	LOC_Os03g54160	31031146	31041569
OsMADS34 - MADS-box family gene with MIKCc type-box, expressed	3	LOC_Os03g54170	31048351	31055019
OsMADS25 - MADS-box family gene with MIKCc type-box, expressed	4	LOC_Os04g23910	13672710	13675884
OsMADS81 - MADS-box family gene with M-gamma type-box, expressed	4	LOC_Os04g24790	14239637	14240295
OsMADS82 - MADS-box family gene with M-gamma type-box, expressed	4	LOC_Os04g24800	14243635	14244264
OsMADS83 - MADS-box family gene with M-gamma type-box	4	LOC_Os04g24810	14259164	14259793
OsMADS84 - MADS-box family gene with M-gamma type-box, expressed	4	LOC_Os04g25870	15035746	15036375
OsMADS85 - MADS-box family gene with M-gamma type-box, expressed	4	LOC_Os04g25920	15078802	15079431
OsMADS99 - MADS-box family gene with M-gamma type-box	4	LOC_Os04g25930	15082804	15083433
OsMADS64 - MADS-box family gene with M-alpha type-box, expressed	4	LOC_Os04g31804	19042703	19058654
OsMADS61 - MADS-box family gene with MIKCc type-box, expressed	4	LOC_Os04g38770	23040160	23041485
OsMADS17 - MADS-box family gene with MIKCc type-box, expressed	4	LOC_Os04g49150	29308245	29314112
OsMADS31 - MADS-box family gene with MIKCc type-box, expressed	4	LOC_Os04g52410	31143083	31144886
OsMADS66 - MADS-box family gene with MIKCc type-box, expressed	5	LOC_Os05g11380	6426374	6434650
OsMADS58 - MADS-box family gene with MIKCc type-box, expressed	5	LOC_Os05g11414	6447183	6458100
OsMADS70 - MADS-box family gene with M-alpha type-box, expressed	5	LOC_Os05g23780	13656346	13657002
OsMADS4 - MADS-box family gene with MIKCc type-box, expressed	5	LOC_Os05g34940	20759446	20762662
OsMADS5 - MADS-box family gene with MIKCc type-box, expressed	6	LOC_Os06g06750	3162801	3169415
OsMADS55 - MADS-box family gene with MIKCc type-box, expressed	6	LOC_Os06g11330	5953593	5963184
OsMADS63 - MADS-box family gene with	6	LOC_Os06g11970	6372353	6376374

Gene name	chr.	MSUv7_LocID	MSUv7_coordL	MSUv7_coordR
MIKC* type-box, expressed				
OsMADS71 - MADS-box family gene with M-alpha type-box, expressed	6	LOC_Os06g22760	13244910	13245626
OsMADS59 - MADS-box family gene with MIKCc type-box	6	LOC_Os06g23950	13996414	13997319
OsMADS75 - MADS-box family gene with M-alpha type-box, expressed	6	LOC_Os06g30810	17869243	17869875
OsMADS76 - MADS-box family gene with M-alpha type-box, expressed	6	LOC_Os06g30830	17887762	17891217
OsMADS30 - MADS-box family gene with MIKCc type-box, expressed	6	LOC_Os06g45650	27637555	27641578
OsMADS16 - MADS-box family gene with MIKCc type-box, expressed	6	LOC_Os06g49840	30173485	30178066
OsMADS15 - MADS-box family gene with MIKCc type-box, expressed	7	LOC_Os07g01820	471174	477655
OsMADS90 - MADS-box family gene with M-beta type-box, expressed	7	LOC_Os07g04170	1781625	1785426
OsMADS18 - MADS-box family gene with MIKCc type-box, expressed	7	LOC_Os07g41370	24788476	24793884
OsMADS26 - MADS-box family gene with MIKCc type-box, expressed	8	LOC_Os08g02070	679358	681739
OsMADS23 - MADS-box family gene with MIKCc type-box, expressed	8	LOC_Os08g33488	20897215	20906881
OsMADS62 - MADS-box family gene with MIKC* type-box, expressed	8	LOC_Os08g38590	24391630	24393867
OsMADS7 - MADS-box family gene with MIKCc type-box, expressed	8	LOC_Os08g41950	26507180	26512261
OsMADS37 - MADS-box family gene with MIKC* type-box, expressed	8	LOC_Os08g41960	26518976	26529016
OsMADS77 - MADS-box family gene with M-alpha type-box, expressed	9	LOC_Os09g02780	1272405	1272941
OsMADS78 - MADS-box family gene with M-alpha type-box, expressed	9	LOC_Os09g02830	1325181	1325807
OsMADS8 - MADS-box family gene with MIKCc type-box, expressed	9	LOC_Os09g32948	19653389	19659766
OsMADS56 - MADS-box family gene with MIKCc type-box, expressed	10	LOC_Os10g39130	20862952	20873797
OsMADS68 - MADS-box family gene with MIKC* type-box, expressed	11	LOC_Os11g43740	26414394	26418442
OsMADS33 - MADS-box family gene with MIKCc type-box, expressed	12	LOC_Os12g10520	5569433	5573026
OsMADS13 - MADS-box family gene with MIKCc type-box, expressed	12	LOC_Os12g10540	5584593	5590285
OsMADS73 - MADS-box family gene with M-alpha type-box, expressed	12	LOC_Os12g21850	12303478	12304062
OsMADS74 - MADS-box family gene with M-alpha type-box, expressed	12	LOC_Os12g21880	12319519	12320281

Gene name	chr.	MSUv7_LocID	MSUv7_coordL	MSUv7_coordR
OsMADS20 - MADS-box family gene with MIKCc type-box, expressed	12	LOC_Os12g31748	19106070	19115282
WRKY1	1	LOC_Os01g14440	8084372	8087044
WRKY9	1	LOC_Os01g18584	10470540	10478800
WRKY10	1	LOC_Os01g09100	4572448	4573409
WRKY11	1	LOC_Os01g43650	25009453	25012236
WRKY12	1	LOC_Os01g43550	24945282	24947296
WRKY13	1	LOC_Os01g54600	31409004	31410978
WRKY14	1	LOC_Os01g53040	30480134	30482161
WRKY15	1	LOC_Os01g46800	26687377	26688416
WRKY16	1	LOC_Os01g47560	27196417	27200990
WRKY17	1	LOC_Os01g74140	42946753	42948750
WRKY18	1	LOC_Os01g60520	34996792	35003226
WRKY20	1	LOC_Os01g60540	35008866	35011098
WRKY21	1	LOC_Os01g60640	35062734	35064940
WRKY22	1	LOC_Os01g60490	34981468	34985447
WRKY23	1	LOC_Os01g53260	30604295	30608077
WRKY24	1	LOC_Os01g61080	35347978	35350645
WRKY26	1	LOC_Os01g51690	29720923	29723065
WRKY27	1	LOC_Os01g40430	22824809	22827116
WRKY77	1	LOC_Os01g40260	22731943	22733240
WRKY79	1	LOC_Os01g08710	4340849	4356468
WRKY86	1	LOC_Os01g60600	35032511	35035321
WRKY97	1	LOC_Os01g09080	4566304	4568617
WRKY32	2	LOC_Os02g53100	32489017	32495070
WRKY34	2	LOC_Os02g43560	26280253	26283914
WRKY39	2	LOC_Os02g16540	9446492	9448847
WRKY42	2	LOC_Os02g26430	15521497	15522724
WRKY66	2	LOC_Os02g47060	28726783	28730933
WRKY71	2	LOC_Os02g08440	4542762	4544983
WRKY3	3	LOC_Os03g55080	31323190	31326926
WRKY4	3	LOC_Os03g55164	31391390	31399050
WRKY6	3	LOC_Os03g58420	33286651	33288563
WRKY44	3	LOC_Os03g21710	12394669	12396898
WRKY55a	3	LOC_Os03g20550	11650824	11652144
WRKY55b	3	LOC_Os03g20550	11650824	11652144
WRKY60	3	LOC_Os03g45450	25651039	25652125
WRKY87	3	LOC_Os03g53050	30422191	30425543
WRKY88	3	LOC_Os03g63810	36039164	36043822
WRKY35	4	LOC_Os04g39570	23579837	23587188
WRKY36	4	LOC_Os04g46060	27284275	27290983
WRKY37	4	LOC_Os04g50920	30132491	30136547
WRKY68	4	LOC_Os04g51560	30545175	30546577

Gene name	chr.	MSUv7_LocID	MSUv7_coordL	MSUv7_coordR
WRKY5	5	LOC_Os05g04640	2179520	2184940
WRKY7	5	LOC_Os05g46020	26682472	26684208
WRKY8	5	LOC_Os05g50610	29009136	29013172
WRKY19	5	LOC_Os05g49620	28471802	28473061
WRKY43	5	LOC_Os05g49210	28238562	28241041
WRKY45	5	LOC_Os05g25770	14991579	14993800
WRKY48	5	LOC_Os05g40060	23529423	23530499
WRKY49	5	LOC_Os05g49100	28154693	28157989
WRKY53	5	LOC_Os05g27730	16150266	16152747
WRKY54	5	LOC_Os05g40080	23550611	23551716
WRKY58	5	LOC_Os05g45230	26256951	26257809
WRKY67	5	LOC_Os05g09020	4998210	4999629
WRKY70	5	LOC_Os05g39720	23310474	23313449
WRKY89a	5	LOC_Os05g03900	1743366	1745281
WRKY89b	5	LOC_Os05g03900	1743366	1745281
WRKY90	5	LOC_Os05g14370	8086892	8088575
WRKY91	5	LOC_Os05g40070	23536113	23539013
WRKY92	5	LOC_Os05g50700	29064582	29065815
WRKY28	6	LOC_Os06g44010	26509919	26511699
WRKY31	6	LOC_Os06g30860	17915923	17917083
WRKY73	6	LOC_Os06g05380	2429149	2433254
WRKY93	6	LOC_Os06g06360	2958991	2963006
WRKY29	7	LOC_Os07g02060	630562	634316
WRKY47	7	LOC_Os07g48260	28828793	28832398
WRKY78	7	LOC_Os07g39480	23654076	23659625
WRKY94	7	LOC_Os07g17230	10155452	10159775
WRKY95	7	LOC_Os07g27670	16147913	16149483
WRKY96	7	LOC_Os07g40570	24311898	24315383
WRKY25	8	LOC_Os08g13840	8258454	8259597
WRKY30	8	LOC_Os08g38990	24645860	24649860
WRKY69	8	LOC_Os08g29660	18220041	18222408
WRKY82	8	LOC_Os08g17400	10633195	10639603
WRKY62	9	LOC_Os09g25070	14992803	14994888
WRKY74	9	LOC_Os09g16510	10128825	10131136
WRKY76	9	LOC_Os09g25060	14975932	14977713
WRKY80	9	LOC_Os09g30400	18496949	18501264
WRKY98	9	LOC_Os09g09630	5187963	5190200
WRKY2	10	LOC_Os10g42850	23107503	23108646
WRKY40	11	LOC_Os11g02530	787542	789030
WRKY41	11	LOC_Os11g45920	27779324	27785982
WRKY46a	11	LOC_Os11g02480	759587	763334
WRKY50	11	LOC_Os11g02540	793116	795608
WRKY52	11	LOC_Os11g02470	749998	751210

Gene name	chr.	MSUv7_LocID	MSUv7_coordL	MSUv7_coordR
WRKY59	11	LOC_Os11g45750	27683640	27695070
WRKY61	11	LOC_Os11g45850	27740142	27741375
WRKY72	11	LOC_Os11g29870	17352085	17355820
WRKY100	11	LOC_Os11g02520	782358	783511
WRKY46b	12	LOC_Os12g02420	802489	806097
WRKY56	12	LOC_Os12g02440	817003	823003
WRKY57	12	LOC_Os12g01180	100799	104008
WRKY64	12	LOC_Os12g02450	824302	825793
WRKY65	12	LOC_Os12g02470	836516	838755
WRKY81	12	LOC_Os12g02400	789403	790687
WRKY83	12	LOC_Os12g40570	25100479	25104175
WRKY85	12	LOC_Os12g32250	19473728	19478606

Supplementary Note

The three most significant peaks in the study co-localized with candidate genes within the gibberellin signaling cascade and were identified when mapping for agronomic traits: *GID1 (GIBBERELLIN INSENSITIVE DWARF1),* a soluble gibberellin receptor[1] associated with booting; *SD1* (*SEMIDWARF 1*), a OsGA20-oxidase gibberellin biosynthesis enzyme associated with plant height [2]; and *OsGA2ox-5*, a gibberellin degradation enzyme[3] associated with total shoot biomass (Supplementary Figs. 62-64).

We detected five transcription factors associated with panicle phenotypes. Two of these genes, *ERECT PANICLE 3* (*EP3*, also known as *LARGER PANICLE*) and *BRASSINAZOLE RESISTANT 1* (*BZR1*), were detected when mapping for panicle traits across all varieties in the panel and are transcriptional regulators of the cytokinin and brassinosteroid signaling pathways, respectively[4-6]. *EP3* shared a coexpression network with *DENSE AND ERECT PANICLE 1* (Supplementary Fig. 74), a G-protein with alleles that have been linked to internode length phenotypes[7]. *BZR1* shared a coexpression network with *FLORAL ORGAN NUMBER 1*[8], a member of the *CLAVATA* signaling cascade known to be involved in floral meristem differentiation, and *SHORT PANICLE 1*, a putative nitrogen transporter of unknown function that ubiquitously affects panicle phenotypes when knocked out[9] (Supplementary Fig. 80).

The *FRIZZY PANICLE* (*FZP*) gene, a well characterized transcriptional regulator of primary branch meristem determinancy[10], was detected when mapping across all varieties for the agronomic phenotype secondary branch number (Supplementary Fig. 60). Within *tropical japonica*, we detected a subpopulation specific peak for several primary branch length traits on chromosome 10 (Supplementary Figs. 21-23, 67),

encompassing the transcription factor *WRKY2*[11]. The WRKY gene family regulates a range of biological processes in plants, including disease resistance, drought tolerance, and development [11,12], and one other member in rice has been linked with panicle exsertion and seed formation[13]. Interestingly, *WRKY2* shares a gene coexpression network with multiple genes linked to heading date, flowering, and panicle phenotypes (Supplementary Fig. 82), which is consistent with the quantitative trait locus (QTL) we detect for heading date surrounding *WRKY2* in the recombinant inbred line (RIL) population (Supplementary Fig. 92).

Within the *aus* subpopulation we identified two genes with overlapping associations for different types of traits. The first, *CYP90D3*, was associated with maximum internode length and maximum primary branch length in the upper half of the panicle (Supplementary Fig. 10, 22). *CYP90D3* is a homolog of *D2* (*DWARF EBISU*), which encodes a cytochrome P450 brassinosteroid biosynthesis enzyme that has been linked with subtle changes in plant architecture and panicle phenotypes[14]. A recent study demonstrated that CYP90D2 and CYP90D3 catalyze many of the same reactions *in vitro*[15], suggesting that *CYP90D3* may pleiotropically affect multiple panicle phenotypes similar to its homolog. Interestingly, *CYP90D3* shares a coexpression network with *OsMADS57* (Supplementary Fig. 77), a rice MADS-box gene that regulates *DWARF 14* (*D14*) by forming a complex with rice *TEOSINTE BRANCHED 1* (*OsTB1*); this pathway has been shown to impact vegetative tillering, leading to changes in the number and size of panicles[16]. Finally, maximum internode length was associated with a second hormone gene in *aus*, rice ent-kaurene synthase (*OsKS1*) (Figure 7). *OsKS1* catalyzes an early step in gibberellin biosynthesis and is tandemly linked with two of its homologs on

chromosome 4 (*OsKS2*, *OsKS3*)[3], and is discussed in detail within the main body of the paper.

Supplementary References

1. Ueguchi-Tanaka, M. *et al.* GIBBERELLIN INSENSITIVE DWARF1 encodes a soluble receptor for gibberellin. *Nature* **437,** 693–698 (2005).
2. Ashikari, M. *et al.* Loss-of-function of a rice gibberellin biosynthetic gene, GA20 oxidase (GA20ox-2), led to the rice'green revolution'. *Breed. Sci.* **52,** 143–150 (2002).
3. Sakamoto, T. *et al.* An overview of gibberellin metabolism enzyme genes and their related mutants in rice. *Plant Physiology* **134,** 1642–1653 (2004).
4. Bai, M.-Y. *et al.* Functions of OsBZR1 and 14-3-3 proteins in brassinosteroid signaling in rice. *Proceedings of the National Academy of Sciences* **104,** 13839–13844 (2007).
5. Piao, R. *et al.* Map-based cloning of the ERECT PANICLE 3 gene in rice. *TAG Theoretical and Applied Genetics* **119,** 1497–1506 (2009).
6. Li, M. *et al.* Mutations in the F-box gene LARGER PANICLE improve the panicle architecture and enhance the grain yield in rice. *Plant Biotechnology Journal* no–no (2011). doi:10.1111/j.1467-7652.2011.00610.x
7. Huang, X. *et al.* Natural variation at the DEP1 locus enhances grain yield in rice. *Nat Genet* **41,** 494–497 (2009).
8. Suzaki, T. The gene FLORAL ORGAN NUMBER1 regulates floral meristem size in rice and encodes a leucine-rich repeat receptor kinase orthologous to Arabidopsis CLAVATA1. *Development* **131,** 5649–5657 (2004).
9. Li, S. *et al.* Short panicle1encodes a putative PTR family transporter and determines rice panicle size. *The Plant Journal* **58,** 592–605 (2009).
10. Komatsu, M. FRIZZY PANICLE is required to prevent the formation of axillary meristems and to establish floral meristem identity in rice spikelets. *Development* **130,** 3841–3850 (2003).
11. Ross, C. A., Liu, Y. & Shen, Q. J. The WRKY gene family in rice (Oryza sativa). *J. Integr. Plant Biol.* **49,** 827–842 (2007).
12. Rushton, P. J., Somssich, I. E., Ringler, P. & Shen, Q. J. WRKY transcription factors. *Trends in Plant Science* **15,** 247–258 (2010).
13. Zhang, C.-Q. *et al.* The WRKY transcription factor OsWRKY78 regulates stem elongation and seed development in rice. *Planta* **234,** 541–554 (2011).
14. Hong, Z. A Rice Brassinosteroid-Deficient Mutant, ebisu dwarf (d2), Is Caused by a Loss of Function of a New Member of Cytochrome P450. *The Plant Cell Online* **15,** 2900–2910 (2003).
15. Sakamoto, T., Ohnishi, T., Fujioka, S., Watanabe, B. & Mizutani, M. Rice CYP90D2 and CYP90D3 catalyze C-23 hydroxylation of brassinosteroids in vitro. *Plant Physiol. Biochem.* **58,** 220–226 (2012).
16. Guo, S. *et al.* The interaction between OsMADS57 and OsTB1 modulates rice tillering via DWARF14. *Nature Communications* **4,** 1566–12 (2013).
